# A New Calibrated Bayesian Internal Goodness-of-Fit Method: Sampled Posterior p-Values as Simple and General p-Values That Allow Double Use of the Data

**DOI:** 10.1371/journal.pone.0014770

**Published:** 2011-03-18

**Authors:** Frédéric Gosselin

**Affiliations:** Cemagref, UR EFNO, Nogent-sur-Vernisson, France; Cuban Neuroscience Center, Cuba

## Abstract

**Background:**

Recent approaches mixing frequentist principles with Bayesian inference propose internal goodness-of-fit (GOF) p-values that might be valuable for critical analysis of Bayesian statistical models. However, GOF p-values developed to date only have known probability distributions under restrictive conditions. As a result, no known GOF p-value has a known probability distribution for any discrepancy function.

**Methodology/Principal Findings:**

We show mathematically that a new GOF p-value, called the sampled posterior p-value (SPP), asymptotically has a uniform probability distribution whatever the discrepancy function. In a moderate finite sample context, simulations also showed that the SPP appears stable to relatively uninformative misspecifications of the prior distribution.

**Conclusions/Significance:**

These reasons, together with its numerical simplicity, make the SPP a better canonical GOF p-value than existing GOF p-values.

## Introduction

Statistical model criticism, which tests a fitted statistical parametric model against observed data, is valuable for gaining more confidence in the statistical results [1]-[5]. Box [6] identified model criticism as one of the two main steps in statistical model development. Although many other terms have been used – model adequacy, model checking, model validation, model evaluation [3], [5] –, we will use the term goodness-of-fit to refer to this confrontation between statistical model and observed data. To date, the generally preferred method has been *external* goodness-of-fit, where data used to assess the model are not those used to fit the model. The evaluation is performed either through data splitting or by comparing the model predictions against a completely different dataset [5]. External goodness-of-fit avoids using the data twice, and should result in more interpretable and less circular goodness-of-fit [7], [8]. However, many researchers have proposed *internal* goodness-of-fit methods (see later), where predictions from the fitted model are compared with the observations that were used to estimate the parameters of the model. One obvious advantage of internal goodness-of-fit (GOF) is to allow fuller use of data in model checking. We will therefore focus our attention on these methods, and more precisely on GOF p-values. The GOF p-values we use are Fisherian p-values, i.e. probabilities of “seeing something [with the statistical model] as weird or weirder than you actually saw” [9]. Fisherian p-values compare the model to the data, and therefore differ from Neyman-Pearson tests which compare two models or hypotheses [9]. “Weirdness” is quantified using specific discrepancy functions, which are real-valued functions of data and of statistical model parameters. Fisherian p-values are simply calculated as the quantile of the discrepancy function calculated on the observed data in the probability distribution of discrepancy functions of data and parameters randomly generated according to some given probabilistic scheme associated to the fitted statistical model. Let us assume that, when replicating over hypothetical datasets sampled from a probabilistic model, we know these p-values have a uniform distribution on 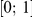 under assumption (A1):

(A1) the likelihood in the statistical model – or inference model, used to analyze data – is the same as the likelihood in the probabilistic model – or sampling model, used to generate data; then an extreme Fisherian p-value – i.e. a p-value very close to 0 or a p-value either very close to 0 or to 1, depending on the discrepancy function – is interpreted as contradicting (A1). The reader will find the mathematical formulation of these statements at the beginning of the Material & Methods section.

When the statistical model is fitted with Bayesian methods, these GOF p-values clearly rely on both Bayesian and frequentist ideas: they are Bayesian because the statistical parameters come either from the prior or the posterior distribution, or modifications thereof, and they are frequentist because they embed the observed data within a set of unobserved datasets sampled from a probabilistic model. This is why such methods are called calibrated Bayesian [10]. Calibrated Bayesian GOF has progressively gained popularity over the last few decades, resulting in a number of more or less sophisticated techniques [6], [11]-[18]. Calibrated Bayesian GOF differ from classical purely Bayesian methods that specify a family of alternative, more complex models and use Bayes Factors to indicate which family of models – the original or the alternative models – is the most likely [6], [19]. Even though this purely Bayesian method does have some interesting features (e.g. discussion in [13]), it cannot deal with the Fisherian view of model checking, i.e. testing whether the data are consistent with a given model, without the need for an alternative hypothesis [9], [10], [20]. What if both the original and the alternative models were inconsistent with the data? Huber [19] qualifies these purely Bayesian procedures as ‘tentative overfitting’, commenting that these Bayesian methods “are based on the unwarranted presumption that by throwing in a few additional parameters one can obtain a perfectly fitting model. But how and where to insert those additional parameters often is far from obvious (...). Remember that Kepler rejected the epicyclic Ptolemaic/Copernican models because he could not obtain an adequate fit within that class.” In turn, we note that emerging Bayesian GOF methods involve nonparametric alternatives [21]-[23], thus enriching the Bayesian GOF toolbox.

Given that frequentist statistics are believed to be more powerful than Bayesian statistics for model criticism [6], [12], Little [10] viewed calibrated Bayesian p-values as an improvement over purely Bayesian p-values – and in this article we will indeed focus on calibrated Bayesian techniques. The Material and Methods section begins by proposing a brief overview of what is known on frequentist and calibrated Bayesian GOF p-values under assumption (A1) according to three criteria:

C1: asymptotically with respect to sample size, the probability distribution of the p-value when replicating over observed datasets should be known for a variety of discrepancy functions and priors;
C2: under reasonable finite sample sizes, the probability distribution of the p-value when replicating over observed datasets should be close to a known reference distribution for a variety of discrepancy functions and priors;
C3: the p-values should be numerically inexpensive and relatively easy to implement based on a Monte Carlo Markov Chain or frequentist model fit [3], [16].

Conditions (C1) and (C2) are required in order to use candidate GOF p-values as described above in the Fisherian perspective. Having p-values that work for very different probability distributions and any discrepancy function has an obvious advantage: it provides users with assurance that they can use the method for different kinds of statistical models, and that they have sufficient flexibility to check the model [4], [15], [20], [24]. Condition (C3) is motivated by time constraints in the application of such methods.

As will be seen in the Material and Methods section – to which point we defer a precise definition of the p-values – some calibrated Bayesian and classical frequentist GOF p-values share the difficulty that their probability distribution is generally unknown, even asymptotically; this contradicts (C1), which makes it difficult to interpret the surprise resulting from a given p-value [14]-[17]. For this reason, posterior predictive p-values (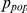) [4], [13], which are possibly the most widely used in modern applied Bayesian settings, have come under challenge from the statistical literature [14], [15], [17]. Other calibrated Bayesian GOF p-values prove very computer-intensive – thus contradicting (C3). Finally, most of them do not apply to general discrepancy functions – thus contradicting (C1) and (C2). Three of the reviewed p-values – the prior predictive p-value (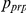; [6]), the plug-in half-sample ML p-value (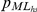; [25]) and the normalized sampled posterior p-value (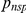), developed in [16], [18] – meet these three criteria, provided we have the same prior and likelihood in the data analysis as we had when generating data – for 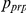 and 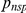, and provided that the discrepancy function depends solely on normalized data – for 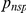, on uniformized data for 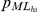
– or on data – for 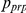. Normalized data are simple transformations of the observed data that:

- calculate uniformized data in 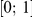, which are the values of the empirical cumulative distribution at observed values – based on the probability distribution used in the statistical likelihood and on a suitable parameter value;
- calculate the inverse cumulative function of the standard normal distribution on these uniformized data (cf. legend of Table 1 for a mathematical formulation).

**Table 1:** Discrepancy functions 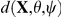 considered in the simulations of this paper.

*Description*	*General shape of the discrepancy function*
Test statistic function	*t(* **X**)
Test statistic function on normalized data	*t(* **Y**)
Other kinds of discrepancy functions	Centered mean (denoted meanc), variance (denoted varc), log-likelihood (LL)

NOTE: 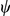 denotes a vector of length *n*, composed of random numbers from the uniform distribution that are independent from each other and from all the other random variables considered. 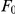 denotes the cumulative distribution function of the standard normal distribution, and 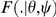 denotes the cumulative distribution function 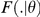 of the density 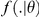 of the model – or a randomized version of it when X is discrete:

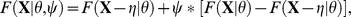
where 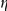 is a small positive number so that 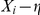 remains bigger than the closest smaller discrete value to 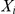. Normalized data are defined as 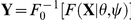.

We considered the following *t* functions: mean, variance, and only in the case of unnormalized data, 
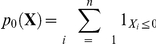
 and maximum (only for comparing 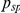 with 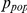 under the Poisson model), and only in the case of normalized data, skewness, kurtosis, and
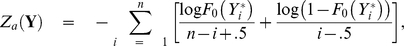
where 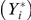 denotes the ascending ordered version of 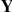. 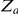 is obtained as 
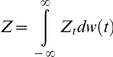
, with the likelihood ratio statistic as 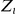 and an adequate weight function 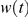 [37]. Centered mean and variance are the empirical mean and variance minus the mean and variance expected with 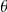.

The mathematical results we know for 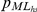 are limited to uniformized data. Also, we know that, in general, 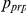 strongly depends on the prior chosen in data analysis, which is not the case for 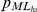. But is this also the case for 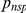
? Indeed, what happens to 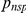 when the prior used in data analysis does not correspond to the prior used in data generation? Also, what happens when discrepancy functions are more general, i.e. dependent on statistical parameters or on unnormalized data – which leads to the more general sampled posterior p-value (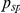)? And does 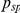 or 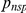 apply to discrete data? Finally, are 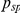 or 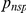 more powerful than 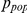 for detecting discrepancies between the data and the statistical model in situations when the likelihood in the statistical model is not the same as the likelihood in the probabilistic model? And how do 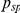 and 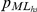 compare in such situations? In the second part of the paper, we study the promising p-values 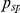 or 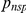 both mathematically and through simulations. Our main results are that:

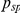
 meets criterion (C1);
- provided the prior distribution in the statistical analysis is equally or less informative than the prior in the probabilistic model, simulations on simple models indicate that 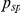 has an approximately uniform distribution and fulfils criterion (C2) with sample size from several dozens to several hundreds; and
- based on a specific example, 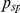 and 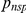 are shown to be more powerful p-values than 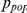 and as powerful as 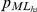.

This yields an easier way of calculating GOF p-values than the methods proposed in [7], [14], [17], [26]. In the last part of the paper, we discuss the benefits and drawbacks of this new p-value. Leading out of this discussion, 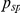, 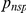 and 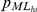 appear to be preferable to 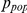 and other p-values.

## Materials and Methods

### Review of published results

For the sake of simplicity, this section will concentrate only on the mathematical setting for continuous observations. The case of discrete valued observations will be dealt with in the next section.

Suppose that we have observed a realization 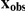 of a random variable **X**, 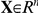. We propose a parametric probability family model, 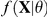, 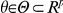, for the density of **X** given 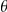, and a prior probability distribution 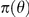 for 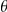. Although some of the results in this paper might also extend to cases where the prior is improper and the posterior is proper, we will assume (A2) throughout, i.e.:

(A2) the prior distribution is proper.

This paper will walk us through an investigation of the fit of the above statistical model with the observed data 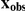. We do so by comparing the distribution of a given discrepancy function 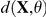 – where **X** and 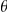 are simulated in some way from the statistical model – with the value involving observed data, 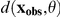, using the Fisherian p-value:
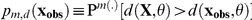
as a measure of compatibility, where 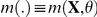 is a reference probability density for 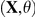 that depends on the statistical model. Each GOF p-value is defined by a reference density *m* and a discrepancy function *d* [15], [20]. When the discrepancy function *d* does not depend on 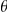, Robins et al. [15] propose to shift terms and call *d* a test statistic function.

Our setting has so far been purely Bayesian. The frequentist part of the setting is defined by a probabilistic model for the random sampling of data **x**∼*m*_0_ according to a given density
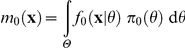
based on the parametric probability family model, 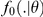, and on a prior probability distribution 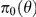 – which can be a Dirac or point mass distribution. Following many authors [14]-[17], [27], we require that, under (A1) (i.e. 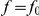), the probability distribution of 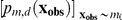 be known at least asymptotically – i.e. when the size *n* of 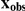 tends to infinity – and, more precisely, that this distribution be the uniform distribution on 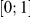, i.e.
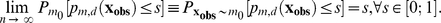
Such GOF p-values will hereafter be called *asymptotically uniform*.

The classical p-values proposed in the literature meet criterion (C3). They correspond to the following reference densities:

the *plug-in ML density*: 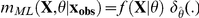, where 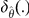 is the Dirac function at 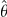, which is the Maximum Likelihood Estimator (MLE) of 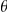 – given 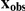 and the likelihood *f*. Even though other values than the MLE can be used for 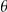 in a plug-in p-value (cf. [16]), this is a reference density that is used at least implicitly in many frequentist diagnostic tools (cf. graphical tools in [1], [2]);
the *prior predictive density*: 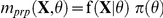 [6];
the *posterior predictive density*: 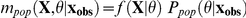, where 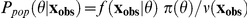 is the posterior density of 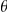, given 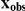, and 
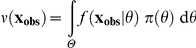
 is the marginal density of 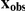 [12], [13].

This paper will not go further in investigating the prior predictive p-value – dubbed 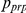 – because of its strong dependence on the statistical prior 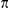, in contradiction with (C2) [10], [14] (also see Text S6).

With 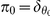 for some fixed 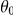, and under the general assumption that the function *d* is a function of **X** alone that has a normal limiting distribution, Robins et al. [15] showed that the plug-in ML and posterior predictive p-values – respectively dubbed 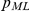 and 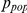 – are asymptotically uniform when the asymptotic mean of 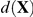 does not depend on 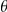. If the asymptotic mean of 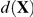 depends on 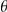, then as shown by Robins et al. [15], 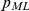 and 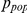 are generally not asymptotically uniform: more precisely, they are conservative p-values, which means the probability of extreme values is lower than the nominal probabilities from the uniform distribution. These p-values therefore only fulfill criterion (C1) if we greatly restrict the discrepancy functions considered.

This has led to the development of other p-values associated with less classical densities *m*, among which:

the *post-processing* method of the posterior predictive p-value to render it a uniform p-value [17];
the *partial posterior predictive density*: 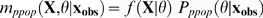, where 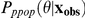 is the partial posterior density of 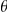, proportional to 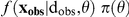 where 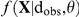 is the density function of **X** conditional on the value of 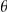 and on 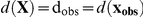 [14];
the *conditional predictive density*: 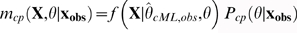, where 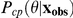 is the density that is proportional to 
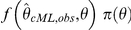
, where 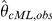 is the maximizer of the likelihood 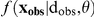 and where 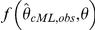 is the marginal density of the random variable 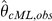 evaluated at its observed value [15];
the *plug-in half-sample ML density*: 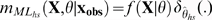, where 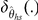 is the Dirac function at 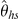, which is the MLE of 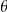 given a *half random sample of* 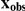 and likelihood *f* [25];
what we hereby term the *sampled posterior p-value* (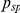) developed in [16], [18], based on 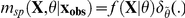, where 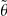 is a unique value of 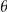, which is a random sample of the posterior distribution 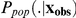.

With 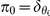 for some fixed 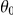, it has been proved mathematically, under certain assumptions, that the partial posterior predictive and conditional predictive p-values are asymptotically uniform p-values whatever the test statistic function and the prior distribution [15], thus fulfilling criterion (C1), with restrictions on discrepancy functions. However, due to criterion (C3), we will consider neither the partial posterior predictive density, nor the conditional predictive p-value [15], [16] nor the post-processing method in [17] in this paper.

Durbin [28] showed that the plug-in half-sample ML p-value 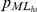 was asymptotically uniform provided it was used on uniformized data and with specific test statistic functions. This p-value has seldom been adopted, although Stephens [25] stressed its usefulness.

Johnson [16] proved that for a specific discrepancy measure, the sampled posterior p-value is also asymptotically uniform. More recently, Johnson [18] showed that if:

the statistical model – including the prior 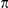 – is the same as the probabilistic model – including the prior 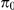 – from which the data were sampled; and

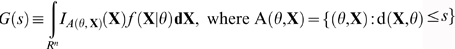
 depends solely on *s*, whatever the value of 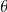 – i.e. in short, if 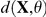 is pivotal;

then 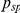 is not only asymptotically uniform, but is also uniform whatever the sample size. Normalized sampled posterior p-values (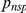) that use test statistics on normalized transformations of **X** possess this property. These p-values thus fulfill criteria (C1) and (C2) but with restrictions on discrepancy functions and on the prior distribution, as 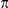 must be equal to 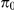.

### Simulation setting

What do we know about 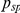, with more general discrepancy functions? We will show in the Results section that, for any discrepancy function, 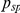 is uniform for 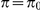 and asymptotically uniform for 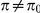, including for discrete-valued discrepancy functions. We also wanted to include discrete-valued discrepancy functions, due to the discrete nature of either the random variables **X** or the discrepancy function. We will therefore consider the following modified p-value:
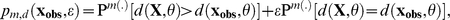
where 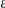 is drawn from a uniform distribution, independently of the other random variables.

Based on the mathematical results to come, 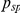 appears a promising p-value that applies widely in terms of discrepancy functions, and – asymptotically – in terms of prior distributions. However, these results no longer hold when the land of asymptotia is obviously not reached, as can be the case in hierarchical models or in models that fit parameters with a limited number of observations (see, for instance, the last model in the Poisson example in [16]). Furthermore, when sample size is moderate and the statistical prior does not correspond to the data generation prior, we have no clear information on how close 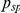 is to being uniform. We therefore used simulations to study how 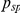 behaves in a finite sample context under four scenarios.

#### Objectives and scenarios

Our first scenario was performed to illustrate the uniformity results in the Results section when 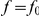 and 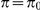, while the three other scenarios were conceived to study in a finite sample context the distance to uniformity of the empirical distribution of 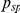, 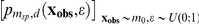, for different kinds of discrepancies between the probabilistic and statistical prior distributions:

*Scenario 1*: *Perfect fit between the probabilistic and statistical models*. Here, the model that generated the data and the model used to fit the data were exactly the same – including for the prior distribution.

*Scenario 2*: *The statistical and probabilistic models differ only by the dispersion of their priors.*

*Scenario 3*: *The statistical and probabilistic models differ only by the centering and dispersion of their priors.*

*Scenario 4*: *The statistical and probabilistic models differ only by their priors, the probabilistic prior* 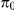 *being a Dirac distribution*. This setting is the same as in Scenario 1, except that data were generated from fixed parameters chosen at the mean of their statistical prior under Scenario 1.

Finally, we compared 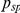 with 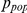 and 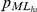 under Scenario 4 and a modification of Scenario 4 in which 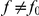 to illustrate the conservativeness of 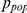 and the potentially good properties of 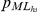
under Scenario 4, and to study the difference of power between the three p-values.

#### Models and methods

We dealt with these issues on the following parametric models, both for data generation and data analysis, which involved conjugate priors [4] (also see Table 2):

Poisson model: 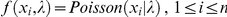 with a Gamma prior for λ: 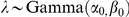;
Normal model: 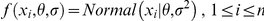 with the priors: 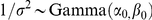 and 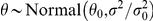;
Bernoulli model: 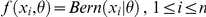 with a Beta prior for 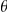: 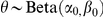.

**Table 2:** Summary of the models considered in simulations.

Poisson model	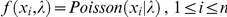 and 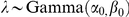
Normal model	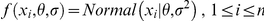 , 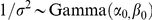 and 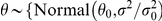
Bernoulli model	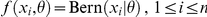 and 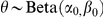

NOTE: For each dataset, 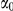, 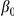, 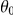 and 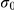 were held fixed in the data generation and data analysis steps.

For each dataset, 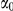, 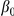, 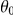 and 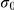 were held fixed in data generation and data analysis but were allowed to differ between the two phases. As conjugate priors were used, the explicit formula for the posterior distribution was known [4] and thus used under R 2.2.1 software [29] to fit the Bayesian models to the data.

Under Scenario 1, the priors were as above, with some parameters held fixed and some parameters that were capable of varying between datasets:

for the Poisson model, constant mean and random index of dispersion of the Gamma prior: 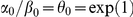 and 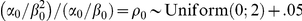;
for the Normal model, constant mean and random variance of the prior for 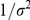: 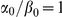 and 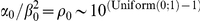, and constant 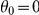, 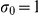 in the prior for 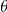; and

for the Bernoulli model, 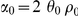 and 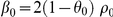, with 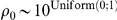 and 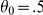.

The setting of Scenario 2 is the same as in Scenario 1, except that 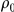 is replaced in the statistical model by 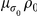 in the Poisson and normal cases and by 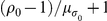 in the Bernoulli case, where 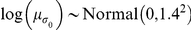. Scenario 3 differs from Scenario 2 by 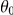 values in the statistical model that are no longer fixed but drawn at random according to 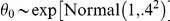 in the Poisson case, 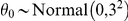 in the Normal case and 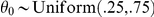 in the Bernoulli case. The distributions for parameters 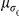 and 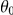 in Scenarios 2 and 3 were chosen to vary the levels of informativeness and off-centering of the statistical prior with respect to the probabilistic prior. Finally, in Scenario 4, data were generated from fixed parameters, chosen at the mean of their statistical prior under Scenario 1, i.e. 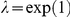 in the Poisson case, 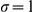 and 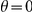 in the Gaussian case, and 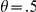 in the Bernoulli case.

We considered three kinds of discrepancy function, 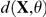, i.e. test statistics, test statistics on normalized data and other discrepancy functions (cf. Table 1). Test statistics on normalized data were introduced because they define pivotal quantities used by [18] to find results under the condition 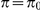.

The number of observations in each dataset, *n*, was a random figure between 20 and 1,000: 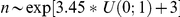 with probability 0.7 and 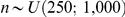 with probability 0.3. *n* was rounded to the nearest ten or – if the value was above 200 – to the nearest hundred. We used 5,000 sampled values of 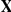 to calculate p-values. The programs were run either on a DELL Latitude D830 Intel Centrino T7250 or on a server with two dual-core Opteron 2.2 GHz processors and 3 Gb of RAM. One hundred thousand replicated datasets were studied under Scenarios 2 to 4 and 10,000 under Scenario 1. To illustrate the dependence of 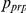 on the statistical prior distribution, we also calculated the 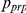 based on 3,000 datasets under Scenario 2.

The p-value associated to each dataset and each chosen discrepancy function differed from the classical calculation for predictive p-values. Let us denote:
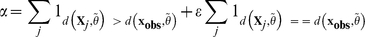
and
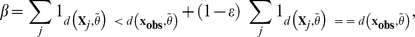
where 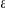 is a random value from the uniform distribution. Instead of the classical formula 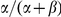 [4], the p-value was drawn at random from the beta distribution with the respective shape parameters 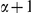 and 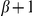. Indeed, it can be shown that this distribution is the posterior distribution of the underlying p-value 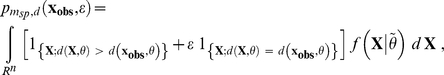
once we have observed or sampled 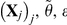 and 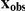, provided the prior of the p-value is uninformative [4] (p.40). In contrast, the use of 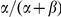 can result in significant departures from the uniform distribution, which would be due to the calculation method and not to the underlying p-value; this would especially occur with a low number of replicated data 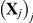 or to estimate the tails of the uniform distribution (see Text S9).

The resulting p-values were considered as sampled from the distribution 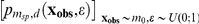. They were numerically compared with the uniform distribution, through Kolmogorov-Smirnov tests, which are adequate and easy to calculate for such continuous valued distributions, as well as through binomial two-sided tests for the proportion of p-values that were in the 5% or 1% extremities of the 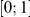 interval. As stated above, we used a uniform random number 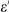 to ventilate between the “less extreme” and “more extreme” categories, the probability of the event when the proportion simulated from the binomial distribution was equal to the observed proportion. This guaranteed a uniform distribution of the associated p-value. For the proportion of p-values in the 5% or 1% extremities of the 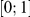 interval, we also calculated the posterior density of the estimated proportion from the observed number, using a beta distribution as above. We then analyzed where the posterior estimates were positioned relative to intervals around the target probabilities of 5% or 1%. For example, we distinguished cases where 95% of the estimates of the underlying proportion of p-values fell in the interval 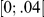 (proportion of p-values is estimated to be non-negligibly less than 5%), from cases where 95% of the estimates fell in the interval 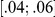 (proportion of p-values is estimated to be negligibly different from 5%), and from cases where 95% of the estimates fell in the interval 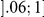 (proportion of p-values is estimated to be non-negligibly greater than 5%) (see Text S1).

#### Comparing 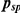 with 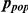 and 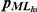 **under the Poisson model**

**Figure 1:**
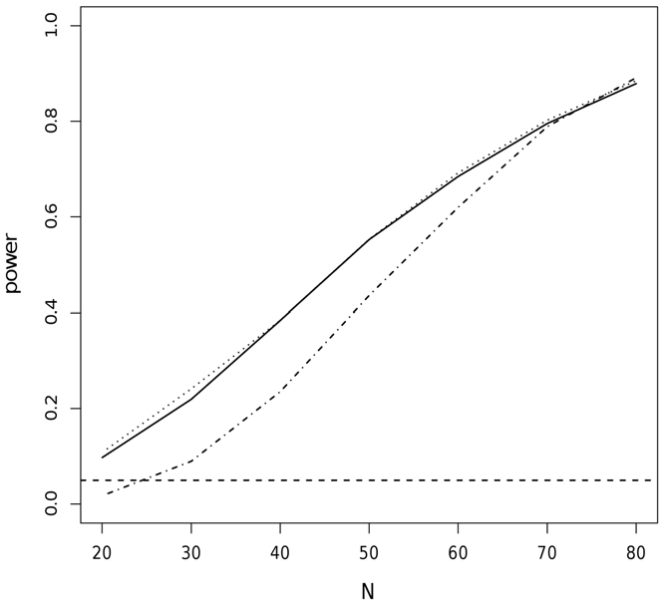
Power of the sampled posterior (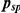; solid line), half-sample ML (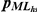; dotted line) and posterior predictive (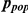; dotted-dashed line) p-values. Power of the p-values 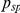 (solid line), 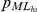 (dotted line) and 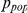 (dotted-dashed line) used with the maximum test statistic to detect departures from the Poisson distribution at the level of p = 0.05 when data are distributed according to a Polya distribution with 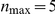. Power is plotted as a function of sample size N varying between 20 and 80. 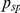 and 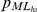 were equivalent in terms of power, and both were more powerful than 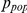 except at the highest sample sizes. The dotted baseline level corresponds to p = 0.05.

Finally, for the Poisson model, we compared 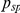 with 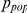 and 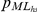 under Scenario 4 and a modification of Scenario 4 in which 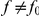. We used the same test statistics as above, plus the maximum function. Forty-thousand datasets were generated as in Scenario 4 or from a Polya distribution [30] with a maximum value 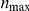 drawn at random from the values 4 and 5, and a mean and variance equal to those of the aforementioned Poisson distribution. The sample size was drawn at random from between 20 and 50, except for Figure 1 where it was sampled from the set (20,30,40,50,60,70,80).

The R commands to run and analyze the simulations described above can be found in Text S8.

## Results

### The sampled posterior p-value: mathematical results

#### 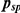 is uniform when 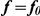 and 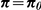


The following lemma extends Johnson's [18] results on test statistics applied on normalized data to general discrepancy functions, including discrete-valued discrepancy functions:

**Lemma**: Assume that 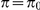 is proper, and 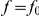 – so that assumptions (A1) and (A2) are met. Then, for every discrepancy function *d*, the probability distribution of 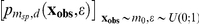 is uniform, i.e.
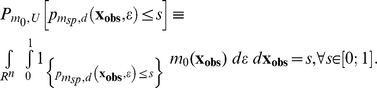



*Proof*. The proof of this Lemma follows the same line as the proof of the Lemma in [18]. For the sake of clarity, let us denote 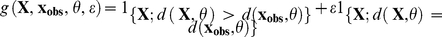, so that 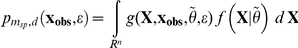. Then, by simply substituting the place where the marginal density 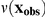
occurs in the integrals,
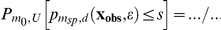


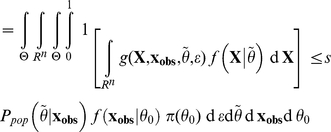


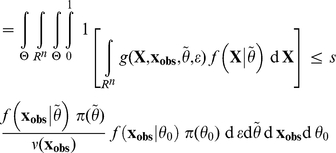


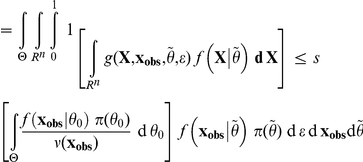


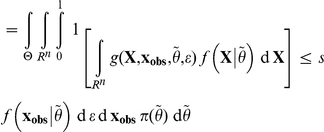
However, in this last equation, conditional on 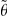, 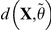 and 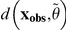 in function *g* have the same probability distribution and are independent. Then, still conditional on 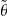, due to the very definition of *g*, 
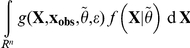
 has a uniform distribution between 0 and 1 when 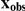 then 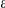 are sampled as specified in the integral. For this reason, the above formula can be rewritten as:
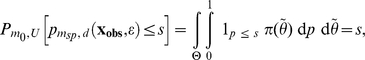
which yields our result.

#### 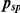 is asymptotically uniform when 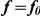 and 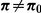


The above result shows that 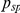 is uniform provided (A1), (A2) and the statistical prior 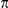 – which generates the posterior distribution 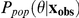 – is the same as the probabilistic prior 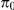. We can extend this result when both priors differ by showing that that under conditions:

on the likelihood – including the identifiability of the model, and the independence of observations;
on the priors – including that for every 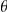 such that 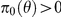, we must have 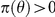;
on the discrepancy function – its continuity relative to 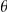;
on the parameter space 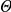 – its compactness;

then, 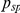 is asymptotically uniform under (A1) and (A2).

*Sketch of proof*. If we assume that the parameter space 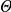 is compact, that the model is identifiable and that the random variables are independent and identically distributed, i.e. 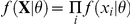, then when the size *n* of the sample 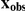 drawn from 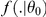 for a given 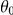 tends to infinity, whatever the neighborhood *A* of 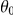, 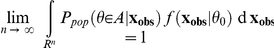, i.e. 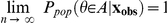, 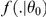-almost surely [4] (p.587 in Appendix B). From the continuity of 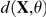 relative to 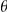 conditions, we deduce that 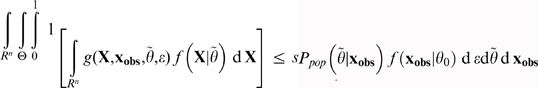 is asymptotically equal to 
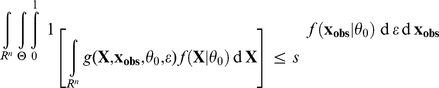
, which as in the proof of the above Lemma is equal to *s*. Since these quantities are bounded by 1, we get through an integration over 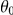 according to the prior 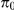, that 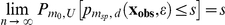.

We speculate that the proof in Gelman *et al.* [4] (p.587 in Appendix B) can be extended to the case where the random variables are independent but not identically distributed – i.e. 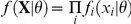
– provided the 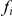 distributions are sampled from a common probability law, making it possible to use Kolmogorov's strong law of large numbers instead of the usual law of large numbers employed in [4].

*Discussion*. In these conditions, 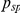 is asymptotically uniform even when 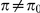. These results also hold when 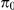 is a Dirac distribution 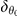. Under more stringent conditions on the likelihood and the prior, these results can be made sharper – and inform on the speed of convergence – by using the convergence of the posterior distribution to normality [4], [31]-[33].

#### The sampled posterior p-value: simulation results

Our above results are mathematical and mostly asymptotic. We now study the finite sample behavior of the sampled posterior p-value based on our simulations. Overall, our results for Scenario 1 – corresponding to a perfect matching of the statistical and probabilistic models – were in accordance with our expectations: 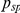 and 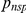 then had behaviors compatible with uniform p-values (Text S1).

When the statistical prior had the same mode but was sharper than the probabilistic prior in Scenario 2, 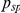 and 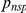 yielded poor results for the studied sample sizes (Table 3 and Text S2), in contrast with their asymptotic good behavior (previous section). Conversely, when the statistical prior was less informative than the probabilistic prior, both p-values were much closer to being uniform (Text S2), in sharp contrast with 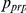 (Text S6).

**Table 3:** Behavior of 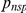 relative to the uniform distribution under Scenario 2, depending on the interval housing the statistical prior sharpness parameter 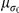.

Interval to which *μ_σ0_* belongs	[.0;.40[	[.40;1.01[	[1.01;2.59[	[2.59;∞[
*D*	.080^***^	.005	.01^*^	.009^*^
*P_5%_*	.091^***,++^	.049^00^	.050^00^	.052^00^
*P_1%_*	.035^***,++^	.011^0^	.009^0^	.011^0^

Kolomogorov-Smirnov distance (*D*) between the simulated 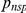 and the uniform distribution and frequency (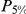 and 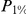) of 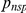 found at the 5% and 1% extremities of the unit interval for the Poisson model with 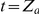 in Scenario 2 based on 100,000 different datasets. In this Scenario, the statistical prior 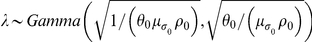, has a different sharpness to the probabilistic prior 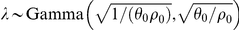. The statistics for the overall sample were 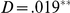, 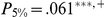 and 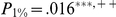. These results illustrate that for 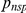 to be approximately uniform when the statistical prior is not the same as the probabilistic prior, it is preferable for the statistical prior to be less informative rather than more informative compared with the probabilistic prior. Similar results were found for other test statistics and other probability distributions (cf. Text S2).

NOTE: The notation for the significance of the tests is as follows: (*) means that the test is significant at a level between .05 and .1; * between .01 and .05; ** between .0001 and .01; *** less than .0001. The notation system for the study of the negligibility of departures from expected values is as follows, for 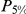: 00 (respectively, 0) means 95% of the estimated values of the underlying p-value are in the interval 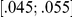 (resp. 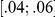); ++ (respectively, +) means 95% of the estimated values are in the interval 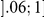 (resp. 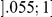); – (respectively, -) means 95% of the estimated values are in the interval 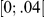 (resp. 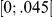). For 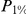, the notations are the same but with cutoff points divided by 5.

Except in one case, 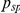 and 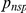 were also not far from being asymptotically uniform in Scenario 4 when the true parameter value was equal to the mode of the statistical prior (cf. Text S4). An exception was observed for the Bernoulli model with 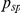 and 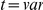 or 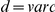: in this case, 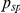 did not approach uniformity, even with relatively high sample sizes. On the whole, however, 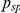 and 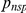 were further from being uniform for small sample sizes in Scenario 4 than in Scenario 2 with uninformative statistical priors.

De-centering of the statistical prior (Scenario 3) yielded 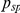 and 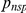 values that were further from the uniform distribution (Table 4 and Text S3). However, 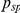 and 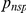 remained relatively close to being uniform when the statistical prior was less informative than the probabilistic prior and when de-centering was not too strong.

**Table 4:** Behavior of 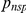 relative to the uniform distribution under Scenario 3, based on the frequency of 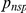
values found at the 5% extremities of the unit interval, depending on the interval of the statistical prior sharpness parameter 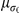 (in rows) and off-centering parameter 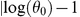 (in columns).

Interval to which μ_σ0_ (row) and |log(θ_0_) -1| (column) belong	[.0;.14[	[.14;.28[	[.28;.47[	[.47; 1.73[
[.0;.40[	.093^***,++^	.106^***,++^	.157^***,++^	.241^***,++^
[.40;1.01[	.053^0^	.053^0^	.054^0^	.081^***,++^
[1.01;2.59[	.050^0^	.054^0^	.046^0^	.056^*^
[2.59;∞[	.050^0^	.053^0^	.051^0^	.050^0^

Frequency (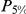) of 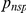 that are at the 5% extremities of the unit interval, for the Poisson model with 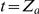 under Scenario 3, according to the values of 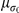 and 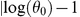, for 100,000 different simulated datasets. Similar results were found for other t functions and for the Poisson distribution, with more significant results for certain other t functions when 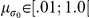 and 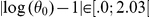 (see Text S3).

NOTE: The notation system for the significance of the tests and the negligibility of departures from expected values are as in Table 3. Qualitatively similar results were found for 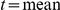 and 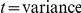. For 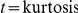 and 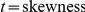, results were much less strongly and much less frequently significant.

Comparing 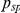 against 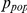 and 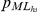 for the Poisson model under Scenario 4 with 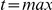 showed that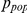 was conservative, as expected by the mathematical results in [15] while 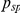 and 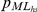 were closer to being uniform for sample sizes 20 and 50 (Text S5). When the true distribution was a Polya distribution instead of a Poisson distribution, 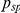 and 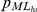 were of similar and greater power, except for the highest sample sizes where 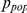 tended to be slightly more powerful (Figure 1). A difference in power of 10 to 20% in favor of 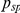, 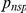 or 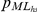 was not uncommon and was observed with various discrepancy functions (Figure 1 and Table 5).

**Table 5:** Difference in power between 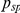 or 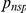
and 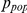 according to sample size (in columns) and discrepancy function (in rows).

Sample size *N*	20	50
Skewness on normalized data	.050	.103
Kurtosis on normalized data	.119	.243
Za on normalized data	.031	.135
Maximum on normalized data	.068	.204
Maximum on raw data	.083	.113

Difference in power between 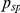 or 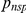
and 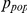 for detecting departures from the Poisson distribution at the 5% level and when the true distribution is a Polya distribution with maximum value 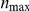 equal to 5, with the sample size *N* equal to 20 or 50. Various discrepancy functions and test statistic functions are considered. The difference in power is positive, indicating more power for 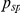 or 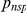. The magnitude of the difference can be quite substantial, ranging from 0.1 to 0.25. Similar results were obtained between 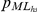 and 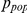, with slightly greater power differences than between 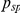 or 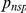
and 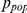.

## Discussion

### Synthesis of results

In this paper, we first recap on various calibrated Bayesian methods for goodness-of-fit (GOF) p-values and extend the results found in [18] for normalized sampled posterior p-values (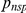) in different directions. We show in particular that similar results apply for the more general 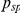 when the data are not normalized and for discrepancy functions that can be discrete-valued rather than only for continuous-valued test statistic functions. We also show that this p-value is asymptotically uniform when the statistical prior differs from the probabilistic prior (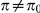). Through simulations, we empirically tested this p-value under 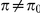 in a finite sample context. The results show that 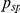 has a relatively correct behavior provided that the statistical prior is “not too informative and not too uninformative”, and not too far off-centered, relative to the probabilistic prior. An exception to this statement occurred in Scenario 4 with the Bernoulli model and 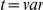 or 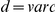, for which 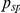 was far from being uniform even for relatively large sample sizes. We think this is because the fixed parameter 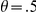 used to sample 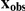 was precisely the parameter value for which the variance was the largest over the full parameter space. This might correspond to a very slow convergence in this specific case or to a restriction of our asymptotic mathematical results, somewhat similar to the convergence at the edge of parameter space in [4] (Section 4.3). A simulation with 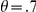 yielded a 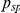 that was much closer to being uniform (Text S4).

Based on these new results and on the review of published results (Material and Methods Section), we shortlisted three alternative methods as simple candidates of asymptotically uniform GOF p-values:

Method 1: 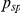, with a variety of discrepancy functions *d* and with not too inadequate statistical priors;

Method 2: 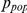 or 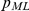 with a test statistic function *t* such that asymptotically the mean of 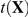 is not dependent on 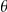 [15]. Examples of such functions include skewness or kurtosis for the normal distribution, skewness for the t distribution, or the ratio between the mean of the sample and its variance for a Poisson distribution;

Method 3: 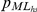, with specific test statistic functions used on uniformized data.

We will also discuss two other, more elaborate sets of methods:

Method 4: partial posterior predictive p-values (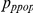) or conditional predictive p-values (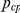) used only with test statistic functions, as developed and proposed by [14], [15], [26];

Method 5: calibrated posterior predictive p-values (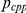) as proposed in [17].

One last method could have been to use 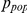 or 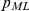 with test statistic functions, knowing that they are conservative [15]. However, our results for 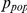 show that we then lose a significant amount of power compared with 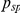 and 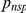 (Figure 1 and Table 5). This strategy will therefore not be considered further here.

### The relative merits of candidate p-values

We hereafter discuss the merits and limits of our preferred method – Method 1 or 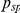 – in comparison with the other candidate methods. With respect to Method 2, Method 1 has the advantage of allowing the use of various discrepancy functions whereas Method 2 requires very specific test statistic functions; this means that different aspects of the probabilistic model can be studied with Method 1 rather than only the *t* functions that characterize the hypothesized probabilistic distribution. We agree with [4], [20], [24] on the necessary adaptation of discrepancy functions to each particular situation where we might want to test departures of data from the model on case-specific features. This makes it possible to include problems involving detection of outliers (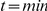 or 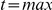) and dependence between observations [24] in model checking. It also means that 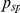 appears more flexible and better applicable to very different probability distributions than Method 2: for more complicated hypothesized distributions, it might be difficult to build *t* functions such that asymptotically the mean of 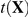 does not depend on 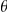.

On a more theoretical grounding, while 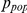 and 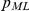 provided default and intuitive responses to question (b) in [34], i.e. “what replications should we compare the data to?” – 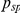 gives a different and less intuitive answer, based on mathematical results: replications should all be sampled from the likelihood based on a unique parameters value, itself sampled from the posterior distribution, and not from multiple parameters values sampled from the same distribution (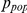) or from the Maximum Likelihood parameters (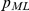).

In comparison with 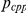 (Method 5), the main advantage of 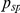 is its much weaker computational cost inside MCMC computations, including for complicated models. By contrast, 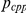 entails multiplying the MCMC computational burden by the number of “repetitions” of the model on which post-processing is based. This would take from at least a hundred to a thousand times longer than 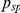. From our point of view, this is a major problem, especially in cases such as hierarchical models on large datasets. Therefore, the choice between Methods 1 and 5 may primarily depend on the length of time required to fit the model.

Regarding Method 4, the apparent weakness of 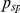 compared with the results in [26] for 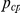 is that we have no information on when the asymptotic behavior is reached – except when the whole statistical model is the same as the probabilistic model used to sample the data. Nevertheless, our simulation results do show that provided the priors are not too informative or too uninformative, and not too far off-centered, 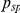 is not very far from being uniform. An advantage of 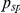 over 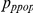 or 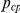 is its simplicity: we do not need to calculate the calibrated likelihood of the model with respect to the test statistic, as we do for 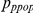 or 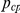. Moreover, if one wishes to calculate *N* different p-values based on different test statistics, it can be done inside the same numerical fitting in the case of 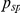 but must be done *N* times on *N* different calibrated likelihoods for 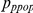 or 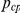. A final advantage of 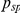 over 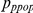 or 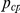 is that we have mathematical results for discrepancy functions in general, rather than just for test statistic functions as is the case for 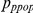 and 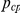.

Methods 1 and 3 appear very close in terms of applicability and, in the example studied, in terms of power. Their respective powers could be studied in more detail in the future. A common feature of both methods is that they give random results, in the sense that we can randomly reach different p-values for the same observed data 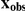. A small advantage in favor of 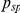 in Method 1 is that it does not require a separate fit on the half-sample, which contrasts with Method 3. A stronger advantage for Method 1 is that its asymptotic validity is proved for general discrepancy functions, whereas the mathematical results we have for 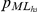 in Method 3 only apply to specific test statistic functions of uniformized data [28]. This in particular implies that we have no mathematical result on the asymptotic uniformity of 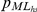 in Figure 1.

We therefore propose using 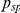 and 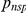 as a good GOF strategy, which is unrestricted with respect to distributions and *d* functions and which has a reasonable numerical and coding cost. To our knowledge, these are the only p-values that have a known asymptotic probability distribution whatever the discrepancy function.

### Notes on how to use the 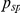


This section discusses two points related to the strategy of using 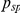: the choice of prior distribution, and the choice of the parameter value(s) used to sample “new data” and normalize it.

First, our results indicate that we should generally prefer priors that are moderately less informative in data analysis than in data sampling (Table 4 and Appendices 2 and 3). This statement somewhat echoes similar considerations in [17] (Section 9.3). If this result were to be generalizable, it would mean that when 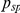 indicates a significant departure from the uniform distribution, depending on whether the prior is judged as too informative (or respectively, too uninformative), the same model should be tested with less informative (or respectively more informative) priors. An alternative might be to use 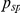 in a frequentist setting, provided the asymptotic assumption of normality of the estimators is assumed correct (cf. next section). If significant departures from a uniform distribution are still found, the probability distribution used in the likelihood should be reconsidered in data analysis.

Second, 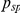 involves a single sampled value 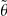 value of the model parameter 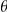, which means that the 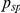 method might give different random results on the same dataset with the same model [18]. An alternative solution would be to use the probabilistic bounds method proposed in [18] (Section 2.3). A further potential alternative we propose, with the formalism of 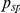 (see Table 1), could be –:

1. for each dataset 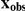 and function *d*, draw at random 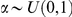;
2. after MCMC, calculate the sampled posterior p-values 
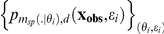
 associated with the 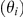 s sampled from the posterior distribution associated with 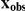 and 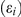 sampled from the uniform distribution;
3. consider the empirical 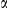-quantile of the latter distribution.

Provided analysts use the same value for 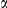 drawn at random at the beginning of the first analysis for the same dataset, this would guarantee a better comparability of the analysis of the same dataset by different analysts.

### Final global remarks

In contrast to the likelihood principle, calibrated Bayesian techniques involve the use of artificial data – i.e. data that were not observed. This makes pure Bayesians reluctant to use these techniques [35]. Indeed, internal calibrated Bayesian goodness-of-fit is sometimes considered to be a hopeless cause, where proponents want to have the cake – i.e. estimate model parameters based on all the data available – and eat it too – by confronting the fitted model to the same data that were used to fit it. Calibrated internal goodness-of-fit consequently attracts criticism for using the data twice [8]. Strikingly, 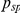 seems to provide a nearly uniform p-value, although it uses the data 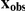 twice: once to estimate the posterior distribution – from which 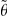 is sampled – and once again to calculate 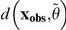. It therefore appears to warrant the same criticisms as 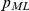 or 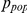, which were supposed to justify their lack of asymptotical uniformity. Johnson [16] explains it in these terms, in the context of chi-square statistics: “Heuristically, the idea [...] is that the degrees of freedom lost by substituting the grouped MLE for 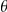 in Pearson's 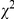 statistic are exactly recovered by replacing the MLE with a sampled value from the posterior [distribution]”. The proof of Lemma 1 in the Results section reveals another explanation: as we are working on *sampled* data to fit statistical models, we should also agree to work on *sampled* parameters to criticize the model. Indeed, this double sampling allowed us to make the roles of data and parameters symmetrical, enabling us to prove our mathematical results. Therefore, the problem lies less in that a GOF p-value uses data twice, but more in *how* it uses the data twice – see [36] on the need to more precisely define what we mean by “using the data twice”.

We have applied 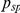 and 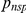 in a Bayesian context. However, as stressed in [16], these p-values might also be used with frequentist methods when the asymptotic assumption of normality of the estimators is correct. Indeed, we applied 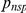 on the Poisson case by drawing a value of 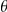 at random on the log scale from a normal distribution with the estimated mean as mean and with the estimated standard error as standard error fitted with a Poisson generalized linear model (glm). The results indicate as good a behavior as 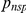 used in Bayesian models under Scenarios 1 and 4 (Text S7).

Little [10] once wrote that Bayesian statistics were relatively weak for model assessment compared to frequentist statistics. Although the underused 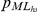 might be a good frequentist GOF p-value if its properties are known for more general discrepancy functions, our results highlight an even more attractive solution that mixes frequentist reasoning with a completely Bayesian modeling formulation, by using the sampled posterior p-values (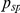) in a calibrated Bayesian framework. The transposition of 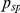 into a frequentist setting has been shown to be correct in the above example, and could therefore represent another potential “frequentist” solution. However, we believe that for the not-so-infrequent cases where the normal approximation of the estimate distribution is not accurate – as can be found for binomial or Poisson regression with a high proportion of zero values – a Bayesian framework is more adequate than a frequentist setting for sampling a value of 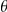.

## Supporting Information

Text S1 Results of Scenario 1.
(0.23 MB DOC)

Text S2 Results of Scenario 2.
(0.37 MB DOC)

Text S3 Results of Scenario 3.
(0.40 MB DOC)

Text S4 Results of Scenario 4.
(0.40 MB DOC)

Text S5 Results of Scenario 4 for the sampled posterior and the posterior predictive p-values.
(0.06 MB DOC)

Text S6 A simple illustration of the strong dependence of prior predictive p-values on the prior distribution.
(0.06 MB DOC)

Text S7 Results of the normalized sampled posterior p-value with frequentist Poisson generalized linear models.
(0.07 MB DOC)

Text S8 R commands to run and analyze the simulations.
(0.93 MB DOC)

Text S9 R commands to illustrate the discrepancy between the rough deterministic and the stochastic methods to transform α and β to p-values.
(0.05 MB DOC)
