# Supplementary material for: A New Calibrated Bayesian Internal Goodness-of-Fit Method: Sampled Posterior p-Values as Simple and General p-Values That Allow Double Use of the Data
Source: PLoS One. 2011 Mar 18;6(3):e14770. doi: 10.1371/journal.pone.0014770 (PMC3060804; doi:10.1371/journal.pone.0014770)
Supplement: Text S1 — Results of Scenario 1. (0.23 MB DOC) [file pone.0014770.s001.doc]

New Calibrated Bayesian Internal Goodness-of-Fit Methods: Sampled Posterior P-values as Simple and General P-values that Allow Double Use of the Data

Frédéric Gosselin

Cemagref, UR EFNO, F-45290 Nogent-sur-Vernisson, France

E-mail: [frederic.gosselin@cemagref.fr](mailto:frederic.gosselin@cemagref.fr)

*Results of Scenario 1*

# Text S1. Scenario 1 $p_{sp}$ and $p_{nsp}$ results

In the following tables, we present the results of $p_{sp}$ (sampled posterior p-values), and its normalized version, $p_{nsp}$, in the case of Scenario 1:

Scenario 1. In our first scenario, the model which generated the data and the model used to fit the data were exactly the same – including for the prior distribution. The common priors were $\lambda\sim\text{Gamma}\left(\alpha_{\text{0,}}\beta_{0}\right)$with $\alpha_{0}/\beta_{0}=\theta_{0}=\text{exp}\left(1\right)$ and $\left(\alpha_{0}/\beta_{0}^{2}\right)/\left(\alpha_{0}/\beta_{0}\right)=\rho_{0}\sim\text{Uniform}\left(0;2\right)+.05$, $1/\sigma^{2}\sim\text{Gamma}\left(\alpha_{\text{0,}}\beta_{0}\right)$ and $\theta\sim\text{Normal}\left(\theta_{0},\sigma^{2}/\sigma_{0}^{2}\right)$ with $\alpha_{0}/\beta_{0}\equiv1$, $\alpha_{0}/\beta_{0}^{2}=\rho_{0}\sim10^{\left(\text{Uniform}\left(0;1\right)-1\right)}$, $\theta_{0}\equiv0$, and $\sigma_{0}\equiv1$, and $\theta\sim\text{Beta}\left(\alpha_{\text{0,}}\beta_{0}\right)$ with $\alpha_{0}=2\theta_{0}\rho_{0}$ and $\beta_{0}=2\left(1-\theta_{0}\right)\rho_{0}$, with $\rho_{0}\sim10^{\text{Uniform}\left(0;1\right)}$ and $\theta_{0}=.5$, for the Poisson, Normal and Bernoulli cases, respectively.

In the following tables, we display the Kolmogorow-Smirnov statistic of the comparison of the p-values with a uniform distribution (ks.D), the proportion of values in the 5% extreme positions on the interval [0;1] (p.05), and the same for 1% (p.01). 10,000 data set sampling and analyses were performed. *n* denotes the sample size and # the number of simulations corresponding to the sample size. For proportions, we used tail-area tests to detect significant departures from the point null hypotheses and also studied whether the posterior density of the underlying proportion was negligibly or non-negligibly different from the target proportions.

The notation for the significance of the tests is as follows: (*) means that the test is significant at a level between .05 and .1; * between .01 and .05; ** between .0001 and .01; *** less than .0001. The notation for the study of negligible/non-negligible departures from the expected values were as follows:

– for the proportion of the p-values in the 5% extreme positions on the [0;1] (p.05): 00 (respectively, 0) means 95% of the estimated values of the underlying p-value are in the interval $\left[.045;.055\right]$ (resp. $\left[.04;.06\right]$); ++ (respectively, +) means 95% of the estimated of the underlying p-value are in the interval $\text{].06};\text{1]}$ (resp. $\text{].055};\text{1]}$); -- (respectively, -) means 95% of the estimated of the underlying p-value are in the interval $\text{[0};\text{.04[}$ (resp. $\text{[0};\text{.045[}$);

– for the proportion of the p-values in the 1% extreme positions on the [0;1] (p.01): 00 (respectively, 0) means 95% of the estimated values of the underlying p-value are in the interval $\left[.009;.011\right]$ (resp. $\left[.008;.012\right]$); ++ (respectively, +) means 95% of the estimated of the underlying p-value are in the interval $\text{].012};\text{1]}$ (resp. $\text{].011};\text{1]}$); -- (respectively, -) means 95% of the estimated of the underlying p-value are in the interval $\text{[0};\text{.008[}$ (resp. $\text{[0};\text{.009[}$).

In the following results, it may seem that some departure from the uniform distribution appears for p.01 and large values of n, in the case of $p_{sp}$, $t=mean$ or $p_{sp}$, $d=meanc$. We checked this specific situation with independent simulations on a larger sample size – 20,000 data sets generated – and confirmed that there was no significant departure from the uniform distribution (results not shown).

## Case 1: Poisson

$p_{nsp}$, $t=mean$

*n* ks.D p.05 p.01

1 [ 20, 80) 0.011 0.050 0 0.010

2 [ 80, 300) 0.010 0.051 0 0.012

3 [300, 600) 0.021 0.048 0 0.011

4 [600,1000] 0.017 0.050 0 0.007

5 ALL 0.007 0.050 00 0.010

$p_{nsp}$, $t=var$

*n* ks.D p.05 p.01

1 [ 20, 80) 0.012 0.052 0 0.013

2 [ 80, 300) 0.014 0.047 0 0.010

3 [300, 600) 0.024 (*) 0.055 0.012

4 [600,1000] 0.019 0.042 (*) 0.008

5 ALL 0.010 0.050 00 0.011

$p_{nsp}$, $t=skew$

*n* ks.D p.05 p.01

1 [ 20, 80) 0.016 0.046 0.009

2 [ 80, 300) 0.015 0.050 0 0.011

3 [300, 600) 0.013 0.053 0.010

4 [600,1000] 0.019 0.053 0.009

5 ALL 0.010 0.050 00 0.010 0

$p_{nsp}$, $t=kurt$

*n* ks.D p.05 p.01

1 [ 20, 80) 0.012 0.053 0.010

2 [ 80, 300) 0.023 0.054 0.011

3 [300, 600) 0.015 0.042 * 0.008

4 [600,1000] 0.028 (*) 0.048 0 0.010

5 ALL 0.012 0.049 00 0.010 0

$p_{nsp}$, $t=Z_{a}$

*n* ks.D p.05 p.01

1 [ 20, 80) 0.020 0.050 0 0.010

2 [ 80, 300) 0.014 0.055 0.013

3 [300, 600) 0.018 0.044 0.008

4 [600,1000] 0.009 0.046 0.008

5 ALL 0.005 0.049 00 0.010

$p_{sp}$, $t=mean$

*n* ks.D p.05 p.01

1 [ 20, 80) 0.013 0.048 0 0.011

2 [ 80, 300) 0.016 0.054 0.013

3 [300, 600) 0.018 0.051 0 0.011

4 [600,1000] 0.013 0.056 0.007

5 ALL 0.005 0.052 0 0.011

$p_{sp}$, $t=var$

*n* ks.D p.05 p.01

1 [ 20, 80) 0.010 0.047 0 0.009

2 [ 80, 300) 0.016 0.047 0 0.007

3 [300, 600) 0.017 0.057 (*) 0.012

4 [600,1000] 0.015 0.044 0.009

5 ALL 0.008 0.049 00 0.010 0

$p_{sp}$, $t=p_{0}$

*n* ks.D p.05 p.01

1 [ 20, 80) 0.024 (*) 0.052 0 0.010

2 [ 80, 300) 0.008 0.052 0.011

3 [300, 600) 0.017 0.052 0.009

4 [600,1000] 0.024 0.054 0.011

5 ALL 0.006 0.053 0 0.010

$p_{sp}$, $d=meanc$

*n* ks.D p.05 p.01

1 [ 20, 80) 0.012 0.049 0 0.010

2 [ 80, 300) 0.016 0.053 0.013

3 [300, 600) 0.019 0.054 0.012

4 [600,1000] 0.014 0.054 0.005 *,-

5 ALL 0.005 0.052 0 0.010

$p_{sp}$, $d=varc$

*n* ks.D p.05 p.01

1 [ 20, 80) 0.011 0.047 0 0.010

2 [ 80, 300) 0.015 0.047 0 0.007

3 [300, 600) 0.016 0.057 (*) 0.012

4 [600,1000] 0.015 0.046 0.008

5 ALL 0.009 0.050 00 0.009

$p_{sp}$, $d=LL$

*n* ks.D p.05 p.01

1 [ 20, 80) 0.012 0.050 0 0.012

2 [ 80, 300) 0.021 0.048 0 0.008

3 [300, 600) 0.011 0.053 0.009

4 [600,1000] 0.022 0.049 0 0.007 (*)

5 ALL 0.009 0.050 00 0.009

## Case 2: Normal

$p_{nsp}$, $t=mean$

*n* ks.D p.05 p.01

1 [ 20, 80) 0.016 0.050 0 0.009

2 [ 80, 300) 0.019 0.052 0 0.010

3 [300, 600) 0.027 * 0.046 0 0.009

4 [600,1000] 0.018 0.052 0.005 *,-

5 ALL 0.013 (*) 0.050 00 0.008

$p_{nsp}$, $t=var$

*n* ks.D p.05 p.01

1 [ 20, 80) 0.015 0.049 0 0.011

2 [ 80, 300) 0.010 0.050 0 0.008

3 [300, 600) 0.021 0.048 0 0.011

4 [600,1000] 0.026 0.047 0.008

5 ALL 0.011 0.049 00 0.009

$p_{nsp}$, $t=skew$

*n* ks.D p.05 p.01

1 [ 20, 80) 0.012 0.053 0.010

2 [ 80, 300) 0.010 0.053 0.011

3 [300, 600) 0.016 0.046 0.010

4 [600,1000] 0.014 0.044 0.009

5 ALL 0.006 0.049 00 0.010

$p_{nsp}$, $t=kurt$

*n* ks.D p.05 p.01

1 [ 20, 80) 0.011 0.048 0 0.011

2 [ 80, 300) 0.020 0.051 0 0.011

3 [300, 600) 0.022 0.045 0.009

4 [600,1000] 0.025 0.049 0 0.009

5 ALL 0.009 0.048 0 0.010

$p_{nsp}$, $t=Z_{a}$

*n* ks.D p.05 p.01

1 [ 20, 80) 0.014 0.050 0 0.009

2 [ 80, 300) 0.011 0.050 0 0.012

3 [300, 600) 0.018 0.048 0 0.008

4 [600,1000] 0.021 0.055 0.014 (*)

5 ALL 0.008 0.050 00 0.011

$p_{sp}$, $t=mean$

*n* ks.D p.05 p.01

1 [ 20, 80) 0.016 0.050 0 0.008

2 [ 80, 300) 0.019 0.052 0.010

3 [300, 600) 0.027 * 0.046 0 0.009

4 [600,1000] 0.017 0.051 0.005 *,-

5 ALL 0.013 (*) 0.050 00 0.008 (*)

$p_{sp}$, $t=var$

*n* ks.D p.05 p.01

1 [ 20, 80) 0.015 0.049 0 0.010

2 [ 80, 300) 0.010 0.050 0 0.007

3 [300, 600) 0.021 0.049 0 0.011

4 [600,1000] 0.027 0.046 0.007

5 ALL 0.011 0.049 00 0.009

$p_{sp}$, $t=p_{0}$

*n* ks.D p.05 p.01

1 [ 20, 80) 0.011 0.046 0.009

2 [ 80, 300) 0.019 0.048 0 0.011

3 [300, 600) 0.024 (*) 0.042 (*) 0.009

4 [600,1000] 0.022 0.057 0.009

5 ALL 0.006 0.048 0 0.009

$p_{sp}$, $d=meanc$

*n* ks.D p.05 p.01

1 [ 20, 80) 0.016 0.051 0 0.007 (*)

2 [ 80, 300) 0.018 0.051 0 0.010

3 [300, 600) 0.026 * 0.046 0.009

4 [600,1000] 0.018 0.054 0.005 **,-

5 ALL 0.013 (*) 0.050 00 0.008 *

$p_{sp}$, $d=varc$

*n* ks.D p.05 p.01

1 [ 20, 80) 0.014 0.049 0 0.012

2 [ 80, 300) 0.010 0.050 0 0.008

3 [300, 600) 0.022 0.050 0 0.011

4 [600,1000] 0.026 0.047 0.008

5 ALL 0.011 0.049 00 0.010 0

$p_{sp}$, $d=LL$

*n* ks.D p.05 p.01

1 [ 20, 80) 0.019 0.047 0 0.011

2 [ 80, 300) 0.011 0.051 0 0.009

3 [300, 600) 0.022 0.048 0 0.012

4 [600,1000] 0.024 0.045 0.007

5 ALL 0.012 0.048 0 0.010 0

## Case 3: Bernoulli

$p_{nsp}$, $t=mean$

*n* ks.D p.05 p.01

1 [ 20, 80) 0.015 0.049 0 0.009

2 [ 80, 300) 0.010 0.056 0.013

3 [300, 600) 0.009 0.047 0 0.011

4 [600,1000] 0.018 0.049 0 0.012

5 ALL 0.005 0.050 00 0.011

$p_{nsp}$, $t=var$

*n* ks.D p.05 p.01

1 [ 20, 80) 0.026 (*) 0.054 0.009

2 [ 80, 300) 0.012 0.045 0.009

3 [300, 600) 0.018 0.055 0.012

4 [600,1000] 0.015 0.054 0.009

5 ALL 0.013 (*) 0.052 0 0.010 0

$p_{nsp}$, $t=skew$

*n* ks.D p.05 p.01

1 [ 20, 80) 0.027 * 0.057 (*) 0.013

2 [ 80, 300) 0.016 0.047 0 0.013

3 [300, 600) 0.013 0.046 0.011

4 [600,1000] 0.021 0.056 0.007

5 ALL 0.008 0.051 0 0.011

$p_{nsp}$, $t=kurt$

*n* ks.D p.05 p.01

1 [ 20, 80) 0.015 0.049 0 0.014 (*)

2 [ 80, 300) 0.012 0.047 0 0.009

3 [300, 600) 0.018 0.045 0.012

4 [600,1000] 0.014 0.051 0 0.011

5 ALL 0.007 0.048 0 0.011

$p_{nsp}$, $t=Z_{a}$

*n* ks.D p.05 p.01

1 [ 20, 80) 0.021 0.051 0 0.012

2 [ 80, 300) 0.009 0.049 0 0.009

3 [300, 600) 0.011 0.045 0.009

4 [600,1000] 0.022 0.055 0.011

5 ALL 0.010 0.050 00 0.010

$p_{sp}$, $t=mean$

*n* ks.D p.05 p.01

1 [ 20, 80) 0.012 0.048 0 0.010

2 [ 80, 300) 0.011 0.053 0.011

3 [300, 600) 0.009 0.047 0 0.011

4 [600,1000] 0.013 0.056 0.009

5 ALL 0.006 0.051 00 0.011

$p_{sp}$, $t=var$

*n* ks.D p.05 p.01

1 [ 20, 80) 0.027 * 0.054 0.011

2 [ 80, 300) 0.014 0.049 0 0.009

3 [300, 600) 0.023 0.047 0 0.011

4 [600,1000] 0.022 0.052 0.010

5 ALL 0.005 0.050 00 0.010

$p_{sp}$, $t=p_{0}$

*n* ks.D p.05 p.01

1 [ 20, 80) 0.009 0.046 0.009

2 [ 80, 300) 0.015 0.049 0 0.012

3 [300, 600) 0.012 0.048 0 0.011

4 [600,1000] 0.013 0.056 0.011

5 ALL 0.006 0.049 00 0.011

$p_{sp}$, $d=meanc$

*n* ks.D p.05 p.01

1 [ 20, 80) 0.012 0.048 0 0.010

2 [ 80, 300) 0.011 0.053 0.011

3 [300, 600) 0.009 0.047 0 0.011

4 [600,1000] 0.013 0.056 0.009

5 ALL 0.006 0.051 00 0.011

$p_{sp}$, $d=varc$

*n* ks.D p.05 p.01

1 [ 20, 80) 0.028 * 0.053 0.010

2 [ 80, 300) 0.013 0.051 0 0.008

3 [300, 600) 0.024 (*) 0.047 0 0.011

4 [600,1000] 0.022 0.052 0.009

5 ALL 0.007 0.051 00 0.010 0

$p_{sp}$, $d=LL$

*n* ks.D p.05 p.01

1 [ 20, 80) 0.018 0.047 0 0.009

2 [ 80, 300) 0.016 0.053 0.011

3 [300, 600) 0.025 (*) 0.049 0 0.011

4 [600,1000] 0.022 0.055 0.010

5 ALL 0.007 0.051 00 0.010
