# Supplementary material for: A New Calibrated Bayesian Internal Goodness-of-Fit Method: Sampled Posterior p-Values as Simple and General p-Values That Allow Double Use of the Data
Source: PLoS One. 2011 Mar 18;6(3):e14770. doi: 10.1371/journal.pone.0014770 (PMC3060804; doi:10.1371/journal.pone.0014770)
Supplement: Text S2 — Results of Scenario 2. (0.37 MB DOC) [file pone.0014770.s002.doc]

New Calibrated Bayesian Internal Goodness-of-Fit Methods: Sampled Posterior P-values as Simple and General P-values that Allow Double Use of the Data

Frédéric Gosselin

Cemagref, UR EFNO, F-45290 Nogent-sur-Vernisson, France

E-mail: [frederic.gosselin@cemagref.fr](mailto:frederic.gosselin@cemagref.fr)

*Results of Scenario 2*

# Text S2. Scenario 2 $p_{sp}$ and $p_{nsp}$ results

We here display the results for Scenario 2:

Scenario 2. The setting is the same as in Scenario 1, except that $\rho_{0}$ is replaced in the statistical model by $\mu_{\sigma_{0}}\rho_{0}$ in the Poisson and normal cases and by $\left(\rho_{0}-1\right)/\mu_{\sigma_{0}}+1$ in the Bernoulli case, where $\text{log}\left(\mu_{\sigma_{0}}\right)\sim\text{Normal}\left(\text{0, 1.4}^{2}\right)$;

Simulations were performed on $p_{sp}$ and $p_{nsp}$. In the following tables, we display the Kolmogorow-Smirnov statistic of the comparison of the p-values with a uniform distribution (ks.D), the proportion of values in the 5% extreme positions on the interval [0;1] (p.05), and the same for 1% (p.01), according to the interval to which $\mu_{\sigma_{0}}$ (in rows) and the sample size *n* (in columns) belonged. 100,000 data set sampling and analyzes were performed. The notation for the significance of the tests is the same as in Supplementary Text S1.

## Poisson models

$p_{nsp}$, $t=mean$

+-------------+-----------------+-----------------+-----------------+-----------------+-----------------+

| | [ 20, 80) | [ 80, 300) | [300, 600) | [600,1000] | ALL |

+-------------+-----------------+-----------------+-----------------+-----------------+-----------------+

|[0.00, 0.40)| ks.D=0.102 *** | ks.D=0.082 *** | ks.D=0.052 *** | ks.D=0.040 *** | ks.D=0.069 *** |

| | p.05=0.167***,++| p.05=0.134***,++| p.05=0.098***,++| p.05=0.086***,++| p.05=0.123***,++|

| | p.01=0.076***,++| p.01=0.060***,++| p.01=0.038***,++| p.01=0.028***,++| p.01=0.051***,++|

+-------------+-----------------+-----------------+-----------------+-----------------+-----------------+

|[0.40, 1.01)| ks.D=0.021 ** | ks.D=0.014 | ks.D=0.017 * | ks.D=0.014 | ks.D=0.012 ** |

| | p.05=0.059 ** | p.05=0.055 (*) | p.05=0.049 0 | p.05=0.051 0 | p.05=0.053 *,0 |

| | p.01=0.013 * | p.01=0.010 | p.01=0.010 | p.01=0.011 | p.01=0.011 0 |

+-------------+-----------------+-----------------+-----------------+-----------------+-----------------+

|[1.01, 2.59)| ks.D=0.014 | ks.D=0.010 | ks.D=0.011 | ks.D=0.017 (*) | ks.D=0.010 * |

| | p.05=0.046 0 | p.05=0.051 0 | p.05=0.050 0 | p.05=0.049 0 | p.05=0.049 00 |

| | p.01=0.009 | p.01=0.012 | p.01=0.010 | p.01=0.011 | p.01=0.010 0 |

+-------------+-----------------+-----------------+-----------------+-----------------+-----------------+

|[2.59,530.25]| ks.D=0.010 | ks.D=0.016 | ks.D=0.015 (*) | ks.D=0.015 | ks.D=0.011 ** |

| | p.05=0.051 0 | p.05=0.049 0 | p.05=0.049 0 | p.05=0.047 0 | p.05=0.049 00 |

| | p.01=0.009 | p.01=0.010 | p.01=0.011 | p.01=0.010 | p.01=0.010 0 |

+-------------+-----------------+-----------------+-----------------+-----------------+-----------------+

|ALL | ks.D=0.029 *** | ks.D=0.018 *** | ks.D=0.013 *** | ks.D=0.008 | ks.D=0.016 *** |

| | p.05=0.081***,++| p.05=0.072***,++| p.05=0.062***,+ | p.05=0.058***,+ | p.05=0.069***,++|

| | p.01=0.027***,++| p.01=0.023***,++| p.01=0.017***,++| p.01=0.015***,++| p.01=0.021***,++|

+-------------+-----------------+-----------------+-----------------+-----------------+-----------------+

$p_{nsp}$, $t=var$

+-------------+-----------------+-----------------+-----------------+-----------------+-----------------+

| | [ 20, 80) | [ 80, 300) | [300, 600) | [600,1000] | ALL |

+-------------+-----------------+-----------------+-----------------+-----------------+-----------------+

|[0.00, 0.40)| ks.D=0.026 ** | ks.D=0.025 ** | ks.D=0.032 *** | ks.D=0.009 | ks.D=0.021 *** |

| | p.05=0.058 ** | p.05=0.056 * | p.05=0.054 0 | p.05=0.058 ** | p.05=0.056***,0 |

| | p.01=0.012 | p.01=0.010 | p.01=0.011 | p.01=0.011 | p.01=0.011 (*) |

+-------------+-----------------+-----------------+-----------------+-----------------+-----------------+

|[0.40, 1.01)| ks.D=0.008 | ks.D=0.008 | ks.D=0.011 | ks.D=0.007 | ks.D=0.004 |

| | p.05=0.047 0 | p.05=0.048 0 | p.05=0.050 0 | p.05=0.053 0 | p.05=0.049 00 |

| | p.01=0.008 | p.01=0.011 | p.01=0.011 | p.01=0.011 | p.01=0.010 0 |

+-------------+-----------------+-----------------+-----------------+-----------------+-----------------+

|[1.01, 2.59)| ks.D=0.008 | ks.D=0.007 | ks.D=0.016 * | ks.D=0.012 | ks.D=0.007 |

| | p.05=0.044 *,0 | p.05=0.053 0 | p.05=0.049 0 | p.05=0.048 0 | p.05=0.049 00 |

| | p.01=0.008 * | p.01=0.009 | p.01=0.008 | p.01=0.009 | p.01=0.008 ** |

+-------------+-----------------+-----------------+-----------------+-----------------+-----------------+

|[2.59,530.25]| ks.D=0.012 | ks.D=0.008 | ks.D=0.010 | ks.D=0.016 | ks.D=0.005 |

| | p.05=0.052 0 | p.05=0.051 0 | p.05=0.046(*),0 | p.05=0.055 (*) | p.05=0.051 00 |

| | p.01=0.013 * | p.01=0.012 | p.01=0.010 | p.01=0.011 | p.01=0.012 * |

+-------------+-----------------+-----------------+-----------------+-----------------+-----------------+

|ALL | ks.D=0.007 | ks.D=0.008 (*) | ks.D=0.010 * | ks.D=0.006 | ks.D=0.005 * |

| | p.05=0.050 00 | p.05=0.052 00 | p.05=0.050 00 | p.05=0.053 *,0 | p.05=0.051(*),00|

| | p.01=0.010 0 | p.01=0.010 0 | p.01=0.010 0 | p.01=0.010 0 | p.01=0.010 00 |

+-------------+-----------------+-----------------+-----------------+-----------------+-----------------+

$p_{nsp}$, $t=skew$

+-------------+-----------------+-----------------+-----------------+-----------------+-----------------+

| | [ 20, 80) | [ 80, 300) | [300, 600) | [600,1000] | ALL |

+-------------+-----------------+-----------------+-----------------+-----------------+-----------------+

|[0.00, 0.40)| ks.D=0.014 | ks.D=0.006 | ks.D=0.014 | ks.D=0.015 | ks.D=0.010 * |

| | p.05=0.047 0 | p.05=0.053 0 | p.05=0.051 0 | p.05=0.048 0 | p.05=0.050 00 |

| | p.01=0.008 | p.01=0.010 | p.01=0.010 | p.01=0.008 (*) | p.01=0.009 0 |

+-------------+-----------------+-----------------+-----------------+-----------------+-----------------+

|[0.40, 1.01)| ks.D=0.010 | ks.D=0.010 | ks.D=0.009 | ks.D=0.012 | ks.D=0.006 |

| | p.05=0.051 0 | p.05=0.046 0 | p.05=0.050 0 | p.05=0.048 0 | p.05=0.049 00 |

| | p.01=0.010 | p.01=0.009 | p.01=0.010 | p.01=0.009 | p.01=0.009 0 |

+-------------+-----------------+-----------------+-----------------+-----------------+-----------------+

|[1.01, 2.59)| ks.D=0.008 | ks.D=0.010 | ks.D=0.013 | ks.D=0.010 | ks.D=0.004 |

| | p.05=0.048 0 | p.05=0.051 0 | p.05=0.048 0 | p.05=0.048 0 | p.05=0.049 00 |

| | p.01=0.010 | p.01=0.010 | p.01=0.008 | p.01=0.009 | p.01=0.009 0 |

+-------------+-----------------+-----------------+-----------------+-----------------+-----------------+

|[2.59,530.25]| ks.D=0.013 | ks.D=0.015 | ks.D=0.013 | ks.D=0.009 | ks.D=0.004 |

| | p.05=0.048 0 | p.05=0.048 0 | p.05=0.049 0 | p.05=0.051 0 | p.05=0.049 00 |

| | p.01=0.009 | p.01=0.010 | p.01=0.011 | p.01=0.011 | p.01=0.010 0 |

+-------------+-----------------+-----------------+-----------------+-----------------+-----------------+

|ALL | ks.D=0.006 | ks.D=0.004 | ks.D=0.005 | ks.D=0.004 | ks.D=0.002 |

| | p.05=0.048 00 | p.05=0.049 00 | p.05=0.050 00 | p.05=0.049 00 | p.05=0.049 00 |

| | p.01=0.009 0 | p.01=0.010 0 | p.01=0.010 0 | p.01=0.009 0 | p.01=0.010 00 |

+-------------+-----------------+-----------------+-----------------+-----------------+-----------------+

$p_{nsp}$, $t=kurt$

+-------------+-----------------+-----------------+-----------------+-----------------+-----------------+

| | [ 20, 80) | [ 80, 300) | [300, 600) | [600,1000] | ALL |

+-------------+-----------------+-----------------+-----------------+-----------------+-----------------+

|[0.00, 0.40)| ks.D=0.011 | ks.D=0.009 | ks.D=0.013 | ks.D=0.010 | ks.D=0.006 |

| | p.05=0.052 0 | p.05=0.054 0 | p.05=0.053 0 | p.05=0.056 (*) | p.05=0.054 **,0 |

| | p.01=0.011 | p.01=0.013 (*) | p.01=0.009 | p.01=0.011 | p.01=0.011 0 |

+-------------+-----------------+-----------------+-----------------+-----------------+-----------------+

|[0.40, 1.01)| ks.D=0.013 | ks.D=0.015 | ks.D=0.006 | ks.D=0.008 | ks.D=0.006 |

| | p.05=0.057 * | p.05=0.057 ** | p.05=0.047 0 | p.05=0.053 0 | p.05=0.053 *,0 |

| | p.01=0.011 | p.01=0.013 (*) | p.01=0.012 | p.01=0.009 | p.01=0.011 (*) |

+-------------+-----------------+-----------------+-----------------+-----------------+-----------------+

|[1.01, 2.59)| ks.D=0.010 | ks.D=0.008 | ks.D=0.011 | ks.D=0.015 | ks.D=0.004 |

| | p.05=0.048 0 | p.05=0.048 0 | p.05=0.052 0 | p.05=0.049 0 | p.05=0.049 00 |

| | p.01=0.011 | p.01=0.009 | p.01=0.011 | p.01=0.010 | p.01=0.010 0 |

+-------------+-----------------+-----------------+-----------------+-----------------+-----------------+

|[2.59,530.25]| ks.D=0.015 (*) | ks.D=0.007 | ks.D=0.011 | ks.D=0.011 | ks.D=0.007 |

| | p.05=0.050 0 | p.05=0.048 0 | p.05=0.054(*),0 | p.05=0.050 0 | p.05=0.051 00 |

| | p.01=0.009 | p.01=0.009 | p.01=0.011 | p.01=0.010 | p.01=0.010 0 |

+-------------+-----------------+-----------------+-----------------+-----------------+-----------------+

|ALL | ks.D=0.007 | ks.D=0.005 | ks.D=0.003 | ks.D=0.007 | ks.D=0.003 |

| | p.05=0.052 00 | p.05=0.052 00 | p.05=0.052 00 | p.05=0.052 00 | p.05=0.052**,00 |

| | p.01=0.010 0 | p.01=0.011 0 | p.01=0.011 0 | p.01=0.010 0 | p.01=0.010 00 |

+-------------+-----------------+-----------------+-----------------+-----------------+-----------------+

$p_{nsp}$, $t=Z_{a}$

+-------------+-----------------+-----------------+-----------------+-----------------+-----------------+

| | [ 20, 80) | [ 80, 300) | [300, 600) | [600,1000] | ALL |

+-------------+-----------------+-----------------+-----------------+-----------------+-----------------+

|[0.00, 0.40)| ks.D=0.131 *** | ks.D=0.098 *** | ks.D=0.057 *** | ks.D=0.038 *** | ks.D=0.080 *** |

| | p.05=0.119***,++| p.05=0.101***,++| p.05=0.076***,++| p.05=0.064***,+ | p.05=0.091***,++|

| | p.01=0.054***,++| p.01=0.040***,++| p.01=0.026***,++| p.01=0.016***,++| p.01=0.035***,++|

+-------------+-----------------+-----------------+-----------------+-----------------+-----------------+

|[0.40, 1.01)| ks.D=0.012 | ks.D=0.008 | ks.D=0.009 | ks.D=0.009 | ks.D=0.005 |

| | p.05=0.052 0 | p.05=0.052 0 | p.05=0.046 0 | p.05=0.047 0 | p.05=0.049 00 |

| | p.01=0.013 * | p.01=0.010 | p.01=0.009 | p.01=0.011 | p.01=0.011 0 |

+-------------+-----------------+-----------------+-----------------+-----------------+-----------------+

|[1.01, 2.59)| ks.D=0.018 * | ks.D=0.009 | ks.D=0.010 | ks.D=0.012 | ks.D=0.010 * |

| | p.05=0.047 0 | p.05=0.055(*),0 | p.05=0.051 0 | p.05=0.048 0 | p.05=0.050 00 |

| | p.01=0.008 | p.01=0.011 | p.01=0.010 | p.01=0.008 | p.01=0.009 0 |

+-------------+-----------------+-----------------+-----------------+-----------------+-----------------+

|[2.59,530.25]| ks.D=0.008 | ks.D=0.009 | ks.D=0.018 * | ks.D=0.016 | ks.D=0.009 * |

| | p.05=0.052 0 | p.05=0.053 0 | p.05=0.052 0 | p.05=0.049 0 | p.05=0.052 00 |

| | p.01=0.010 | p.01=0.010 | p.01=0.011 | p.01=0.011 | p.01=0.011 0 |

+-------------+-----------------+-----------------+-----------------+-----------------+-----------------+

|ALL | ks.D=0.033 *** | ks.D=0.023 *** | ks.D=0.014 *** | ks.D=0.010 * | ks.D=0.019 *** |

| | p.05=0.068***,++| p.05=0.065***,++| p.05=0.056***,0 | p.05=0.052 00 | p.05=0.061***,+ |

| | p.01=0.021***,++| p.01=0.018***,++| p.01=0.014***,++| p.01=0.012 * | p.01=0.016***,++|

+-------------+-----------------+-----------------+-----------------+-----------------+-----------------+

$p_{sp}$, $t=mean$

+-------------+-----------------+-----------------+-----------------+-----------------+-----------------+

| | [ 20, 80) | [ 80, 300) | [300, 600) | [600,1000] | ALL |

+-------------+-----------------+-----------------+-----------------+-----------------+-----------------+

|[0.00, 0.40)| ks.D=0.106 *** | ks.D=0.085 *** | ks.D=0.056 *** | ks.D=0.041 *** | ks.D=0.071 *** |

| | p.05=0.174***,++| p.05=0.140***,++| p.05=0.101***,++| p.05=0.083***,++| p.05=0.126***,++|

| | p.01=0.085***,++| p.01=0.065***,++| p.01=0.043***,++| p.01=0.028***,++| p.01=0.056***,++|

+-------------+-----------------+-----------------+-----------------+-----------------+-----------------+

|[0.40, 1.01)| ks.D=0.020 * | ks.D=0.010 | ks.D=0.017 * | ks.D=0.014 | ks.D=0.010 * |

| | p.05=0.057 ** | p.05=0.057 * | p.05=0.049 0 | p.05=0.048 0 | p.05=0.053 *,0 |

| | p.01=0.014 **,+ | p.01=0.010 | p.01=0.010 | p.01=0.010 | p.01=0.011 |

+-------------+-----------------+-----------------+-----------------+-----------------+-----------------+

|[1.01, 2.59)| ks.D=0.009 | ks.D=0.008 | ks.D=0.012 | ks.D=0.018 (*) | ks.D=0.009 * |

| | p.05=0.047 0 | p.05=0.051 0 | p.05=0.049 0 | p.05=0.048 0 | p.05=0.049 00 |

| | p.01=0.010 | p.01=0.012 | p.01=0.009 | p.01=0.011 | p.01=0.010 0 |

+-------------+-----------------+-----------------+-----------------+-----------------+-----------------+

|[2.59,530.25]| ks.D=0.011 | ks.D=0.016 (*) | ks.D=0.017 * | ks.D=0.014 | ks.D=0.011 ** |

| | p.05=0.051 0 | p.05=0.049 0 | p.05=0.048 0 | p.05=0.047 0 | p.05=0.049 00 |

| | p.01=0.009 | p.01=0.011 | p.01=0.010 | p.01=0.010 | p.01=0.010 0 |

+-------------+-----------------+-----------------+-----------------+-----------------+-----------------+

|ALL | ks.D=0.030 *** | ks.D=0.020 *** | ks.D=0.014 *** | ks.D=0.009 (*) | ks.D=0.018 *** |

| | p.05=0.083***,++| p.05=0.074***,++| p.05=0.062***,+ | p.05=0.057***,0 | p.05=0.069***,++|

| | p.01=0.030***,++| p.01=0.024***,++| p.01=0.018***,++| p.01=0.015***,++| p.01=0.022***,++|

+-------------+-----------------+-----------------+-----------------+-----------------+-----------------+

$p_{sp}$, $t=var$

+-------------+-----------------+-----------------+-----------------+-----------------+-----------------+

| | [ 20, 80) | [ 80, 300) | [300, 600) | [600,1000] | ALL |

+-------------+-----------------+-----------------+-----------------+-----------------+-----------------+

|[0.00, 0.40)| ks.D=0.052 *** | ks.D=0.044 *** | ks.D=0.041 *** | ks.D=0.017 (*) | ks.D=0.037 *** |

| | p.05=0.083***,++| p.05=0.075***,++| p.05=0.069***,++| p.05=0.056 * | p.05=0.072***,++|

| | p.01=0.024***,++| p.01=0.021***,++| p.01=0.017***,++| p.01=0.014 **,+ | p.01=0.019***,++|

+-------------+-----------------+-----------------+-----------------+-----------------+-----------------+

|[0.40, 1.01)| ks.D=0.015 (*) | ks.D=0.010 | ks.D=0.018 * | ks.D=0.010 | ks.D=0.009 * |

| | p.05=0.045(*),0 | p.05=0.054 0 | p.05=0.051 0 | p.05=0.047 0 | p.05=0.050 00 |

| | p.01=0.010 | p.01=0.009 | p.01=0.010 | p.01=0.012 | p.01=0.010 0 |

+-------------+-----------------+-----------------+-----------------+-----------------+-----------------+

|[1.01, 2.59)| ks.D=0.012 | ks.D=0.010 | ks.D=0.010 | ks.D=0.011 | ks.D=0.007 |

| | p.05=0.046 0 | p.05=0.054 0 | p.05=0.049 0 | p.05=0.052 0 | p.05=0.050 00 |

| | p.01=0.007 **,- | p.01=0.010 | p.01=0.010 | p.01=0.009 | p.01=0.009 (*) |

+-------------+-----------------+-----------------+-----------------+-----------------+-----------------+

|[2.59,530.25]| ks.D=0.014 | ks.D=0.015 | ks.D=0.010 | ks.D=0.017 | ks.D=0.010 ** |

| | p.05=0.050 0 | p.05=0.051 0 | p.05=0.047 0 | p.05=0.051 0 | p.05=0.049 00 |

| | p.01=0.011 | p.01=0.012 | p.01=0.009 | p.01=0.007 * | p.01=0.010 0 |

+-------------+-----------------+-----------------+-----------------+-----------------+-----------------+

|ALL | ks.D=0.013 ** | ks.D=0.011 ** | ks.D=0.011 ** | ks.D=0.004 | ks.D=0.008 *** |

| | p.05=0.056***,0 | p.05=0.059***,+ | p.05=0.054 **,0 | p.05=0.052 00 | p.05=0.055***,0 |

| | p.01=0.013***,+ | p.01=0.013***,+ | p.01=0.012 ** | p.01=0.011 0 | p.01=0.012***,+ |

+-------------+-----------------+-----------------+-----------------+-----------------+-----------------+

$p_{sp}$, $t=p_{0}$

+-------------+-----------------+-----------------+-----------------+-----------------+-----------------+

| | [ 20, 80) | [ 80, 300) | [300, 600) | [600,1000] | ALL |

+-------------+-----------------+-----------------+-----------------+-----------------+-----------------+

|[0.00, 0.40)| ks.D=0.077 *** | ks.D=0.055 *** | ks.D=0.041 *** | ks.D=0.033 *** | ks.D=0.050 *** |

| | p.05=0.091***,++| p.05=0.079***,++| p.05=0.064***,+ | p.05=0.065***,+ | p.05=0.075***,++|

| | p.01=0.034***,++| p.01=0.029***,++| p.01=0.019***,++| p.01=0.016***,++| p.01=0.025***,++|

+-------------+-----------------+-----------------+-----------------+-----------------+-----------------+

|[0.40, 1.01)| ks.D=0.018 * | ks.D=0.009 | ks.D=0.010 | ks.D=0.012 | ks.D=0.008 (*) |

| | p.05=0.055(*),0 | p.05=0.049 0 | p.05=0.049 0 | p.05=0.049 0 | p.05=0.051 00 |

| | p.01=0.010 | p.01=0.011 | p.01=0.011 | p.01=0.011 | p.01=0.011 |

+-------------+-----------------+-----------------+-----------------+-----------------+-----------------+

|[1.01, 2.59)| ks.D=0.012 | ks.D=0.008 | ks.D=0.013 | ks.D=0.014 | ks.D=0.009 (*) |

| | p.05=0.049 0 | p.05=0.050 0 | p.05=0.046 0 | p.05=0.048 0 | p.05=0.048 00 |

| | p.01=0.009 | p.01=0.008 | p.01=0.009 | p.01=0.010 | p.01=0.009 0 |

+-------------+-----------------+-----------------+-----------------+-----------------+-----------------+

|[2.59,530.25]| ks.D=0.020 ** | ks.D=0.026 ** | ks.D=0.018 * | ks.D=0.013 | ks.D=0.015 *** |

| | p.05=0.050 0 | p.05=0.049 0 | p.05=0.049 0 | p.05=0.049 0 | p.05=0.049 00 |

| | p.01=0.009 | p.01=0.010 | p.01=0.011 | p.01=0.011 | p.01=0.010 0 |

+-------------+-----------------+-----------------+-----------------+-----------------+-----------------+

|ALL | ks.D=0.019 *** | ks.D=0.014 ** | ks.D=0.009 * | ks.D=0.011 * | ks.D=0.012 *** |

| | p.05=0.061***,+ | p.05=0.057***,0 | p.05=0.052(*),00| p.05=0.053(*),0 | p.05=0.056***,0 |

| | p.01=0.016***,++| p.01=0.014***,++| p.01=0.013***,+ | p.01=0.012 ** | p.01=0.014***,++|

+-------------+-----------------+-----------------+-----------------+-----------------+-----------------+

$p_{sp}$, $d=meanc$

+-------------+-----------------+-----------------+-----------------+-----------------+-----------------+

| | [ 20, 80) | [ 80, 300) | [300, 600) | [600,1000] | ALL |

+-------------+-----------------+-----------------+-----------------+-----------------+-----------------+

|[0.00, 0.40)| ks.D=0.106 *** | ks.D=0.085 *** | ks.D=0.056 *** | ks.D=0.041 *** | ks.D=0.071 *** |

| | p.05=0.174***,++| p.05=0.140***,++| p.05=0.101***,++| p.05=0.083***,++| p.05=0.126***,++|

| | p.01=0.085***,++| p.01=0.065***,++| p.01=0.043***,++| p.01=0.028***,++| p.01=0.056***,++|

+-------------+-----------------+-----------------+-----------------+-----------------+-----------------+

|[0.40, 1.01)| ks.D=0.020 * | ks.D=0.010 | ks.D=0.017 * | ks.D=0.014 | ks.D=0.010 * |

| | p.05=0.057 ** | p.05=0.057 * | p.05=0.049 0 | p.05=0.048 0 | p.05=0.053 *,0 |

| | p.01=0.014 **,+ | p.01=0.010 | p.01=0.010 | p.01=0.010 | p.01=0.011 |

+-------------+-----------------+-----------------+-----------------+-----------------+-----------------+

|[1.01, 2.59)| ks.D=0.009 | ks.D=0.008 | ks.D=0.012 | ks.D=0.018 (*) | ks.D=0.009 * |

| | p.05=0.047 0 | p.05=0.051 0 | p.05=0.049 0 | p.05=0.048 0 | p.05=0.049 00 |

| | p.01=0.010 | p.01=0.012 | p.01=0.009 | p.01=0.011 | p.01=0.010 0 |

+-------------+-----------------+-----------------+-----------------+-----------------+-----------------+

|[2.59,530.25]| ks.D=0.011 | ks.D=0.016 (*) | ks.D=0.017 * | ks.D=0.014 | ks.D=0.011 ** |

| | p.05=0.051 0 | p.05=0.049 0 | p.05=0.048 0 | p.05=0.047 0 | p.05=0.049 00 |

| | p.01=0.009 | p.01=0.011 | p.01=0.010 | p.01=0.010 | p.01=0.010 0 |

+-------------+-----------------+-----------------+-----------------+-----------------+-----------------+

|ALL | ks.D=0.030 *** | ks.D=0.020 *** | ks.D=0.014 *** | ks.D=0.009 (*) | ks.D=0.018 *** |

| | p.05=0.083***,++| p.05=0.074***,++| p.05=0.062***,+ | p.05=0.057***,0 | p.05=0.069***,++|

| | p.01=0.030***,++| p.01=0.024***,++| p.01=0.018***,++| p.01=0.015***,++| p.01=0.022***,++|

+-------------+-----------------+-----------------+-----------------+-----------------+-----------------+

$p_{sp}$, $d=varc$

+-------------+-----------------+-----------------+-----------------+-----------------+-----------------+

| | [ 20, 80) | [ 80, 300) | [300, 600) | [600,1000] | ALL |

+-------------+-----------------+-----------------+-----------------+-----------------+-----------------+

|[0.00, 0.40)| ks.D=0.052 *** | ks.D=0.044 *** | ks.D=0.041 *** | ks.D=0.017 (*) | ks.D=0.037 *** |

| | p.05=0.083***,++| p.05=0.075***,++| p.05=0.069***,++| p.05=0.056 * | p.05=0.072***,++|

| | p.01=0.024***,++| p.01=0.021***,++| p.01=0.017***,++| p.01=0.014 **,+ | p.01=0.019***,++|

+-------------+-----------------+-----------------+-----------------+-----------------+-----------------+

|[0.40, 1.01)| ks.D=0.015 (*) | ks.D=0.010 | ks.D=0.018 * | ks.D=0.010 | ks.D=0.009 * |

| | p.05=0.045(*),0 | p.05=0.054 0 | p.05=0.051 0 | p.05=0.047 0 | p.05=0.050 00 |

| | p.01=0.010 | p.01=0.009 | p.01=0.010 | p.01=0.012 | p.01=0.010 0 |

+-------------+-----------------+-----------------+-----------------+-----------------+-----------------+

|[1.01, 2.59)| ks.D=0.012 | ks.D=0.010 | ks.D=0.010 | ks.D=0.011 | ks.D=0.007 |

| | p.05=0.046 0 | p.05=0.054 0 | p.05=0.049 0 | p.05=0.052 0 | p.05=0.050 00 |

| | p.01=0.007 **,- | p.01=0.010 | p.01=0.010 | p.01=0.009 | p.01=0.009 (*) |

+-------------+-----------------+-----------------+-----------------+-----------------+-----------------+

|[2.59,530.25]| ks.D=0.014 | ks.D=0.015 | ks.D=0.010 | ks.D=0.017 | ks.D=0.010 ** |

| | p.05=0.050 0 | p.05=0.051 0 | p.05=0.047 0 | p.05=0.051 0 | p.05=0.049 00 |

| | p.01=0.011 | p.01=0.012 | p.01=0.009 | p.01=0.007 (*) | p.01=0.010 0 |

+-------------+-----------------+-----------------+-----------------+-----------------+-----------------+

|ALL | ks.D=0.013 ** | ks.D=0.011 ** | ks.D=0.011 ** | ks.D=0.004 | ks.D=0.008 *** |

| | p.05=0.056***,0 | p.05=0.059***,+ | p.05=0.054 **,0 | p.05=0.052 00 | p.05=0.055***,0 |

| | p.01=0.013***,+ | p.01=0.013***,+ | p.01=0.012 ** | p.01=0.011 0 | p.01=0.012***,+ |

+-------------+-----------------+-----------------+-----------------+-----------------+-----------------+

$p_{sp}$, $d=LL$

+-------------+-----------------+-----------------+-----------------+-----------------+-----------------+

| | [ 20, 80) | [ 80, 300) | [300, 600) | [600,1000] | ALL |

+-------------+-----------------+-----------------+-----------------+-----------------+-----------------+

|[0.00, 0.40)| ks.D=0.069 *** | ks.D=0.059 *** | ks.D=0.061 *** | ks.D=0.033 *** | ks.D=0.055 *** |

| | p.05=0.102***,++| p.05=0.084***,++| p.05=0.075***,++| p.05=0.063***,+ | p.05=0.082***,++|

| | p.01=0.035***,++| p.01=0.028***,++| p.01=0.020***,++| p.01=0.016***,++| p.01=0.025***,++|

+-------------+-----------------+-----------------+-----------------+-----------------+-----------------+

|[0.40, 1.01)| ks.D=0.017 (*) | ks.D=0.009 | ks.D=0.023 ** | ks.D=0.016 | ks.D=0.011 ** |

| | p.05=0.049 0 | p.05=0.053 0 | p.05=0.054 0 | p.05=0.050 0 | p.05=0.051 00 |

| | p.01=0.010 | p.01=0.012 | p.01=0.012 (*) | p.01=0.011 | p.01=0.011 * |

+-------------+-----------------+-----------------+-----------------+-----------------+-----------------+

|[1.01, 2.58)| ks.D=0.012 | ks.D=0.010 | ks.D=0.018 * | ks.D=0.014 | ks.D=0.010 * |

| | p.05=0.048 0 | p.05=0.052 0 | p.05=0.046 0 | p.05=0.054 0 | p.05=0.050 00 |

| | p.01=0.009 | p.01=0.011 | p.01=0.009 | p.01=0.011 | p.01=0.010 0 |

+-------------+-----------------+-----------------+-----------------+-----------------+-----------------+

|[2.58,530.25]| ks.D=0.022 ** | ks.D=0.019 * | ks.D=0.014 | ks.D=0.021 * | ks.D=0.015 *** |

| | p.05=0.053 0 | p.05=0.053 0 | p.05=0.047 0 | p.05=0.051 0 | p.05=0.051 00 |

| | p.01=0.011 | p.01=0.012 | p.01=0.008 | p.01=0.008 (*) | p.01=0.010 0 |

+-------------+-----------------+-----------------+-----------------+-----------------+-----------------+

|ALL | ks.D=0.018 *** | ks.D=0.012 ** | ks.D=0.015 *** | ks.D=0.007 | ks.D=0.012 *** |

| | p.05=0.063***,++| p.05=0.060***,+ | p.05=0.056***,0 | p.05=0.054 **,0 | p.05=0.058***,+ |

| | p.01=0.016***,++| p.01=0.015***,++| p.01=0.013***,+ | p.01=0.012 * | p.01=0.014***,++|

+-------------+-----------------+-----------------+-----------------+-----------------+-----------------+

## Normal models

$p_{nsp}$, $t=mean$

+-------------+-----------------+-----------------+-----------------+-----------------+-----------------+

| | [ 20, 80) | [ 80, 300) | [300, 600) | [600,1000] | ALL |

+-------------+-----------------+-----------------+-----------------+-----------------+-----------------+

|[0.00, 0.40)| ks.D=0.010 | ks.D=0.009 | ks.D=0.011 | ks.D=0.018 (*) | ks.D=0.008 (*) |

| | p.05=0.052 0 | p.05=0.052 0 | p.05=0.048 0 | p.05=0.054 0 | p.05=0.051 00 |

| | p.01=0.012 | p.01=0.009 | p.01=0.009 | p.01=0.010 | p.01=0.010 0 |

+-------------+-----------------+-----------------+-----------------+-----------------+-----------------+

|[0.40, 1.01)| ks.D=0.014 | ks.D=0.010 | ks.D=0.011 | ks.D=0.015 | ks.D=0.005 |

| | p.05=0.049 0 | p.05=0.051 0 | p.05=0.050 0 | p.05=0.045 0 | p.05=0.049 00 |

| | p.01=0.010 | p.01=0.009 | p.01=0.012 | p.01=0.008 | p.01=0.010 0 |

+-------------+-----------------+-----------------+-----------------+-----------------+-----------------+

|[1.01, 2.58)| ks.D=0.015 | ks.D=0.009 | ks.D=0.008 | ks.D=0.013 | ks.D=0.004 |

| | p.05=0.056 * | p.05=0.044 *,0 | p.05=0.052 0 | p.05=0.052 0 | p.05=0.051 00 |

| | p.01=0.012 | p.01=0.010 | p.01=0.012 (*) | p.01=0.011 | p.01=0.011 * |

+-------------+-----------------+-----------------+-----------------+-----------------+-----------------+

|[2.58,372.79]| ks.D=0.008 | ks.D=0.007 | ks.D=0.021 ** | ks.D=0.012 | ks.D=0.006 |

| | p.05=0.048 0 | p.05=0.048 0 | p.05=0.050 0 | p.05=0.052 0 | p.05=0.049 00 |

| | p.01=0.012 | p.01=0.012 | p.01=0.009 | p.01=0.009 | p.01=0.011 0 |

+-------------+-----------------+-----------------+-----------------+-----------------+-----------------+

|ALL | ks.D=0.004 | ks.D=0.004 | ks.D=0.007 | ks.D=0.006 | ks.D=0.004 (*) |

| | p.05=0.051 00 | p.05=0.049 00 | p.05=0.050 00 | p.05=0.051 00 | p.05=0.050 00 |

| | p.01=0.011 (*) | p.01=0.010 0 | p.01=0.011 0 | p.01=0.010 0 | p.01=0.010 00 |

+-------------+-----------------+-----------------+-----------------+-----------------+-----------------+

$p_{nsp}$, $t=var$

+-------------+-----------------+-----------------+-----------------+-----------------+-----------------+

| | [ 20, 80) | [ 80, 300) | [300, 600) | [600,1000] | ALL |

+-------------+-----------------+-----------------+-----------------+-----------------+-----------------+

|[0.00, 0.40)| ks.D=0.107 *** | ks.D=0.089 *** | ks.D=0.061 *** | ks.D=0.041 *** | ks.D=0.073 *** |

| | p.05=0.169***,++| p.05=0.156***,++| p.05=0.128***,++| p.05=0.101***,++| p.05=0.140***,++|

| | p.01=0.075***,++| p.01=0.080***,++| p.01=0.060***,++| p.01=0.040***,++| p.01=0.065***,++|

+-------------+-----------------+-----------------+-----------------+-----------------+-----------------+

|[0.40, 1.01)| ks.D=0.025 ** | ks.D=0.014 | ks.D=0.010 | ks.D=0.014 | ks.D=0.010 * |

| | p.05=0.058 ** | p.05=0.056 (*) | p.05=0.052 0 | p.05=0.052 0 | p.05=0.054 **,0 |

| | p.01=0.016***,++| p.01=0.012 | p.01=0.010 | p.01=0.009 | p.01=0.012 ** |

+-------------+-----------------+-----------------+-----------------+-----------------+-----------------+

|[1.01, 2.58)| ks.D=0.011 | ks.D=0.007 | ks.D=0.013 | ks.D=0.018 (*) | ks.D=0.003 |

| | p.05=0.050 0 | p.05=0.047 0 | p.05=0.047 0 | p.05=0.048 0 | p.05=0.048 00 |

| | p.01=0.010 | p.01=0.009 | p.01=0.010 | p.01=0.008 | p.01=0.009 0 |

+-------------+-----------------+-----------------+-----------------+-----------------+-----------------+

|[2.58,372.79]| ks.D=0.018 * | ks.D=0.016 (*) | ks.D=0.008 | ks.D=0.012 | ks.D=0.004 |

| | p.05=0.048 0 | p.05=0.048 0 | p.05=0.048 0 | p.05=0.051 0 | p.05=0.049 00 |

| | p.01=0.009 | p.01=0.010 | p.01=0.009 | p.01=0.008 (*) | p.01=0.009 0 |

+-------------+-----------------+-----------------+-----------------+-----------------+-----------------+

|ALL | ks.D=0.029 *** | ks.D=0.025 *** | ks.D=0.019 *** | ks.D=0.010 * | ks.D=0.021 *** |

| | p.05=0.081***,++| p.05=0.077***,++| p.05=0.069***,++| p.05=0.063***,++| p.05=0.073***,++|

| | p.01=0.027***,++| p.01=0.028***,++| p.01=0.022***,++| p.01=0.016***,++| p.01=0.024***,++|

+-------------+-----------------+-----------------+-----------------+-----------------+-----------------+

$p_{nsp}$, $t=skew$

+-------------+-----------------+-----------------+-----------------+-----------------+-----------------+

| | [ 20, 80) | [ 80, 300) | [300, 600) | [600,1000] | ALL |

+-------------+-----------------+-----------------+-----------------+-----------------+-----------------+

|[0.00, 0.40)| ks.D=0.010 | ks.D=0.010 | ks.D=0.010 | ks.D=0.009 | ks.D=0.004 |

| | p.05=0.050 0 | p.05=0.048 0 | p.05=0.051 0 | p.05=0.044 * | p.05=0.049 00 |

| | p.01=0.009 | p.01=0.009 | p.01=0.010 | p.01=0.009 | p.01=0.009 0 |

+-------------+-----------------+-----------------+-----------------+-----------------+-----------------+

|[0.40, 1.01)| ks.D=0.007 | ks.D=0.016 (*) | ks.D=0.008 | ks.D=0.013 | ks.D=0.007 |

| | p.05=0.051 0 | p.05=0.060 **,+ | p.05=0.048 0 | p.05=0.050 0 | p.05=0.052(*),00|

| | p.01=0.010 | p.01=0.013 * | p.01=0.008 | p.01=0.010 | p.01=0.010 0 |

+-------------+-----------------+-----------------+-----------------+-----------------+-----------------+

|[1.01, 2.58)| ks.D=0.009 | ks.D=0.008 | ks.D=0.014 | ks.D=0.012 | ks.D=0.008 |

| | p.05=0.050 0 | p.05=0.053 0 | p.05=0.051 0 | p.05=0.055 (*) | p.05=0.052 00 |

| | p.01=0.012 | p.01=0.010 | p.01=0.012 | p.01=0.013 (*) | p.01=0.012 * |

+-------------+-----------------+-----------------+-----------------+-----------------+-----------------+

|[2.58,372.79]| ks.D=0.013 | ks.D=0.011 | ks.D=0.013 | ks.D=0.011 | ks.D=0.006 |

| | p.05=0.049 0 | p.05=0.050 0 | p.05=0.050 0 | p.05=0.044 * | p.05=0.048 00 |

| | p.01=0.009 | p.01=0.009 | p.01=0.012 (*) | p.01=0.010 | p.01=0.010 0 |

+-------------+-----------------+-----------------+-----------------+-----------------+-----------------+

|ALL | ks.D=0.003 | ks.D=0.006 | ks.D=0.004 | ks.D=0.005 | ks.D=0.002 |

| | p.05=0.050 00 | p.05=0.053 *,0 | p.05=0.050 00 | p.05=0.048 00 | p.05=0.050 00 |

| | p.01=0.010 0 | p.01=0.010 0 | p.01=0.010 0 | p.01=0.010 0 | p.01=0.010 00 |

+-------------+-----------------+-----------------+-----------------+-----------------+-----------------+

$p_{nsp}$, $t=kurt$

+-------------+-----------------+-----------------+-----------------+-----------------+-----------------+

| | [ 20, 80) | [ 80, 300) | [300, 600) | [600,1000] | ALL |

+-------------+-----------------+-----------------+-----------------+-----------------+-----------------+

|[0.00, 0.40)| ks.D=0.014 | ks.D=0.007 | ks.D=0.006 | ks.D=0.014 | ks.D=0.003 |

| | p.05=0.047 0 | p.05=0.054 0 | p.05=0.046 0 | p.05=0.053 0 | p.05=0.049 00 |

| | p.01=0.007 * | p.01=0.013 *,+ | p.01=0.011 | p.01=0.013 * | p.01=0.011 |

+-------------+-----------------+-----------------+-----------------+-----------------+-----------------+

|[0.40, 1.01)| ks.D=0.013 | ks.D=0.020 * | ks.D=0.007 | ks.D=0.014 | ks.D=0.011 ** |

| | p.05=0.046 0 | p.05=0.050 0 | p.05=0.050 0 | p.05=0.052 0 | p.05=0.049 00 |

| | p.01=0.010 | p.01=0.010 | p.01=0.010 | p.01=0.008 | p.01=0.010 0 |

+-------------+-----------------+-----------------+-----------------+-----------------+-----------------+

|[1.01, 2.58)| ks.D=0.009 | ks.D=0.009 | ks.D=0.010 | ks.D=0.011 | ks.D=0.004 |

| | p.05=0.052 0 | p.05=0.051 0 | p.05=0.047 0 | p.05=0.050 0 | p.05=0.050 00 |

| | p.01=0.012 | p.01=0.010 | p.01=0.010 | p.01=0.008 (*) | p.01=0.010 0 |

+-------------+-----------------+-----------------+-----------------+-----------------+-----------------+

|[2.58,372.79]| ks.D=0.009 | ks.D=0.023 ** | ks.D=0.005 | ks.D=0.009 | ks.D=0.006 |

| | p.05=0.047 0 | p.05=0.052 0 | p.05=0.048 0 | p.05=0.051 0 | p.05=0.050 00 |

| | p.01=0.009 | p.01=0.010 | p.01=0.009 | p.01=0.008 | p.01=0.009 0 |

+-------------+-----------------+-----------------+-----------------+-----------------+-----------------+

|ALL | ks.D=0.007 | ks.D=0.009 * | ks.D=0.003 | ks.D=0.006 | ks.D=0.003 |

| | p.05=0.048 00 | p.05=0.052 00 | p.05=0.048 00 | p.05=0.051 00 | p.05=0.050 00 |

| | p.01=0.010 0 | p.01=0.011 | p.01=0.010 0 | p.01=0.009 0 | p.01=0.010 00 |

+-------------+-----------------+-----------------+-----------------+-----------------+-----------------+

$p_{nsp}$, $t=Z_{a}$

+-------------+-----------------+-----------------+-----------------+-----------------+-----------------+

| | [ 20, 80) | [ 80, 300) | [300, 600) | [600,1000] | ALL |

+-------------+-----------------+-----------------+-----------------+-----------------+-----------------+

|[0.00, 0.40)| ks.D=0.105 *** | ks.D=0.087 *** | ks.D=0.060 *** | ks.D=0.047 *** | ks.D=0.075 *** |

| | p.05=0.085***,++| p.05=0.094***,++| p.05=0.076***,++| p.05=0.069***,++| p.05=0.081***,++|

| | p.01=0.027***,++| p.01=0.033***,++| p.01=0.027***,++| p.01=0.021***,++| p.01=0.027***,++|

+-------------+-----------------+-----------------+-----------------+-----------------+-----------------+

|[0.40, 1.01)| ks.D=0.018 * | ks.D=0.017 (*) | ks.D=0.016 (*) | ks.D=0.014 | ks.D=0.009 * |

| | p.05=0.052 0 | p.05=0.049 0 | p.05=0.051 0 | p.05=0.053 0 | p.05=0.051 00 |

| | p.01=0.011 | p.01=0.010 | p.01=0.010 | p.01=0.011 | p.01=0.010 0 |

+-------------+-----------------+-----------------+-----------------+-----------------+-----------------+

|[1.01, 2.58)| ks.D=0.007 | ks.D=0.012 | ks.D=0.015 (*) | ks.D=0.012 | ks.D=0.005 |

| | p.05=0.051 0 | p.05=0.048 0 | p.05=0.049 0 | p.05=0.049 0 | p.05=0.049 00 |

| | p.01=0.010 | p.01=0.012 | p.01=0.012 | p.01=0.009 | p.01=0.011 0 |

+-------------+-----------------+-----------------+-----------------+-----------------+-----------------+

|[2.58,372.79]| ks.D=0.009 | ks.D=0.009 | ks.D=0.014 | ks.D=0.011 | ks.D=0.008 |

| | p.05=0.052 0 | p.05=0.049 0 | p.05=0.044 *,0 | p.05=0.051 0 | p.05=0.049 00 |

| | p.01=0.009 | p.01=0.009 | p.01=0.008 (*) | p.01=0.011 | p.01=0.009 0 |

+-------------+-----------------+-----------------+-----------------+-----------------+-----------------+

|ALL | ks.D=0.028 *** | ks.D=0.025 *** | ks.D=0.017 *** | ks.D=0.012 ** | ks.D=0.020 *** |

| | p.05=0.060***,+ | p.05=0.060***,+ | p.05=0.055 **,0 | p.05=0.056 **,0 | p.05=0.058***,+ |

| | p.01=0.014***,++| p.01=0.016***,++| p.01=0.014***,++| p.01=0.013***,+ | p.01=0.014***,++|

+-------------+-----------------+-----------------+-----------------+-----------------+-----------------+

$p_{sp}$, $t=mean$

+-------------+-----------------+-----------------+-----------------+-----------------+-----------------+

| | [ 20, 80) | [ 80, 300) | [300, 600) | [600,1000] | ALL |

+-------------+-----------------+-----------------+-----------------+-----------------+-----------------+

|[0.00, 0.40)| ks.D=0.010 | ks.D=0.010 | ks.D=0.010 | ks.D=0.018 (*) | ks.D=0.008 (*) |

| | p.05=0.052 0 | p.05=0.052 0 | p.05=0.048 0 | p.05=0.054 0 | p.05=0.051 00 |

| | p.01=0.012 | p.01=0.008 (*) | p.01=0.009 | p.01=0.010 | p.01=0.010 0 |

+-------------+-----------------+-----------------+-----------------+-----------------+-----------------+

|[0.40, 1.01)| ks.D=0.014 | ks.D=0.011 | ks.D=0.011 | ks.D=0.015 | ks.D=0.005 |

| | p.05=0.050 0 | p.05=0.051 0 | p.05=0.049 0 | p.05=0.045 0 | p.05=0.049 00 |

| | p.01=0.010 | p.01=0.009 | p.01=0.012 (*) | p.01=0.009 | p.01=0.010 0 |

+-------------+-----------------+-----------------+-----------------+-----------------+-----------------+

|[1.01, 2.58)| ks.D=0.015 | ks.D=0.008 | ks.D=0.008 | ks.D=0.014 | ks.D=0.004 |

| | p.05=0.056 * | p.05=0.044 *,0 | p.05=0.052 0 | p.05=0.052 0 | p.05=0.051 00 |

| | p.01=0.012 | p.01=0.011 | p.01=0.012 (*) | p.01=0.011 | p.01=0.012 * |

+-------------+-----------------+-----------------+-----------------+-----------------+-----------------+

|[2.58,372.79]| ks.D=0.008 | ks.D=0.008 | ks.D=0.021 ** | ks.D=0.012 | ks.D=0.006 |

| | p.05=0.048 0 | p.05=0.048 0 | p.05=0.050 0 | p.05=0.051 0 | p.05=0.049 00 |

| | p.01=0.011 | p.01=0.012 | p.01=0.009 | p.01=0.009 | p.01=0.010 0 |

+-------------+-----------------+-----------------+-----------------+-----------------+-----------------+

|ALL | ks.D=0.004 | ks.D=0.004 | ks.D=0.007 | ks.D=0.006 | ks.D=0.004 * |

| | p.05=0.052 00 | p.05=0.049 00 | p.05=0.050 00 | p.05=0.051 00 | p.05=0.050 00 |

| | p.01=0.011 (*) | p.01=0.010 0 | p.01=0.011 0 | p.01=0.010 0 | p.01=0.010 00 |

+-------------+-----------------+-----------------+-----------------+-----------------+-----------------+

$p_{sp}$, $t=var$

+-------------+-----------------+-----------------+-----------------+-----------------+-----------------+

| | [ 20, 80) | [ 80, 300) | [300, 600) | [600,1000] | ALL |

+-------------+-----------------+-----------------+-----------------+-----------------+-----------------+

|[0.00, 0.40)| ks.D=0.107 *** | ks.D=0.089 *** | ks.D=0.061 *** | ks.D=0.041 *** | ks.D=0.073 *** |

| | p.05=0.169***,++| p.05=0.157***,++| p.05=0.127***,++| p.05=0.101***,++| p.05=0.140***,++|

| | p.01=0.076***,++| p.01=0.079***,++| p.01=0.060***,++| p.01=0.040***,++| p.01=0.065***,++|

+-------------+-----------------+-----------------+-----------------+-----------------+-----------------+

|[0.40, 1.01)| ks.D=0.024 ** | ks.D=0.015 | ks.D=0.010 | ks.D=0.014 | ks.D=0.009 * |

| | p.05=0.059 ** | p.05=0.056 * | p.05=0.052 0 | p.05=0.052 0 | p.05=0.055 **,0 |

| | p.01=0.015***,++| p.01=0.012 | p.01=0.010 | p.01=0.009 | p.01=0.012 * |

+-------------+-----------------+-----------------+-----------------+-----------------+-----------------+

|[1.01, 2.58)| ks.D=0.010 | ks.D=0.007 | ks.D=0.013 | ks.D=0.018 (*) | ks.D=0.004 |

| | p.05=0.050 0 | p.05=0.047 0 | p.05=0.047 0 | p.05=0.049 0 | p.05=0.048 00 |

| | p.01=0.009 | p.01=0.009 | p.01=0.010 | p.01=0.009 | p.01=0.009 0 |

+-------------+-----------------+-----------------+-----------------+-----------------+-----------------+

|[2.58,372.79]| ks.D=0.018 * | ks.D=0.016 (*) | ks.D=0.007 | ks.D=0.012 | ks.D=0.004 |

| | p.05=0.048 0 | p.05=0.047 0 | p.05=0.049 0 | p.05=0.051 0 | p.05=0.049 00 |

| | p.01=0.009 | p.01=0.010 | p.01=0.009 | p.01=0.008 (*) | p.01=0.009 (*) |

+-------------+-----------------+-----------------+-----------------+-----------------+-----------------+

|ALL | ks.D=0.028 *** | ks.D=0.025 *** | ks.D=0.019 *** | ks.D=0.010 * | ks.D=0.021 *** |

| | p.05=0.082***,++| p.05=0.077***,++| p.05=0.069***,++| p.05=0.063***,++| p.05=0.073***,++|

| | p.01=0.027***,++| p.01=0.028***,++| p.01=0.022***,++| p.01=0.017***,++| p.01=0.024***,++|

+-------------+-----------------+-----------------+-----------------+-----------------+-----------------+

$p_{sp}$, $t=p_{0}$

+-------------+-----------------+-----------------+-----------------+-----------------+-----------------+

| | [ 20, 80) | [ 80, 300) | [300, 600) | [600,1000] | ALL |

+-------------+-----------------+-----------------+-----------------+-----------------+-----------------+

|[0.00, 0.40)| ks.D=0.019 * | ks.D=0.018 * | ks.D=0.018 * | ks.D=0.020 * | ks.D=0.016 *** |

| | p.05=0.066***,++| p.05=0.065***,++| p.05=0.065***,++| p.05=0.060 **,+ | p.05=0.064***,++|

| | p.01=0.015***,++| p.01=0.017***,++| p.01=0.016***,++| p.01=0.015**,++ | p.01=0.016***,++|

+-------------+-----------------+-----------------+-----------------+-----------------+-----------------+

|[0.40, 1.01)| ks.D=0.009 | ks.D=0.010 | ks.D=0.009 | ks.D=0.009 | ks.D=0.004 |

| | p.05=0.049 0 | p.05=0.052 0 | p.05=0.050 0 | p.05=0.045(*),0 | p.05=0.049 00 |

| | p.01=0.008 | p.01=0.009 | p.01=0.009 | p.01=0.007 * | p.01=0.009 * |

+-------------+-----------------+-----------------+-----------------+-----------------+-----------------+

|[1.01, 2.58)| ks.D=0.012 | ks.D=0.009 | ks.D=0.010 | ks.D=0.013 | ks.D=0.004 |

| | p.05=0.050 0 | p.05=0.049 0 | p.05=0.052 0 | p.05=0.050 0 | p.05=0.050 00 |

| | p.01=0.010 | p.01=0.010 | p.01=0.011 | p.01=0.009 | p.01=0.010 0 |

+-------------+-----------------+-----------------+-----------------+-----------------+-----------------+

|[2.58,372.79]| ks.D=0.009 | ks.D=0.008 | ks.D=0.008 | ks.D=0.009 | ks.D=0.004 |

| | p.05=0.050 0 | p.05=0.050 0 | p.05=0.045(*),0 | p.05=0.047 0 | p.05=0.048 00 |

| | p.01=0.010 | p.01=0.011 | p.01=0.009 | p.01=0.010 | p.01=0.010 0 |

+-------------+-----------------+-----------------+-----------------+-----------------+-----------------+

|ALL | ks.D=0.007 | ks.D=0.008 (*) | ks.D=0.009 * | ks.D=0.008 (*) | ks.D=0.005 * |

| | p.05=0.054 **,0 | p.05=0.054 **,0 | p.05=0.053 *,0 | p.05=0.050 00 | p.05=0.053***,00|

| | p.01=0.011 | p.01=0.012 ** | p.01=0.011 * | p.01=0.010 0 | p.01=0.011 **,0 |

+-------------+-----------------+-----------------+-----------------+-----------------+-----------------+

$p_{sp}$, $d=meanc$

+-------------+-----------------+-----------------+-----------------+-----------------+-----------------+

| | [ 20, 80) | [ 80, 300) | [300, 600) | [600,1000] | ALL |

+-------------+-----------------+-----------------+-----------------+-----------------+-----------------+

|[0.00, 0.40)| ks.D=0.010 | ks.D=0.010 | ks.D=0.011 | ks.D=0.017 (*) | ks.D=0.008 |

| | p.05=0.053 0 | p.05=0.053 0 | p.05=0.049 0 | p.05=0.053 0 | p.05=0.052 00 |

| | p.01=0.012 (*) | p.01=0.008 (*) | p.01=0.009 | p.01=0.009 | p.01=0.010 0 |

+-------------+-----------------+-----------------+-----------------+-----------------+-----------------+

|[0.40, 1.01)| ks.D=0.015 (*) | ks.D=0.011 | ks.D=0.011 | ks.D=0.015 | ks.D=0.006 |

| | p.05=0.049 0 | p.05=0.051 0 | p.05=0.051 0 | p.05=0.045 0 | p.05=0.049 00 |

| | p.01=0.010 | p.01=0.009 | p.01=0.011 | p.01=0.009 | p.01=0.010 0 |

+-------------+-----------------+-----------------+-----------------+-----------------+-----------------+

|[1.01, 2.58)| ks.D=0.015 | ks.D=0.010 | ks.D=0.008 | ks.D=0.014 | ks.D=0.003 |

| | p.05=0.055 (*) | p.05=0.045 0 | p.05=0.053 0 | p.05=0.054 0 | p.05=0.052 00 |

| | p.01=0.011 | p.01=0.010 | p.01=0.012 | p.01=0.009 | p.01=0.011 0 |

+-------------+-----------------+-----------------+-----------------+-----------------+-----------------+

|[2.58,372.79]| ks.D=0.009 | ks.D=0.008 | ks.D=0.023 ** | ks.D=0.012 | ks.D=0.007 |

| | p.05=0.048 0 | p.05=0.050 0 | p.05=0.050 0 | p.05=0.051 0 | p.05=0.050 00 |

| | p.01=0.011 | p.01=0.012 | p.01=0.008 | p.01=0.009 | p.01=0.010 0 |

+-------------+-----------------+-----------------+-----------------+-----------------+-----------------+

|ALL | ks.D=0.004 | ks.D=0.005 | ks.D=0.007 | ks.D=0.007 | ks.D=0.005 * |

| | p.05=0.051 00 | p.05=0.050 00 | p.05=0.051 00 | p.05=0.051 00 | p.05=0.051 00 |

| | p.01=0.011 0 | p.01=0.009 0 | p.01=0.010 0 | p.01=0.009 0 | p.01=0.010 00 |

+-------------+-----------------+-----------------+-----------------+-----------------+-----------------+

$p_{sp}$, $d=varc$

+-------------+-----------------+-----------------+-----------------+-----------------+-----------------+

| | [ 20, 80) | [ 80, 300) | [300, 600) | [600,1000] | ALL |

+-------------+-----------------+-----------------+-----------------+-----------------+-----------------+

|[0.00, 0.40)| ks.D=0.107 *** | ks.D=0.088 *** | ks.D=0.061 *** | ks.D=0.041 *** | ks.D=0.073 *** |

| | p.05=0.168***,++| p.05=0.157***,++| p.05=0.125***,++| p.05=0.101***,++| p.05=0.139***,++|

| | p.01=0.077***,++| p.01=0.079***,++| p.01=0.058***,++| p.01=0.041***,++| p.01=0.065***,++|

+-------------+-----------------+-----------------+-----------------+-----------------+-----------------+

|[0.40, 1.01)| ks.D=0.024 ** | ks.D=0.014 | ks.D=0.011 | ks.D=0.013 | ks.D=0.010 * |

| | p.05=0.057 ** | p.05=0.056 * | p.05=0.050 0 | p.05=0.052 0 | p.05=0.054 **,0 |

| | p.01=0.016***,++| p.01=0.012 | p.01=0.009 | p.01=0.009 | p.01=0.012 * |

+-------------+-----------------+-----------------+-----------------+-----------------+-----------------+

|[1.01, 2.58)| ks.D=0.010 | ks.D=0.008 | ks.D=0.012 | ks.D=0.019 (*) | ks.D=0.004 |

| | p.05=0.048 0 | p.05=0.048 0 | p.05=0.049 0 | p.05=0.049 0 | p.05=0.048 00 |

| | p.01=0.009 | p.01=0.010 | p.01=0.010 | p.01=0.010 | p.01=0.010 0 |

+-------------+-----------------+-----------------+-----------------+-----------------+-----------------+

|[2.58,372.79]| ks.D=0.018 * | ks.D=0.017 (*) | ks.D=0.008 | ks.D=0.011 | ks.D=0.004 |

| | p.05=0.048 0 | p.05=0.047 0 | p.05=0.050 0 | p.05=0.052 0 | p.05=0.049 00 |

| | p.01=0.009 | p.01=0.009 | p.01=0.009 | p.01=0.009 | p.01=0.009 0 |

+-------------+-----------------+-----------------+-----------------+-----------------+-----------------+

|ALL | ks.D=0.028 *** | ks.D=0.024 *** | ks.D=0.020 *** | ks.D=0.011 * | ks.D=0.021 *** |

| | p.05=0.080***,++| p.05=0.077***,++| p.05=0.069***,++| p.05=0.063***,++| p.05=0.073***,++|

| | p.01=0.028***,++| p.01=0.028***,++| p.01=0.022***,++| p.01=0.017***,++| p.01=0.024***,++|

+-------------+-----------------+-----------------+-----------------+-----------------+-----------------+

$p_{sp}$, $d=LL$

+-------------+-----------------+-----------------+-----------------+-----------------+-----------------+

| | [ 20, 80) | [ 80, 300) | [300, 600) | [600,1000] | ALL |

+-------------+-----------------+-----------------+-----------------+-----------------+-----------------+

|[0.00, 0.40)| ks.D=0.107 *** | ks.D=0.087 *** | ks.D=0.061 *** | ks.D=0.040 *** | ks.D=0.072 *** |

| | p.05=0.163***,++| p.05=0.157***,++| p.05=0.126***,++| p.05=0.101***,++| p.05=0.138***,++|

| | p.01=0.072***,++| p.01=0.079***,++| p.01=0.057***,++| p.01=0.041***,++| p.01=0.063***,++|

+-------------+-----------------+-----------------+-----------------+-----------------+-----------------+

|[0.40, 1.01)| ks.D=0.024 ** | ks.D=0.012 | ks.D=0.010 | ks.D=0.015 | ks.D=0.010 * |

| | p.05=0.058 ** | p.05=0.057 * | p.05=0.051 0 | p.05=0.052 0 | p.05=0.054 **,0 |

| | p.01=0.016***,++| p.01=0.011 | p.01=0.009 | p.01=0.010 | p.01=0.011 * |

+-------------+-----------------+-----------------+-----------------+-----------------+-----------------+

|[1.01, 2.58)| ks.D=0.010 | ks.D=0.007 | ks.D=0.011 | ks.D=0.015 | ks.D=0.003 |

| | p.05=0.046 0 | p.05=0.047 0 | p.05=0.047 0 | p.05=0.050 0 | p.05=0.048(*),00|

| | p.01=0.009 | p.01=0.010 | p.01=0.011 | p.01=0.009 | p.01=0.010 0 |

+-------------+-----------------+-----------------+-----------------+-----------------+-----------------+

|[2.58,372.79]| ks.D=0.020 * | ks.D=0.014 | ks.D=0.008 | ks.D=0.011 | ks.D=0.003 |

| | p.05=0.049 0 | p.05=0.048 0 | p.05=0.049 0 | p.05=0.052 0 | p.05=0.050 00 |

| | p.01=0.008 | p.01=0.009 | p.01=0.009 | p.01=0.008 | p.01=0.009 * |

+-------------+-----------------+-----------------+-----------------+-----------------+-----------------+

|ALL | ks.D=0.028 *** | ks.D=0.025 *** | ks.D=0.019 *** | ks.D=0.010 * | ks.D=0.020 *** |

| | p.05=0.079***,++| p.05=0.077***,++| p.05=0.068***,++| p.05=0.064***,++| p.05=0.072***,++|

| | p.01=0.026***,++| p.01=0.027***,++| p.01=0.022***,++| p.01=0.017***,++| p.01=0.023***,++|

+-------------+-----------------+-----------------+-----------------+-----------------+-----------------+

## Bernoulli models

$p_{nsp}$, $t=mean$

+-------------+-----------------+-----------------+-----------------+-----------------+-----------------+

| | [ 20, 80) | [ 80, 300) | [300, 600) | [600,1000] | ALL |

+-------------+-----------------+-----------------+-----------------+-----------------+-----------------+

|[0.00, 0.40)| ks.D=0.055 *** | ks.D=0.039 *** | ks.D=0.034 *** | ks.D=0.023 ** | ks.D=0.033 *** |

| | p.05=0.103***,++| p.05=0.102***,++| p.05=0.081***,++| p.05=0.075***,++| p.05=0.091***,++|

| | p.01=0.036***,++| p.01=0.036***,++| p.01=0.027***,++| p.01=0.023***,++| p.01=0.031***,++|

+-------------+-----------------+-----------------+-----------------+-----------------+-----------------+

|[0.40, 1.01)| ks.D=0.015 | ks.D=0.017 (*) | ks.D=0.010 | ks.D=0.014 | ks.D=0.004 |

| | p.05=0.053 0 | p.05=0.053 0 | p.05=0.046 0 | p.05=0.051 0 | p.05=0.051 00 |

| | p.01=0.011 | p.01=0.012 (*) | p.01=0.009 | p.01=0.010 | p.01=0.010 0 |

+-------------+-----------------+-----------------+-----------------+-----------------+-----------------+

|[1.01, 2.56)| ks.D=0.010 | ks.D=0.013 | ks.D=0.007 | ks.D=0.013 | ks.D=0.005 |

| | p.05=0.046 0 | p.05=0.051 0 | p.05=0.055(*),0 | p.05=0.049 0 | p.05=0.050 00 |

| | p.01=0.009 | p.01=0.011 | p.01=0.010 | p.01=0.009 | p.01=0.010 0 |

+-------------+-----------------+-----------------+-----------------+-----------------+-----------------+

|[2.56,415.82]| ks.D=0.011 | ks.D=0.019 * | ks.D=0.006 | ks.D=0.012 | ks.D=0.005 |

| | p.05=0.056 * | p.05=0.052 0 | p.05=0.051 0 | p.05=0.057 * | p.05=0.054 **,0 |

| | p.01=0.012 | p.01=0.010 | p.01=0.012 | p.01=0.009 | p.01=0.011 0 |

+-------------+-----------------+-----------------+-----------------+-----------------+-----------------+

|ALL | ks.D=0.013 ** | ks.D=0.010 * | ks.D=0.010 ** | ks.D=0.006 | ks.D=0.008 *** |

| | p.05=0.065***,++| p.05=0.065***,++| p.05=0.058***,+ | p.05=0.058***,+ | p.05=0.062***,++|

| | p.01=0.017***,++| p.01=0.018***,++| p.01=0.014***,++| p.01=0.013 **,+ | p.01=0.016***,++|

+-------------+-----------------+-----------------+-----------------+-----------------+-----------------+

$p_{nsp}$, $t=var$

+-------------+-----------------+-----------------+-----------------+-----------------+-----------------+

| | [ 20, 80) | [ 80, 300) | [300, 600) | [600,1000] | ALL |

+-------------+-----------------+-----------------+-----------------+-----------------+-----------------+

|[0.00, 0.40)| ks.D=0.066 *** | ks.D=0.046 *** | ks.D=0.048 *** | ks.D=0.035 *** | ks.D=0.046 *** |

| | p.05=0.063***,+ | p.05=0.062***,+ | p.05=0.058 ** | p.05=0.063***,+ | p.05=0.061***,+ |

| | p.01=0.015***,++| p.01=0.016***,++| p.01=0.013 *,+ | p.01=0.017***,++| p.01=0.015***,++|

+-------------+-----------------+-----------------+-----------------+-----------------+-----------------+

|[0.40, 1.01)| ks.D=0.016 (*) | ks.D=0.018 * | ks.D=0.007 | ks.D=0.013 | ks.D=0.011 ** |

| | p.05=0.050 0 | p.05=0.050 0 | p.05=0.046 0 | p.05=0.052 0 | p.05=0.049 00 |

| | p.01=0.008 (*) | p.01=0.010 | p.01=0.011 | p.01=0.010 | p.01=0.010 0 |

+-------------+-----------------+-----------------+-----------------+-----------------+-----------------+

|[1.01, 2.56)| ks.D=0.006 | ks.D=0.023 ** | ks.D=0.008 | ks.D=0.010 | ks.D=0.005 |

| | p.05=0.049 0 | p.05=0.044 *,0 | p.05=0.046(*),0 | p.05=0.053 0 | p.05=0.048(*),00|

| | p.01=0.011 | p.01=0.007 * | p.01=0.010 | p.01=0.011 | p.01=0.010 0 |

+-------------+-----------------+-----------------+-----------------+-----------------+-----------------+

|[2.56,415.82]| ks.D=0.029 *** | ks.D=0.020 * | ks.D=0.019 * | ks.D=0.016 | ks.D=0.018 *** |

| | p.05=0.051 0 | p.05=0.050 0 | p.05=0.052 0 | p.05=0.048 0 | p.05=0.051 00 |

| | p.01=0.011 | p.01=0.009 | p.01=0.010 | p.01=0.009 | p.01=0.010 0 |

+-------------+-----------------+-----------------+-----------------+-----------------+-----------------+

|ALL | ks.D=0.015 *** | ks.D=0.009 * | ks.D=0.011 ** | ks.D=0.010 * | ks.D=0.010 *** |

| | p.05=0.053 *,0 | p.05=0.052 00 | p.05=0.051 00 | p.05=0.054 **,0 | p.05=0.052**,00 |

| | p.01=0.011 * | p.01=0.011 0 | p.01=0.011 (*) | p.01=0.011 * | p.01=0.011 **,0 |

+-------------+-----------------+-----------------+-----------------+-----------------+-----------------+

$p_{nsp}$, $t=skew$

+-------------+-----------------+-----------------+-----------------+-----------------+-----------------+

| | [ 20, 80) | [ 80, 300) | [300, 600) | [600,1000] | ALL |

+-------------+-----------------+-----------------+-----------------+-----------------+-----------------+

|[0.00, 0.40)| ks.D=0.019 * | ks.D=0.013 | ks.D=0.014 | ks.D=0.009 | ks.D=0.010 ** |

| | p.05=0.062***,+ | p.05=0.058 ** | p.05=0.058 ** | p.05=0.052 0 | p.05=0.058***,+ |

| | p.01=0.015**,++ | p.01=0.012 (*) | p.01=0.015**,++ | p.01=0.010 | p.01=0.013***,++|

+-------------+-----------------+-----------------+-----------------+-----------------+-----------------+

|[0.40, 1.01)| ks.D=0.007 | ks.D=0.014 | ks.D=0.007 | ks.D=0.014 | ks.D=0.007 |

| | p.05=0.051 0 | p.05=0.047 0 | p.05=0.053 0 | p.05=0.056 (*) | p.05=0.052 00 |

| | p.01=0.010 | p.01=0.010 | p.01=0.010 | p.01=0.009 | p.01=0.010 0 |

+-------------+-----------------+-----------------+-----------------+-----------------+-----------------+

|[1.01, 2.56)| ks.D=0.012 | ks.D=0.008 | ks.D=0.008 | ks.D=0.010 | ks.D=0.006 |

| | p.05=0.052 0 | p.05=0.053 0 | p.05=0.051 0 | p.05=0.048 0 | p.05=0.051 00 |

| | p.01=0.012 | p.01=0.011 | p.01=0.012 (*) | p.01=0.009 | p.01=0.011 |

+-------------+-----------------+-----------------+-----------------+-----------------+-----------------+

|[2.56,415.82]| ks.D=0.009 | ks.D=0.017 (*) | ks.D=0.007 | ks.D=0.015 | ks.D=0.005 |

| | p.05=0.048 0 | p.05=0.044 * | p.05=0.051 0 | p.05=0.056 * | p.05=0.050 00 |

| | p.01=0.009 | p.01=0.010 | p.01=0.010 | p.01=0.014 **,+ | p.01=0.011 0 |

+-------------+-----------------+-----------------+-----------------+-----------------+-----------------+

|ALL | ks.D=0.008 (*) | ks.D=0.007 | ks.D=0.004 | ks.D=0.006 | ks.D=0.005 ** |

| | p.05=0.053 *,0 | p.05=0.050 00 | p.05=0.053 *,0 | p.05=0.053 *,0 | p.05=0.053**,00 |

| | p.01=0.011 * | p.01=0.011 | p.01=0.012 ** | p.01=0.010 0 | p.01=0.011***,0 |

+-------------+-----------------+-----------------+-----------------+-----------------+-----------------+

$p_{nsp}$, $t=kurt$

+-------------+-----------------+-----------------+-----------------+-----------------+-----------------+

| | [ 20, 80) | [ 80, 300) | [300, 600) | [600,1000] | ALL |

+-------------+-----------------+-----------------+-----------------+-----------------+-----------------+

|[0.00, 0.40)| ks.D=0.049 *** | ks.D=0.054 *** | ks.D=0.034 *** | ks.D=0.023 ** | ks.D=0.039 *** |

| | p.05=0.053 0 | p.05=0.056 * | p.05=0.050 0 | p.05=0.052 0 | p.05=0.053 *,0 |

| | p.01=0.011 | p.01=0.012 | p.01=0.012 | p.01=0.012 | p.01=0.012 * |

+-------------+-----------------+-----------------+-----------------+-----------------+-----------------+

|[0.40, 1.01)| ks.D=0.012 | ks.D=0.018 * | ks.D=0.010 | ks.D=0.014 | ks.D=0.010 * |

| | p.05=0.052 0 | p.05=0.055 0 | p.05=0.050 0 | p.05=0.049 0 | p.05=0.051 00 |

| | p.01=0.011 | p.01=0.010 | p.01=0.011 | p.01=0.009 | p.01=0.010 0 |

+-------------+-----------------+-----------------+-----------------+-----------------+-----------------+

|[1.01, 2.56)| ks.D=0.009 | ks.D=0.010 | ks.D=0.007 | ks.D=0.011 | ks.D=0.006 |

| | p.05=0.056 * | p.05=0.046 0 | p.05=0.047 0 | p.05=0.046 0 | p.05=0.049 00 |

| | p.01=0.013 * | p.01=0.009 | p.01=0.009 | p.01=0.009 | p.01=0.010 0 |

+-------------+-----------------+-----------------+-----------------+-----------------+-----------------+

|[2.56,415.82]| ks.D=0.011 | ks.D=0.023 ** | ks.D=0.007 | ks.D=0.010 | ks.D=0.009 * |

| | p.05=0.051 0 | p.05=0.053 0 | p.05=0.050 0 | p.05=0.050 0 | p.05=0.051 00 |

| | p.01=0.010 | p.01=0.010 | p.01=0.010 | p.01=0.009 | p.01=0.010 0 |

+-------------+-----------------+-----------------+-----------------+-----------------+-----------------+

|ALL | ks.D=0.013 ** | ks.D=0.015 *** | ks.D=0.012 ** | ks.D=0.006 | ks.D=0.011 *** |

| | p.05=0.053 *,0 | p.05=0.052(*),00| p.05=0.049 00 | p.05=0.049 00 | p.05=0.051 00 |

| | p.01=0.011 | p.01=0.010 0 | p.01=0.010 0 | p.01=0.010 0 | p.01=0.010 00 |

+-------------+-----------------+-----------------+-----------------+-----------------+-----------------+

$p_{nsp}$, $t=Z_{a}$

+-------------+-----------------+-----------------+-----------------+-----------------+-----------------+

| | [ 20, 80) | [ 80, 300) | [300, 600) | [600,1000] | ALL |

+-------------+-----------------+-----------------+-----------------+-----------------+-----------------+

|[0.00, 0.40)| ks.D=0.089 *** | ks.D=0.073 *** | ks.D=0.045 *** | ks.D=0.028 ** | ks.D=0.057 *** |

| | p.05=0.081***,++| p.05=0.081***,++| p.05=0.068***,++| p.05=0.065***,+ | p.05=0.074***,++|

| | p.01=0.025***,++| p.01=0.028***,++| p.01=0.016***,++| p.01=0.018***,++| p.01=0.022***,++|

+-------------+-----------------+-----------------+-----------------+-----------------+-----------------+

|[0.40, 1.01)| ks.D=0.009 | ks.D=0.011 | ks.D=0.007 | ks.D=0.009 | ks.D=0.005 |

| | p.05=0.052 0 | p.05=0.052 0 | p.05=0.049 0 | p.05=0.047 0 | p.05=0.050 00 |

| | p.01=0.011 | p.01=0.010 | p.01=0.009 | p.01=0.009 | p.01=0.010 0 |

+-------------+-----------------+-----------------+-----------------+-----------------+-----------------+

|[1.01, 2.56)| ks.D=0.012 | ks.D=0.019 * | ks.D=0.010 | ks.D=0.013 | ks.D=0.006 |

| | p.05=0.051 0 | p.05=0.051 0 | p.05=0.053 0 | p.05=0.048 0 | p.05=0.051 00 |

| | p.01=0.010 | p.01=0.012 (*) | p.01=0.011 | p.01=0.010 | p.01=0.011 0 |

+-------------+-----------------+-----------------+-----------------+-----------------+-----------------+

|[2.56,415.82]| ks.D=0.008 | ks.D=0.010 | ks.D=0.011 | ks.D=0.011 | ks.D=0.004 |

| | p.05=0.050 0 | p.05=0.046 0 | p.05=0.048 0 | p.05=0.050 0 | p.05=0.049 00 |

| | p.01=0.012 | p.01=0.012 (*) | p.01=0.010 | p.01=0.012 (*) | p.01=0.012 ** |

+-------------+-----------------+-----------------+-----------------+-----------------+-----------------+

|ALL | ks.D=0.025 *** | ks.D=0.016 *** | ks.D=0.012 ** | ks.D=0.011 * | ks.D=0.016 *** |

| | p.05=0.059***,+ | p.05=0.058***,+ | p.05=0.055 **,0 | p.05=0.053(*),0 | p.05=0.056***,0 |

| | p.01=0.015***,++| p.01=0.016***,++| p.01=0.012 ** | p.01=0.012 **,+ | p.01=0.014***,++|

+-------------+-----------------+-----------------+-----------------+-----------------+-----------------+

$p_{sp}$, $t=mean$

+-------------+-----------------+-----------------+-----------------+-----------------+-----------------+

| | [ 20, 80) | [ 80, 300) | [300, 600) | [600,1000] | ALL |

+-------------+-----------------+-----------------+-----------------+-----------------+-----------------+

|[0.00, 0.40)| ks.D=0.075*** | ks.D=0.06*** | ks.D=0.05*** | ks.D=0.031*** | ks.D=0.049*** |

| | p.05=0.138***,++| p.05=0.131***,++| p.05=0.103***,++| p.05=0.089***,++| p.05=0.116***,++|

| | p.01=0.056***,++| p.01=0.055***,++| p.01=0.042***,++| p.01=0.034***,++| p.01=0.047***,++|

+-------------+-----------------+-----------------+-----------------+-----------------+-----------------+

|[0.40, 1.01)| ks.D=0.019 * | ks.D=0.011 | ks.D=0.008 | ks.D=0.017(*) | ks.D=0.008(*) |

| | p.05=0.056 * | p.05=0.050 0 | p.05=0.050 0 | p.05=0.052 0 | p.05=0.052 00 |

| | p.01=0.012 | p.01=0.010 | p.01=0.009 | p.01=0.010 | p.01=0.010 0 |

+-------------+-----------------+-----------------+-----------------+-----------------+-----------------+

|[1.01, 2.56)| ks.D=0.012 | ks.D=0.008 | ks.D=0.012 | ks.D=0.014 | ks.D=0.004 |

| | p.05=0.044 *,0 | p.05=0.047 0 | p.05=0.057 ** | p.05=0.054 0 | p.05=0.051 00 |

| | p.01=0.011 | p.01=0.010 | p.01=0.010 | p.01=0.010 | p.01=0.010 0 |

+-------------+-----------------+-----------------+-----------------+-----------------+-----------------+

|[2.56,415.82]| ks.D=0.011 | ks.D=0.016(*) | ks.D=0.006 | ks.D=0.012 | ks.D=0.006 |

| | p.05=0.045(*),0 | p.05=0.048 0 | p.05=0.049 0 | p.05=0.053 0 | p.05=0.049 00 |

| | p.01=0.008 | p.01=0.009 | p.01=0.010 | p.01=0.011 | p.01=0.010 0 |

+-------------+-----------------+-----------------+-----------------+-----------------+-----------------+

|ALL | ks.D=0.017*** | ks.D=0.015*** | ks.D=0.015*** | ks.D=0.011** | ks.D=0.014*** |

| | p.05=0.071***,++| p.05=0.069***,++| p.05=0.065***,++| p.05=0.062***,+ | p.05=0.067***,++|

| | p.01=0.022***,++| p.01=0.022***,++| p.01=0.018***,++| p.01=0.016***,++| p.01=0.020***,++|

+-------------+-----------------+-----------------+-----------------+-----------------+-----------------+

$p_{sp}$, $t=var$

+-------------+-----------------+-----------------+-----------------+-----------------+-----------------+

| | [ 20, 80) | [ 80, 300) | [300, 600) | [600,1000] | ALL |

+-------------+-----------------+-----------------+-----------------+-----------------+-----------------+

|[0.00, 0.40)| ks.D=0.174 *** | ks.D=0.170 *** | ks.D=0.135 *** | ks.D=0.112 *** | ks.D=0.148 *** |

| | p.05=0.132***,++| p.05=0.131***,++| p.05=0.104***,++| p.05=0.091***,++| p.05=0.115***,++|

| | p.01=0.053***,++| p.01=0.057***,++| p.01=0.043***,++| p.01=0.034***,++| p.01=0.047***,++|

+-------------+-----------------+-----------------+-----------------+-----------------+-----------------+

|[0.40, 1.01)| ks.D=0.045 *** | ks.D=0.036 *** | ks.D=0.022 ** | ks.D=0.015 | ks.D=0.027 *** |

| | p.05=0.059 ** | p.05=0.051 0 | p.05=0.049 0 | p.05=0.053 0 | p.05=0.053 *,0 |

| | p.01=0.012 | p.01=0.011 | p.01=0.009 | p.01=0.010 | p.01=0.011 0 |

+-------------+-----------------+-----------------+-----------------+-----------------+-----------------+

|[1.01, 2.56)| ks.D=0.029 *** | ks.D=0.017 (*) | ks.D=0.014 | ks.D=0.024 ** | ks.D=0.015 *** |

| | p.05=0.047 0 | p.05=0.047 0 | p.05=0.056 * | p.05=0.053 0 | p.05=0.051 00 |

| | p.01=0.009 | p.01=0.010 | p.01=0.010 | p.01=0.011 | p.01=0.010 0 |

+-------------+-----------------+-----------------+-----------------+-----------------+-----------------+

|[2.56,415.82]| ks.D=0.074 *** | ks.D=0.045 *** | ks.D=0.031 *** | ks.D=0.027 ** | ks.D=0.043 *** |

| | p.05=0.047 0 | p.05=0.046 0 | p.05=0.051 0 | p.05=0.054 0 | p.05=0.049 00 |

| | p.01=0.008 | p.01=0.010 | p.01=0.010 | p.01=0.010 | p.01=0.009 0 |

+-------------+-----------------+-----------------+-----------------+-----------------+-----------------+

|ALL | ks.D=0.036 *** | ks.D=0.041 *** | ks.D=0.034 *** | ks.D=0.025 *** | ks.D=0.033 *** |

| | p.05=0.072***,++| p.05=0.069***,++| p.05=0.065***,++| p.05=0.063***,++| p.05=0.067***,++|

| | p.01=0.021***,++| p.01=0.022***,++| p.01=0.018***,++| p.01=0.016***,++| p.01=0.019***,++|

+-------------+-----------------+-----------------+-----------------+-----------------+-----------------+

$p_{sp}$, $t=p_{0}$

+-------------+-----------------+-----------------+-----------------+-----------------+-----------------+

| | [ 20, 80) | [ 80, 300) | [300, 600) | [600,1000] | ALL |

+-------------+-----------------+-----------------+-----------------+-----------------+-----------------+

|[0.00, 0.40)| ks.D=0.074 *** | ks.D=0.060 *** | ks.D=0.049 *** | ks.D=0.030 *** | ks.D=0.049 *** |

| | p.05=0.139***,++| p.05=0.129***,++| p.05=0.105***,++| p.05=0.090***,++| p.05=0.117***,++|

| | p.01=0.057***,++| p.01=0.056***,++| p.01=0.043***,++| p.01=0.034***,++| p.01=0.048***,++|

+-------------+-----------------+-----------------+-----------------+-----------------+-----------------+

|[0.40, 1.01)| ks.D=0.017 (*) | ks.D=0.011 | ks.D=0.009 | ks.D=0.015 | ks.D=0.006 |

| | p.05=0.060 **,+ | p.05=0.050 0 | p.05=0.050 0 | p.05=0.050 0 | p.05=0.053 *,0 |

| | p.01=0.012 | p.01=0.010 | p.01=0.008 | p.01=0.010 | p.01=0.010 0 |

+-------------+-----------------+-----------------+-----------------+-----------------+-----------------+

|[1.01, 2.56)| ks.D=0.014 | ks.D=0.008 | ks.D=0.012 | ks.D=0.014 | ks.D=0.004 |

| | p.05=0.044 *,0 | p.05=0.048 0 | p.05=0.058 ** | p.05=0.055 | p.05=0.051 00 |

| | p.01=0.011 | p.01=0.010 | p.01=0.010 | p.01=0.010 | p.01=0.010 0 |

+-------------+-----------------+-----------------+-----------------+-----------------+-----------------+

|[2.56,415.82]| ks.D=0.013 | ks.D=0.016 | ks.D=0.006 | ks.D=0.012 | ks.D=0.006 |

| | p.05=0.046(*),0 | p.05=0.047 0 | p.05=0.051 0 | p.05=0.054 0 | p.05=0.049 00 |

| | p.01=0.009 | p.01=0.009 | p.01=0.010 | p.01=0.010 | p.01=0.010 0 |

+-------------+-----------------+-----------------+-----------------+-----------------+-----------------+

|ALL | ks.D=0.017 *** | ks.D=0.015 *** | ks.D=0.015 *** | ks.D=0.011 * | ks.D=0.014 *** |

| | p.05=0.073***,++| p.05=0.069***,++| p.05=0.066***,++| p.05=0.062***,+ | p.05=0.068***,++|

| | p.01=0.022***,++| p.01=0.022***,++| p.01=0.018***,++| p.01=0.016***,++| p.01=0.020***,++|

+-------------+-----------------+-----------------+-----------------+-----------------+-----------------+

$p_{sp}$, $d=meanc$

+-------------+-----------------+-----------------+-----------------+-----------------+-----------------+

| | [ 20, 80) | [ 80, 300) | [300, 600) | [600,1000] | ALL |

+-------------+-----------------+-----------------+-----------------+-----------------+-----------------+

|[0.00, 0.40)| ks.D=0.075 *** | ks.D=0.060 *** | ks.D=0.050 *** | ks.D=0.031 *** | ks.D=0.049 *** |

| | p.05=0.138***,++| p.05=0.131***,++| p.05=0.103***,++| p.05=0.089***,++| p.05=0.116***,++|

| | p.01=0.056***,++| p.01=0.055***,++| p.01=0.042***,++| p.01=0.034***,++| p.01=0.047***,++|

+-------------+-----------------+-----------------+-----------------+-----------------+-----------------+

|[0.40, 1.01)| ks.D=0.019 * | ks.D=0.011 | ks.D=0.008 | ks.D=0.017 (*) | ks.D=0.008 (*) |

| | p.05=0.056 * | p.05=0.050 0 | p.05=0.050 0 | p.05=0.052 0 | p.05=0.052 00 |

| | p.01=0.012 | p.01=0.010 | p.01=0.009 | p.01=0.010 | p.01=0.010 0 |

+-------------+-----------------+-----------------+-----------------+-----------------+-----------------+

|[1.01, 2.56)| ks.D=0.012 | ks.D=0.008 | ks.D=0.012 | ks.D=0.014 | ks.D=0.004 |

| | p.05=0.044 *,0 | p.05=0.047 0 | p.05=0.057 * | p.05=0.054 0 | p.05=0.051 00 |

| | p.01=0.011 | p.01=0.010 | p.01=0.010 | p.01=0.010 | p.01=0.010 0 |

+-------------+-----------------+-----------------+-----------------+-----------------+-----------------+

|[2.56,415.82]| ks.D=0.011 | ks.D=0.016 (*) | ks.D=0.006 | ks.D=0.012 | ks.D=0.006 |

| | p.05=0.045(*),0 | p.05=0.048 0 | p.05=0.049 0 | p.05=0.053 0 | p.05=0.049 00 |

| | p.01=0.008 | p.01=0.009 | p.01=0.010 | p.01=0.011 | p.01=0.010 0 |

+-------------+-----------------+-----------------+-----------------+-----------------+-----------------+

|ALL | ks.D=0.017 *** | ks.D=0.015 *** | ks.D=0.015 *** | ks.D=0.011 ** | ks.D=0.014 *** |

| | p.05=0.071***,++| p.05=0.069***,++| p.05=0.065***,++| p.05=0.062***,+ | p.05=0.067***,++|

| | p.01=0.022***,++| p.01=0.022***,++| p.01=0.018***,++| p.01=0.016***,++| p.01=0.020***,++|

+-------------+-----------------+-----------------+-----------------+-----------------+-----------------+

$p_{sp}$, $d=varc$

+-------------+-----------------+-----------------+-----------------+-----------------+-----------------+

| | [ 20, 80) | [ 80, 300) | [300, 600) | [600,1000] | ALL |

+-------------+-----------------+-----------------+-----------------+-----------------+-----------------+

|[0.00, 0.40)| ks.D=0.174 *** | ks.D=0.170 *** | ks.D=0.135 *** | ks.D=0.112 *** | ks.D=0.148 *** |

| | p.05=0.132***,++| p.05=0.131***,++| p.05=0.104***,++| p.05=0.091***,++| p.05=0.115***,++|

| | p.01=0.053***,++| p.01=0.057***,++| p.01=0.043***,++| p.01=0.034***,++| p.01=0.047***,++|

+-------------+-----------------+-----------------+-----------------+-----------------+-----------------+

|[0.40, 1.01)| ks.D=0.045 *** | ks.D=0.036 *** | ks.D=0.022 ** | ks.D=0.015 | ks.D=0.027 *** |

| | p.05=0.059 ** | p.05=0.051 0 | p.05=0.049 0 | p.05=0.053 0 | p.05=0.053(*),00|

| | p.01=0.012 | p.01=0.011 | p.01=0.009 | p.01=0.010 | p.01=0.011 0 |

+-------------+-----------------+-----------------+-----------------+-----------------+-----------------+

|[1.01, 2.56)| ks.D=0.029 *** | ks.D=0.017 (*) | ks.D=0.014 | ks.D=0.024 ** | ks.D=0.015 *** |

| | p.05=0.047 0 | p.05=0.047 0 | p.05=0.056 * | p.05=0.053 0 | p.05=0.051 00 |

| | p.01=0.010 | p.01=0.010 | p.01=0.010 | p.01=0.011 | p.01=0.010 0 |

+-------------+-----------------+-----------------+-----------------+-----------------+-----------------+

|[2.56,415.82]| ks.D=0.074 *** | ks.D=0.045 *** | ks.D=0.031 *** | ks.D=0.027 ** | ks.D=0.043 *** |

| | p.05=0.047 0 | p.05=0.046 0 | p.05=0.051 0 | p.05=0.054 0 | p.05=0.049 00 |

| | p.01=0.008 | p.01=0.010 | p.01=0.010 | p.01=0.010 | p.01=0.009 0 |

+-------------+-----------------+-----------------+-----------------+-----------------+-----------------+

|ALL | ks.D=0.036 *** | ks.D=0.041 *** | ks.D=0.034 *** | ks.D=0.025 *** | ks.D=0.033 *** |

| | p.05=0.072***,++| p.05=0.069***,++| p.05=0.065***,++| p.05=0.063***,++| p.05=0.067***,++|

| | p.01=0.021***,++| p.01=0.022***,++| p.01=0.018***,++| p.01=0.016***,++| p.01=0.019***,++|

+-------------+-----------------+-----------------+-----------------+-----------------+-----------------+

$p_{sp}$, $d=LL$

+-------------+-----------------+-----------------+-----------------+-----------------+-----------------+

| | [ 20, 80) | [ 80, 300) | [300, 600) | [600,1000] | ALL |

+-------------+-----------------+-----------------+-----------------+-----------------+-----------------+

|[0.00, 0.40)| ks.D=0.163 *** | ks.D=0.166 *** | ks.D=0.134 *** | ks.D=0.111 *** | ks.D=0.142 *** |

| | p.05=0.139***,++| p.05=0.129***,++| p.05=0.104***,++| p.05=0.089***,++| p.05=0.116***,++|

| | p.01=0.056***,++| p.01=0.054***,++| p.01=0.042***,++| p.01=0.034***,++| p.01=0.047***,++|

+-------------+-----------------+-----------------+-----------------+-----------------+-----------------+

|[0.40, 1.01)| ks.D=0.040 *** | ks.D=0.037 *** | ks.D=0.026 ** | ks.D=0.014 | ks.D=0.028 *** |

| | p.05=0.058 ** | p.05=0.050 0 | p.05=0.050 0 | p.05=0.051 0 | p.05=0.053(*),00|

| | p.01=0.013 **,+ | p.01=0.010 | p.01=0.009 | p.01=0.010 | p.01=0.011 0 |

+-------------+-----------------+-----------------+-----------------+-----------------+-----------------+

|[1.01, 2.56)| ks.D=0.028 *** | ks.D=0.019 * | ks.D=0.013 | ks.D=0.020 * | ks.D=0.017 *** |

| | p.05=0.047 0 | p.05=0.048 0 | p.05=0.056 * | p.05=0.054 0 | p.05=0.052 00 |

| | p.01=0.011 | p.01=0.011 | p.01=0.010 | p.01=0.010 | p.01=0.011 0 |

+-------------+-----------------+-----------------+-----------------+-----------------+-----------------+

|[2.56,415.82]| ks.D=0.069 *** | ks.D=0.043 *** | ks.D=0.033 *** | ks.D=0.029 ** | ks.D=0.043 *** |

| | p.05=0.046(*),0 | p.05=0.048 0 | p.05=0.049 0 | p.05=0.053 0 | p.05=0.049 00 |

| | p.01=0.008 (*) | p.01=0.010 | p.01=0.011 | p.01=0.010 | p.01=0.010 0 |

+-------------+-----------------+-----------------+-----------------+-----------------+-----------------+

|ALL | ks.D=0.033 *** | ks.D=0.040 *** | ks.D=0.035 *** | ks.D=0.025 *** | ks.D=0.033 *** |

| | p.05=0.073***,++| p.05=0.069***,++| p.05=0.065***,++| p.05=0.062***,+ | p.05=0.067***,++|

| | p.01=0.022***,++| p.01=0.021***,++| p.01=0.018***,++| p.01=0.016***,++| p.01=0.020***,++|

+-------------+-----------------+-----------------+-----------------+-----------------+-----------------+
