# Supplementary material for: A New Calibrated Bayesian Internal Goodness-of-Fit Method: Sampled Posterior p-Values as Simple and General p-Values That Allow Double Use of the Data
Source: PLoS One. 2011 Mar 18;6(3):e14770. doi: 10.1371/journal.pone.0014770 (PMC3060804; doi:10.1371/journal.pone.0014770)
Supplement: Text S3 — Results of Scenario 3. (0.40 MB DOC) [file pone.0014770.s003.doc]

New Calibrated Bayesian Internal Goodness-of-Fit Methods: Sampled Posterior P-values as Simple and General P-values that Allow Double Use of the Data

Frédéric Gosselin

Cemagref, UR EFNO, F-45290 Nogent-sur-Vernisson, France

E-mail: [frederic.gosselin@cemagref.fr](mailto:frederic.gosselin@cemagref.fr)

*Results of Scenario 3*

# Text S3. Scenario 3 $p_{sp}$ and $p_{nsp}$ results

*Scenario 3*. The setting is the same as in Scenario 2, except that the values of $\theta_{0}$ in the statistical model are no longer fixed but drawn at random according to $\theta_{0}\sim\text{exp}\left[\text{Normal}\left(\text{1,.4}^{2}\right)\right]$ in the Poisson case, $\theta_{0}\sim\text{Normal}\left(\text{0,3}^{2}\right)$ in the normal case and $\theta_{0}\sim\text{Uniform}\left(.25,.75\right)$ in the Bernoulli case.

Simulations were performed on $p_{sp}$ and $p_{nsp}$. In the following tables, we display the Kolmogorow-Smirnov statistic of the comparison of the p-values with a uniform distribution (ks.D), the proportion of values in the 5% extreme positions on the interval [0;1] (p.05), and the same for 1% (p.01), according to the interval to which $\mu_{\sigma_{0}}$ (in rows) and $\left|\text{log}\left(\theta_{0}\right)-1\right|$ for Poisson models, $\left|\theta_{0}\right|$ for normal models or $\left|\theta_{0}-.5\right|$ for Bernoulli models (in columns) belonged. 100,000 data set samples were considered. The notation for the significance of the tests is the same as in Supplementary Text S1.

## Poisson models

$p_{nsp}$, $t=mean$

+-------------+-----------------+-----------------+-----------------+-----------------+-----------------+

| | [0.00,0.14) | [0.14,0.28) | [0.28,0.47) | [0.47,1.73] | ALL |

+-------------+-----------------+-----------------+-----------------+-----------------+-----------------+

|[0.00, 0.40)| ks.D=0.078 *** | ks.D=0.085 *** | ks.D=0.136 *** | ks.D=0.192 *** | ks.D=0.120 *** |

| | p.05=0.122***,++| p.05=0.145***,++| p.05=0.212***,++| p.05=0.316***,++| p.05=0.197***,++|

| | p.01=0.055***,++| p.01=0.068***,++| p.01=0.126***,++| p.01=0.217***,++| p.01=0.115***,++|

+-------------+-----------------+-----------------+-----------------+-----------------+-----------------+

|[0.40, 1.01)| ks.D=0.012 | ks.D=0.012 | ks.D=0.036 *** | ks.D=0.082 *** | ks.D=0.033 *** |

| | p.05=0.056 * | p.05=0.058 ** | p.05=0.066***,++| p.05=0.107***,++| p.05=0.071***,++|

| | p.01=0.011 | p.01=0.011 | p.01=0.015***,++| p.01=0.041***,++| p.01=0.019***,++|

+-------------+-----------------+-----------------+-----------------+-----------------+-----------------+

|[1.01, 2.59)| ks.D=0.016 (*) | ks.D=0.011 | ks.D=0.013 | ks.D=0.031 *** | ks.D=0.010 * |

| | p.05=0.052 0 | p.05=0.046 0 | p.05=0.053 0 | p.05=0.066***,++| p.05=0.054 **,0 |

| | p.01=0.012 | p.01=0.010 | p.01=0.011 | p.01=0.016***,++| p.01=0.012 ** |

+-------------+-----------------+-----------------+-----------------+-----------------+-----------------+

|[2.59,530.25]| ks.D=0.018 * | ks.D=0.014 | ks.D=0.009 | ks.D=0.014 | ks.D=0.008 (*) |

| | p.05=0.051 0 | p.05=0.053 0 | p.05=0.051 0 | p.05=0.053 0 | p.05=0.052 00 |

| | p.01=0.011 | p.01=0.010 | p.01=0.010 | p.01=0.010 | p.01=0.010 0 |

+-------------+-----------------+-----------------+-----------------+-----------------+-----------------+

|ALL | ks.D=0.018 *** | ks.D=0.024 *** | ks.D=0.046 *** | ks.D=0.072 *** | ks.D=0.039 *** |

| | p.05=0.070***,++| p.05=0.075***,++| p.05=0.096***,++| p.05=0.136***,++| p.05=0.094***,++|

| | p.01=0.022***,++| p.01=0.025***,++| p.01=0.041***,++| p.01=0.071***,++| p.01=0.039***,++|

+-------------+-----------------+-----------------+-----------------+-----------------+-----------------+

$p_{nsp}$, $t=var$

+-------------+-----------------+-----------------+-----------------+-----------------+-----------------+

| | [0.00,0.14) | [0.14,0.28) | [0.28,0.47) | [0.47,1.73] | ALL |

+-------------+-----------------+-----------------+-----------------+-----------------+-----------------+

|[0.00, 0.40)| ks.D=0.020 * | ks.D=0.022 ** | ks.D=0.029 *** | ks.D=0.056 *** | ks.D=0.031 *** |

| | p.05=0.056 * | p.05=0.056 * | p.05=0.063***,+ | p.05=0.073***,++| p.05=0.062***,+ |

| | p.01=0.012 (*) | p.01=0.012 (*) | p.01=0.016***,++| p.01=0.020***,++| p.01=0.015***,++|

+-------------+-----------------+-----------------+-----------------+-----------------+-----------------+

|[0.40, 1.01)| ks.D=0.005 | ks.D=0.009 | ks.D=0.016 (*) | ks.D=0.018 * | ks.D=0.007 |

| | p.05=0.050 0 | p.05=0.050 0 | p.05=0.050 0 | p.05=0.053 0 | p.05=0.051 00 |

| | p.01=0.009 | p.01=0.008 | p.01=0.010 | p.01=0.012 | p.01=0.010 0 |

+-------------+-----------------+-----------------+-----------------+-----------------+-----------------+

|[1.01, 2.59)| ks.D=0.005 | ks.D=0.008 | ks.D=0.011 | ks.D=0.005 | ks.D=0.004 |

| | p.05=0.042 ** | p.05=0.051 0 | p.05=0.048 0 | p.05=0.049 0 | p.05=0.048(*),00|

| | p.01=0.007 * | p.01=0.010 | p.01=0.009 | p.01=0.008 (*) | p.01=0.008 ** |

+-------------+-----------------+-----------------+-----------------+-----------------+-----------------+

|[2.59,530.25]| ks.D=0.015 | ks.D=0.011 | ks.D=0.013 | ks.D=0.008 | ks.D=0.009 * |

| | p.05=0.051 0 | p.05=0.053 0 | p.05=0.053 0 | p.05=0.050 0 | p.05=0.052 00 |

| | p.01=0.010 | p.01=0.011 | p.01=0.011 | p.01=0.011 | p.01=0.011 0 |

+-------------+-----------------+-----------------+-----------------+-----------------+-----------------+

|ALL | ks.D=0.004 | ks.D=0.007 | ks.D=0.007 | ks.D=0.017 *** | ks.D=0.008 *** |

| | p.05=0.050 00 | p.05=0.053(*),0 | p.05=0.053 *,0 | p.05=0.057***,0 | p.05=0.053***,00|

| | p.01=0.010 0 | p.01=0.010 0 | p.01=0.011 * | p.01=0.013***,+ | p.01=0.011 **,0 |

+-------------+-----------------+-----------------+-----------------+-----------------+-----------------+

$p_{nsp}$, $t=skew$

+-------------+-----------------+-----------------+-----------------+-----------------+-----------------+

| | [0.00,0.14) | [0.14,0.28) | [0.28,0.47) | [0.47,1.73] | ALL |

+-------------+-----------------+-----------------+-----------------+-----------------+-----------------+

|[0.00, 0.40)| ks.D=0.011 | ks.D=0.012 | ks.D=0.011 | ks.D=0.026 ** | ks.D=0.011 ** |

| | p.05=0.050 0 | p.05=0.051 0 | p.05=0.050 0 | p.05=0.047 0 | p.05=0.050 00 |

| | p.01=0.009 | p.01=0.011 | p.01=0.010 | p.01=0.009 | p.01=0.010 0 |

+-------------+-----------------+-----------------+-----------------+-----------------+-----------------+

|[0.40, 1.01)| ks.D=0.011 | ks.D=0.010 | ks.D=0.008 | ks.D=0.006 | ks.D=0.005 |

| | p.05=0.051 0 | p.05=0.053 0 | p.05=0.048 0 | p.05=0.047 0 | p.05=0.050 00 |

| | p.01=0.010 | p.01=0.011 | p.01=0.010 | p.01=0.011 | p.01=0.011 0 |

+-------------+-----------------+-----------------+-----------------+-----------------+-----------------+

|[1.01, 2.59)| ks.D=0.010 | ks.D=0.013 | ks.D=0.011 | ks.D=0.008 | ks.D=0.004 |

| | p.05=0.051 0 | p.05=0.048 0 | p.05=0.046 0 | p.05=0.054 0 | p.05=0.050 00 |

| | p.01=0.010 | p.01=0.010 | p.01=0.008 (*) | p.01=0.009 | p.01=0.009 0 |

+-------------+-----------------+-----------------+-----------------+-----------------+-----------------+

|[2.59,530.25]| ks.D=0.010 | ks.D=0.005 | ks.D=0.011 | ks.D=0.013 | ks.D=0.006 |

| | p.05=0.045 *,0 | p.05=0.046 0 | p.05=0.046 0 | p.05=0.047 0 | p.05=0.046 **,0 |

| | p.01=0.008 | p.01=0.010 | p.01=0.009 | p.01=0.011 | p.01=0.010 0 |

+-------------+-----------------+-----------------+-----------------+-----------------+-----------------+

|ALL | ks.D=0.004 | ks.D=0.005 | ks.D=0.004 | ks.D=0.007 | ks.D=0.002 |

| | p.05=0.049 00 | p.05=0.050 00 | p.05=0.048(*),00| p.05=0.049 00 | p.05=0.049(*),00|

| | p.01=0.009 0 | p.01=0.010 0 | p.01=0.009 0 | p.01=0.010 0 | p.01=0.010 00 |

+-------------+-----------------+-----------------+-----------------+-----------------+-----------------+

$p_{nsp}$, $t=kurt$

+-------------+-----------------+-----------------+-----------------+-----------------+-----------------+

| | [0.00,0.14) | [0.14,0.28) | [0.28,0.47) | [0.47,1.73] | ALL |

+-------------+-----------------+-----------------+-----------------+-----------------+-----------------+

|[0.00, 0.40)| ks.D=0.007 | ks.D=0.014 | ks.D=0.008 | ks.D=0.009 | ks.D=0.003 |

| | p.05=0.052 0 | p.05=0.050 0 | p.05=0.052 0 | p.05=0.047 0 | p.05=0.050 00 |

| | p.01=0.011 | p.01=0.010 | p.01=0.009 | p.01=0.011 | p.01=0.010 0 |

+-------------+-----------------+-----------------+-----------------+-----------------+-----------------+

|[0.40, 1.01)| ks.D=0.011 | ks.D=0.012 | ks.D=0.013 | ks.D=0.017 (*) | ks.D=0.004 |

| | p.05=0.048 0 | p.05=0.053 0 | p.05=0.053 0 | p.05=0.050 0 | p.05=0.051 00 |

| | p.01=0.008 | p.01=0.012 | p.01=0.012 | p.01=0.012 | p.01=0.011 |

+-------------+-----------------+-----------------+-----------------+-----------------+-----------------+

|[1.01, 2.59)| ks.D=0.008 | ks.D=0.011 | ks.D=0.007 | ks.D=0.007 | ks.D=0.004 |

| | p.05=0.051 0 | p.05=0.047 0 | p.05=0.042 ** | p.05=0.051 0 | p.05=0.048 00 |

| | p.01=0.010 | p.01=0.009 | p.01=0.009 | p.01=0.011 | p.01=0.010 0 |

+-------------+-----------------+-----------------+-----------------+-----------------+-----------------+

|[2.59,530.25]| ks.D=0.013 | ks.D=0.008 | ks.D=0.011 | ks.D=0.010 | ks.D=0.005 |

| | p.05=0.053 0 | p.05=0.051 0 | p.05=0.045(*),0 | p.05=0.049 0 | p.05=0.050 00 |

| | p.01=0.011 | p.01=0.010 | p.01=0.008 (*) | p.01=0.011 | p.01=0.010 0 |

+-------------+-----------------+-----------------+-----------------+-----------------+-----------------+

|ALL | ks.D=0.004 | ks.D=0.004 | ks.D=0.004 | ks.D=0.005 | ks.D=0.002 |

| | p.05=0.051 00 | p.05=0.050 00 | p.05=0.048 00 | p.05=0.049 00 | p.05=0.050 00 |

| | p.01=0.010 0 | p.01=0.010 0 | p.01=0.009 0 | p.01=0.011 (*) | p.01=0.010 00 |

+-------------+-----------------+-----------------+-----------------+-----------------+-----------------+

$p_{nsp}$, $t=Z_{a}$

+-------------+-----------------+-----------------+-----------------+-----------------+-----------------+

| | [0.00,0.14) | [0.14,0.28) | [0.28,0.47) | [0.47,1.73] | ALL |

+-------------+-----------------+-----------------+-----------------+-----------------+-----------------+

|[0.00, 0.40)| ks.D=0.083 *** | ks.D=0.102 *** | ks.D=0.160 *** | ks.D=0.260 *** | ks.D=0.148 *** |

| | p.05=0.093***,++| p.05=0.106***,++| p.05=0.157***,++| p.05=0.241***,++| p.05=0.148***,++|

| | p.01=0.034***,++| p.01=0.046***,++| p.01=0.086***,++| p.01=0.164***,++| p.01=0.082***,++|

+-------------+-----------------+-----------------+-----------------+-----------------+-----------------+

|[0.40, 1.01)| ks.D=0.015 (*) | ks.D=0.024 ** | ks.D=0.031 *** | ks.D=0.064 *** | ks.D=0.032 *** |

| | p.05=0.053 0 | p.05=0.053 0 | p.05=0.054 0 | p.05=0.081***,++| p.05=0.060***,+ |

| | p.01=0.009 | p.01=0.011 | p.01=0.013 * | p.01=0.025***,++| p.01=0.014***,++|

+-------------+-----------------+-----------------+-----------------+-----------------+-----------------+

|[1.01, 2.59)| ks.D=0.011 | ks.D=0.007 | ks.D=0.011 | ks.D=0.024 ** | ks.D=0.007 |

| | p.05=0.050 0 | p.05=0.054 0 | p.05=0.046 0 | p.05=0.056 * | p.05=0.051 00 |

| | p.01=0.012 | p.01=0.008 | p.01=0.007 * | p.01=0.014 **,+ | p.01=0.010 0 |

+-------------+-----------------+-----------------+-----------------+-----------------+-----------------+

|[2.59,530.25]| ks.D=0.016 (*) | ks.D=0.010 | ks.D=0.011 | ks.D=0.012 | ks.D=0.005 |

| | p.05=0.050 0 | p.05=0.053 0 | p.05=0.051 0 | p.05=0.050 0 | p.05=0.051 00 |

| | p.01=0.009 | p.01=0.011 | p.01=0.008 | p.01=0.008 | p.01=0.009 0 |

+-------------+-----------------+-----------------+-----------------+-----------------+-----------------+

|ALL | ks.D=0.024 *** | ks.D=0.033 *** | ks.D=0.045 *** | ks.D=0.084 *** | ks.D=0.045 *** |

| | p.05=0.062***,+ | p.05=0.066***,++| p.05=0.077***,++| p.05=0.108***,++| p.05=0.078***,++|

| | p.01=0.016***,++| p.01=0.019***,++| p.01=0.029***,++| p.01=0.053***,++| p.01=0.029***,++|

+-------------+-----------------+-----------------+-----------------+-----------------+-----------------+

$p_{sp}$, $t=mean$

+-------------+-----------------+-----------------+-----------------+-----------------+-----------------+

| | [0.00,0.14) | [0.14,0.28) | [0.28,0.47) | [0.47,1.73] | ALL |

+-------------+-----------------+-----------------+-----------------+-----------------+-----------------+

|[0.00, 0.40)| ks.D=0.082 *** | ks.D=0.091 *** | ks.D=0.141 *** | ks.D=0.202 *** | ks.D=0.126 *** |

| | p.05=0.128***,++| p.05=0.151***,++| p.05=0.216***,++| p.05=0.326***,++| p.05=0.204***,++|

| | p.01=0.060***,++| p.01=0.075***,++| p.01=0.133***,++| p.01=0.225***,++| p.01=0.122***,++|

+-------------+-----------------+-----------------+-----------------+-----------------+-----------------+

|[0.40, 1.01)| ks.D=0.013 | ks.D=0.019 * | ks.D=0.042 *** | ks.D=0.086 *** | ks.D=0.036 *** |

| | p.05=0.056 * | p.05=0.055 (*) | p.05=0.070***,++| p.05=0.109***,++| p.05=0.072***,++|

| | p.01=0.013 *,+ | p.01=0.011 | p.01=0.016***,++| p.01=0.045***,++| p.01=0.021***,++|

+-------------+-----------------+-----------------+-----------------+-----------------+-----------------+

|[1.01, 2.59)| ks.D=0.017 * | ks.D=0.008 | ks.D=0.013 | ks.D=0.032 *** | ks.D=0.010 * |

| | p.05=0.052 0 | p.05=0.050 0 | p.05=0.055 (*) | p.05=0.067***,++| p.05=0.056***,0 |

| | p.01=0.010 | p.01=0.009 | p.01=0.011 | p.01=0.016***,++| p.01=0.012 * |

+-------------+-----------------+-----------------+-----------------+-----------------+-----------------+

|[2.59,530.25]| ks.D=0.018 * | ks.D=0.014 | ks.D=0.007 | ks.D=0.013 | ks.D=0.011 ** |

| | p.05=0.051 0 | p.05=0.053 0 | p.05=0.053 0 | p.05=0.053 0 | p.05=0.053(*),00|

| | p.01=0.009 | p.01=0.012 | p.01=0.008 | p.01=0.011 | p.01=0.010 0 |

+-------------+-----------------+-----------------+-----------------+-----------------+-----------------+

|ALL | ks.D=0.020 *** | ks.D=0.025 *** | ks.D=0.046 *** | ks.D=0.076 *** | ks.D=0.040 *** |

| | p.05=0.072***,++| p.05=0.077***,++| p.05=0.099***,++| p.05=0.139***,++| p.05=0.096***,++|

| | p.01=0.023***,++| p.01=0.027***,++| p.01=0.042***,++| p.01=0.075***,++| p.01=0.041***,++|

+-------------+-----------------+-----------------+-----------------+-----------------+-----------------+

$p_{sp}$, $t=var$

+-------------+-----------------+-----------------+-----------------+-----------------+-----------------+

| | [0.00,0.14) | [0.14,0.28) | [0.28,0.47) | [0.47,1.73] | ALL |

+-------------+-----------------+-----------------+-----------------+-----------------+-----------------+

|[0.00, 0.40)| ks.D=0.040 *** | ks.D=0.048 *** | ks.D=0.069 *** | ks.D=0.100 *** | ks.D=0.062 *** |

| | p.05=0.074***,++| p.05=0.076***,++| p.05=0.102***,++| p.05=0.142***,++| p.05=0.098***,++|

| | p.01=0.021***,++| p.01=0.021***,++| p.01=0.038***,++| p.01=0.069***,++| p.01=0.037***,++|

+-------------+-----------------+-----------------+-----------------+-----------------+-----------------+

|[0.40, 1.01)| ks.D=0.007 | ks.D=0.012 | ks.D=0.019 * | ks.D=0.035 *** | ks.D=0.015 *** |

| | p.05=0.052 0 | p.05=0.050 0 | p.05=0.051 0 | p.05=0.066***,++| p.05=0.055 **,0 |

| | p.01=0.011 | p.01=0.009 | p.01=0.011 | p.01=0.014**,++ | p.01=0.011 * |

+-------------+-----------------+-----------------+-----------------+-----------------+-----------------+

|[1.01, 2.59)| ks.D=0.009 | ks.D=0.009 | ks.D=0.010 | ks.D=0.017 * | ks.D=0.005 |

| | p.05=0.047 0 | p.05=0.050 0 | p.05=0.045(*),0 | p.05=0.055(*),0 | p.05=0.049 00 |

| | p.01=0.009 | p.01=0.011 | p.01=0.009 | p.01=0.009 | p.01=0.009 0 |

+-------------+-----------------+-----------------+-----------------+-----------------+-----------------+

|[2.59,530.25]| ks.D=0.023 ** | ks.D=0.013 | ks.D=0.011 | ks.D=0.007 | ks.D=0.010 ** |

| | p.05=0.047 0 | p.05=0.052 0 | p.05=0.050 0 | p.05=0.052 0 | p.05=0.050 00 |

| | p.01=0.012 | p.01=0.012 | p.01=0.012 | p.01=0.011 | p.01=0.012 * |

+-------------+-----------------+-----------------+-----------------+-----------------+-----------------+

|ALL | ks.D=0.006 | ks.D=0.013 ** | ks.D=0.020 *** | ks.D=0.035 *** | ks.D=0.018 *** |

| | p.05=0.055 **,0 | p.05=0.057***,0 | p.05=0.062***,+ | p.05=0.079***,++| p.05=0.063***,++|

| | p.01=0.013***,++| p.01=0.013***,+ | p.01=0.018***,++| p.01=0.026***,++| p.01=0.017***,++|

+-------------+-----------------+-----------------+-----------------+-----------------+-----------------+

$p_{sp}$, $t=p_{0}$

+-------------+-----------------+-----------------+-----------------+-----------------+-----------------+

| | [0.00,0.14) | [0.14,0.28) | [0.28,0.47) | [0.47,1.73] | ALL |

+-------------+-----------------+-----------------+-----------------+-----------------+-----------------+

|[0.00, 0.40)| ks.D=0.050 *** | ks.D=0.060 *** | ks.D=0.087 *** | ks.D=0.118 *** | ks.D=0.075 *** |

| | p.05=0.075***,++| p.05=0.084***,++| p.05=0.112***,++| p.05=0.166***,++| p.05=0.108***,++|

| | p.01=0.025***,++| p.01=0.028***,++| p.01=0.049***,++| p.01=0.091***,++| p.01=0.048***,++|

+-------------+-----------------+-----------------+-----------------+-----------------+-----------------+

|[0.40, 1.01)| ks.D=0.014 | ks.D=0.010 | ks.D=0.020 * | ks.D=0.043 *** | ks.D=0.017 *** |

| | p.05=0.049 0 | p.05=0.054 0 | p.05=0.058 ** | p.05=0.063***,+ | p.05=0.056***,0 |

| | p.01=0.012 | p.01=0.012 (*) | p.01=0.011 | p.01=0.020***,++| p.01=0.014***,++|

+-------------+-----------------+-----------------+-----------------+-----------------+-----------------+

|[1.01, 2.59)| ks.D=0.014 | ks.D=0.012 | ks.D=0.007 | ks.D=0.016 (*) | ks.D=0.003 |

| | p.05=0.051 0 | p.05=0.049 0 | p.05=0.050 0 | p.05=0.058 ** | p.05=0.052 00 |

| | p.01=0.010 | p.01=0.010 | p.01=0.010 | p.01=0.010 | p.01=0.010 0 |

+-------------+-----------------+-----------------+-----------------+-----------------+-----------------+

|[2.59,530.25]| ks.D=0.025 ** | ks.D=0.021 * | ks.D=0.016 (*) | ks.D=0.017 (*) | ks.D=0.014 *** |

| | p.05=0.053 0 | p.05=0.051 0 | p.05=0.054 0 | p.05=0.050 0 | p.05=0.052 00 |

| | p.01=0.012 | p.01=0.010 | p.01=0.010 | p.01=0.011 | p.01=0.010 0 |

+-------------+-----------------+-----------------+-----------------+-----------------+-----------------+

|ALL | ks.D=0.013 ** | ks.D=0.015 *** | ks.D=0.025 *** | ks.D=0.039 *** | ks.D=0.022 *** |

| | p.05=0.057***,0 | p.05=0.059***,+ | p.05=0.068***,++| p.05=0.084***,++| p.05=0.067***,++|

| | p.01=0.015***,++| p.01=0.015***,++| p.01=0.020***,++| p.01=0.033***,++| p.01=0.021***,++|

+-------------+-----------------+-----------------+-----------------+-----------------+-----------------+

$p_{sp}$, $d=meanc$

+-------------+-----------------+-----------------+-----------------+-----------------+-----------------+

| | [0.00,0.14) | [0.14,0.28) | [0.28,0.47) | [0.47,1.73] | ALL |

+-------------+-----------------+-----------------+-----------------+-----------------+-----------------+

|[0.00, 0.40)| ks.D=0.082 *** | ks.D=0.091 *** | ks.D=0.141 *** | ks.D=0.202 *** | ks.D=0.126 *** |

| | p.05=0.128***,++| p.05=0.151***,++| p.05=0.216***,++| p.05=0.326***,++| p.05=0.204***,++|

| | p.01=0.060***,++| p.01=0.075***,++| p.01=0.133***,++| p.01=0.225***,++| p.01=0.122***,++|

+-------------+-----------------+-----------------+-----------------+-----------------+-----------------+

|[0.40, 1.01)| ks.D=0.013 | ks.D=0.019 * | ks.D=0.042 *** | ks.D=0.086 *** | ks.D=0.036 *** |

| | p.05=0.056 * | p.05=0.055(*),0 | p.05=0.070***,++| p.05=0.109***,++| p.05=0.072***,++|

| | p.01=0.013 *,+ | p.01=0.011 | p.01=0.016***,++| p.01=0.045***,++| p.01=0.021***,++|

+-------------+-----------------+-----------------+-----------------+-----------------+-----------------+

|[1.01, 2.59)| ks.D=0.017 * | ks.D=0.008 | ks.D=0.013 | ks.D=0.032 *** | ks.D=0.010 * |

| | p.05=0.052 0 | p.05=0.050 0 | p.05=0.055 (*) | p.05=0.067***,++| p.05=0.056***,0 |

| | p.01=0.010 | p.01=0.009 | p.01=0.011 | p.01=0.016***,++| p.01=0.012 * |

+-------------+-----------------+-----------------+-----------------+-----------------+-----------------+

|[2.59,530.25]| ks.D=0.018 * | ks.D=0.014 | ks.D=0.007 | ks.D=0.013 | ks.D=0.011 ** |

| | p.05=0.051 0 | p.05=0.053 0 | p.05=0.053 0 | p.05=0.053 0 | p.05=0.053(*),0 |

| | p.01=0.009 | p.01=0.012 | p.01=0.008 | p.01=0.011 | p.01=0.010 0 |

+-------------+-----------------+-----------------+-----------------+-----------------+-----------------+

|ALL | ks.D=0.020 *** | ks.D=0.025 *** | ks.D=0.046 *** | ks.D=0.076 *** | ks.D=0.040 *** |

| | p.05=0.072***,++| p.05=0.077***,++| p.05=0.099***,++| p.05=0.139***,++| p.05=0.096***,++|

| | p.01=0.023***,++| p.01=0.027***,++| p.01=0.042***,++| p.01=0.075***,++| p.01=0.041***,++|

+-------------+-----------------+-----------------+-----------------+-----------------+-----------------+

$p_{sp}$, $d=varc$

+-------------+-----------------+-----------------+-----------------+-----------------+-----------------+

| | [0.00,0.14) | [0.14,0.28) | [0.28,0.47) | [0.47,1.73] | ALL |

+-------------+-----------------+-----------------+-----------------+-----------------+-----------------+

|[0.00, 0.40)| ks.D=0.040 *** | ks.D=0.048 *** | ks.D=0.069 *** | ks.D=0.100 *** | ks.D=0.062 *** |

| | p.05=0.074***,++| p.05=0.076***,++| p.05=0.102***,++| p.05=0.142***,++| p.05=0.098***,++|

| | p.01=0.021***,++| p.01=0.021***,++| p.01=0.038***,++| p.01=0.069***,++| p.01=0.037***,++|

+-------------+-----------------+-----------------+-----------------+-----------------+-----------------+

|[0.40, 1.01)| ks.D=0.007 | ks.D=0.012 | ks.D=0.019 * | ks.D=0.035 *** | ks.D=0.015 *** |

| | p.05=0.052 0 | p.05=0.050 0 | p.05=0.051 0 | p.05=0.066***,++| p.05=0.055 **,0 |

| | p.01=0.011 | p.01=0.009 | p.01=0.011 | p.01=0.014**,++ | p.01=0.011 * |

+-------------+-----------------+-----------------+-----------------+-----------------+-----------------+

|[1.01, 2.59)| ks.D=0.009 | ks.D=0.009 | ks.D=0.010 | ks.D=0.017 * | ks.D=0.005 |

| | p.05=0.047 0 | p.05=0.050 0 | p.05=0.045(*),0 | p.05=0.055(*),0 | p.05=0.049 00 |

| | p.01=0.009 | p.01=0.011 | p.01=0.009 | p.01=0.009 | p.01=0.009 0 |

+-------------+-----------------+-----------------+-----------------+-----------------+-----------------+

|[2.59,530.25]| ks.D=0.023 ** | ks.D=0.013 | ks.D=0.011 | ks.D=0.007 | ks.D=0.010 ** |

| | p.05=0.047 0 | p.05=0.052 0 | p.05=0.050 0 | p.05=0.052 0 | p.05=0.050 00 |

| | p.01=0.012 | p.01=0.012 | p.01=0.012 | p.01=0.011 | p.01=0.012 * |

+-------------+-----------------+-----------------+-----------------+-----------------+-----------------+

|ALL | ks.D=0.006 | ks.D=0.013 ** | ks.D=0.020 *** | ks.D=0.035 *** | ks.D=0.018 *** |

| | p.05=0.055 **,0 | p.05=0.057***,0 | p.05=0.062***,+ | p.05=0.079***,++| p.05=0.063***,++|

| | p.01=0.013***,++| p.01=0.013***,+ | p.01=0.018***,++| p.01=0.026***,++| p.01=0.017***,++|

+-------------+-----------------+-----------------+-----------------+-----------------+-----------------+

$p_{sp}$, $d=LL$

+-------------+-----------------+-----------------+-----------------+-----------------+-----------------+

| | [0.00,0.14) | [0.14,0.28) | [0.28,0.47) | [0.47,1.73] | ALL |

+-------------+-----------------+-----------------+-----------------+-----------------+-----------------+

|[0.00, 0.40)| ks.D=0.059 *** | ks.D=0.058 *** | ks.D=0.065 *** | ks.D=0.064 *** | ks.D=0.060 *** |

| | p.05=0.078***,++| p.05=0.082***,++| p.05=0.095***,++| p.05=0.115***,++| p.05=0.092***,++|

| | p.01=0.025***,++| p.01=0.023***,++| p.01=0.037***,++| p.01=0.053***,++| p.01=0.034***,++|

+-------------+-----------------+-----------------+-----------------+-----------------+-----------------+

|[0.40, 1.01)| ks.D=0.008 | ks.D=0.012 | ks.D=0.022 ** | ks.D=0.030 *** | ks.D=0.016 *** |

| | p.05=0.051 0 | p.05=0.054 0 | p.05=0.052 0 | p.05=0.058 ** | p.05=0.054 **,0 |

| | p.01=0.011 | p.01=0.010 | p.01=0.013 * | p.01=0.015***,++| p.01=0.012 **,+ |

+-------------+-----------------+-----------------+-----------------+-----------------+-----------------+

|[1.01, 2.58)| ks.D=0.011 | ks.D=0.014 | ks.D=0.007 | ks.D=0.014 | ks.D=0.008 (*) |

| | p.05=0.048 0 | p.05=0.050 0 | p.05=0.049 0 | p.05=0.053 0 | p.05=0.050 00 |

| | p.01=0.010 | p.01=0.010 | p.01=0.011 | p.01=0.011 | p.01=0.010 0 |

+-------------+-----------------+-----------------+-----------------+-----------------+-----------------+

|[2.58,530.25]| ks.D=0.025 ** | ks.D=0.018 * | ks.D=0.018 * | ks.D=0.018 * | ks.D=0.017 *** |

| | p.05=0.050 0 | p.05=0.048 0 | p.05=0.051 0 | p.05=0.052 0 | p.05=0.050 00 |

| | p.01=0.011 | p.01=0.011 | p.01=0.013 * | p.01=0.011 | p.01=0.011 * |

+-------------+-----------------+-----------------+-----------------+-----------------+-----------------+

|ALL | ks.D=0.011 ** | ks.D=0.015 *** | ks.D=0.020 *** | ks.D=0.023 *** | ks.D=0.016 *** |

| | p.05=0.057***,0 | p.05=0.059***,+ | p.05=0.062***,+ | p.05=0.070***,++| p.05=0.062***,++|

| | p.01=0.014***,++| p.01=0.013***,++| p.01=0.019***,++| p.01=0.023***,++| p.01=0.017***,++|

+-------------+-----------------+-----------------+-----------------+-----------------+-----------------+

## Normal models

$p_{nsp}$, $t=mean$

+-------------+-----------------+-----------------+-----------------+-----------------+-----------------+

| | [0.0, 1.1) | [1.1, 2.1) | [2.1, 3.6) | [3.6,13.9] | ALL |

+-------------+-----------------+-----------------+-----------------+-----------------+-----------------+

|[0.00, 0.40)| ks.D=0.007 | ks.D=0.015 | ks.D=0.015 | ks.D=0.034 *** | ks.D=0.012 ** |

| | p.05=0.050 0 | p.05=0.061 **,+ | p.05=0.059 ** | p.05=0.076***,++| p.05=0.061***,+ |

| | p.01=0.010 | p.01=0.013 * | p.01=0.013 * | p.01=0.020***,++| p.01=0.014***,++|

+-------------+-----------------+-----------------+-----------------+-----------------+-----------------+

|[0.40, 1.01)| ks.D=0.010 | ks.D=0.013 | ks.D=0.011 | ks.D=0.028 ** | ks.D=0.011 ** |

| | p.05=0.054(*),0 | p.05=0.055 (*) | p.05=0.059 ** | p.05=0.067***,++| p.05=0.059***,+ |

| | p.01=0.011 | p.01=0.011 | p.01=0.009 | p.01=0.016***,++| p.01=0.012 ** |

+-------------+-----------------+-----------------+-----------------+-----------------+-----------------+

|[1.01, 2.58)| ks.D=0.011 | ks.D=0.007 | ks.D=0.015 (*) | ks.D=0.025 ** | ks.D=0.008 (*) |

| | p.05=0.052 0 | p.05=0.052 0 | p.05=0.062***,+ | p.05=0.059 ** | p.05=0.056***,0 |

| | p.01=0.011 | p.01=0.011 | p.01=0.015**,++ | p.01=0.014**,++ | p.01=0.013***,+ |

+-------------+-----------------+-----------------+-----------------+-----------------+-----------------+

|[2.58,372.79]| ks.D=0.009 | ks.D=0.007 | ks.D=0.007 | ks.D=0.023 ** | ks.D=0.007 |

| | p.05=0.052 0 | p.05=0.054 0 | p.05=0.057 * | p.05=0.068***,++| p.05=0.057***,+ |

| | p.01=0.009 | p.01=0.010 | p.01=0.015***,++| p.01=0.017***,++| p.01=0.013***,+ |

+-------------+-----------------+-----------------+-----------------+-----------------+-----------------+

|ALL | ks.D=0.006 | ks.D=0.008 (*) | ks.D=0.010 * | ks.D=0.021 *** | ks.D=0.009 *** |

| | p.05=0.052 00 | p.05=0.056 **,0 | p.05=0.059***,+ | p.05=0.068***,++| p.05=0.058***,+ |

| | p.01=0.010 0 | p.01=0.011 * | p.01=0.013***,+ | p.01=0.017***,++| p.01=0.013***,++|

+-------------+-----------------+-----------------+-----------------+-----------------+-----------------+

$p_{nsp}$, $t=var$

+-------------+-----------------+-----------------+-----------------+-----------------+-----------------+

| | [0.0, 1.1) | [1.1, 2.1) | [2.1, 3.6) | [3.6,13.9] | ALL |

+-------------+-----------------+-----------------+-----------------+-----------------+-----------------+

|[0.00, 0.40)| ks.D=0.073 *** | ks.D=0.078 *** | ks.D=0.116 *** | ks.D=0.273 *** | ks.D=0.124 *** |

| | p.05=0.134***,++| p.05=0.158***,++| p.05=0.160***,++| p.05=0.272***,++| p.05=0.179***,++|

| | p.01=0.062***,++| p.01=0.076***,++| p.01=0.073***,++| p.01=0.160***,++| p.01=0.091***,++|

+-------------+-----------------+-----------------+-----------------+-----------------+-----------------+

|[0.40, 1.01)| ks.D=0.015 (*) | ks.D=0.053 *** | ks.D=0.119 *** | ks.D=0.329 *** | ks.D=0.123 *** |

| | p.05=0.058 ** | p.05=0.067***,++| p.05=0.098***,++| p.05=0.242***,++| p.05=0.115***,++|

| | p.01=0.013 * | p.01=0.013 *,+ | p.01=0.028***,++| p.01=0.134***,++| p.01=0.046***,++|

+-------------+-----------------+-----------------+-----------------+-----------------+-----------------+

|[1.01, 2.58)| ks.D=0.021 ** | ks.D=0.051 *** | ks.D=0.141 *** | ks.D=0.356 *** | ks.D=0.134 *** |

| | p.05=0.051 0 | p.05=0.057 * | p.05=0.089***,++| p.05=0.256***,++| p.05=0.110***,++|

| | p.01=0.009 | p.01=0.014 **,+ | p.01=0.026***,++| p.01=0.141***,++| p.01=0.046***,++|

+-------------+-----------------+-----------------+-----------------+-----------------+-----------------+

|[2.58,372.79]| ks.D=0.011 | ks.D=0.065 *** | ks.D=0.152 *** | ks.D=0.369 *** | ks.D=0.139 *** |

| | p.05=0.045(*),0 | p.05=0.060 ** | p.05=0.091***,++| p.05=0.264***,++| p.05=0.112***,++|

| | p.01=0.009 | p.01=0.011 | p.01=0.027***,++| p.01=0.154***,++| p.01=0.049***,++|

+-------------+-----------------+-----------------+-----------------+-----------------+-----------------+

|ALL | ks.D=0.019 *** | ks.D=0.054 *** | ks.D=0.127 *** | ks.D=0.329 *** | ks.D=0.127 *** |

| | p.05=0.072***,++| p.05=0.086***,++| p.05=0.109***,++| p.05=0.259***,++| p.05=0.129***,++|

| | p.01=0.024***,++| p.01=0.029***,++| p.01=0.039***,++| p.01=0.147***,++| p.01=0.058***,++|

+-------------+-----------------+-----------------+-----------------+-----------------+-----------------+

$p_{nsp}$, $t=skew$

+-------------+-----------------+-----------------+-----------------+-----------------+-----------------+

| | [0.0, 1.1) | [1.1, 2.1) | [2.1, 3.6) | [3.6,13.9] | ALL |

+-------------+-----------------+-----------------+-----------------+-----------------+-----------------+

|[0.00, 0.40)| ks.D=0.015 | ks.D=0.010 | ks.D=0.009 | ks.D=0.015 | ks.D=0.004 |

| | p.05=0.046 0 | p.05=0.045(*),0 | p.05=0.047 0 | p.05=0.053 0 | p.05=0.048 00 |

| | p.01=0.010 | p.01=0.008 (*) | p.01=0.010 | p.01=0.010 | p.01=0.010 0 |

+-------------+-----------------+-----------------+-----------------+-----------------+-----------------+

|[0.40, 1.01)| ks.D=0.007 | ks.D=0.013 | ks.D=0.008 | ks.D=0.018 * | ks.D=0.007 |

| | p.05=0.052 0 | p.05=0.053 0 | p.05=0.050 0 | p.05=0.055 (*) | p.05=0.052(*),00|

| | p.01=0.009 | p.01=0.008 | p.01=0.011 | p.01=0.012 (*) | p.01=0.010 0 |

+-------------+-----------------+-----------------+-----------------+-----------------+-----------------+

|[1.01, 2.58)| ks.D=0.022 ** | ks.D=0.019 * | ks.D=0.007 | ks.D=0.013 | ks.D=0.008 |

| | p.05=0.050 0 | p.05=0.049 0 | p.05=0.054 0 | p.05=0.057 * | p.05=0.052(*),00|

| | p.01=0.009 | p.01=0.013 * | p.01=0.011 | p.01=0.011 | p.01=0.011 |

+-------------+-----------------+-----------------+-----------------+-----------------+-----------------+

|[2.58,372.79]| ks.D=0.010 | ks.D=0.009 | ks.D=0.009 | ks.D=0.013 | ks.D=0.006 |

| | p.05=0.049 0 | p.05=0.051 0 | p.05=0.047 0 | p.05=0.048 0 | p.05=0.049 00 |

| | p.01=0.008 | p.01=0.011 | p.01=0.010 | p.01=0.008 (*) | p.01=0.009 0 |

+-------------+-----------------+-----------------+-----------------+-----------------+-----------------+

|ALL | ks.D=0.007 (*) | ks.D=0.007 | ks.D=0.005 | ks.D=0.003 | ks.D=0.002 |

| | p.05=0.049 00 | p.05=0.049 00 | p.05=0.049 00 | p.05=0.053 *,0 | p.05=0.050 00 |

| | p.01=0.009 0 | p.01=0.010 0 | p.01=0.010 0 | p.01=0.010 0 | p.01=0.010 00 |

+-------------+-----------------+-----------------+-----------------+-----------------+-----------------+

$p_{nsp}$, $t=kurt$

+-------------+-----------------+-----------------+-----------------+-----------------+-----------------+

| | [0.0, 1.1) | [1.1, 2.1) | [2.1, 3.6) | [3.6,13.9] | ALL |

+-------------+-----------------+-----------------+-----------------+-----------------+-----------------+

|[0.00, 0.40)| ks.D=0.009 | ks.D=0.007 | ks.D=0.009 | ks.D=0.013 | ks.D=0.003 |

| | p.05=0.053 0 | p.05=0.049 0 | p.05=0.050 0 | p.05=0.042 ** | p.05=0.049 00 |

| | p.01=0.011 | p.01=0.011 | p.01=0.012 | p.01=0.010 | p.01=0.011 |

+-------------+-----------------+-----------------+-----------------+-----------------+-----------------+

|[0.40, 1.01)| ks.D=0.012 | ks.D=0.010 | ks.D=0.006 | ks.D=0.022 ** | ks.D=0.011 ** |

| | p.05=0.047 0 | p.05=0.046 0 | p.05=0.053 0 | p.05=0.052 0 | p.05=0.049 00 |

| | p.01=0.010 | p.01=0.010 | p.01=0.010 | p.01=0.008 | p.01=0.010 0 |

+-------------+-----------------+-----------------+-----------------+-----------------+-----------------+

|[1.01, 2.58)| ks.D=0.010 | ks.D=0.011 | ks.D=0.013 | ks.D=0.009 | ks.D=0.004 |

| | p.05=0.046 0 | p.05=0.049 0 | p.05=0.052 0 | p.05=0.052 0 | p.05=0.050 00 |

| | p.01=0.008 (*) | p.01=0.011 | p.01=0.009 | p.01=0.012 | p.01=0.010 0 |

+-------------+-----------------+-----------------+-----------------+-----------------+-----------------+

|[2.58,372.79]| ks.D=0.013 | ks.D=0.012 | ks.D=0.014 | ks.D=0.009 | ks.D=0.006 |

| | p.05=0.053 0 | p.05=0.051 0 | p.05=0.047 0 | p.05=0.047 0 | p.05=0.050 00 |

| | p.01=0.010 | p.01=0.008 | p.01=0.008 * | p.01=0.009 | p.01=0.009 (*) |

+-------------+-----------------+-----------------+-----------------+-----------------+-----------------+

|ALL | ks.D=0.006 | ks.D=0.004 | ks.D=0.006 | ks.D=0.010 * | ks.D=0.004 |

| | p.05=0.050 00 | p.05=0.049 00 | p.05=0.050 00 | p.05=0.048 00 | p.05=0.049 00 |

| | p.01=0.010 0 | p.01=0.010 0 | p.01=0.010 0 | p.01=0.010 0 | p.01=0.010 00 |

+-------------+-----------------+-----------------+-----------------+-----------------+-----------------+

$p_{nsp}$, $t=Z_{a}$

+-------------+-----------------+-----------------+-----------------+-----------------+-----------------+

| | [0.0, 1.1) | [1.1, 2.1) | [2.1, 3.6) | [3.6,13.9] | ALL |

+-------------+-----------------+-----------------+-----------------+-----------------+-----------------+

|[0.00, 0.40)| ks.D=0.074 *** | ks.D=0.102 *** | ks.D=0.111 *** | ks.D=0.234 *** | ks.D=0.125 *** |

| | p.05=0.078***,++| p.05=0.088***,++| p.05=0.103***,++| p.05=0.184***,++| p.05=0.111***,++|

| | p.01=0.026***,++| p.01=0.032***,++| p.01=0.038***,++| p.01=0.097***,++| p.01=0.047***,++|

+-------------+-----------------+-----------------+-----------------+-----------------+-----------------+

|[0.40, 1.01)| ks.D=0.017 * | ks.D=0.035 *** | ks.D=0.059 *** | ks.D=0.219 *** | ks.D=0.078 *** |

| | p.05=0.051 0 | p.05=0.059 ** | p.05=0.074***,++| p.05=0.176***,++| p.05=0.089***,++|

| | p.01=0.012 | p.01=0.014 **,+ | p.01=0.021***,++| p.01=0.081***,++| p.01=0.031***,++|

+-------------+-----------------+-----------------+-----------------+-----------------+-----------------+

|[1.01, 2.58)| ks.D=0.007 | ks.D=0.018 (*) | ks.D=0.074 *** | ks.D=0.229 *** | ks.D=0.076 *** |

| | p.05=0.052 0 | p.05=0.055 (*) | p.05=0.077***,++| p.05=0.176***,++| p.05=0.088***,++|

| | p.01=0.010 | p.01=0.011 | p.01=0.021***,++| p.01=0.086***,++| p.01=0.031***,++|

+-------------+-----------------+-----------------+-----------------+-----------------+-----------------+

|[2.58,372.79]| ks.D=0.013 | ks.D=0.027 ** | ks.D=0.070 *** | ks.D=0.229 *** | ks.D=0.078 *** |

| | p.05=0.051 0 | p.05=0.053 0 | p.05=0.076***,++| p.05=0.189***,++| p.05=0.091***,++|

| | p.01=0.010 | p.01=0.013 * | p.01=0.019***,++| p.01=0.091***,++| p.01=0.032***,++|

+-------------+-----------------+-----------------+-----------------+-----------------+-----------------+

|ALL | ks.D=0.023 *** | ks.D=0.045 *** | ks.D=0.076 *** | ks.D=0.226 *** | ks.D=0.088 *** |

| | p.05=0.058***,+ | p.05=0.064***,++| p.05=0.082***,++| p.05=0.181***,++| p.05=0.095***,++|

| | p.01=0.015***,++| p.01=0.017***,++| p.01=0.025***,++| p.01=0.089***,++| p.01=0.035***,++|

+-------------+-----------------+-----------------+-----------------+-----------------+-----------------+

$p_{sp}$, $t=mean$

+-------------+-----------------+-----------------+-----------------+-----------------+-----------------+

| | [0.0, 1.1) | [1.1, 2.1) | [2.1, 3.6) | [3.6,13.9] | ALL |

+-------------+-----------------+-----------------+-----------------+-----------------+-----------------+

|[0.00, 0.40)| ks.D=0.007 | ks.D=0.016 (*) | ks.D=0.016 (*) | ks.D=0.033 *** | ks.D=0.012 ** |

| | p.05=0.050 0 | p.05=0.061 **,+ | p.05=0.059 ** | p.05=0.077***,++| p.05=0.061***,+ |

| | p.01=0.010 | p.01=0.013 * | p.01=0.013 * | p.01=0.020***,++| p.01=0.014***,++|

+-------------+-----------------+-----------------+-----------------+-----------------+-----------------+

|[0.40, 1.01)| ks.D=0.010 | ks.D=0.012 | ks.D=0.011 | ks.D=0.029 *** | ks.D=0.011 ** |

| | p.05=0.055(*),0 | p.05=0.055 (*) | p.05=0.058 ** | p.05=0.067***,++| p.05=0.058***,+ |

| | p.01=0.010 | p.01=0.012 | p.01=0.009 | p.01=0.016***,++| p.01=0.012 * |

+-------------+-----------------+-----------------+-----------------+-----------------+-----------------+

|[1.01, 2.58)| ks.D=0.011 | ks.D=0.008 | ks.D=0.016 (*) | ks.D=0.026 ** | ks.D=0.009 (*) |

| | p.05=0.052 0 | p.05=0.052 0 | p.05=0.063***,+ | p.05=0.059 ** | p.05=0.057***,0 |

| | p.01=0.011 | p.01=0.011 | p.01=0.014**,++ | p.01=0.015**,++ | p.01=0.013***,+ |

+-------------+-----------------+-----------------+-----------------+-----------------+-----------------+

|[2.58,372.79]| ks.D=0.010 | ks.D=0.008 | ks.D=0.007 | ks.D=0.023 ** | ks.D=0.006 |

| | p.05=0.052 0 | p.05=0.054 0 | p.05=0.057 * | p.05=0.067***,++| p.05=0.057***,0 |

| | p.01=0.009 | p.01=0.010 | p.01=0.015***,++| p.01=0.017***,++| p.01=0.013***,+ |

+-------------+-----------------+-----------------+-----------------+-----------------+-----------------+

|ALL | ks.D=0.006 | ks.D=0.008 (*) | ks.D=0.009 * | ks.D=0.021 *** | ks.D=0.009 *** |

| | p.05=0.052 00 | p.05=0.055 **,0 | p.05=0.059***,+ | p.05=0.068***,++| p.05=0.058***,+ |

| | p.01=0.010 0 | p.01=0.011 * | p.01=0.013***,+ | p.01=0.017***,++| p.01=0.013***,++|

+-------------+-----------------+-----------------+-----------------+-----------------+-----------------+

$p_{sp}$, $t=var$

+-------------+-----------------+-----------------+-----------------+-----------------+-----------------+

| | [0.0, 1.1) | [1.1, 2.1) | [2.1, 3.6) | [3.6,13.9] | ALL |

+-------------+-----------------+-----------------+-----------------+-----------------+-----------------+

|[0.00, 0.40)| ks.D=0.073 *** | ks.D=0.078 *** | ks.D=0.116 *** | ks.D=0.272 *** | ks.D=0.123 *** |

| | p.05=0.134***,++| p.05=0.158***,++| p.05=0.159***,++| p.05=0.272***,++| p.05=0.178***,++|

| | p.01=0.063***,++| p.01=0.076***,++| p.01=0.073***,++| p.01=0.159***,++| p.01=0.091***,++|

+-------------+-----------------+-----------------+-----------------+-----------------+-----------------+

|[0.40, 1.01)| ks.D=0.014 | ks.D=0.052 *** | ks.D=0.119 *** | ks.D=0.328 *** | ks.D=0.123 *** |

| | p.05=0.058 ** | p.05=0.067***,++| p.05=0.099***,++| p.05=0.243***,++| p.05=0.115***,++|

| | p.01=0.013 * | p.01=0.013 *,+ | p.01=0.029***,++| p.01=0.134***,++| p.01=0.046***,++|

+-------------+-----------------+-----------------+-----------------+-----------------+-----------------+

|[1.01, 2.58)| ks.D=0.020 ** | ks.D=0.050 *** | ks.D=0.141 *** | ks.D=0.356 *** | ks.D=0.134 *** |

| | p.05=0.050 0 | p.05=0.057 * | p.05=0.088***,++| p.05=0.255***,++| p.05=0.110***,++|

| | p.01=0.010 | p.01=0.014 **,+ | p.01=0.026***,++| p.01=0.141***,++| p.01=0.046***,++|

+-------------+-----------------+-----------------+-----------------+-----------------+-----------------+

|[2.58,372.79]| ks.D=0.011 | ks.D=0.065 *** | ks.D=0.152 *** | ks.D=0.369 *** | ks.D=0.139 *** |

| | p.05=0.045(*),0 | p.05=0.060 ** | p.05=0.090***,++| p.05=0.265***,++| p.05=0.112***,++|

| | p.01=0.010 | p.01=0.011 | p.01=0.027***,++| p.01=0.154***,++| p.01=0.049***,++|

+-------------+-----------------+-----------------+-----------------+-----------------+-----------------+

|ALL | ks.D=0.019 *** | ks.D=0.053 *** | ks.D=0.126 *** | ks.D=0.329 *** | ks.D=0.127 *** |

| | p.05=0.072***,++| p.05=0.086***,++| p.05=0.109***,++| p.05=0.259***,++| p.05=0.129***,++|

| | p.01=0.024***,++| p.01=0.029***,++| p.01=0.039***,++| p.01=0.147***,++| p.01=0.058***,++|

+-------------+-----------------+-----------------+-----------------+-----------------+-----------------+

$p_{sp}$, $t=p_{0}$

+-------------+-----------------+-----------------+-----------------+-----------------+-----------------+

| | [0.0, 1.1) | [1.1, 2.1) | [2.1, 3.6) | [3.6,13.9] | ALL |

+-------------+-----------------+-----------------+-----------------+-----------------+-----------------+

|[0.00, 0.40)| ks.D=0.019 * | ks.D=0.025 ** | ks.D=0.024 ** | ks.D=0.053 *** | ks.D=0.026 *** |

| | p.05=0.067***,++| p.05=0.072***,++| p.05=0.074***,++| p.05=0.116***,++| p.05=0.081***,++|

| | p.01=0.016***,++| p.01=0.022***,++| p.01=0.021***,++| p.01=0.042***,++| p.01=0.025***,++|

+-------------+-----------------+-----------------+-----------------+-----------------+-----------------+

|[0.40, 1.01)| ks.D=0.012 | ks.D=0.014 | ks.D=0.016 (*) | ks.D=0.051 *** | ks.D=0.017 *** |

| | p.05=0.057 * | p.05=0.056 * | p.05=0.066***,++| p.05=0.107***,++| p.05=0.071***,++|

| | p.01=0.011 | p.01=0.012 | p.01=0.016***,++| p.01=0.035***,++| p.01=0.018***,++|

+-------------+-----------------+-----------------+-----------------+-----------------+-----------------+

|[1.01, 2.58)| ks.D=0.011 | ks.D=0.015 | ks.D=0.019 * | ks.D=0.055 *** | ks.D=0.018 *** |

| | p.05=0.049 0 | p.05=0.053 0 | p.05=0.066***,++| p.05=0.099***,++| p.05=0.066***,++|

| | p.01=0.012 | p.01=0.012 | p.01=0.017***,++| p.01=0.036***,++| p.01=0.019***,++|

+-------------+-----------------+-----------------+-----------------+-----------------+-----------------+

|[2.58,372.79]| ks.D=0.011 | ks.D=0.008 | ks.D=0.021 ** | ks.D=0.049 *** | ks.D=0.018 *** |

| | p.05=0.054 0 | p.05=0.051 0 | p.05=0.070***,++| p.05=0.109***,++| p.05=0.070***,++|

| | p.01=0.010 | p.01=0.012 | p.01=0.016***,++| p.01=0.039***,++| p.01=0.019***,++|

+-------------+-----------------+-----------------+-----------------+-----------------+-----------------+

|ALL | ks.D=0.008 (*) | ks.D=0.010 * | ks.D=0.018 *** | ks.D=0.047 *** | ks.D=0.019 *** |

| | p.05=0.057***,0 | p.05=0.058***,+ | p.05=0.069***,++| p.05=0.108***,++| p.05=0.072***,++|

| | p.01=0.012 **,+ | p.01=0.015***,++| p.01=0.018***,++| p.01=0.038***,++| p.01=0.020***,++|

+-------------+-----------------+-----------------+-----------------+-----------------+-----------------+

$p_{sp}$, $d=meanc$

+-------------+-----------------+-----------------+-----------------+-----------------+-----------------+

| | [0.0, 1.1) | [1.1, 2.1) | [2.1, 3.6) | [3.6,13.9] | ALL |

+-------------+-----------------+-----------------+-----------------+-----------------+-----------------+

|[0.00, 0.40)| ks.D=0.006 | ks.D=0.015 | ks.D=0.015 | ks.D=0.033 *** | ks.D=0.012 ** |

| | p.05=0.051 0 | p.05=0.059 ** | p.05=0.058 ** | p.05=0.076***,++| p.05=0.061***,+ |

| | p.01=0.009 | p.01=0.013 *,+ | p.01=0.013 * | p.01=0.020***,++| p.01=0.014***,++|

+-------------+-----------------+-----------------+-----------------+-----------------+-----------------+

|[0.40, 1.01)| ks.D=0.009 | ks.D=0.014 | ks.D=0.012 | ks.D=0.028 ** | ks.D=0.011 ** |

| | p.05=0.053 0 | p.05=0.054 0 | p.05=0.060 **,+ | p.05=0.067***,++| p.05=0.059***,+ |

| | p.01=0.011 | p.01=0.011 | p.01=0.010 | p.01=0.016***,++| p.01=0.012 ** |

+-------------+-----------------+-----------------+-----------------+-----------------+-----------------+

|[1.01, 2.58)| ks.D=0.011 | ks.D=0.006 | ks.D=0.016 (*) | ks.D=0.026 ** | ks.D=0.008 (*) |

| | p.05=0.051 0 | p.05=0.052 0 | p.05=0.062***,+ | p.05=0.058 ** | p.05=0.056***,0 |

| | p.01=0.009 | p.01=0.012 (*) | p.01=0.014 **,+ | p.01=0.015**,++ | p.01=0.013 **,+ |

+-------------+-----------------+-----------------+-----------------+-----------------+-----------------+

|[2.58,372.79]| ks.D=0.009 | ks.D=0.007 | ks.D=0.008 | ks.D=0.023 ** | ks.D=0.007 |

| | p.05=0.052 0 | p.05=0.053 0 | p.05=0.057 ** | p.05=0.067***,++| p.05=0.057***,0 |

| | p.01=0.009 | p.01=0.010 | p.01=0.016***,++| p.01=0.020***,++| p.01=0.014***,++|

+-------------+-----------------+-----------------+-----------------+-----------------+-----------------+

|ALL | ks.D=0.006 | ks.D=0.009 * | ks.D=0.010 * | ks.D=0.022 *** | ks.D=0.008 *** |

| | p.05=0.052 00 | p.05=0.054 **,0 | p.05=0.059***,+ | p.05=0.067***,++| p.05=0.058***,+ |

| | p.01=0.010 0 | p.01=0.012 * | p.01=0.013***,++| p.01=0.018***,++| p.01=0.013***,++|

+-------------+-----------------+-----------------+-----------------+-----------------+-----------------+

$p_{sp}$, $d=varc$

+-------------+-----------------+-----------------+-----------------+-----------------+-----------------+

| | [0.0, 1.1) | [1.1, 2.1) | [2.1, 3.6) | [3.6,13.9] | ALL |

+-------------+-----------------+-----------------+-----------------+-----------------+-----------------+

|[0.00, 0.40)| ks.D=0.074 *** | ks.D=0.077 *** | ks.D=0.115 *** | ks.D=0.272 *** | ks.D=0.124 *** |

| | p.05=0.133***,++| p.05=0.157***,++| p.05=0.162***,++| p.05=0.272***,++| p.05=0.179***,++|

| | p.01=0.063***,++| p.01=0.077***,++| p.01=0.074***,++| p.01=0.161***,++| p.01=0.092***,++|

+-------------+-----------------+-----------------+-----------------+-----------------+-----------------+

|[0.40, 1.01)| ks.D=0.015 | ks.D=0.053 *** | ks.D=0.119 *** | ks.D=0.327 *** | ks.D=0.123 *** |

| | p.05=0.057 * | p.05=0.066***,++| p.05=0.097***,++| p.05=0.244***,++| p.05=0.114***,++|

| | p.01=0.012 | p.01=0.015**,++ | p.01=0.030***,++| p.01=0.135***,++| p.01=0.047***,++|

+-------------+-----------------+-----------------+-----------------+-----------------+-----------------+

|[1.01, 2.58)| ks.D=0.019 * | ks.D=0.050 *** | ks.D=0.140 *** | ks.D=0.356 *** | ks.D=0.134 *** |

| | p.05=0.051 0 | p.05=0.057 * | p.05=0.089***,++| p.05=0.257***,++| p.05=0.111***,++|

| | p.01=0.010 | p.01=0.013 *,+ | p.01=0.026***,++| p.01=0.143***,++| p.01=0.046***,++|

+-------------+-----------------+-----------------+-----------------+-----------------+-----------------+

|[2.58,372.79]| ks.D=0.011 | ks.D=0.065 *** | ks.D=0.152 *** | ks.D=0.368 *** | ks.D=0.139 *** |

| | p.05=0.046 0 | p.05=0.060 **,+ | p.05=0.091***,++| p.05=0.263***,++| p.05=0.112***,++|

| | p.01=0.008 | p.01=0.011 | p.01=0.027***,++| p.01=0.153***,++| p.01=0.048***,++|

+-------------+-----------------+-----------------+-----------------+-----------------+-----------------+

|ALL | ks.D=0.019 *** | ks.D=0.054 *** | ks.D=0.127 *** | ks.D=0.329 *** | ks.D=0.127 *** |

| | p.05=0.072***,++| p.05=0.086***,++| p.05=0.110***,++| p.05=0.259***,++| p.05=0.129***,++|

| | p.01=0.023***,++| p.01=0.030***,++| p.01=0.039***,++| p.01=0.148***,++| p.01=0.058***,++|

+-------------+-----------------+-----------------+-----------------+-----------------+-----------------+

$p_{sp}$, $d=LL$

+-------------+-----------------+-----------------+-----------------+-----------------+-----------------+

| | [0.0, 1.1) | [1.1, 2.1) | [2.1, 3.6) | [3.6,13.9] | ALL |

+-------------+-----------------+-----------------+-----------------+-----------------+-----------------+

|[0.00, 0.40)| ks.D=0.073 *** | ks.D=0.075 *** | ks.D=0.114 *** | ks.D=0.265 *** | ks.D=0.120 *** |

| | p.05=0.135***,++| p.05=0.157***,++| p.05=0.162***,++| p.05=0.267***,++| p.05=0.178***,++|

| | p.01=0.063***,++| p.01=0.076***,++| p.01=0.075***,++| p.01=0.154***,++| p.01=0.090***,++|

+-------------+-----------------+-----------------+-----------------+-----------------+-----------------+

|[0.40, 1.01)| ks.D=0.014 | ks.D=0.054 *** | ks.D=0.116 *** | ks.D=0.322 *** | ks.D=0.120 *** |

| | p.05=0.058 ** | p.05=0.068***,++| p.05=0.095***,++| p.05=0.236***,++| p.05=0.113***,++|

| | p.01=0.012 (*) | p.01=0.012 (*) | p.01=0.028***,++| p.01=0.128***,++| p.01=0.044***,++|

+-------------+-----------------+-----------------+-----------------+-----------------+-----------------+

|[1.01, 2.58)| ks.D=0.019 * | ks.D=0.048 *** | ks.D=0.136 *** | ks.D=0.352 *** | ks.D=0.131 *** |

| | p.05=0.051 0 | p.05=0.055 (*) | p.05=0.084***,++| p.05=0.250***,++| p.05=0.107***,++|

| | p.01=0.009 | p.01=0.013 * | p.01=0.024***,++| p.01=0.136***,++| p.01=0.044***,++|

+-------------+-----------------+-----------------+-----------------+-----------------+-----------------+

|[2.58,372.79]| ks.D=0.012 | ks.D=0.065 *** | ks.D=0.145 *** | ks.D=0.362 *** | ks.D=0.137 *** |

| | p.05=0.045(*),0 | p.05=0.059 ** | p.05=0.085***,++| p.05=0.259***,++| p.05=0.109***,++|

| | p.01=0.009 | p.01=0.010 | p.01=0.027***,++| p.01=0.145***,++| p.01=0.046***,++|

+-------------+-----------------+-----------------+-----------------+-----------------+-----------------+

|ALL | ks.D=0.018 *** | ks.D=0.051 *** | ks.D=0.123 *** | ks.D=0.323 *** | ks.D=0.124 *** |

| | p.05=0.072***,++| p.05=0.086***,++| p.05=0.106***,++| p.05=0.253***,++| p.05=0.127***,++|

| | p.01=0.024***,++| p.01=0.028***,++| p.01=0.038***,++| p.01=0.141***,++| p.01=0.056***,++|

+-------------+-----------------+-----------------+-----------------+-----------------+-----------------+

## Bernoulli models

$p_{nsp}$, $t=mean$

+-------------+-----------------+-----------------+-----------------+-----------------+-----------------+

| | [0.00,0.07) | [0.07,0.13) | [0.13,0.20) | [0.20,0.25] | ALL |

+-------------+-----------------+-----------------+-----------------+-----------------+-----------------+

|[0.00, 0.40)| ks.D=0.038 *** | ks.D=0.043 *** | ks.D=0.063 *** | ks.D=0.077 *** | ks.D=0.052 *** |

| | p.05=0.096***,++| p.05=0.106***,++| p.05=0.126***,++| p.05=0.167***,++| p.05=0.122***,++|

| | p.01=0.032***,++| p.01=0.038***,++| p.01=0.054***,++| p.01=0.088***,++| p.01=0.052***,++|

+-------------+-----------------+-----------------+-----------------+-----------------+-----------------+

|[0.40, 1.01)| ks.D=0.012 | ks.D=0.017 (*) | ks.D=0.016 (*) | ks.D=0.032 *** | ks.D=0.012 ** |

| | p.05=0.048 0 | p.05=0.058 ** | p.05=0.059 ** | p.05=0.068***,++| p.05=0.058***,+ |

| | p.01=0.011 | p.01=0.012 | p.01=0.012 (*) | p.01=0.016***,++| p.01=0.013***,+ |

+-------------+-----------------+-----------------+-----------------+-----------------+-----------------+

|[1.01, 2.56)| ks.D=0.014 | ks.D=0.007 | ks.D=0.006 | ks.D=0.011 | ks.D=0.005 |

| | p.05=0.054 0 | p.05=0.049 0 | p.05=0.050 0 | p.05=0.056 * | p.05=0.052 00 |

| | p.01=0.011 | p.01=0.011 | p.01=0.010 | p.01=0.012 | p.01=0.011 0 |

+-------------+-----------------+-----------------+-----------------+-----------------+-----------------+

|[2.56,415.82]| ks.D=0.013 | ks.D=0.011 | ks.D=0.015 | ks.D=0.010 | ks.D=0.006 |

| | p.05=0.052 0 | p.05=0.052 0 | p.05=0.051 0 | p.05=0.048 0 | p.05=0.051 00 |

| | p.01=0.010 | p.01=0.010 | p.01=0.012 | p.01=0.008 | p.01=0.010 0 |

+-------------+-----------------+-----------------+-----------------+-----------------+-----------------+

|ALL | ks.D=0.010 * | ks.D=0.013 ** | ks.D=0.018 *** | ks.D=0.029 *** | ks.D=0.016 *** |

| | p.05=0.063***,++| p.05=0.067***,++| p.05=0.072***,++| p.05=0.085***,++| p.05=0.071***,++|

| | p.01=0.016***,++| p.01=0.018***,++| p.01=0.022***,++| p.01=0.031***,++| p.01=0.022***,++|

+-------------+-----------------+-----------------+-----------------+-----------------+-----------------+

$p_{nsp}$, $t=var$

+-------------+-----------------+-----------------+-----------------+-----------------+-----------------+

| | [0.00,0.07) | [0.07,0.13) | [0.13,0.20) | [0.20,0.25] | ALL |

+-------------+-----------------+-----------------+-----------------+-----------------+-----------------+

|[0.00, 0.40)| ks.D=0.053 *** | ks.D=0.043 *** | ks.D=0.037 *** | ks.D=0.033 *** | ks.D=0.038 *** |

| | p.05=0.059 ** | p.05=0.059 ** | p.05=0.063***,+ | p.05=0.058 ** | p.05=0.060***,+ |

| | p.01=0.011 | p.01=0.016***,++| p.01=0.015**,++ | p.01=0.013 * | p.01=0.014***,++|

+-------------+-----------------+-----------------+-----------------+-----------------+-----------------+

|[0.40, 1.01)| ks.D=0.014 | ks.D=0.015 | ks.D=0.008 | ks.D=0.012 | ks.D=0.006 |

| | p.05=0.045(*),0 | p.05=0.052 0 | p.05=0.050 0 | p.05=0.050 0 | p.05=0.049 00 |

| | p.01=0.009 | p.01=0.011 | p.01=0.010 | p.01=0.011 | p.01=0.010 0 |

+-------------+-----------------+-----------------+-----------------+-----------------+-----------------+

|[1.01, 2.56)| ks.D=0.011 | ks.D=0.010 | ks.D=0.022 ** | ks.D=0.015 | ks.D=0.010 * |

| | p.05=0.053 0 | p.05=0.047 0 | p.05=0.044 *,0 | p.05=0.048 0 | p.05=0.048 00 |

| | p.01=0.010 | p.01=0.009 | p.01=0.009 | p.01=0.009 | p.01=0.009 0 |

+-------------+-----------------+-----------------+-----------------+-----------------+-----------------+

|[2.56,415.82]| ks.D=0.021 ** | ks.D=0.013 | ks.D=0.016 (*) | ks.D=0.020 * | ks.D=0.014 *** |

| | p.05=0.048 0 | p.05=0.047 0 | p.05=0.053 0 | p.05=0.052 0 | p.05=0.050 00 |

| | p.01=0.010 | p.01=0.008 (*) | p.01=0.010 | p.01=0.010 | p.01=0.009 0 |

+-------------+-----------------+-----------------+-----------------+-----------------+-----------------+

|ALL | ks.D=0.007 | ks.D=0.012 ** | ks.D=0.004 | ks.D=0.008 | ks.D=0.006 ** |

| | p.05=0.051 00 | p.05=0.051 00 | p.05=0.052(*),00| p.05=0.052 00 | p.05=0.052 *,00 |

| | p.01=0.010 0 | p.01=0.011 | p.01=0.011 | p.01=0.011 0 | p.01=0.011 *,0 |

+-------------+-----------------+-----------------+-----------------+-----------------+-----------------+

$p_{nsp}$, $t=skew$

+-------------+-----------------+-----------------+-----------------+-----------------+-----------------+

| | [0.00,0.07) | [0.07,0.13) | [0.13,0.20) | [0.20,0.25] | ALL |

+-------------+-----------------+-----------------+-----------------+-----------------+-----------------+

|[0.00, 0.40)| ks.D=0.011 | ks.D=0.013 | ks.D=0.020 ** | ks.D=0.038 *** | ks.D=0.016 *** |

| | p.05=0.060 **,+ | p.05=0.061 **,+ | p.05=0.060 **,+ | p.05=0.073***,++| p.05=0.063***,++|

| | p.01=0.014 **,+ | p.01=0.015**,++ | p.01=0.014**,++ | p.01=0.022***,++| p.01=0.016***,++|

+-------------+-----------------+-----------------+-----------------+-----------------+-----------------+

|[0.40, 1.01)| ks.D=0.011 | ks.D=0.009 | ks.D=0.017 * | ks.D=0.012 | ks.D=0.008 |

| | p.05=0.050 0 | p.05=0.055(*),0 | p.05=0.054 0 | p.05=0.054 0 | p.05=0.053 *,0 |

| | p.01=0.010 | p.01=0.011 | p.01=0.010 | p.01=0.012 | p.01=0.011 |

+-------------+-----------------+-----------------+-----------------+-----------------+-----------------+

|[1.01, 2.56)| ks.D=0.012 | ks.D=0.008 | ks.D=0.011 | ks.D=0.010 | ks.D=0.007 |

| | p.05=0.049 0 | p.05=0.050 0 | p.05=0.050 0 | p.05=0.047 0 | p.05=0.049 00 |

| | p.01=0.011 | p.01=0.010 | p.01=0.010 | p.01=0.011 | p.01=0.010 0 |

+-------------+-----------------+-----------------+-----------------+-----------------+-----------------+

|[2.56,415.82]| ks.D=0.011 | ks.D=0.008 | ks.D=0.010 | ks.D=0.009 | ks.D=0.005 |

| | p.05=0.051 0 | p.05=0.046 0 | p.05=0.048 0 | p.05=0.054 0 | p.05=0.050 00 |

| | p.01=0.010 | p.01=0.009 | p.01=0.010 | p.01=0.011 | p.01=0.010 0 |

+-------------+-----------------+-----------------+-----------------+-----------------+-----------------+

|ALL | ks.D=0.008 | ks.D=0.006 | ks.D=0.010 ** | ks.D=0.012 ** | ks.D=0.006 ** |

| | p.05=0.052(*),00| p.05=0.053 *,0 | p.05=0.053 *,0 | p.05=0.057***,0 | p.05=0.054***,00|

| | p.01=0.011 * | p.01=0.011 | p.01=0.011 (*) | p.01=0.014***,++| p.01=0.012***,+ |

+-------------+-----------------+-----------------+-----------------+-----------------+-----------------+

$p_{nsp}$, $t=kurt$

+-------------+-----------------+-----------------+-----------------+-----------------+-----------------+

| | [0.00,0.07) | [0.07,0.13) | [0.13,0.20) | [0.20,0.25] | ALL |

+-------------+-----------------+-----------------+-----------------+-----------------+-----------------+

|[0.00, 0.40)| ks.D=0.040 *** | ks.D=0.033 *** | ks.D=0.040 *** | ks.D=0.038 *** | ks.D=0.036 *** |

| | p.05=0.057 ** | p.05=0.053 0 | p.05=0.052 0 | p.05=0.059 ** | p.05=0.055 **,0 |

| | p.01=0.012 (*) | p.01=0.011 | p.01=0.011 | p.01=0.014 **,+ | p.01=0.012 ** |

+-------------+-----------------+-----------------+-----------------+-----------------+-----------------+

|[0.40, 1.01)| ks.D=0.015 | ks.D=0.006 | ks.D=0.009 | ks.D=0.011 | ks.D=0.004 |

| | p.05=0.052 0 | p.05=0.054 0 | p.05=0.045(*),0 | p.05=0.054 0 | p.05=0.051 00 |

| | p.01=0.010 | p.01=0.009 | p.01=0.010 | p.01=0.013 *,+ | p.01=0.010 0 |

+-------------+-----------------+-----------------+-----------------+-----------------+-----------------+

|[1.01, 2.56)| ks.D=0.009 | ks.D=0.009 | ks.D=0.009 | ks.D=0.008 | ks.D=0.005 |

| | p.05=0.051 0 | p.05=0.049 0 | p.05=0.054 0 | p.05=0.053 0 | p.05=0.052 00 |

| | p.01=0.011 | p.01=0.007 * | p.01=0.009 | p.01=0.012 | p.01=0.010 0 |

+-------------+-----------------+-----------------+-----------------+-----------------+-----------------+

|[2.56,415.82]| ks.D=0.019 * | ks.D=0.009 | ks.D=0.010 | ks.D=0.011 | ks.D=0.009 * |

| | p.05=0.048 0 | p.05=0.049 0 | p.05=0.046 0 | p.05=0.054 0 | p.05=0.049 00 |

| | p.01=0.012 | p.01=0.010 | p.01=0.009 | p.01=0.010 | p.01=0.010 0 |

+-------------+-----------------+-----------------+-----------------+-----------------+-----------------+

|ALL | ks.D=0.011 ** | ks.D=0.012 ** | ks.D=0.010 ** | ks.D=0.012 ** | ks.D=0.009 *** |

| | p.05=0.052 00 | p.05=0.051 00 | p.05=0.050 00 | p.05=0.055 **,0 | p.05=0.052**,00 |

| | p.01=0.011 (*) | p.01=0.009 0 | p.01=0.010 0 | p.01=0.012 ** | p.01=0.011(*),0 |

+-------------+-----------------+-----------------+-----------------+-----------------+-----------------+

$p_{nsp}$, $t=Z_{a}$

+-------------+-----------------+-----------------+-----------------+-----------------+-----------------+

| | [0.00,0.07) | [0.07,0.13) | [0.13,0.20) | [0.20,0.25] | ALL |

+-------------+-----------------+-----------------+-----------------+-----------------+-----------------+

|[0.00, 0.40)| ks.D=0.065 *** | ks.D=0.077 *** | ks.D=0.092 *** | ks.D=0.120 *** | ks.D=0.086 *** |

| | p.05=0.073***,++| p.05=0.077***,++| p.05=0.092***,++| p.05=0.114***,++| p.05=0.088***,++|

| | p.01=0.023***,++| p.01=0.024***,++| p.01=0.032***,++| p.01=0.055***,++| p.01=0.033***,++|

+-------------+-----------------+-----------------+-----------------+-----------------+-----------------+

|[0.40, 1.01)| ks.D=0.011 | ks.D=0.017 (*) | ks.D=0.015 (*) | ks.D=0.034 *** | ks.D=0.015 *** |

| | p.05=0.049 0 | p.05=0.050 0 | p.05=0.054 0 | p.05=0.054 0 | p.05=0.052 00 |

| | p.01=0.009 | p.01=0.011 | p.01=0.011 | p.01=0.014**,++ | p.01=0.011 |

+-------------+-----------------+-----------------+-----------------+-----------------+-----------------+

|[1.01, 2.56)| ks.D=0.015 | ks.D=0.013 | ks.D=0.013 | ks.D=0.019 * | ks.D=0.006 |

| | p.05=0.051 0 | p.05=0.047 0 | p.05=0.047 0 | p.05=0.053 0 | p.05=0.049 00 |

| | p.01=0.010 | p.01=0.008 | p.01=0.008 * | p.01=0.011 | p.01=0.009 0 |

+-------------+-----------------+-----------------+-----------------+-----------------+-----------------+

|[2.56,415.82]| ks.D=0.016 (*) | ks.D=0.014 | ks.D=0.009 | ks.D=0.008 | ks.D=0.008 (*) |

| | p.05=0.054 0 | p.05=0.044 * | p.05=0.052 0 | p.05=0.050 0 | p.05=0.050 00 |

| | p.01=0.011 | p.01=0.009 | p.01=0.011 | p.01=0.008 | p.01=0.010 0 |

+-------------+-----------------+-----------------+-----------------+-----------------+-----------------+

|ALL | ks.D=0.016 *** | ks.D=0.023 *** | ks.D=0.024 *** | ks.D=0.042 *** | ks.D=0.024 *** |

| | p.05=0.057***,0 | p.05=0.055 **,0 | p.05=0.061***,+ | p.05=0.068***,++| p.05=0.060***,+ |

| | p.01=0.013***,++| p.01=0.013***,+ | p.01=0.015***,++| p.01=0.022***,++| p.01=0.016***,++|

+-------------+-----------------+-----------------+-----------------+-----------------+-----------------+

$p_{sp}$, $t=mean$

+-------------+-----------------+-----------------+-----------------+-----------------+-----------------+

| | [0.00,0.07) | [0.07,0.13) | [0.13,0.20) | [0.20,0.25] | ALL |

+-------------+-----------------+-----------------+-----------------+-----------------+-----------------+

|[0.00, 0.40)| ks.D=0.051 *** | ks.D=0.063 *** | ks.D=0.086 *** | ks.D=0.109 *** | ks.D=0.072 *** |

| | p.05=0.123***,++| p.05=0.138***,++| p.05=0.166***,++| p.05=0.222***,++| p.05=0.160***,++|

| | p.01=0.051***,++| p.01=0.060***,++| p.01=0.083***,++| p.01=0.127***,++| p.01=0.079***,++|

+-------------+-----------------+-----------------+-----------------+-----------------+-----------------+

|[0.40, 1.01)| ks.D=0.012 | ks.D=0.020 * | ks.D=0.022 ** | ks.D=0.037 *** | ks.D=0.016 *** |

| | p.05=0.051 0 | p.05=0.062***,+ | p.05=0.068***,++| p.05=0.086***,++| p.05=0.066***,++|

| | p.01=0.010 | p.01=0.014**,++ | p.01=0.014 **,+ | p.01=0.026***,++| p.01=0.016***,++|

+-------------+-----------------+-----------------+-----------------+-----------------+-----------------+

|[1.01, 2.56)| ks.D=0.009 | ks.D=0.006 | ks.D=0.015 | ks.D=0.017 (*) | ks.D=0.008 |

| | p.05=0.053 0 | p.05=0.051 0 | p.05=0.050 0 | p.05=0.057 * | p.05=0.052(*),00|

| | p.01=0.012 | p.01=0.011 | p.01=0.010 | p.01=0.013 * | p.01=0.011 * |

+-------------+-----------------+-----------------+-----------------+-----------------+-----------------+

|[2.56,415.82]| ks.D=0.016 (*) | ks.D=0.008 | ks.D=0.008 | ks.D=0.011 | ks.D=0.006 |

| | p.05=0.046 0 | p.05=0.053 0 | p.05=0.050 0 | p.05=0.052 0 | p.05=0.050 00 |

| | p.01=0.009 | p.01=0.010 | p.01=0.009 | p.01=0.010 | p.01=0.010 0 |

+-------------+-----------------+-----------------+-----------------+-----------------+-----------------+

|ALL | ks.D=0.014 ** | ks.D=0.020 *** | ks.D=0.027 *** | ks.D=0.037 *** | ks.D=0.023 *** |

| | p.05=0.069***,++| p.05=0.076***,++| p.05=0.084***,++| p.05=0.105***,++| p.05=0.083***,++|

| | p.01=0.021***,++| p.01=0.024***,++| p.01=0.029***,++| p.01=0.044***,++| p.01=0.029***,++|

+-------------+-----------------+-----------------+-----------------+-----------------+-----------------+

$p_{sp}$, $t=var$

+-------------+-----------------+-----------------+-----------------+-----------------+-----------------+

| | [0.00,0.07) | [0.07,0.13) | [0.13,0.20) | [0.20,0.25] | ALL |

+-------------+-----------------+-----------------+-----------------+-----------------+-----------------+

|[0.00, 0.40)| ks.D=0.142 *** | ks.D=0.131 *** | ks.D=0.104 *** | ks.D=0.103 *** | ks.D=0.115 *** |

| | p.05=0.117***,++| p.05=0.121***,++| p.05=0.136***,++| p.05=0.168***,++| p.05=0.135***,++|

| | p.01=0.049***,++| p.01=0.052***,++| p.01=0.059***,++| p.01=0.081***,++| p.01=0.059***,++|

+-------------+-----------------+-----------------+-----------------+-----------------+-----------------+

|[0.40, 1.01)| ks.D=0.026 ** | ks.D=0.032 *** | ks.D=0.025 ** | ks.D=0.024 ** | ks.D=0.022 *** |

| | p.05=0.051 0 | p.05=0.059 ** | p.05=0.060 **,+ | p.05=0.076***,++| p.05=0.061***,+ |

| | p.01=0.011 | p.01=0.013 *,+ | p.01=0.010 | p.01=0.021***,++| p.01=0.014***,++|

+-------------+-----------------+-----------------+-----------------+-----------------+-----------------+

|[1.01, 2.56)| ks.D=0.016 (*) | ks.D=0.026 ** | ks.D=0.029 *** | ks.D=0.036 *** | ks.D=0.024 *** |

| | p.05=0.052 0 | p.05=0.049 0 | p.05=0.048 0 | p.05=0.055 (*) | p.05=0.051 00 |

| | p.01=0.011 | p.01=0.011 | p.01=0.009 | p.01=0.011 | p.01=0.010 0 |

+-------------+-----------------+-----------------+-----------------+-----------------+-----------------+

|[2.56,415.82]| ks.D=0.039 *** | ks.D=0.041 *** | ks.D=0.037 *** | ks.D=0.046 *** | ks.D=0.038 *** |

| | p.05=0.046 0 | p.05=0.050 0 | p.05=0.049 0 | p.05=0.049 0 | p.05=0.049 00 |

| | p.01=0.009 | p.01=0.009 | p.01=0.011 | p.01=0.010 | p.01=0.010 0 |

+-------------+-----------------+-----------------+-----------------+-----------------+-----------------+

|ALL | ks.D=0.031 *** | ks.D=0.030 *** | ks.D=0.027 *** | ks.D=0.034 *** | ks.D=0.027 *** |

| | p.05=0.067***,++| p.05=0.070***,++| p.05=0.074***,++| p.05=0.087***,++| p.05=0.074***,++|

| | p.01=0.020***,++| p.01=0.022***,++| p.01=0.022***,++| p.01=0.031***,++| p.01=0.024***,++|

+-------------+-----------------+-----------------+-----------------+-----------------+-----------------+

$p_{sp}$, $t=p_{0}$

+-------------+-----------------+-----------------+-----------------+-----------------+-----------------+

| | [0.00,0.07) | [0.07,0.13) | [0.13,0.20) | [0.20,0.25] | ALL |

+-------------+-----------------+-----------------+-----------------+-----------------+-----------------+

|[0.00, 0.40)| ks.D=0.052 *** | ks.D=0.062 *** | ks.D=0.086 *** | ks.D=0.107 *** | ks.D=0.072 *** |

| | p.05=0.124***,++| p.05=0.137***,++| p.05=0.166***,++| p.05=0.223***,++| p.05=0.161***,++|

| | p.01=0.053***,++| p.01=0.062***,++| p.01=0.083***,++| p.01=0.128***,++| p.01=0.080***,++|

+-------------+-----------------+-----------------+-----------------+-----------------+-----------------+

|[0.40, 1.01)| ks.D=0.011 | ks.D=0.020 * | ks.D=0.023 ** | ks.D=0.037 *** | ks.D=0.017 *** |

| | p.05=0.049 0 | p.05=0.062***,+ | p.05=0.065***,++| p.05=0.083***,++| p.05=0.064***,++|

| | p.01=0.011 | p.01=0.014 **,+ | p.01=0.013 **,+ | p.01=0.024***,++| p.01=0.015***,++|

+-------------+-----------------+-----------------+-----------------+-----------------+-----------------+

|[1.01, 2.56)| ks.D=0.008 | ks.D=0.006 | ks.D=0.013 | ks.D=0.018 (*) | ks.D=0.006 |

| | p.05=0.052 0 | p.05=0.052 0 | p.05=0.052 0 | p.05=0.059 ** | p.05=0.053 *,0 |

| | p.01=0.011 | p.01=0.010 | p.01=0.012 | p.01=0.012 (*) | p.01=0.011 * |

+-------------+-----------------+-----------------+-----------------+-----------------+-----------------+

|[2.56,415.82]| ks.D=0.016 (*) | ks.D=0.007 | ks.D=0.008 | ks.D=0.011 | ks.D=0.007 |

| | p.05=0.047 0 | p.05=0.050 0 | p.05=0.050 0 | p.05=0.053 0 | p.05=0.050 00 |

| | p.01=0.008 (*) | p.01=0.009 | p.01=0.011 | p.01=0.011 | p.01=0.009 0 |

+-------------+-----------------+-----------------+-----------------+-----------------+-----------------+

|ALL | ks.D=0.014 *** | ks.D=0.020 *** | ks.D=0.027 *** | ks.D=0.036 *** | ks.D=0.023 *** |

| | p.05=0.069***,++| p.05=0.076***,++| p.05=0.084***,++| p.05=0.105***,++| p.05=0.083***,++|

| | p.01=0.021***,++| p.01=0.024***,++| p.01=0.030***,++| p.01=0.044***,++| p.01=0.029***,++|

+-------------+-----------------+-----------------+-----------------+-----------------+-----------------+

$p_{sp}$, $d=meanc$

+-------------+-----------------+-----------------+-----------------+-----------------+-----------------+

| | [0.00,0.07) | [0.07,0.13) | [0.13,0.20) | [0.20,0.25] | ALL |

+-------------+-----------------+-----------------+-----------------+-----------------+-----------------+

|[0.00, 0.40)| ks.D=0.051 *** | ks.D=0.063 *** | ks.D=0.086 *** | ks.D=0.109 *** | ks.D=0.072 *** |

| | p.05=0.123***,++| p.05=0.138***,++| p.05=0.166***,++| p.05=0.222***,++| p.05=0.160***,++|

| | p.01=0.051***,++| p.01=0.060***,++| p.01=0.083***,++| p.01=0.127***,++| p.01=0.079***,++|

+-------------+-----------------+-----------------+-----------------+-----------------+-----------------+

|[0.40, 1.01)| ks.D=0.012 | ks.D=0.020 * | ks.D=0.022 ** | ks.D=0.037 *** | ks.D=0.016 *** |

| | p.05=0.051 0 | p.05=0.062***,+ | p.05=0.068***,++| p.05=0.086***,++| p.05=0.066***,++|

| | p.01=0.010 | p.01=0.014**,++ | p.01=0.014 **,+ | p.01=0.026***,++| p.01=0.016***,++|

+-------------+-----------------+-----------------+-----------------+-----------------+-----------------+

|[1.01, 2.56)| ks.D=0.009 | ks.D=0.006 | ks.D=0.015 | ks.D=0.017 (*) | ks.D=0.008 |

| | p.05=0.053 0 | p.05=0.051 0 | p.05=0.050 0 | p.05=0.057 * | p.05=0.052(*),00|

| | p.01=0.012 | p.01=0.011 | p.01=0.010 | p.01=0.013 * | p.01=0.011 * |

+-------------+-----------------+-----------------+-----------------+-----------------+-----------------+

|[2.56,415.82]| ks.D=0.016 (*) | ks.D=0.008 | ks.D=0.008 | ks.D=0.011 | ks.D=0.006 |

| | p.05=0.046 0 | p.05=0.053 0 | p.05=0.050 0 | p.05=0.052 0 | p.05=0.050 00 |

| | p.01=0.009 | p.01=0.010 | p.01=0.009 | p.01=0.010 | p.01=0.010 0 |

+-------------+-----------------+-----------------+-----------------+-----------------+-----------------+

|ALL | ks.D=0.014 ** | ks.D=0.020 *** | ks.D=0.027 *** | ks.D=0.037 *** | ks.D=0.023 *** |

| | p.05=0.069***,++| p.05=0.076***,++| p.05=0.084***,++| p.05=0.105***,++| p.05=0.083***,++|

| | p.01=0.021***,++| p.01=0.024***,++| p.01=0.029***,++| p.01=0.044***,++| p.01=0.029***,++|

+-------------+-----------------+-----------------+-----------------+-----------------+-----------------+

$p_{sp}$, $d=varc$

+-------------+-----------------+-----------------+-----------------+-----------------+-----------------+

| | [0.00,0.07) | [0.07,0.13) | [0.13,0.20) | [0.20,0.25] | ALL |

+-------------+-----------------+-----------------+-----------------+-----------------+-----------------+

|[0.00, 0.40)| ks.D=0.142 *** | ks.D=0.131 *** | ks.D=0.104 *** | ks.D=0.103 *** | ks.D=0.115 *** |

| | p.05=0.117***,++| p.05=0.121***,++| p.05=0.137***,++| p.05=0.168***,++| p.05=0.135***,++|

| | p.01=0.049***,++| p.01=0.052***,++| p.01=0.059***,++| p.01=0.081***,++| p.01=0.059***,++|

+-------------+-----------------+-----------------+-----------------+-----------------+-----------------+

|[0.40, 1.01)| ks.D=0.026 ** | ks.D=0.032 *** | ks.D=0.025 ** | ks.D=0.024 ** | ks.D=0.022 *** |

| | p.05=0.051 0 | p.05=0.059 ** | p.05=0.060 **,+ | p.05=0.076***,++| p.05=0.061***,+ |

| | p.01=0.011 | p.01=0.013 * | p.01=0.010 | p.01=0.021***,++| p.01=0.014***,++|

+-------------+-----------------+-----------------+-----------------+-----------------+-----------------+

|[1.01, 2.56)| ks.D=0.016 (*) | ks.D=0.026 ** | ks.D=0.029 *** | ks.D=0.036 *** | ks.D=0.024 *** |

| | p.05=0.052 0 | p.05=0.049 0 | p.05=0.048 0 | p.05=0.055 (*) | p.05=0.051 00 |

| | p.01=0.011 | p.01=0.011 | p.01=0.009 | p.01=0.011 | p.01=0.010 0 |

+-------------+-----------------+-----------------+-----------------+-----------------+-----------------+

|[2.56,415.82]| ks.D=0.039 *** | ks.D=0.041 *** | ks.D=0.037 *** | ks.D=0.046 *** | ks.D=0.038 *** |

| | p.05=0.046 0 | p.05=0.050 0 | p.05=0.049 0 | p.05=0.049 0 | p.05=0.049 00 |

| | p.01=0.009 | p.01=0.009 | p.01=0.011 | p.01=0.010 | p.01=0.010 0 |

+-------------+-----------------+-----------------+-----------------+-----------------+-----------------+

|ALL | ks.D=0.031 *** | ks.D=0.030 *** | ks.D=0.027 *** | ks.D=0.034 *** | ks.D=0.027 *** |

| | p.05=0.067***,++| p.05=0.070***,++| p.05=0.074***,++| p.05=0.088***,++| p.05=0.074***,++|

| | p.01=0.020***,++| p.01=0.022***,++| p.01=0.022***,++| p.01=0.031***,++| p.01=0.024***,++|

+-------------+-----------------+-----------------+-----------------+-----------------+-----------------+

$p_{sp}$, $d=LL$

+-------------+-----------------+-----------------+-----------------+-----------------+-----------------+

| | [0.00,0.07) | [0.07,0.13) | [0.13,0.20) | [0.20,0.25] | ALL |

+-------------+-----------------+-----------------+-----------------+-----------------+-----------------+

|[0.00, 0.40)| ks.D=0.136 *** | ks.D=0.114 *** | ks.D=0.084 *** | ks.D=0.136 *** | ks.D=0.096 *** |

| | p.05=0.123***,++| p.05=0.139***,++| p.05=0.167***,++| p.05=0.224***,++| p.05=0.161***,++|

| | p.01=0.052***,++| p.01=0.062***,++| p.01=0.083***,++| p.01=0.126***,++| p.01=0.079***,++|

+-------------+-----------------+-----------------+-----------------+-----------------+-----------------+

|[0.40, 1.01)| ks.D=0.026 ** | ks.D=0.028 ** | ks.D=0.023 ** | ks.D=0.044 *** | ks.D=0.018 *** |

| | p.05=0.052 0 | p.05=0.062***,+ | p.05=0.067***,++| p.05=0.084***,++| p.05=0.066***,++|

| | p.01=0.011 | p.01=0.015**,++ | p.01=0.014 **,+ | p.01=0.026***,++| p.01=0.016***,++|

+-------------+-----------------+-----------------+-----------------+-----------------+-----------------+

|[1.01, 2.56)| ks.D=0.018 * | ks.D=0.026 ** | ks.D=0.033 *** | ks.D=0.041 *** | ks.D=0.027 *** |

| | p.05=0.052 0 | p.05=0.050 0 | p.05=0.051 0 | p.05=0.058 ** | p.05=0.053(*),0 |

| | p.01=0.012 | p.01=0.011 | p.01=0.011 | p.01=0.014 *,+ | p.01=0.012 * |

+-------------+-----------------+-----------------+-----------------+-----------------+-----------------+

|[2.56,415.82]| ks.D=0.039 *** | ks.D=0.040 *** | ks.D=0.038 *** | ks.D=0.044 *** | ks.D=0.038 *** |

| | p.05=0.044 *,0 | p.05=0.052 0 | p.05=0.049 0 | p.05=0.050 0 | p.05=0.049 00 |

| | p.01=0.008 | p.01=0.010 | p.01=0.009 | p.01=0.011 | p.01=0.009 0 |

+-------------+-----------------+-----------------+-----------------+-----------------+-----------------+

|ALL | ks.D=0.029 *** | ks.D=0.025 *** | ks.D=0.031 *** | ks.D=0.060 *** | ks.D=0.023 *** |

| | p.05=0.068***,++| p.05=0.076***,++| p.05=0.084***,++| p.05=0.105***,++| p.05=0.083***,++|

| | p.01=0.021***,++| p.01=0.024***,++| p.01=0.029***,++| p.01=0.045***,++| p.01=0.029***,++|

+-------------+-----------------+-----------------+-----------------+-----------------+-----------------+
