# Supplementary material for: A New Calibrated Bayesian Internal Goodness-of-Fit Method: Sampled Posterior p-Values as Simple and General p-Values That Allow Double Use of the Data
Source: PLoS One. 2011 Mar 18;6(3):e14770. doi: 10.1371/journal.pone.0014770 (PMC3060804; doi:10.1371/journal.pone.0014770)
Supplement: Text S4 — Results of Scenario 4. (0.40 MB DOC) [file pone.0014770.s004.doc]

New Calibrated Bayesian Internal Goodness-of-Fit Methods: Sampled Posterior P-values as Simple and General P-values that Allow Double Use of the Data

Frédéric Gosselin

Cemagref, UR EFNO, F-45290 Nogent-sur-Vernisson, France

E-mail: [frederic.gosselin@cemagref.fr](mailto:frederic.gosselin@cemagref.fr)

*Results of Scenario 4*

# Scenario 4 $p_{sp}$ and $p_{nsp}$ results

*Scenario 4*. The setting is the same as in Scenario 1, except that data were generated from fixed parameters, chosen at the mean of their statistical prior under Scenario 1, i.e. $\lambda=\text{exp}\left(1\right)$ in the Poisson case, $\sigma=1$ and $\theta=0$ in the Gaussian case and $\theta=0.5$ in the Bernoulli case.

Simulations were performed on $p_{sp}$ and $p_{nsp}$. In the following tables, we display the Kolmogorow-Smirnov statistic of the comparison of the p-values with a uniform distribution (ks.D), the proportion of values in the 5% extreme positions on the interval [0;1] (p.05), and the same for 1% (p.01), according to the interval to which $\rho_{0}$ (respectively $1/\rho_{0}$) for Poisson and Normal models (resp. Bernoulli models) (in rows) and the sample size *n* (in columns) belonged. Remember that here $\rho_{0}$ is the random parameter that characterizes the non-informativeness of the statistical prior for the Poisson and Normal cases and the informativeness for the Bernoulli case. 100,000 data set samples were considered. The notation for the significance of the tests is the same as in Supplementary Text S1.

## Poisson models

$p_{nsp}$, $t=mean$

+-----------+-----------------+-----------------+-----------------+-----------------+-----------------+

| | [ 20, 80) | [ 80, 300) | [300, 600) | [600,1000] | ALL |

+-----------+-----------------+-----------------+-----------------+-----------------+-----------------+

|[0.05,0.56)| ks.D=0.018 * | ks.D=0.017 (*) | ks.D=0.023 ** | ks.D=0.011 | ks.D=0.014 ** |

| | p.05=0.046(*),0 | p.05=0.047 0 | p.05=0.046 0 | p.05=0.046 0 | p.05=0.046 **,0 |

| | p.01=0.010 | p.01=0.011 | p.01=0.008 | p.01=0.009 | p.01=0.010 0 |

+-----------+-----------------+-----------------+-----------------+-----------------+-----------------+

|[0.56,1.06)| ks.D=0.024 ** | ks.D=0.019 * | ks.D=0.012 | ks.D=0.012 | ks.D=0.014 ** |

| | p.05=0.047 0 | p.05=0.046 0 | p.05=0.048 0 | p.05=0.049 0 | p.05=0.048(*),00|

| | p.01=0.008 (*) | p.01=0.009 | p.01=0.008 | p.01=0.009 | p.01=0.008 * |

+-----------+-----------------+-----------------+-----------------+-----------------+-----------------+

|[1.06,1.56)| ks.D=0.029 *** | ks.D=0.014 | ks.D=0.013 | ks.D=0.010 | ks.D=0.010 * |

| | p.05=0.047 0 | p.05=0.050 0 | p.05=0.050 0 | p.05=0.046 0 | p.05=0.048 00 |

| | p.01=0.009 | p.01=0.008 | p.01=0.010 | p.01=0.008 | p.01=0.009(*),0 |

+-----------+-----------------+-----------------+-----------------+-----------------+-----------------+

|[1.56,2.05]| ks.D=0.019 * | ks.D=0.012 | ks.D=0.009 | ks.D=0.008 | ks.D=0.008 |

| | p.05=0.050 0 | p.05=0.052 0 | p.05=0.049 0 | p.05=0.047 0 | p.05=0.049 00 |

| | p.01=0.013 * | p.01=0.013 * | p.01=0.009 | p.01=0.009 | p.01=0.011 |

+-----------+-----------------+-----------------+-----------------+-----------------+-----------------+

|ALL | ks.D=0.021 *** | ks.D=0.010 * | ks.D=0.010 ** | ks.D=0.006 | ks.D=0.010 *** |

| | p.05=0.047(*),00| p.05=0.049 00 | p.05=0.048 00 | p.05=0.047(*),00| p.05=0.048**,00 |

| | p.01=0.010 0 | p.01=0.010 0 | p.01=0.009 * | p.01=0.009 | p.01=0.009(*),0 |

+-----------+-----------------+-----------------+-----------------+-----------------+-----------------+

$p_{nsp}$, $t=var$

+-----------+-----------------+-----------------+-----------------+-----------------+-----------------+

| | [ 20, 80) | [ 80, 300) | [300, 600) | [600,1000] | ALL |

+-----------+-----------------+-----------------+-----------------+-----------------+-----------------+

|[0.05,0.56)| ks.D=0.007 | ks.D=0.010 | ks.D=0.013 | ks.D=0.011 | ks.D=0.005 |

| | p.05=0.051 0 | p.05=0.049 0 | p.05=0.048 0 | p.05=0.048 0 | p.05=0.049 00 |

| | p.01=0.009 | p.01=0.010 | p.01=0.010 | p.01=0.009 | p.01=0.009 0 |

+-----------+-----------------+-----------------+-----------------+-----------------+-----------------+

|[0.56,1.06)| ks.D=0.014 | ks.D=0.010 | ks.D=0.015 (*) | ks.D=0.020 * | ks.D=0.006 |

| | p.05=0.052 0 | p.05=0.052 0 | p.05=0.048 0 | p.05=0.045(*),0 | p.05=0.050 00 |

| | p.01=0.011 | p.01=0.009 | p.01=0.010 | p.01=0.008 | p.01=0.010 0 |

+-----------+-----------------+-----------------+-----------------+-----------------+-----------------+

|[1.06,1.56)| ks.D=0.018 * | ks.D=0.006 | ks.D=0.007 | ks.D=0.007 | ks.D=0.007 |

| | p.05=0.050 0 | p.05=0.044 *,0 | p.05=0.049 0 | p.05=0.046 0 | p.05=0.048(*),00|

| | p.01=0.011 | p.01=0.009 | p.01=0.009 | p.01=0.009 | p.01=0.009 0 |

+-----------+-----------------+-----------------+-----------------+-----------------+-----------------+

|[1.56,2.05]| ks.D=0.019 * | ks.D=0.007 | ks.D=0.010 | ks.D=0.016 | ks.D=0.009 * |

| | p.05=0.050 0 | p.05=0.051 0 | p.05=0.049 0 | p.05=0.051 0 | p.05=0.050 00 |

| | p.01=0.010 | p.01=0.011 | p.01=0.009 | p.01=0.011 | p.01=0.010 0 |

+-----------+-----------------+-----------------+-----------------+-----------------+-----------------+

|ALL | ks.D=0.012 ** | ks.D=0.004 | ks.D=0.008 (*) | ks.D=0.007 | ks.D=0.004 (*) |

| | p.05=0.051 00 | p.05=0.049 00 | p.05=0.049 00 | p.05=0.047(*),0 | p.05=0.049 00 |

| | p.01=0.010 0 | p.01=0.010 0 | p.01=0.009 0 | p.01=0.009 0 | p.01=0.010 00 |

+-----------+-----------------+-----------------+-----------------+-----------------+-----------------+

$p_{nsp}$, $t=skew$

+-----------+-----------------+-----------------+-----------------+-----------------+-----------------+

| | [ 20, 80) | [ 80, 300) | [300, 600) | [600,1000] | ALL |

+-----------+-----------------+-----------------+-----------------+-----------------+-----------------+

|[0.05,0.56)| ks.D=0.014 | ks.D=0.009 | ks.D=0.011 | ks.D=0.013 | ks.D=0.003 |

| | p.05=0.053 0 | p.05=0.048 0 | p.05=0.046 0 | p.05=0.052 0 | p.05=0.050 00 |

| | p.01=0.011 | p.01=0.011 | p.01=0.009 | p.01=0.012 | p.01=0.010 0 |

+-----------+-----------------+-----------------+-----------------+-----------------+-----------------+

|[0.56,1.06)| ks.D=0.010 | ks.D=0.009 | ks.D=0.005 | ks.D=0.016 | ks.D=0.005 |

| | p.05=0.050 0 | p.05=0.056 * | p.05=0.050 0 | p.05=0.045(*),0 | p.05=0.050 00 |

| | p.01=0.009 | p.01=0.012 | p.01=0.011 | p.01=0.008 | p.01=0.010 0 |

+-----------+-----------------+-----------------+-----------------+-----------------+-----------------+

|[1.06,1.56)| ks.D=0.013 | ks.D=0.008 | ks.D=0.011 | ks.D=0.013 | ks.D=0.004 |

| | p.05=0.053 0 | p.05=0.046 0 | p.05=0.051 0 | p.05=0.051 0 | p.05=0.050 00 |

| | p.01=0.012 (*) | p.01=0.008 (*) | p.01=0.011 | p.01=0.009 | p.01=0.010 0 |

+-----------+-----------------+-----------------+-----------------+-----------------+-----------------+

|[1.56,2.05]| ks.D=0.013 | ks.D=0.017 (*) | ks.D=0.013 | ks.D=0.019 * | ks.D=0.010 * |

| | p.05=0.050 0 | p.05=0.046 0 | p.05=0.051 0 | p.05=0.048 0 | p.05=0.049 00 |

| | p.01=0.009 | p.01=0.011 | p.01=0.010 | p.01=0.011 | p.01=0.010 0 |

+-----------+-----------------+-----------------+-----------------+-----------------+-----------------+

|ALL | ks.D=0.007 | ks.D=0.005 | ks.D=0.004 | ks.D=0.009 (*) | ks.D=0.004 (*) |

| | p.05=0.052 00 | p.05=0.049 00 | p.05=0.050 00 | p.05=0.049 00 | p.05=0.050 00 |

| | p.01=0.010 0 | p.01=0.010 0 | p.01=0.010 0 | p.01=0.010 0 | p.01=0.010 00 |

+-----------+-----------------+-----------------+-----------------+-----------------+-----------------+

$p_{nsp}$, $t=kurt$

+-----------+-----------------+-----------------+-----------------+-----------------+-----------------+

| | [ 20, 80) | [ 80, 300) | [300, 600) | [600,1000] | ALL |

+-----------+-----------------+-----------------+-----------------+-----------------+-----------------+

|[0.05,0.56)| ks.D=0.009 | ks.D=0.007 | ks.D=0.011 | ks.D=0.018 (*) | ks.D=0.004 |

| | p.05=0.052 0 | p.05=0.049 0 | p.05=0.051 0 | p.05=0.053 0 | p.05=0.051 00 |

| | p.01=0.010 | p.01=0.012 | p.01=0.008 | p.01=0.011 | p.01=0.010 0 |

+-----------+-----------------+-----------------+-----------------+-----------------+-----------------+

|[0.56,1.06)| ks.D=0.017 * | ks.D=0.006 | ks.D=0.012 | ks.D=0.016 | ks.D=0.008 (*) |

| | p.05=0.049 0 | p.05=0.052 0 | p.05=0.047 0 | p.05=0.043 * | p.05=0.048 00 |

| | p.01=0.011 | p.01=0.011 | p.01=0.009 | p.01=0.010 | p.01=0.010 0 |

+-----------+-----------------+-----------------+-----------------+-----------------+-----------------+

|[1.06,1.56)| ks.D=0.013 | ks.D=0.012 | ks.D=0.008 | ks.D=0.006 | ks.D=0.007 |

| | p.05=0.055 * | p.05=0.048 0 | p.05=0.057 ** | p.05=0.053 0 | p.05=0.054 **,0 |

| | p.01=0.011 | p.01=0.010 | p.01=0.012 | p.01=0.010 | p.01=0.011 0 |

+-----------+-----------------+-----------------+-----------------+-----------------+-----------------+

|[1.56,2.05]| ks.D=0.009 | ks.D=0.009 | ks.D=0.011 | ks.D=0.016 | ks.D=0.004 |

| | p.05=0.053 0 | p.05=0.044 *,0 | p.05=0.045 *,0 | p.05=0.050 0 | p.05=0.048 00 |

| | p.01=0.010 | p.01=0.010 | p.01=0.009 | p.01=0.009 | p.01=0.009 0 |

+-----------+-----------------+-----------------+-----------------+-----------------+-----------------+

|ALL | ks.D=0.006 | ks.D=0.004 | ks.D=0.003 | ks.D=0.008 | ks.D=0.003 |

| | p.05=0.052 00 | p.05=0.049 00 | p.05=0.050 00 | p.05=0.050 00 | p.05=0.050 00 |

| | p.01=0.010 0 | p.01=0.011 0 | p.01=0.009 0 | p.01=0.010 0 | p.01=0.010 00 |

+-----------+-----------------+-----------------+-----------------+-----------------+-----------------+

$p_{nsp}$, $t=Z_{a}$

+-----------+-----------------+-----------------+-----------------+-----------------+-----------------+

| | [ 20, 80) | [ 80, 300) | [300, 600) | [600,1000] | ALL |

+-----------+-----------------+-----------------+-----------------+-----------------+-----------------+

|[0.05,0.56)| ks.D=0.020 ** | ks.D=0.015 | ks.D=0.017 * | ks.D=0.011 | ks.D=0.013 ** |

| | p.05=0.051 0 | p.05=0.055 0 | p.05=0.050 0 | p.05=0.047 0 | p.05=0.051 00 |

| | p.01=0.010 | p.01=0.011 | p.01=0.010 | p.01=0.010 | p.01=0.010 0 |

+-----------+-----------------+-----------------+-----------------+-----------------+-----------------+

|[0.56,1.06)| ks.D=0.020 ** | ks.D=0.009 | ks.D=0.014 | ks.D=0.013 | ks.D=0.006 |

| | p.05=0.051 0 | p.05=0.046 0 | p.05=0.051 0 | p.05=0.048 0 | p.05=0.049 00 |

| | p.01=0.010 | p.01=0.010 | p.01=0.010 | p.01=0.007 * | p.01=0.010 0 |

+-----------+-----------------+-----------------+-----------------+-----------------+-----------------+

|[1.06,1.56)| ks.D=0.008 | ks.D=0.010 | ks.D=0.012 | ks.D=0.011 | ks.D=0.004 |

| | p.05=0.054 0 | p.05=0.050 0 | p.05=0.049 0 | p.05=0.049 0 | p.05=0.051 00 |

| | p.01=0.012 (*) | p.01=0.010 | p.01=0.011 | p.01=0.010 | p.01=0.011 |

+-----------+-----------------+-----------------+-----------------+-----------------+-----------------+

|[1.56,2.05]| ks.D=0.010 | ks.D=0.008 | ks.D=0.006 | ks.D=0.007 | ks.D=0.004 |

| | p.05=0.049 0 | p.05=0.053 0 | p.05=0.050 0 | p.05=0.049 0 | p.05=0.050 00 |

| | p.01=0.010 | p.01=0.011 | p.01=0.009 | p.01=0.010 | p.01=0.010 0 |

+-----------+-----------------+-----------------+-----------------+-----------------+-----------------+

|ALL | ks.D=0.009 * | ks.D=0.007 | ks.D=0.004 | ks.D=0.005 | ks.D=0.005 * |

| | p.05=0.051 00 | p.05=0.051 00 | p.05=0.050 00 | p.05=0.048 00 | p.05=0.050 00 |

| | p.01=0.011 0 | p.01=0.011 0 | p.01=0.010 0 | p.01=0.009 0 | p.01=0.010 00 |

+-----------+-----------------+-----------------+-----------------+-----------------+-----------------+

$p_{sp}$, $t=mean$

+-----------+-----------------+-----------------+-----------------+-----------------+-----------------+

| | [ 20, 80) | [ 80, 300) | [300, 600) | [600,1000] | ALL |

+-----------+-----------------+-----------------+-----------------+-----------------+-----------------+

|[0.05,0.56)| ks.D=0.017 * | ks.D=0.017 (*) | ks.D=0.025 ** | ks.D=0.009 | ks.D=0.013 ** |

| | p.05=0.042 ** | p.05=0.044 *,0 | p.05=0.045(*),0 | p.05=0.047 0 | p.05=0.044***,0 |

| | p.01=0.010 | p.01=0.009 | p.01=0.010 | p.01=0.008 | p.01=0.009 0 |

+-----------+-----------------+-----------------+-----------------+-----------------+-----------------+

|[0.56,1.06)| ks.D=0.027 ** | ks.D=0.023 ** | ks.D=0.013 | ks.D=0.012 | ks.D=0.015 *** |

| | p.05=0.046(*),0 | p.05=0.045(*),0 | p.05=0.047 0 | p.05=0.050 0 | p.05=0.047 *,0 |

| | p.01=0.008 | p.01=0.007 * | p.01=0.009 | p.01=0.009 | p.01=0.008 ** |

+-----------+-----------------+-----------------+-----------------+-----------------+-----------------+

|[1.06,1.56)| ks.D=0.033 *** | ks.D=0.014 | ks.D=0.011 | ks.D=0.010 | ks.D=0.011 ** |

| | p.05=0.049 0 | p.05=0.048 0 | p.05=0.050 0 | p.05=0.047 0 | p.05=0.049 00 |

| | p.01=0.010 | p.01=0.010 | p.01=0.010 | p.01=0.009 | p.01=0.010 0 |

+-----------+-----------------+-----------------+-----------------+-----------------+-----------------+

|[1.56,2.05]| ks.D=0.022 ** | ks.D=0.010 | ks.D=0.008 | ks.D=0.009 | ks.D=0.009 * |

| | p.05=0.051 0 | p.05=0.053 0 | p.05=0.050 0 | p.05=0.049 0 | p.05=0.051 00 |

| | p.01=0.013 * | p.01=0.012 | p.01=0.008 | p.01=0.009 | p.01=0.011 0 |

+-----------+-----------------+-----------------+-----------------+-----------------+-----------------+

|ALL | ks.D=0.022 *** | ks.D=0.012 ** | ks.D=0.008 (*) | ks.D=0.006 | ks.D=0.010 *** |

| | p.05=0.047 *,0 | p.05=0.048(*),00| p.05=0.048 00 | p.05=0.048 00 | p.05=0.048**,00 |

| | p.01=0.010 0 | p.01=0.009 0 | p.01=0.009 0 | p.01=0.009 (*) | p.01=0.009(*),0 |

+-----------+-----------------+-----------------+-----------------+-----------------+-----------------+

$p_{sp}$, $t=var$

+-----------+-----------------+-----------------+-----------------+-----------------+-----------------+

| | [ 20, 80) | [ 80, 300) | [300, 600) | [600,1000] | ALL |

+-----------+-----------------+-----------------+-----------------+-----------------+-----------------+

|[0.05,0.56)| ks.D=0.013 | ks.D=0.015 | ks.D=0.007 | ks.D=0.010 | ks.D=0.006 |

| | p.05=0.044 *,0 | p.05=0.048 0 | p.05=0.048 0 | p.05=0.052 0 | p.05=0.048(*),00|

| | p.01=0.009 | p.01=0.011 | p.01=0.007 **,- | p.01=0.010 | p.01=0.009(*),0 |

+-----------+-----------------+-----------------+-----------------+-----------------+-----------------+

|[0.56,1.06)| ks.D=0.022 ** | ks.D=0.012 | ks.D=0.013 | ks.D=0.030 ** | ks.D=0.012 ** |

| | p.05=0.054 0 | p.05=0.049 0 | p.05=0.051 0 | p.05=0.052 0 | p.05=0.051 00 |

| | p.01=0.009 | p.01=0.009 | p.01=0.010 | p.01=0.008 (*) | p.01=0.009 0 |

+-----------+-----------------+-----------------+-----------------+-----------------+-----------------+

|[1.06,1.56)| ks.D=0.030 *** | ks.D=0.011 | ks.D=0.012 | ks.D=0.011 | ks.D=0.014 *** |

| | p.05=0.047 0 | p.05=0.047 0 | p.05=0.050 0 | p.05=0.048 0 | p.05=0.048 00 |

| | p.01=0.010 | p.01=0.008 | p.01=0.010 | p.01=0.010 | p.01=0.010 0 |

+-----------+-----------------+-----------------+-----------------+-----------------+-----------------+

|[1.56,2.05]| ks.D=0.022 ** | ks.D=0.014 | ks.D=0.008 | ks.D=0.012 | ks.D=0.010 ** |

| | p.05=0.052 0 | p.05=0.049 0 | p.05=0.049 0 | p.05=0.047 0 | p.05=0.049 00 |

| | p.01=0.010 | p.01=0.010 | p.01=0.009 | p.01=0.007 (*) | p.01=0.009 0 |

+-----------+-----------------+-----------------+-----------------+-----------------+-----------------+

|ALL | ks.D=0.021 *** | ks.D=0.010 * | ks.D=0.004 | ks.D=0.010 * | ks.D=0.009 *** |

| | p.05=0.049 00 | p.05=0.048 00 | p.05=0.049 00 | p.05=0.050 00 | p.05=0.049 00 |

| | p.01=0.010 0 | p.01=0.010 0 | p.01=0.009(*),0 | p.01=0.009 * | p.01=0.009 *,0 |

+-----------+-----------------+-----------------+-----------------+-----------------+-----------------+

$p_{sp}$, $t=p_{0}$

+-----------+-----------------+-----------------+-----------------+-----------------+-----------------+

| | [ 20, 80) | [ 80, 300) | [300, 600) | [600,1000] | ALL |

+-----------+-----------------+-----------------+-----------------+-----------------+-----------------+

|[0.05,0.56)| ks.D=0.028 *** | ks.D=0.023 ** | ks.D=0.020 ** | ks.D=0.013 | ks.D=0.017 *** |

| | p.05=0.049 0 | p.05=0.045(*),0 | p.05=0.046 0 | p.05=0.049 0 | p.05=0.047(*),00|

| | p.01=0.010 | p.01=0.009 | p.01=0.011 | p.01=0.011 | p.01=0.010 0 |

+-----------+-----------------+-----------------+-----------------+-----------------+-----------------+

|[0.56,1.06)| ks.D=0.024 ** | ks.D=0.016 (*) | ks.D=0.018 * | ks.D=0.016 | ks.D=0.017 *** |

| | p.05=0.050 0 | p.05=0.047 0 | p.05=0.050 0 | p.05=0.053 0 | p.05=0.050 00 |

| | p.01=0.010 | p.01=0.009 | p.01=0.009 | p.01=0.012 | p.01=0.010 0 |

+-----------+-----------------+-----------------+-----------------+-----------------+-----------------+

|[1.06,1.56)| ks.D=0.031 *** | ks.D=0.016 (*) | ks.D=0.010 | ks.D=0.016 | ks.D=0.014 *** |

| | p.05=0.058 ** | p.05=0.053 0 | p.05=0.052 0 | p.05=0.048 0 | p.05=0.053 *,0 |

| | p.01=0.013 **,+ | p.01=0.009 | p.01=0.011 | p.01=0.010 | p.01=0.011 0 |

+-----------+-----------------+-----------------+-----------------+-----------------+-----------------+

|[1.56,2.05]| ks.D=0.025 ** | ks.D=0.021 * | ks.D=0.011 | ks.D=0.013 | ks.D=0.013 ** |

| | p.05=0.058 ** | p.05=0.048 0 | p.05=0.056 * | p.05=0.051 0 | p.05=0.054 *,0 |

| | p.01=0.012 (*) | p.01=0.009 | p.01=0.011 | p.01=0.010 | p.01=0.011 0 |

+-----------+-----------------+-----------------+-----------------+-----------------+-----------------+

|ALL | ks.D=0.022 *** | ks.D=0.013 ** | ks.D=0.011 ** | ks.D=0.012 ** | ks.D=0.014 *** |

| | p.05=0.054 **,0 | p.05=0.048 00 | p.05=0.051 00 | p.05=0.050 00 | p.05=0.051 00 |

| | p.01=0.011 * | p.01=0.009 0 | p.01=0.010 0 | p.01=0.011 0 | p.01=0.010 00 |

+-----------+-----------------+-----------------+-----------------+-----------------+-----------------+

$p_{sp}$, $d=meanc$

+-----------+-----------------+-----------------+-----------------+-----------------+-----------------+

| | [ 20, 80) | [ 80, 300) | [300, 600) | [600,1000] | ALL |

+-----------+-----------------+-----------------+-----------------+-----------------+-----------------+

|[0.05,0.56)| ks.D=0.017 * | ks.D=0.017 (*) | ks.D=0.025 ** | ks.D=0.009 | ks.D=0.013 ** |

| | p.05=0.042 ** | p.05=0.044 *,0 | p.05=0.045(*),0 | p.05=0.047 0 | p.05=0.044***,0 |

| | p.01=0.010 | p.01=0.009 | p.01=0.010 | p.01=0.008 | p.01=0.009 0 |

+-----------+-----------------+-----------------+-----------------+-----------------+-----------------+

|[0.56,1.06)| ks.D=0.027 ** | ks.D=0.023 ** | ks.D=0.013 | ks.D=0.012 | ks.D=0.015 *** |

| | p.05=0.046(*),0 | p.05=0.045(*),0 | p.05=0.047 0 | p.05=0.050 0 | p.05=0.047 *,0 |

| | p.01=0.008 | p.01=0.007 * | p.01=0.009 | p.01=0.009 | p.01=0.008 ** |

+-----------+-----------------+-----------------+-----------------+-----------------+-----------------+

|[1.06,1.56)| ks.D=0.033 *** | ks.D=0.014 | ks.D=0.011 | ks.D=0.010 | ks.D=0.011 ** |

| | p.05=0.049 0 | p.05=0.048 0 | p.05=0.050 0 | p.05=0.047 0 | p.05=0.049 00 |

| | p.01=0.010 | p.01=0.010 | p.01=0.010 | p.01=0.009 | p.01=0.010 0 |

+-----------+-----------------+-----------------+-----------------+-----------------+-----------------+

|[1.56,2.05]| ks.D=0.022 ** | ks.D=0.010 | ks.D=0.008 | ks.D=0.009 | ks.D=0.009 * |

| | p.05=0.051 0 | p.05=0.053 0 | p.05=0.050 0 | p.05=0.049 0 | p.05=0.051 00 |

| | p.01=0.013 * | p.01=0.012 | p.01=0.008 | p.01=0.009 | p.01=0.011 0 |

+-----------+-----------------+-----------------+-----------------+-----------------+-----------------+

|ALL | ks.D=0.022 *** | ks.D=0.012 ** | ks.D=0.008 (*) | ks.D=0.006 | ks.D=0.010 *** |

| | p.05=0.047 *,0 | p.05=0.048(*),00| p.05=0.048 00 | p.05=0.048 00 | p.05=0.048**,00 |

| | p.01=0.010 0 | p.01=0.009 0 | p.01=0.009 0 | p.01=0.009 (*) | p.01=0.009(*),0 |

+-----------+-----------------+-----------------+-----------------+-----------------+-----------------+

$p_{sp}$, $d=varc$

+-----------+-----------------+-----------------+-----------------+-----------------+-----------------+

| | [ 20, 80) | [ 80, 300) | [300, 600) | [600,1000] | ALL |

+-----------+-----------------+-----------------+-----------------+-----------------+-----------------+

|[0.05,0.56)| ks.D=0.013 | ks.D=0.015 | ks.D=0.007 | ks.D=0.010 | ks.D=0.006 |

| | p.05=0.045 *,0 | p.05=0.048 0 | p.05=0.048 0 | p.05=0.052 0 | p.05=0.048(*),00|

| | p.01=0.009 | p.01=0.011 | p.01=0.007 **,- | p.01=0.010 | p.01=0.009(*),0 |

+-----------+-----------------+-----------------+-----------------+-----------------+-----------------+

|[0.56,1.06)| ks.D=0.023 ** | ks.D=0.012 | ks.D=0.013 | ks.D=0.030 ** | ks.D=0.012 ** |

| | p.05=0.054 0 | p.05=0.049 0 | p.05=0.051 0 | p.05=0.052 0 | p.05=0.051 00 |

| | p.01=0.009 | p.01=0.009 | p.01=0.010 | p.01=0.008 (*) | p.01=0.009 0 |

+-----------+-----------------+-----------------+-----------------+-----------------+-----------------+

|[1.06,1.56)| ks.D=0.030 *** | ks.D=0.012 | ks.D=0.012 | ks.D=0.011 | ks.D=0.014 *** |

| | p.05=0.047 0 | p.05=0.047 0 | p.05=0.050 0 | p.05=0.048 0 | p.05=0.048 00 |

| | p.01=0.010 | p.01=0.008 | p.01=0.010 | p.01=0.010 | p.01=0.010 0 |

+-----------+-----------------+-----------------+-----------------+-----------------+-----------------+

|[1.56,2.05]| ks.D=0.022 ** | ks.D=0.013 | ks.D=0.008 | ks.D=0.012 | ks.D=0.010 ** |

| | p.05=0.051 0 | p.05=0.049 0 | p.05=0.049 0 | p.05=0.047 0 | p.05=0.049 00 |

| | p.01=0.010 | p.01=0.010 | p.01=0.009 | p.01=0.007 (*) | p.01=0.009 0 |

+-----------+-----------------+-----------------+-----------------+-----------------+-----------------+

|ALL | ks.D=0.021 *** | ks.D=0.010 * | ks.D=0.004 | ks.D=0.010 * | ks.D=0.009 *** |

| | p.05=0.049 00 | p.05=0.048 00 | p.05=0.049 00 | p.05=0.050 00 | p.05=0.049 00 |

| | p.01=0.010 0 | p.01=0.010 0 | p.01=0.009(*),0 | p.01=0.009 * | p.01=0.009 *,0 |

+-----------+-----------------+-----------------+-----------------+-----------------+-----------------+

$p_{sp}$, $d=LL$

+-----------+-----------------+-----------------+-----------------+-----------------+-----------------+

| | [ 20, 80) | [ 80, 300) | [300, 600) | [600,1000] | ALL |

+-----------+-----------------+-----------------+-----------------+-----------------+-----------------+

|[0.05,0.56)| ks.D=0.019 * | ks.D=0.017 * | ks.D=0.009 | ks.D=0.011 | ks.D=0.011 ** |

| | p.05=0.045(*),0 | p.05=0.046 0 | p.05=0.046 0 | p.05=0.050 0 | p.05=0.047 *,0 |

| | p.01=0.009 | p.01=0.009 | p.01=0.008 (*) | p.01=0.010 | p.01=0.009 (*) |

+-----------+-----------------+-----------------+-----------------+-----------------+-----------------+

|[0.56,1.06)| ks.D=0.028 *** | ks.D=0.014 | ks.D=0.009 | ks.D=0.026 ** | ks.D=0.013 ** |

| | p.05=0.053 0 | p.05=0.051 0 | p.05=0.052 0 | p.05=0.052 0 | p.05=0.052 00 |

| | p.01=0.010 | p.01=0.008 * | p.01=0.011 | p.01=0.008 (*) | p.01=0.009 0 |

+-----------+-----------------+-----------------+-----------------+-----------------+-----------------+

|[1.06,1.56)| ks.D=0.043 *** | ks.D=0.013 | ks.D=0.015 (*) | ks.D=0.013 | ks.D=0.017 *** |

| | p.05=0.052 0 | p.05=0.049 0 | p.05=0.047 0 | p.05=0.051 0 | p.05=0.050 00 |

| | p.01=0.011 | p.01=0.009 | p.01=0.009 | p.01=0.009 | p.01=0.009 0 |

+-----------+-----------------+-----------------+-----------------+-----------------+-----------------+

|[1.56,2.05]| ks.D=0.031 *** | ks.D=0.015 | ks.D=0.011 | ks.D=0.012 | ks.D=0.014 ** |

| | p.05=0.053 0 | p.05=0.051 0 | p.05=0.050 0 | p.05=0.050 0 | p.05=0.051 00 |

| | p.01=0.010 | p.01=0.012 | p.01=0.010 | p.01=0.007 (*) | p.01=0.010 0 |

+-----------+-----------------+-----------------+-----------------+-----------------+-----------------+

|ALL | ks.D=0.028 *** | ks.D=0.012 ** | ks.D=0.006 | ks.D=0.010 * | ks.D=0.013 *** |

| | p.05=0.051 00 | p.05=0.049 00 | p.05=0.049 00 | p.05=0.051 00 | p.05=0.050 00 |

| | p.01=0.010 0 | p.01=0.009 0 | p.01=0.010 0 | p.01=0.008 * | p.01=0.009 *,0 |

+-----------+-----------------+-----------------+-----------------+-----------------+-----------------+

## Normal models

$p_{nsp}$, $t=mean$

+-----------+-----------------+-----------------+-----------------+-----------------+-----------------+

| | [ 20, 80) | [ 80, 300) | [300, 600) | [600,1000] | ALL |

+-----------+-----------------+-----------------+-----------------+-----------------+-----------------+

|[0.10,0.19)| ks.D=0.014 | ks.D=0.009 | ks.D=0.008 | ks.D=0.011 | ks.D=0.005 |

| | p.05=0.048 0 | p.05=0.050 0 | p.05=0.050 0 | p.05=0.054 0 | p.05=0.050 00 |

| | p.01=0.010 | p.01=0.011 | p.01=0.011 | p.01=0.010 | p.01=0.010 0 |

+-----------+-----------------+-----------------+-----------------+-----------------+-----------------+

|[0.19,0.33)| ks.D=0.013 | ks.D=0.017 (*) | ks.D=0.005 | ks.D=0.009 | ks.D=0.004 |

| | p.05=0.046 0 | p.05=0.046 0 | p.05=0.050 0 | p.05=0.050 0 | p.05=0.048 00 |

| | p.01=0.009 | p.01=0.009 | p.01=0.010 | p.01=0.011 | p.01=0.010 0 |

+-----------+-----------------+-----------------+-----------------+-----------------+-----------------+

|[0.33,0.57)| ks.D=0.012 | ks.D=0.010 | ks.D=0.014 | ks.D=0.024 ** | ks.D=0.006 |

| | p.05=0.049 0 | p.05=0.048 0 | p.05=0.050 0 | p.05=0.050 0 | p.05=0.049 00 |

| | p.01=0.010 | p.01=0.009 | p.01=0.011 | p.01=0.009 | p.01=0.010 0 |

+-----------+-----------------+-----------------+-----------------+-----------------+-----------------+

|[0.57,1.00]| ks.D=0.009 | ks.D=0.008 | ks.D=0.012 | ks.D=0.010 | ks.D=0.004 |

| | p.05=0.051 0 | p.05=0.051 0 | p.05=0.050 0 | p.05=0.047 0 | p.05=0.050 00 |

| | p.01=0.011 | p.01=0.012 | p.01=0.010 | p.01=0.009 | p.01=0.010 0 |

+-----------+-----------------+-----------------+-----------------+-----------------+-----------------+

|ALL | ks.D=0.005 | ks.D=0.008 | ks.D=0.006 | ks.D=0.005 | ks.D=0.002 |

| | p.05=0.048 00 | p.05=0.049 00 | p.05=0.050 00 | p.05=0.050 00 | p.05=0.049 00 |

| | p.01=0.010 0 | p.01=0.010 0 | p.01=0.010 0 | p.01=0.009 0 | p.01=0.010 00 |

+-----------+-----------------+-----------------+-----------------+-----------------+-----------------+

$p_{nsp}$, $t=var$

+-----------+-----------------+-----------------+-----------------+-----------------+-----------------+

| | [ 20, 80) | [ 80, 300) | [300, 600) | [600,1000] | ALL |

+-----------+-----------------+-----------------+-----------------+-----------------+-----------------+

|[0.10,0.19)| ks.D=0.043 *** | ks.D=0.024 ** | ks.D=0.019 ** | ks.D=0.015 | ks.D=0.024 *** |

| | p.05=0.032***,--| p.05=0.040 **,- | p.05=0.048 0 | p.05=0.045(*),0 | p.05=0.041***,- |

| | p.01=0.004***,--| p.01=0.007 **,- | p.01=0.010 | p.01=0.010 | p.01=0.008***,- |

+-----------+-----------------+-----------------+-----------------+-----------------+-----------------+

|[0.19,0.33)| ks.D=0.046 *** | ks.D=0.036 *** | ks.D=0.016 (*) | ks.D=0.022 * | ks.D=0.027 *** |

| | p.05=0.030***,--| p.05=0.041 ** | p.05=0.045(*),0 | p.05=0.053 0 | p.05=0.042***,- |

| | p.01=0.004***,--| p.01=0.008 | p.01=0.009 | p.01=0.011 | p.01=0.008 **,- |

+-----------+-----------------+-----------------+-----------------+-----------------+-----------------+

|[0.33,0.57)| ks.D=0.042 *** | ks.D=0.024 ** | ks.D=0.019 * | ks.D=0.013 | ks.D=0.022 *** |

| | p.05=0.038***,- | p.05=0.050 0 | p.05=0.047 0 | p.05=0.051 0 | p.05=0.046 **,0 |

| | p.01=0.007 **,- | p.01=0.007 **,- | p.01=0.010 | p.01=0.009 | p.01=0.008 ** |

+-----------+-----------------+-----------------+-----------------+-----------------+-----------------+

|[0.57,1.00]| ks.D=0.046 *** | ks.D=0.035 *** | ks.D=0.019 * | ks.D=0.015 | ks.D=0.026 *** |

| | p.05=0.043 ** | p.05=0.050 0 | p.05=0.048 0 | p.05=0.050 0 | p.05=0.048(*),00|

| | p.01=0.009 | p.01=0.011 | p.01=0.010 | p.01=0.010 | p.01=0.010 0 |

+-----------+-----------------+-----------------+-----------------+-----------------+-----------------+

|ALL | ks.D=0.041 *** | ks.D=0.028 *** | ks.D=0.016 *** | ks.D=0.013 ** | ks.D=0.024 *** |

| | p.05=0.036***,--| p.05=0.045 **,0 | p.05=0.047 *,00 | p.05=0.050 00 | p.05=0.044***,0 |

| | p.01=0.006***,--| p.01=0.008 ** | p.01=0.010 0 | p.01=0.010 0 | p.01=0.008***,- |

+-----------+-----------------+-----------------+-----------------+-----------------+-----------------+

$p_{nsp}$, $t=skew$

+-----------+-----------------+-----------------+-----------------+-----------------+-----------------+

| | [ 20, 80) | [ 80, 300) | [300, 600) | [600,1000] | ALL |

+-----------+-----------------+-----------------+-----------------+-----------------+-----------------+

|[0.10,0.19)| ks.D=0.007 | ks.D=0.007 | ks.D=0.007 | ks.D=0.010 | ks.D=0.004 |

| | p.05=0.048 0 | p.05=0.052 0 | p.05=0.049 0 | p.05=0.052 0 | p.05=0.050 00 |

| | p.01=0.009 | p.01=0.011 | p.01=0.010 | p.01=0.012 | p.01=0.010 0 |

+-----------+-----------------+-----------------+-----------------+-----------------+-----------------+

|[0.19,0.33)| ks.D=0.011 | ks.D=0.005 | ks.D=0.013 | ks.D=0.008 | ks.D=0.006 |

| | p.05=0.050 0 | p.05=0.050 0 | p.05=0.048 0 | p.05=0.045(*),0 | p.05=0.048 00 |

| | p.01=0.012 | p.01=0.008 | p.01=0.010 | p.01=0.011 | p.01=0.010 0 |

+-----------+-----------------+-----------------+-----------------+-----------------+-----------------+

|[0.33,0.57)| ks.D=0.010 | ks.D=0.014 | ks.D=0.014 | ks.D=0.012 | ks.D=0.005 |

| | p.05=0.052 0 | p.05=0.055(*),0 | p.05=0.052 0 | p.05=0.045 0 | p.05=0.051 00 |

| | p.01=0.010 | p.01=0.011 | p.01=0.009 | p.01=0.009 | p.01=0.010 0 |

+-----------+-----------------+-----------------+-----------------+-----------------+-----------------+

|[0.57,1.00]| ks.D=0.008 | ks.D=0.011 | ks.D=0.008 | ks.D=0.009 | ks.D=0.004 |

| | p.05=0.053 0 | p.05=0.055 (*) | p.05=0.052 0 | p.05=0.051 0 | p.05=0.053 *,0 |

| | p.01=0.013 * | p.01=0.010 | p.01=0.010 | p.01=0.012 (*) | p.01=0.011 * |

+-----------+-----------------+-----------------+-----------------+-----------------+-----------------+

|ALL | ks.D=0.003 | ks.D=0.006 | ks.D=0.004 | ks.D=0.006 | ks.D=0.002 |

| | p.05=0.051 00 | p.05=0.053 *,0 | p.05=0.050 00 | p.05=0.048 00 | p.05=0.051 00 |

| | p.01=0.011 0 | p.01=0.010 0 | p.01=0.010 0 | p.01=0.011 | p.01=0.010 00 |

+-----------+-----------------+-----------------+-----------------+-----------------+-----------------+

$p_{nsp}$, $t=kurt$

+-----------+-----------------+-----------------+-----------------+-----------------+-----------------+

| | [ 20, 80) | [ 80, 300) | [300, 600) | [600,1000] | ALL |

+-----------+-----------------+-----------------+-----------------+-----------------+-----------------+

|[0.10,0.19)| ks.D=0.008 | ks.D=0.016 (*) | ks.D=0.016 (*) | ks.D=0.017 (*) | ks.D=0.004 |

| | p.05=0.047 0 | p.05=0.058 ** | p.05=0.048 0 | p.05=0.051 0 | p.05=0.051 00 |

| | p.01=0.009 | p.01=0.011 | p.01=0.011 | p.01=0.009 | p.01=0.010 0 |

+-----------+-----------------+-----------------+-----------------+-----------------+-----------------+

|[0.19,0.33)| ks.D=0.015 | ks.D=0.012 | ks.D=0.011 | ks.D=0.011 | ks.D=0.007 |

| | p.05=0.047 0 | p.05=0.052 0 | p.05=0.048 0 | p.05=0.053 0 | p.05=0.050 00 |

| | p.01=0.010 | p.01=0.010 | p.01=0.010 | p.01=0.009 | p.01=0.010 0 |

+-----------+-----------------+-----------------+-----------------+-----------------+-----------------+

|[0.33,0.57)| ks.D=0.007 | ks.D=0.013 | ks.D=0.011 | ks.D=0.018 (*) | ks.D=0.009 * |

| | p.05=0.045(*),0 | p.05=0.051 0 | p.05=0.048 0 | p.05=0.051 0 | p.05=0.049 00 |

| | p.01=0.008 | p.01=0.011 | p.01=0.008 | p.01=0.008 | p.01=0.009 (*) |

+-----------+-----------------+-----------------+-----------------+-----------------+-----------------+

|[0.57,1.00]| ks.D=0.014 | ks.D=0.017 (*) | ks.D=0.012 | ks.D=0.006 | ks.D=0.010 * |

| | p.05=0.053 0 | p.05=0.047 0 | p.05=0.046 0 | p.05=0.050 0 | p.05=0.049 00 |

| | p.01=0.011 | p.01=0.011 | p.01=0.009 | p.01=0.010 | p.01=0.010 0 |

+-----------+-----------------+-----------------+-----------------+-----------------+-----------------+

|ALL | ks.D=0.007 | ks.D=0.009 * | ks.D=0.003 | ks.D=0.006 | ks.D=0.004 |

| | p.05=0.048 00 | p.05=0.052 00 | p.05=0.048(*),00| p.05=0.051 00 | p.05=0.050 00 |

| | p.01=0.010 0 | p.01=0.011 0 | p.01=0.009 0 | p.01=0.009 0 | p.01=0.010 00 |

+-----------+-----------------+-----------------+-----------------+-----------------+-----------------+

$p_{nsp}$, $t=Z_{a}$

+-----------+-----------------+-----------------+-----------------+-----------------+-----------------+

| | [ 20, 80) | [ 80, 300) | [300, 600) | [600,1000] | ALL |

+-----------+-----------------+-----------------+-----------------+-----------------+-----------------+

|[0.10,0.19)| ks.D=0.038 *** | ks.D=0.019 * | ks.D=0.016 * | ks.D=0.017 (*) | ks.D=0.018 *** |

| | p.05=0.044 *,0 | p.05=0.049 0 | p.05=0.054 0 | p.05=0.057 * | p.05=0.051 00 |

| | p.01=0.010 | p.01=0.009 | p.01=0.009 | p.01=0.009 | p.01=0.009 0 |

+-----------+-----------------+-----------------+-----------------+-----------------+-----------------+

|[0.19,0.33)| ks.D=0.040 *** | ks.D=0.023 ** | ks.D=0.014 | ks.D=0.011 | ks.D=0.018 *** |

| | p.05=0.047 0 | p.05=0.046 0 | p.05=0.048 0 | p.05=0.050 0 | p.05=0.048 00 |

| | p.01=0.010 | p.01=0.007 * | p.01=0.009 | p.01=0.011 | p.01=0.009 0 |

+-----------+-----------------+-----------------+-----------------+-----------------+-----------------+

|[0.33,0.57)| ks.D=0.032 *** | ks.D=0.015 | ks.D=0.010 | ks.D=0.010 | ks.D=0.012 ** |

| | p.05=0.048 0 | p.05=0.051 0 | p.05=0.043 * | p.05=0.046 0 | p.05=0.047(*),00|

| | p.01=0.009 | p.01=0.011 | p.01=0.010 | p.01=0.010 | p.01=0.010 0 |

+-----------+-----------------+-----------------+-----------------+-----------------+-----------------+

|[0.57,1.00]| ks.D=0.017 * | ks.D=0.017 (*) | ks.D=0.010 | ks.D=0.011 | ks.D=0.010 * |

| | p.05=0.047 0 | p.05=0.046 0 | p.05=0.046 0 | p.05=0.054 0 | p.05=0.048 00 |

| | p.01=0.009 | p.01=0.009 | p.01=0.011 | p.01=0.013 (*) | p.01=0.011 0 |

+-----------+-----------------+-----------------+-----------------+-----------------+-----------------+

|ALL | ks.D=0.029 *** | ks.D=0.014 *** | ks.D=0.009 * | ks.D=0.008 | ks.D=0.013 *** |

| | p.05=0.047 *,0 | p.05=0.048 00 | p.05=0.048 00 | p.05=0.052 00 | p.05=0.049 *,00 |

| | p.01=0.010 0 | p.01=0.009 0 | p.01=0.010 0 | p.01=0.011 0 | p.01=0.010 00 |

+-----------+-----------------+-----------------+-----------------+-----------------+-----------------+

$p_{sp}$, $t=mean$

+-----------+-----------------+-----------------+-----------------+-----------------+-----------------+

| | [ 20, 80) | [ 80, 300) | [300, 600) | [600,1000] | ALL |

+-----------+-----------------+-----------------+-----------------+-----------------+-----------------+

|[0.10,0.19)| ks.D=0.014 | ks.D=0.009 | ks.D=0.008 | ks.D=0.011 | ks.D=0.005 |

| | p.05=0.048 0 | p.05=0.050 0 | p.05=0.050 0 | p.05=0.054 0 | p.05=0.050 00 |

| | p.01=0.010 | p.01=0.011 | p.01=0.011 | p.01=0.010 | p.01=0.010 0 |

+-----------+-----------------+-----------------+-----------------+-----------------+-----------------+

|[0.19,0.33)| ks.D=0.013 | ks.D=0.017 (*) | ks.D=0.005 | ks.D=0.009 | ks.D=0.004 |

| | p.05=0.046 0 | p.05=0.046 0 | p.05=0.050 0 | p.05=0.050 0 | p.05=0.048 00 |

| | p.01=0.009 | p.01=0.009 | p.01=0.010 | p.01=0.011 | p.01=0.010 0 |

+-----------+-----------------+-----------------+-----------------+-----------------+-----------------+

|[0.33,0.57)| ks.D=0.012 | ks.D=0.010 | ks.D=0.014 | ks.D=0.024 ** | ks.D=0.006 |

| | p.05=0.049 0 | p.05=0.048 0 | p.05=0.050 0 | p.05=0.050 0 | p.05=0.049 00 |

| | p.01=0.010 | p.01=0.009 | p.01=0.011 | p.01=0.009 | p.01=0.010 0 |

+-----------+-----------------+-----------------+-----------------+-----------------+-----------------+

|[0.57,1.00]| ks.D=0.009 | ks.D=0.008 | ks.D=0.012 | ks.D=0.010 | ks.D=0.004 |

| | p.05=0.051 0 | p.05=0.051 0 | p.05=0.050 0 | p.05=0.047 0 | p.05=0.050 00 |

| | p.01=0.011 | p.01=0.012 | p.01=0.010 | p.01=0.009 | p.01=0.010 0 |

+-----------+-----------------+-----------------+-----------------+-----------------+-----------------+

|ALL | ks.D=0.005 | ks.D=0.008 | ks.D=0.006 | ks.D=0.005 | ks.D=0.002 |

| | p.05=0.048 00 | p.05=0.049 00 | p.05=0.050 00 | p.05=0.050 00 | p.05=0.049 00 |

| | p.01=0.010 0 | p.01=0.010 0 | p.01=0.010 0 | p.01=0.009 0 | p.01=0.010 00 |

+-----------+-----------------+-----------------+-----------------+-----------------+-----------------+

$p_{sp}$, $t=var$

+-----------+-----------------+-----------------+-----------------+-----------------+-----------------+

| | [ 20, 80) | [ 80, 300) | [300, 600) | [600,1000] | ALL |

+-----------+-----------------+-----------------+-----------------+-----------------+-----------------+

|[0.10,0.19)| ks.D=0.043 *** | ks.D=0.024 ** | ks.D=0.019 ** | ks.D=0.015 | ks.D=0.024 *** |

| | p.05=0.032***,--| p.05=0.040 **,- | p.05=0.048 0 | p.05=0.045(*),0 | p.05=0.041***,- |

| | p.01=0.004***,--| p.01=0.007 *,- | p.01=0.010 | p.01=0.010 | p.01=0.008***,- |

+-----------+-----------------+-----------------+-----------------+-----------------+-----------------+

|[0.19,0.33)| ks.D=0.046 *** | ks.D=0.036 *** | ks.D=0.016 (*) | ks.D=0.022 * | ks.D=0.027 *** |

| | p.05=0.030***,--| p.05=0.041 ** | p.05=0.045(*),0 | p.05=0.053 0 | p.05=0.042***,- |

| | p.01=0.004***,--| p.01=0.008 | p.01=0.009 | p.01=0.011 | p.01=0.008 **,- |

+-----------+-----------------+-----------------+-----------------+-----------------+-----------------+

|[0.33,0.57)| ks.D=0.042 *** | ks.D=0.024 ** | ks.D=0.019 * | ks.D=0.013 | ks.D=0.022 *** |

| | p.05=0.038***,- | p.05=0.050 0 | p.05=0.047 0 | p.05=0.051 0 | p.05=0.046 **,0 |

| | p.01=0.007 **,- | p.01=0.007 **,- | p.01=0.010 | p.01=0.009 | p.01=0.008 ** |

+-----------+-----------------+-----------------+-----------------+-----------------+-----------------+

|[0.57,1.00]| ks.D=0.046 *** | ks.D=0.035 *** | ks.D=0.019 * | ks.D=0.015 | ks.D=0.026 *** |

| | p.05=0.043 ** | p.05=0.050 0 | p.05=0.048 0 | p.05=0.050 0 | p.05=0.047(*),00|

| | p.01=0.009 | p.01=0.011 | p.01=0.010 | p.01=0.010 | p.01=0.010 0 |

+-----------+-----------------+-----------------+-----------------+-----------------+-----------------+

|ALL | ks.D=0.041 *** | ks.D=0.028 *** | ks.D=0.016 *** | ks.D=0.013 ** | ks.D=0.024 *** |

| | p.05=0.036***,--| p.05=0.045 **,0 | p.05=0.047 *,00 | p.05=0.050 00 | p.05=0.044***,0 |

| | p.01=0.006***,--| p.01=0.008 ** | p.01=0.010 0 | p.01=0.010 0 | p.01=0.008***,- |

+-----------+-----------------+-----------------+-----------------+-----------------+-----------------+

$p_{sp}$, $t=p_{0}$

+-----------+-----------------+-----------------+-----------------+-----------------+-----------------+

| | [ 20, 80) | [ 80, 300) | [300, 600) | [600,1000] | ALL |

+-----------+-----------------+-----------------+-----------------+-----------------+-----------------+

|[0.10,0.19)| ks.D=0.015 (*) | ks.D=0.012 | ks.D=0.008 | ks.D=0.015 | ks.D=0.004 |

| | p.05=0.049 0 | p.05=0.049 0 | p.05=0.050 00 | p.05=0.052 0 | p.05=0.050 00 |

| | p.01=0.010 | p.01=0.009 | p.01=0.008 (*) | p.01=0.009 | p.01=0.009(*),0 |

+-----------+-----------------+-----------------+-----------------+-----------------+-----------------+

|[0.19,0.33)| ks.D=0.012 | ks.D=0.013 | ks.D=0.009 | ks.D=0.012 | ks.D=0.005 |

| | p.05=0.048 0 | p.05=0.047 0 | p.05=0.048 0 | p.05=0.046 0 | p.05=0.047(*),00|

| | p.01=0.010 | p.01=0.009 | p.01=0.009 | p.01=0.009 | p.01=0.009 0 |

+-----------+-----------------+-----------------+-----------------+-----------------+-----------------+

|[0.33,0.57)| ks.D=0.016 (*) | ks.D=0.009 | ks.D=0.011 | ks.D=0.020 * | ks.D=0.007 |

| | p.05=0.050 0 | p.05=0.048 0 | p.05=0.050 0 | p.05=0.052 0 | p.05=0.050 00 |

| | p.01=0.010 | p.01=0.010 | p.01=0.010 | p.01=0.010 | p.01=0.010 0 |

+-----------+-----------------+-----------------+-----------------+-----------------+-----------------+

|[0.57,1.00]| ks.D=0.011 | ks.D=0.015 | ks.D=0.009 | ks.D=0.012 | ks.D=0.006 |

| | p.05=0.049 0 | p.05=0.053 0 | p.05=0.056 * | p.05=0.049 0 | p.05=0.052 00 |

| | p.01=0.012 | p.01=0.011 | p.01=0.010 | p.01=0.010 | p.01=0.011 0 |

+-----------+-----------------+-----------------+-----------------+-----------------+-----------------+

|ALL | ks.D=0.004 | ks.D=0.008 | ks.D=0.003 | ks.D=0.006 | ks.D=0.003 |

| | p.05=0.049 00 | p.05=0.049 00 | p.05=0.051 00 | p.05=0.050 00 | p.05=0.050 00 |

| | p.01=0.010 0 | p.01=0.010 0 | p.01=0.009 0 | p.01=0.009 0 | p.01=0.010 00 |

+-----------+-----------------+-----------------+-----------------+-----------------+-----------------+

$p_{sp}$, $d=meanc$

+-----------+-----------------+-----------------+-----------------+-----------------+-----------------+

| | [ 20, 80) | [ 80, 300) | [300, 600) | [600,1000] | ALL |

+-----------+-----------------+-----------------+-----------------+-----------------+-----------------+

|[0.10,0.19)| ks.D=0.015 | ks.D=0.009 | ks.D=0.008 | ks.D=0.012 | ks.D=0.005 |

| | p.05=0.048 0 | p.05=0.050 0 | p.05=0.050 00 | p.05=0.052 0 | p.05=0.050 00 |

| | p.01=0.011 | p.01=0.011 | p.01=0.010 | p.01=0.010 | p.01=0.010 0 |

+-----------+-----------------+-----------------+-----------------+-----------------+-----------------+

|[0.19,0.33)| ks.D=0.013 | ks.D=0.015 | ks.D=0.006 | ks.D=0.009 | ks.D=0.003 |

| | p.05=0.046 0 | p.05=0.046 0 | p.05=0.051 0 | p.05=0.050 0 | p.05=0.048 00 |

| | p.01=0.009 | p.01=0.009 | p.01=0.009 | p.01=0.010 | p.01=0.009 0 |

+-----------+-----------------+-----------------+-----------------+-----------------+-----------------+

|[0.33,0.57)| ks.D=0.013 | ks.D=0.010 | ks.D=0.014 | ks.D=0.023 ** | ks.D=0.007 |

| | p.05=0.050 0 | p.05=0.049 0 | p.05=0.049 0 | p.05=0.052 0 | p.05=0.050 00 |

| | p.01=0.010 | p.01=0.010 | p.01=0.010 | p.01=0.009 | p.01=0.010 0 |

+-----------+-----------------+-----------------+-----------------+-----------------+-----------------+

|[0.57,1.00]| ks.D=0.010 | ks.D=0.007 | ks.D=0.011 | ks.D=0.011 | ks.D=0.004 |

| | p.05=0.051 0 | p.05=0.050 0 | p.05=0.050 0 | p.05=0.047 0 | p.05=0.049 00 |

| | p.01=0.012 | p.01=0.012 | p.01=0.010 | p.01=0.009 | p.01=0.011 0 |

+-----------+-----------------+-----------------+-----------------+-----------------+-----------------+

|ALL | ks.D=0.005 | ks.D=0.007 | ks.D=0.005 | ks.D=0.006 | ks.D=0.002 |

| | p.05=0.049 00 | p.05=0.049 00 | p.05=0.050 00 | p.05=0.051 00 | p.05=0.049 00 |

| | p.01=0.010 0 | p.01=0.010 0 | p.01=0.010 0 | p.01=0.010 0 | p.01=0.010 00 |

+-----------+-----------------+-----------------+-----------------+-----------------+-----------------+

$p_{sp}$, $d=varc$

+-----------+-----------------+-----------------+-----------------+-----------------+-----------------+

| | [ 20, 80) | [ 80, 300) | [300, 600) | [600,1000] | ALL |

+-----------+-----------------+-----------------+-----------------+-----------------+-----------------+

|[0.10,0.19)| ks.D=0.044 *** | ks.D=0.024 ** | ks.D=0.019 ** | ks.D=0.015 | ks.D=0.025 *** |

| | p.05=0.031***,--| p.05=0.041 ** | p.05=0.046 0 | p.05=0.045(*),0 | p.05=0.041***,- |

| | p.01=0.004***,--| p.01=0.007 **,- | p.01=0.010 | p.01=0.008 | p.01=0.007***,- |

+-----------+-----------------+-----------------+-----------------+-----------------+-----------------+

|[0.19,0.33)| ks.D=0.044 *** | ks.D=0.037 *** | ks.D=0.015 (*) | ks.D=0.022 * | ks.D=0.027 *** |

| | p.05=0.032***,--| p.05=0.043 ** | p.05=0.045 *,0 | p.05=0.054 0 | p.05=0.043***,- |

| | p.01=0.005***,--| p.01=0.009 | p.01=0.008 | p.01=0.012 | p.01=0.008 ** |

+-----------+-----------------+-----------------+-----------------+-----------------+-----------------+

|[0.33,0.57)| ks.D=0.042 *** | ks.D=0.025 ** | ks.D=0.018 * | ks.D=0.013 | ks.D=0.022 *** |

| | p.05=0.038***,- | p.05=0.048 0 | p.05=0.048 0 | p.05=0.050 0 | p.05=0.046 **,0 |

| | p.01=0.006**,-- | p.01=0.008 * | p.01=0.010 | p.01=0.010 | p.01=0.008 ** |

+-----------+-----------------+-----------------+-----------------+-----------------+-----------------+

|[0.57,1.00]| ks.D=0.047 *** | ks.D=0.035 *** | ks.D=0.019 * | ks.D=0.015 | ks.D=0.027 *** |

| | p.05=0.043 ** | p.05=0.049 0 | p.05=0.048 0 | p.05=0.051 0 | p.05=0.048(*),00|

| | p.01=0.008 (*) | p.01=0.010 | p.01=0.010 | p.01=0.011 | p.01=0.010 0 |

+-----------+-----------------+-----------------+-----------------+-----------------+-----------------+

|ALL | ks.D=0.041 *** | ks.D=0.028 *** | ks.D=0.016 *** | ks.D=0.013 ** | ks.D=0.024 *** |

| | p.05=0.036***,--| p.05=0.045 **,0 | p.05=0.047 *,0 | p.05=0.050 00 | p.05=0.044***,0 |

| | p.01=0.006***,--| p.01=0.008 ** | p.01=0.010 0 | p.01=0.010 0 | p.01=0.008***,- |

+-----------+-----------------+-----------------+-----------------+-----------------+-----------------+

$p_{sp}$, $d=LL$

+-----------+-----------------+-----------------+-----------------+-----------------+-----------------+

| | [ 20, 80) | [ 80, 300) | [300, 600) | [600,1000] | ALL |

+-----------+-----------------+-----------------+-----------------+-----------------+-----------------+

|[0.10,0.19)| ks.D=0.045 *** | ks.D=0.024 ** | ks.D=0.017 * | ks.D=0.015 | ks.D=0.024 *** |

| | p.05=0.032***,--| p.05=0.039***,- | p.05=0.046 0 | p.05=0.046 0 | p.05=0.041***,- |

| | p.01=0.004***,--| p.01=0.008 * | p.01=0.009 | p.01=0.010 | p.01=0.007***,- |

+-----------+-----------------+-----------------+-----------------+-----------------+-----------------+

|[0.19,0.33)| ks.D=0.040 *** | ks.D=0.036 *** | ks.D=0.016 (*) | ks.D=0.023 ** | ks.D=0.026 *** |

| | p.05=0.033***,--| p.05=0.041 ** | p.05=0.044 *,0 | p.05=0.054 0 | p.05=0.043***,- |

| | p.01=0.004***,--| p.01=0.009 | p.01=0.009 | p.01=0.011 | p.01=0.008 ** |

+-----------+-----------------+-----------------+-----------------+-----------------+-----------------+

|[0.33,0.57)| ks.D=0.040 *** | ks.D=0.025 ** | ks.D=0.019 * | ks.D=0.012 | ks.D=0.022 *** |

| | p.05=0.038***,- | p.05=0.048 0 | p.05=0.049 0 | p.05=0.050 0 | p.05=0.046 **,0 |

| | p.01=0.007 * | p.01=0.007 * | p.01=0.009 | p.01=0.010 | p.01=0.008 ** |

+-----------+-----------------+-----------------+-----------------+-----------------+-----------------+

|[0.57,1.00]| ks.D=0.046 *** | ks.D=0.035 *** | ks.D=0.019 * | ks.D=0.015 | ks.D=0.026 *** |

| | p.05=0.043 ** | p.05=0.050 0 | p.05=0.047 0 | p.05=0.050 0 | p.05=0.047(*),00|

| | p.01=0.008 | p.01=0.010 | p.01=0.010 | p.01=0.012 | p.01=0.010 0 |

+-----------+-----------------+-----------------+-----------------+-----------------+-----------------+

|ALL | ks.D=0.041 *** | ks.D=0.028 *** | ks.D=0.016 *** | ks.D=0.013 ** | ks.D=0.024 *** |

| | p.05=0.037***,--| p.05=0.044***,0 | p.05=0.047 **,0 | p.05=0.050 00 | p.05=0.044***,0 |

| | p.01=0.006***,--| p.01=0.009 * | p.01=0.009 0 | p.01=0.011 0 | p.01=0.009***,0 |

+-----------+-----------------+-----------------+-----------------+-----------------+-----------------+

## Bernoulli models

$p_{nsp}$, $t=mean$

+-----------+-----------------+-----------------+-----------------+-----------------+-----------------+

| | [ 20, 80) | [ 80, 300) | [300, 600) | [600,1000] | ALL |

+-----------+-----------------+-----------------+-----------------+-----------------+-----------------+

|[0.10,0.19)| ks.D=0.018 * | ks.D=0.024 ** | ks.D=0.009 | ks.D=0.008 | ks.D=0.010 ** |

| | p.05=0.037***,- | p.05=0.042 ** | p.05=0.049 0 | p.05=0.053 0 | p.05=0.045 **,0 |

| | p.01=0.006**,-- | p.01=0.008 | p.01=0.009 | p.01=0.010 | p.01=0.008 ** |

+-----------+-----------------+-----------------+-----------------+-----------------+-----------------+

|[0.19,0.33)| ks.D=0.016 (*) | ks.D=0.010 | ks.D=0.008 | ks.D=0.012 | ks.D=0.008 (*) |

| | p.05=0.044 * | p.05=0.051 0 | p.05=0.050 0 | p.05=0.052 0 | p.05=0.049 00 |

| | p.01=0.008 (*) | p.01=0.009 | p.01=0.011 | p.01=0.010 | p.01=0.010 0 |

+-----------+-----------------+-----------------+-----------------+-----------------+-----------------+

|[0.33,0.57)| ks.D=0.022 ** | ks.D=0.010 | ks.D=0.007 | ks.D=0.020 * | ks.D=0.009 * |

| | p.05=0.041 ** | p.05=0.049 0 | p.05=0.047 0 | p.05=0.047 0 | p.05=0.046 **,0 |

| | p.01=0.007 **,- | p.01=0.010 | p.01=0.010 | p.01=0.010 | p.01=0.009 0 |

+-----------+-----------------+-----------------+-----------------+-----------------+-----------------+

|[0.57,1.00]| ks.D=0.008 | ks.D=0.009 | ks.D=0.013 | ks.D=0.015 | ks.D=0.008 |

| | p.05=0.044 *,0 | p.05=0.048 0 | p.05=0.046 0 | p.05=0.048 0 | p.05=0.046 *,0 |

| | p.01=0.008 | p.01=0.012 (*) | p.01=0.009 | p.01=0.008 | p.01=0.009 0 |

+-----------+-----------------+-----------------+-----------------+-----------------+-----------------+

|ALL | ks.D=0.011 ** | ks.D=0.010 * | ks.D=0.006 | ks.D=0.005 | ks.D=0.006 ** |

| | p.05=0.041***,- | p.05=0.047(*),00| p.05=0.048 00 | p.05=0.050 00 | p.05=0.047***,00|

| | p.01=0.007***,- | p.01=0.010 0 | p.01=0.010 0 | p.01=0.009 0 | p.01=0.009 **,0 |

+-----------+-----------------+-----------------+-----------------+-----------------+-----------------+

$p_{nsp}$, $t=var$

+-----------+-----------------+-----------------+-----------------+-----------------+-----------------+

| | [ 20, 80) | [ 80, 300) | [300, 600) | [600,1000] | ALL |

+-----------+-----------------+-----------------+-----------------+-----------------+-----------------+

|[0.10,0.19)| ks.D=0.032 *** | ks.D=0.030 *** | ks.D=0.019 * | ks.D=0.018 (*) | ks.D=0.022 *** |

| | p.05=0.049 0 | p.05=0.051 0 | p.05=0.053 0 | p.05=0.049 0 | p.05=0.051 00 |

| | p.01=0.009 | p.01=0.009 | p.01=0.011 | p.01=0.008 (*) | p.01=0.009 0 |

+-----------+-----------------+-----------------+-----------------+-----------------+-----------------+

|[0.19,0.33)| ks.D=0.030 *** | ks.D=0.032 *** | ks.D=0.020 ** | ks.D=0.020 * | ks.D=0.023 *** |

| | p.05=0.048 0 | p.05=0.052 0 | p.05=0.051 0 | p.05=0.045 0 | p.05=0.049 00 |

| | p.01=0.011 | p.01=0.011 | p.01=0.010 | p.01=0.009 | p.01=0.010 0 |

+-----------+-----------------+-----------------+-----------------+-----------------+-----------------+

|[0.33,0.57)| ks.D=0.050 *** | ks.D=0.028 ** | ks.D=0.034 *** | ks.D=0.010 | ks.D=0.029 *** |

| | p.05=0.045(*),0 | p.05=0.050 0 | p.05=0.050 0 | p.05=0.050 0 | p.05=0.049 00 |

| | p.01=0.009 | p.01=0.010 | p.01=0.011 | p.01=0.011 | p.01=0.010 0 |

+-----------+-----------------+-----------------+-----------------+-----------------+-----------------+

|[0.57,1.00]| ks.D=0.040 *** | ks.D=0.026 ** | ks.D=0.024 ** | ks.D=0.017 | ks.D=0.024 *** |

| | p.05=0.052 0 | p.05=0.048 0 | p.05=0.050 0 | p.05=0.050 0 | p.05=0.050 00 |

| | p.01=0.011 | p.01=0.010 | p.01=0.009 | p.01=0.008 (*) | p.01=0.010 0 |

+-----------+-----------------+-----------------+-----------------+-----------------+-----------------+

|ALL | ks.D=0.035 *** | ks.D=0.028 *** | ks.D=0.022 *** | ks.D=0.012 ** | ks.D=0.023 *** |

| | p.05=0.049 00 | p.05=0.050 00 | p.05=0.051 00 | p.05=0.049 00 | p.05=0.050 00 |

| | p.01=0.010 0 | p.01=0.010 0 | p.01=0.010 0 | p.01=0.009 (*) | p.01=0.010 00 |

+-----------+-----------------+-----------------+-----------------+-----------------+-----------------+

$p_{nsp}$, $t=skew$

+-----------+-----------------+-----------------+-----------------+-----------------+-----------------+

| | [ 20, 80) | [ 80, 300) | [300, 600) | [600,1000] | ALL |

+-----------+-----------------+-----------------+-----------------+-----------------+-----------------+

|[0.10,0.19)| ks.D=0.017 * | ks.D=0.007 | ks.D=0.014 | ks.D=0.007 | ks.D=0.008 (*) |

| | p.05=0.038***,- | p.05=0.048 0 | p.05=0.050 00 | p.05=0.056 * | p.05=0.047(*),00|

| | p.01=0.007 **,- | p.01=0.011 | p.01=0.011 | p.01=0.009 | p.01=0.009 0 |

+-----------+-----------------+-----------------+-----------------+-----------------+-----------------+

|[0.19,0.33)| ks.D=0.019 * | ks.D=0.016 (*) | ks.D=0.009 | ks.D=0.015 | ks.D=0.010 * |

| | p.05=0.042 ** | p.05=0.051 0 | p.05=0.051 0 | p.05=0.054 0 | p.05=0.049 00 |

| | p.01=0.010 | p.01=0.011 | p.01=0.011 | p.01=0.010 | p.01=0.010 0 |

+-----------+-----------------+-----------------+-----------------+-----------------+-----------------+

|[0.33,0.57)| ks.D=0.011 | ks.D=0.011 | ks.D=0.010 | ks.D=0.023 ** | ks.D=0.007 |

| | p.05=0.043 ** | p.05=0.051 0 | p.05=0.048 0 | p.05=0.050 0 | p.05=0.048(*),00|

| | p.01=0.008 * | p.01=0.010 | p.01=0.011 | p.01=0.014 *,+ | p.01=0.010 0 |

+-----------+-----------------+-----------------+-----------------+-----------------+-----------------+

|[0.57,1.00]| ks.D=0.018 * | ks.D=0.013 | ks.D=0.009 | ks.D=0.012 | ks.D=0.007 |

| | p.05=0.045 *,0 | p.05=0.047 0 | p.05=0.046 0 | p.05=0.048 0 | p.05=0.046 **,0 |

| | p.01=0.008 | p.01=0.010 | p.01=0.010 | p.01=0.009 | p.01=0.009 0 |

+-----------+-----------------+-----------------+-----------------+-----------------+-----------------+

|ALL | ks.D=0.013 ** | ks.D=0.007 | ks.D=0.009 * | ks.D=0.008 | ks.D=0.007 ** |

| | p.05=0.042***,- | p.05=0.049 00 | p.05=0.049 00 | p.05=0.052 00 | p.05=0.048**,00 |

| | p.01=0.008 **,- | p.01=0.010 0 | p.01=0.011 0 | p.01=0.010 0 | p.01=0.010 00 |

+-----------+-----------------+-----------------+-----------------+-----------------+-----------------+

$p_{nsp}$, $t=kurt$

+-----------+-----------------+-----------------+-----------------+-----------------+-----------------+

| | [ 20, 80) | [ 80, 300) | [300, 600) | [600,1000] | ALL |

+-----------+-----------------+-----------------+-----------------+-----------------+-----------------+

|[0.10,0.19)| ks.D=0.036 *** | ks.D=0.022 ** | ks.D=0.015 (*) | ks.D=0.017 (*) | ks.D=0.020 *** |

| | p.05=0.055(*),0 | p.05=0.051 0 | p.05=0.050 00 | p.05=0.058 ** | p.05=0.053 *,0 |

| | p.01=0.010 | p.01=0.010 | p.01=0.007 * | p.01=0.012 (*) | p.01=0.010 0 |

+-----------+-----------------+-----------------+-----------------+-----------------+-----------------+

|[0.19,0.33)| ks.D=0.048 *** | ks.D=0.019 * | ks.D=0.012 | ks.D=0.021 * | ks.D=0.023 *** |

| | p.05=0.052 0 | p.05=0.049 0 | p.05=0.050 0 | p.05=0.052 0 | p.05=0.051 00 |

| | p.01=0.011 | p.01=0.010 | p.01=0.012 (*) | p.01=0.011 | p.01=0.011 |

+-----------+-----------------+-----------------+-----------------+-----------------+-----------------+

|[0.33,0.57)| ks.D=0.037 *** | ks.D=0.020 * | ks.D=0.016 (*) | ks.D=0.020 * | ks.D=0.020 *** |

| | p.05=0.055(*),0 | p.05=0.054 0 | p.05=0.047 0 | p.05=0.052 0 | p.05=0.052 00 |

| | p.01=0.011 | p.01=0.012 | p.01=0.010 | p.01=0.010 | p.01=0.011 0 |

+-----------+-----------------+-----------------+-----------------+-----------------+-----------------+

|[0.57,1.00]| ks.D=0.050 *** | ks.D=0.031 *** | ks.D=0.019 * | ks.D=0.019 (*) | ks.D=0.027 *** |

| | p.05=0.054 0 | p.05=0.056 * | p.05=0.053 0 | p.05=0.046 0 | p.05=0.053(*),00|

| | p.01=0.009 | p.01=0.011 | p.01=0.011 | p.01=0.012 | p.01=0.011 0 |

+-----------+-----------------+-----------------+-----------------+-----------------+-----------------+

|ALL | ks.D=0.040 *** | ks.D=0.022 *** | ks.D=0.013 ** | ks.D=0.016 *** | ks.D=0.021 *** |

| | p.05=0.054 **,0 | p.05=0.052(*),00| p.05=0.050 00 | p.05=0.052 00 | p.05=0.052**,00 |

| | p.01=0.010 0 | p.01=0.010 0 | p.01=0.010 0 | p.01=0.012 * | p.01=0.011 0 |

+-----------+-----------------+-----------------+-----------------+-----------------+-----------------+

$p_{nsp}$, $t=Z_{a}$

+-----------+-----------------+-----------------+-----------------+-----------------+-----------------+

| | [ 20, 80) | [ 80, 300) | [300, 600) | [600,1000] | ALL |

+-----------+-----------------+-----------------+-----------------+-----------------+-----------------+

|[0.10,0.19)| ks.D=0.031 *** | ks.D=0.014 | ks.D=0.009 | ks.D=0.008 | ks.D=0.010 ** |

| | p.05=0.049 0 | p.05=0.047 0 | p.05=0.050 0 | p.05=0.045 0 | p.05=0.048 00 |

| | p.01=0.008 | p.01=0.010 | p.01=0.011 | p.01=0.011 | p.01=0.010 0 |

+-----------+-----------------+-----------------+-----------------+-----------------+-----------------+

|[0.19,0.33)| ks.D=0.024 ** | ks.D=0.013 | ks.D=0.008 | ks.D=0.011 | ks.D=0.010 * |

| | p.05=0.046 0 | p.05=0.056 * | p.05=0.047 0 | p.05=0.051 0 | p.05=0.050 00 |

| | p.01=0.007 **,- | p.01=0.012 | p.01=0.008 (*) | p.01=0.011 | p.01=0.009 0 |

+-----------+-----------------+-----------------+-----------------+-----------------+-----------------+

|[0.33,0.57)| ks.D=0.034 *** | ks.D=0.013 | ks.D=0.011 | ks.D=0.008 | ks.D=0.012 ** |

| | p.05=0.043 * | p.05=0.048 0 | p.05=0.046(*),0 | p.05=0.052 0 | p.05=0.047 *,0 |

| | p.01=0.007 **,- | p.01=0.010 | p.01=0.009 | p.01=0.008 | p.01=0.009 * |

+-----------+-----------------+-----------------+-----------------+-----------------+-----------------+

|[0.57,1.00]| ks.D=0.018 * | ks.D=0.011 | ks.D=0.017 * | ks.D=0.020 * | ks.D=0.012 ** |

| | p.05=0.052 0 | p.05=0.053 0 | p.05=0.051 0 | p.05=0.050 0 | p.05=0.051 00 |

| | p.01=0.012 (*) | p.01=0.010 | p.01=0.008 | p.01=0.009 | p.01=0.010 0 |

+-----------+-----------------+-----------------+-----------------+-----------------+-----------------+

|ALL | ks.D=0.023 *** | ks.D=0.009 (*) | ks.D=0.006 | ks.D=0.007 | ks.D=0.010 *** |

| | p.05=0.047(*),00| p.05=0.051 00 | p.05=0.049 00 | p.05=0.049 00 | p.05=0.049 00 |

| | p.01=0.009 * | p.01=0.011 0 | p.01=0.009 0 | p.01=0.010 0 | p.01=0.009(*),0 |

+-----------+-----------------+-----------------+-----------------+-----------------+-----------------+

$p_{sp}$, $t=mean$

+-----------+-----------------+-----------------+-----------------+-----------------+-----------------+

| | [ 20, 80) | [ 80, 300) | [300, 600) | [600,1000] | ALL |

+-----------+-----------------+-----------------+-----------------+-----------------+-----------------+

|[0.10,0.19)| ks.D=0.036 *** | ks.D=0.018 * | ks.D=0.008 | ks.D=0.010 | ks.D=0.015 *** |

| | p.05=0.030***,--| p.05=0.043 ** | p.05=0.045 *,0 | p.05=0.047 0 | p.05=0.041***,- |

| | p.01=0.005***,--| p.01=0.007 * | p.01=0.009 | p.01=0.010 | p.01=0.008***,- |

+-----------+-----------------+-----------------+-----------------+-----------------+-----------------+

|[0.19,0.33)| ks.D=0.019 * | ks.D=0.024 ** | ks.D=0.008 | ks.D=0.011 | ks.D=0.011 ** |

| | p.05=0.037***,- | p.05=0.042 ** | p.05=0.046 0 | p.05=0.047 0 | p.05=0.043***,0 |

| | p.01=0.006***,--| p.01=0.008 | p.01=0.011 | p.01=0.011 | p.01=0.009 (*) |

+-----------+-----------------+-----------------+-----------------+-----------------+-----------------+

|[0.33,0.57)| ks.D=0.018 * | ks.D=0.012 | ks.D=0.008 | ks.D=0.025 ** | ks.D=0.012 ** |

| | p.05=0.041 ** | p.05=0.046 0 | p.05=0.045(*),0 | p.05=0.049 0 | p.05=0.045 **,0 |

| | p.01=0.007 **,- | p.01=0.009 | p.01=0.008 | p.01=0.009 | p.01=0.008 ** |

+-----------+-----------------+-----------------+-----------------+-----------------+-----------------+

|[0.57,1.00]| ks.D=0.014 | ks.D=0.009 | ks.D=0.014 | ks.D=0.011 | ks.D=0.007 |

| | p.05=0.042 ** | p.05=0.049 0 | p.05=0.047 0 | p.05=0.050 0 | p.05=0.047 *,0 |

| | p.01=0.007 * | p.01=0.008 (*) | p.01=0.008 | p.01=0.009 | p.01=0.008 **,- |

+-----------+-----------------+-----------------+-----------------+-----------------+-----------------+

|ALL | ks.D=0.018 *** | ks.D=0.010 * | ks.D=0.005 | ks.D=0.008 | ks.D=0.009 *** |

| | p.05=0.037***,--| p.05=0.045 **,0 | p.05=0.046 **,0 | p.05=0.048 00 | p.05=0.044***,- |

| | p.01=0.006***,--| p.01=0.008 ** | p.01=0.009 0 | p.01=0.010 0 | p.01=0.008***,- |

+-----------+-----------------+-----------------+-----------------+-----------------+-----------------+

$p_{sp}$, $t=var$

+-----------+-----------------+-----------------+-----------------+-----------------+-----------------+

| | [ 20, 80) | [ 80, 300) | [300, 600) | [600,1000] | ALL |

+-----------+-----------------+-----------------+-----------------+-----------------+-----------------+

|[0.10,0.19)| ks.D=0.212 *** | ks.D=0.261 *** | ks.D=0.287 *** | ks.D=0.282 *** | ks.D=0.257 *** |

| | p.05=0.049 0 | p.05=0.062***,+ | p.05=0.066***,++| p.05=0.072***,++| p.05=0.062***,+ |

| | p.01=0.009 | p.01=0.015***,++| p.01=0.016***,++| p.01=0.017***,++| p.01=0.014***,++|

+-----------+-----------------+-----------------+-----------------+-----------------+-----------------+

|[0.19,0.33)| ks.D=0.246 *** | ks.D=0.271 *** | ks.D=0.286 *** | ks.D=0.292 *** | ks.D=0.271 *** |

| | p.05=0.054 0 | p.05=0.063***,+ | p.05=0.066***,++| p.05=0.071***,++| p.05=0.063***,++|

| | p.01=0.010 | p.01=0.013 * | p.01=0.015***,++| p.01=0.016**,++ | p.01=0.013***,++|

+-----------+-----------------+-----------------+-----------------+-----------------+-----------------+

|[0.33,0.57)| ks.D=0.265 *** | ks.D=0.269 *** | ks.D=0.284 *** | ks.D=0.280 *** | ks.D=0.273 *** |

| | p.05=0.065***,++| p.05=0.060 **,+ | p.05=0.064***,+ | p.05=0.071***,++| p.05=0.065***,++|

| | p.01=0.015**,++ | p.01=0.013 * | p.01=0.013 **,+ | p.01=0.015**,++ | p.01=0.014***,++|

+-----------+-----------------+-----------------+-----------------+-----------------+-----------------+

|[0.57,1.00]| ks.D=0.270 *** | ks.D=0.281 *** | ks.D=0.286 *** | ks.D=0.291 *** | ks.D=0.280 *** |

| | p.05=0.060 ** | p.05=0.072***,++| p.05=0.066***,++| p.05=0.076***,++| p.05=0.068***,++|

| | p.01=0.013 * | p.01=0.016***,++| p.01=0.014**,++ | p.01=0.016***,++| p.01=0.015***,++|

+-----------+-----------------+-----------------+-----------------+-----------------+-----------------+

|ALL | ks.D=0.245 *** | ks.D=0.270 *** | ks.D=0.285 *** | ks.D=0.285 *** | ks.D=0.270 *** |

| | p.05=0.057***,0 | p.05=0.065***,++| p.05=0.066***,++| p.05=0.072***,++| p.05=0.064***,++|

| | p.01=0.012 ** | p.01=0.014***,++| p.01=0.015***,++| p.01=0.016***,++| p.01=0.014***,++|

+-----------+-----------------+-----------------+-----------------+-----------------+-----------------+

$p_{sp}$, $t=p_{0}$

+-----------+-----------------+-----------------+-----------------+-----------------+-----------------+

| | [ 20, 80) | [ 80, 300) | [300, 600) | [600,1000] | ALL |

+-----------+-----------------+-----------------+-----------------+-----------------+-----------------+

|[0.10,0.19)| ks.D=0.034 *** | ks.D=0.019 * | ks.D=0.008 | ks.D=0.011 | ks.D=0.015 *** |

| | p.05=0.033***,--| p.05=0.044 * | p.05=0.044 *,0 | p.05=0.046 0 | p.05=0.042***,- |

| | p.01=0.005***,--| p.01=0.007 **,- | p.01=0.010 | p.01=0.009 | p.01=0.008***,- |

+-----------+-----------------+-----------------+-----------------+-----------------+-----------------+

|[0.19,0.33)| ks.D=0.019 * | ks.D=0.025 ** | ks.D=0.009 | ks.D=0.012 | ks.D=0.011 ** |

| | p.05=0.038***,- | p.05=0.042 ** | p.05=0.046 0 | p.05=0.047 0 | p.05=0.043***,0 |

| | p.01=0.006**,-- | p.01=0.008 | p.01=0.010 | p.01=0.010 | p.01=0.009 * |

+-----------+-----------------+-----------------+-----------------+-----------------+-----------------+

|[0.33,0.57)| ks.D=0.018 * | ks.D=0.013 | ks.D=0.007 | ks.D=0.025 ** | ks.D=0.012 ** |

| | p.05=0.042 ** | p.05=0.048 0 | p.05=0.045(*),0 | p.05=0.048 0 | p.05=0.046 **,0 |

| | p.01=0.007 * | p.01=0.009 | p.01=0.009 | p.01=0.010 | p.01=0.008 * |

+-----------+-----------------+-----------------+-----------------+-----------------+-----------------+

|[0.57,1.00]| ks.D=0.012 | ks.D=0.008 | ks.D=0.013 | ks.D=0.011 | ks.D=0.007 |

| | p.05=0.043 ** | p.05=0.049 0 | p.05=0.049 0 | p.05=0.051 0 | p.05=0.048(*),00|

| | p.01=0.008 (*) | p.01=0.008 | p.01=0.008 | p.01=0.009 | p.01=0.008 ** |

+-----------+-----------------+-----------------+-----------------+-----------------+-----------------+

|ALL | ks.D=0.018 *** | ks.D=0.011 ** | ks.D=0.005 | ks.D=0.008 | ks.D=0.009 *** |

| | p.05=0.039***,- | p.05=0.046 **,0 | p.05=0.046 **,0 | p.05=0.048 00 | p.05=0.044***,0 |

| | p.01=0.006***,--| p.01=0.008 ** | p.01=0.009 0 | p.01=0.009 0 | p.01=0.008***,- |

+-----------+-----------------+-----------------+-----------------+-----------------+-----------------+

$p_{sp}$, $d=meanc$

+-----------+-----------------+-----------------+-----------------+-----------------+-----------------+

| | [ 20, 80) | [ 80, 300) | [300, 600) | [600,1000] | ALL |

+-----------+-----------------+-----------------+-----------------+-----------------+-----------------+

|[0.10,0.19)| ks.D=0.036 *** | ks.D=0.018 * | ks.D=0.008 | ks.D=0.010 | ks.D=0.015 *** |

| | p.05=0.030***,--| p.05=0.043 ** | p.05=0.045 *,0 | p.05=0.047 0 | p.05=0.041***,- |

| | p.01=0.005***,--| p.01=0.007 * | p.01=0.009 | p.01=0.010 | p.01=0.008***,- |

+-----------+-----------------+-----------------+-----------------+-----------------+-----------------+

|[0.19,0.33)| ks.D=0.019 * | ks.D=0.024 ** | ks.D=0.008 | ks.D=0.011 | ks.D=0.011 ** |

| | p.05=0.037***,- | p.05=0.042 ** | p.05=0.046 0 | p.05=0.047 0 | p.05=0.043***,0 |

| | p.01=0.006***,--| p.01=0.008 | p.01=0.011 | p.01=0.011 | p.01=0.009 (*) |

+-----------+-----------------+-----------------+-----------------+-----------------+-----------------+

|[0.33,0.57)| ks.D=0.018 * | ks.D=0.012 | ks.D=0.008 | ks.D=0.025 ** | ks.D=0.012 ** |

| | p.05=0.041 ** | p.05=0.046 0 | p.05=0.045(*),0 | p.05=0.049 0 | p.05=0.045 **,0 |

| | p.01=0.007 **,- | p.01=0.009 | p.01=0.008 | p.01=0.009 | p.01=0.008 ** |

+-----------+-----------------+-----------------+-----------------+-----------------+-----------------+

|[0.57,1.00]| ks.D=0.014 | ks.D=0.009 | ks.D=0.014 | ks.D=0.011 | ks.D=0.007 |

| | p.05=0.042 ** | p.05=0.049 0 | p.05=0.047 0 | p.05=0.050 0 | p.05=0.047 *,0 |

| | p.01=0.007 * | p.01=0.008 (*) | p.01=0.008 | p.01=0.009 | p.01=0.008 **,- |

+-----------+-----------------+-----------------+-----------------+-----------------+-----------------+

|ALL | ks.D=0.018 *** | ks.D=0.010 * | ks.D=0.005 | ks.D=0.008 | ks.D=0.009 *** |

| | p.05=0.037***,--| p.05=0.045 **,0 | p.05=0.046 **,0 | p.05=0.048 00 | p.05=0.044***,- |

| | p.01=0.006***,--| p.01=0.008 ** | p.01=0.009 0 | p.01=0.010 0 | p.01=0.008***,- |

+-----------+-----------------+-----------------+-----------------+-----------------+-----------------+

$p_{sp}$, $d=varc$

+-----------+-----------------+-----------------+-----------------+-----------------+-----------------+

| | [ 20, 80) | [ 80, 300) | [300, 600) | [600,1000] | ALL |

+-----------+-----------------+-----------------+-----------------+-----------------+-----------------+

|[0.10,0.19)| ks.D=0.212 *** | ks.D=0.261 *** | ks.D=0.287 *** | ks.D=0.282 *** | ks.D=0.257 *** |

| | p.05=0.049 0 | p.05=0.062***,+ | p.05=0.066***,++| p.05=0.072***,++| p.05=0.062***,+ |

| | p.01=0.009 | p.01=0.015***,++| p.01=0.016***,++| p.01=0.017***,++| p.01=0.014***,++|

+-----------+-----------------+-----------------+-----------------+-----------------+-----------------+

|[0.19,0.33)| ks.D=0.246 *** | ks.D=0.271 *** | ks.D=0.286 *** | ks.D=0.292 *** | ks.D=0.271 *** |

| | p.05=0.054 0 | p.05=0.063***,+ | p.05=0.066***,++| p.05=0.071***,++| p.05=0.063***,++|

| | p.01=0.010 | p.01=0.013 * | p.01=0.015***,++| p.01=0.016**,++ | p.01=0.013***,++|

+-----------+-----------------+-----------------+-----------------+-----------------+-----------------+

|[0.33,0.57)| ks.D=0.265 *** | ks.D=0.269 *** | ks.D=0.284 *** | ks.D=0.280 *** | ks.D=0.273 *** |

| | p.05=0.065***,++| p.05=0.060 **,+ | p.05=0.064***,+ | p.05=0.071***,++| p.05=0.065***,++|

| | p.01=0.015**,++ | p.01=0.013 * | p.01=0.013 **,+ | p.01=0.015**,++ | p.01=0.014***,++|

+-----------+-----------------+-----------------+-----------------+-----------------+-----------------+

|[0.57,1.00]| ks.D=0.270 *** | ks.D=0.281 *** | ks.D=0.286 *** | ks.D=0.291 *** | ks.D=0.280 *** |

| | p.05=0.060 **,+ | p.05=0.072***,++| p.05=0.066***,++| p.05=0.076***,++| p.05=0.068***,++|

| | p.01=0.013 * | p.01=0.016***,++| p.01=0.014**,++ | p.01=0.016***,++| p.01=0.015***,++|

+-----------+-----------------+-----------------+-----------------+-----------------+-----------------+

|ALL | ks.D=0.245 *** | ks.D=0.270 *** | ks.D=0.285 *** | ks.D=0.285 *** | ks.D=0.270 *** |

| | p.05=0.057***,0 | p.05=0.065***,++| p.05=0.066***,++| p.05=0.072***,++| p.05=0.064***,++|

| | p.01=0.012 ** | p.01=0.014***,++| p.01=0.015***,++| p.01=0.016***,++| p.01=0.014***,++|

+-----------+-----------------+-----------------+-----------------+-----------------+-----------------+

$p_{sp}$, $d=LL$

+-----------+-----------------+-----------------+-----------------+-----------------+-----------------+

| | [ 20, 80) | [ 80, 300) | [300, 600) | [600,1000] | ALL |

+-----------+-----------------+-----------------+-----------------+-----------------+-----------------+

|[0.10,0.19)| ks.D=0.178 *** | ks.D=0.228 *** | ks.D=0.253 *** | ks.D=0.256 *** | ks.D=0.226 *** |

| | p.05=0.031***,--| p.05=0.043 ** | p.05=0.044 *,0 | p.05=0.046 0 | p.05=0.041***,- |

| | p.01=0.005***,--| p.01=0.007 * | p.01=0.009 | p.01=0.009 | p.01=0.007***,- |

+-----------+-----------------+-----------------+-----------------+-----------------+-----------------+

|[0.19,0.33)| ks.D=0.213 *** | ks.D=0.237 *** | ks.D=0.248 *** | ks.D=0.259 *** | ks.D=0.237 *** |

| | p.05=0.037***,- | p.05=0.045(*),0 | p.05=0.047 0 | p.05=0.046 0 | p.05=0.044***,0 |

| | p.01=0.006**,-- | p.01=0.009 | p.01=0.010 | p.01=0.011 | p.01=0.009 (*) |

+-----------+-----------------+-----------------+-----------------+-----------------+-----------------+

|[0.33,0.57)| ks.D=0.226 *** | ks.D=0.231 *** | ks.D=0.250 *** | ks.D=0.242 *** | ks.D=0.236 *** |

| | p.05=0.041 **,- | p.05=0.046 0 | p.05=0.045(*),0 | p.05=0.048 0 | p.05=0.045 **,0 |

| | p.01=0.007 * | p.01=0.010 | p.01=0.009 | p.01=0.009 | p.01=0.009 * |

+-----------+-----------------+-----------------+-----------------+-----------------+-----------------+

|[0.57,1.00]| ks.D=0.237 *** | ks.D=0.247 *** | ks.D=0.241 *** | ks.D=0.254 *** | ks.D=0.243 *** |

| | p.05=0.043 ** | p.05=0.050 0 | p.05=0.048 0 | p.05=0.050 0 | p.05=0.048(*),00|

| | p.01=0.007 * | p.01=0.008 (*) | p.01=0.008 (*) | p.01=0.009 | p.01=0.008 ** |

+-----------+-----------------+-----------------+-----------------+-----------------+-----------------+

|ALL | ks.D=0.211 *** | ks.D=0.235 *** | ks.D=0.247 *** | ks.D=0.252 *** | ks.D=0.235 *** |

| | p.05=0.038***,--| p.05=0.046 **,0 | p.05=0.046 **,0 | p.05=0.048 00 | p.05=0.044***,0 |

| | p.01=0.006***,--| p.01=0.008 ** | p.01=0.009 (*) | p.01=0.009 0 | p.01=0.008***,- |

+-----------+-----------------+-----------------+-----------------+-----------------+-----------------+

## Bernoulli models with $\theta=0.7$

$p_{sp}$, $t=var$

+-----------+-----------------+-----------------+-----------------+-----------------+-----------------+

| | [ 20, 80) | [ 80, 300) | [300, 600) | [600,1000] | ALL |

+-----------+-----------------+-----------------+-----------------+-----------------+-----------------+

|[0.10,0.19)| ks.D=0.042 *** | ks.D=0.021 ** | ks.D=0.018 * | ks.D=0.015 | ks.D=0.022 *** |

| | p.05=0.027***,--| p.05=0.036***,- | p.05=0.044 *,0 | p.05=0.045(*),0 | p.05=0.038***,--|

| | p.01=0.005***,--| p.01=0.005***,--| p.01=0.009 | p.01=0.009 | p.01=0.007***,--|

+-----------+-----------------+-----------------+-----------------+-----------------+-----------------+

|[0.19,0.33)| ks.D=0.048 *** | ks.D=0.020 * | ks.D=0.018 * | ks.D=0.015 | ks.D=0.022 *** |

| | p.05=0.031***,--| p.05=0.040 **,- | p.05=0.043 * | p.05=0.047 0 | p.05=0.040***,- |

| | p.01=0.006**,-- | p.01=0.008 (*) | p.01=0.009 | p.01=0.010 | p.01=0.008 ** |

+-----------+-----------------+-----------------+-----------------+-----------------+-----------------+

|[0.33,0.57)| ks.D=0.051 *** | ks.D=0.021 * | ks.D=0.012 | ks.D=0.015 | ks.D=0.019 *** |

| | p.05=0.035***,--| p.05=0.043 * | p.05=0.044 *,0 | p.05=0.048 0 | p.05=0.042***,- |

| | p.01=0.005***,--| p.01=0.007 *,- | p.01=0.008 | p.01=0.008 | p.01=0.007***,- |

+-----------+-----------------+-----------------+-----------------+-----------------+-----------------+

|[0.57,1.00]| ks.D=0.054 *** | ks.D=0.013 | ks.D=0.017 * | ks.D=0.017 | ks.D=0.022 *** |

| | p.05=0.034***,--| p.05=0.050 0 | p.05=0.047 0 | p.05=0.049 0 | p.05=0.045 **,0 |

| | p.01=0.006**,-- | p.01=0.008 | p.01=0.008 (*) | p.01=0.008 (*) | p.01=0.007***,- |

+-----------+-----------------+-----------------+-----------------+-----------------+-----------------+

|ALL | ks.D=0.046 *** | ks.D=0.016 *** | ks.D=0.014 *** | ks.D=0.010 * | ks.D=0.020 *** |

| | p.05=0.032***,--| p.05=0.042***,- | p.05=0.045***,0 | p.05=0.047(*),0 | p.05=0.041***,- |

| | p.01=0.005***,--| p.01=0.007***,--| p.01=0.008 ** | p.01=0.009 (*) | p.01=0.007***,--|

+-----------+-----------------+-----------------+-----------------+-----------------+-----------------+
