# Supplementary material for: A New Calibrated Bayesian Internal Goodness-of-Fit Method: Sampled Posterior p-Values as Simple and General p-Values That Allow Double Use of the Data
Source: PLoS One. 2011 Mar 18;6(3):e14770. doi: 10.1371/journal.pone.0014770 (PMC3060804; doi:10.1371/journal.pone.0014770)
Supplement: Text S5 — Results of Scenario 4 for the sampled posterior and the posterior predictive p-values. (0.06 MB DOC) [file pone.0014770.s005.doc]

New Calibrated Bayesian Internal Goodness-of-Fit Methods: Sampled Posterior P-values as Simple and General P-values that Allow Double Use of the Data

Frédéric Gosselin

Cemagref, UR EFNO, F-45290 Nogent-sur-Vernisson, France

E-mail: [frederic.gosselin@cemagref.fr](mailto:frederic.gosselin@cemagref.fr)

*Results of Scenario 4 with* $p_{sp}$ and $p_{pop}$

# Text S5. Scenario 4 $p_{sp}$, $p_{pop}$ and $p_{ML_{hs}}$ results for the Poisson model

*Scenario 4*. The setting is the same as in Scenario 1, except that data were generated from fixed parameters, chosen at the mean of their statistical prior in Scenario 1, i.e. $\lambda=\text{exp}\left(1\right)$.

Simulations were performed on $p_{sp}$, $p_{pop}$ and $p_{ML_{hs}}$ with $t=max$. In the following tables, we display the Kolmogorow-Smirnov statistic of the comparison of the p-values with a uniform distribution (ks.D), the proportion of values in the 5% extreme positions on the interval [0;1] (p.05), and the same for 1% (p.01), according to the interval to which $\rho_{0}$ (respectively $1/\rho_{0}$) for Poisson and Normal models (resp. Bernoulli models) (in rows) and the sample size *n* (in columns) belonged. Remember that here $\rho_{0}$ is the random parameter that characterizes the non-informativeness of the statistical prior. 10,000 data set samples were considered. The notation for the significance of the tests is the same as in Supplementary Text S1.

$p_{sp}$, $t=max$

+-----------+-----------------+-----------------+-----------------+

| sigma0 | 20 | 50 | ALL |

+-----------+-----------------+-----------------+-----------------+

|[0.05,0.56)| ks.D=0.027 ** | ks.D=0.021 * | ks.D=0.022 ** |

| | p.05=0.041 ** | p.05=0.045 0 | p.05=0.043 **,0 |

| | p.01=0.008 | p.01=0.011 | p.01=0.010 0 |

+-----------+-----------------+-----------------+-----------------+

|[0.56,1.06)| ks.D=0.011 | ks.D=0.015 | ks.D=0.011 |

| | p.05=0.047 0 | p.05=0.049 0 | p.05=0.048 0 |

| | p.01=0.009 | p.01=0.009 | p.01=0.009 |

+-----------+-----------------+-----------------+-----------------+

|[1.06,1.56)| ks.D=0.027 ** | ks.D=0.011 | ks.D=0.018 ** |

| | p.05=0.051 0 | p.05=0.045 0 | p.05=0.048 0 |

| | p.01=0.011 | p.01=0.008 | p.01=0.010 0 |

+-----------+-----------------+-----------------+-----------------+

|[1.56,2.05]| ks.D=0.022 * | ks.D=0.015 | ks.D=0.018 ** |

| | p.05=0.048 0 | p.05=0.050 0 | p.05=0.049 00 |

| | p.01=0.010 | p.01=0.010 | p.01=0.010 0 |

+-----------+-----------------+-----------------+-----------------+

|ALL | ks.D=0.018 *** | ks.D=0.010 * | ks.D=0.013 *** |

| | p.05=0.047 *,0 | p.05=0.047(*),0 | p.05=0.047**,00 |

| | p.01=0.010 0 | p.01=0.010 0 | p.01=0.010 0 |

+-----------+-----------------+-----------------+-----------------+

$p_{pop}$, $t=max$

+-----------+-----------------+-----------------+-----------------+

| sigma0 | 20 | 50 | ALL |

+-----------+-----------------+-----------------+-----------------+

|[0.05,0.56)| ks.D=0.075 *** | ks.D=0.041 *** | ks.D=0.057 *** |

| | p.05=0.016***,--| p.05=0.028***,--| p.05=0.022***,--|

| | p.01=0.001***,--| p.01=0.005**,-- | p.01=0.003***,--|

+-----------+-----------------+-----------------+-----------------+

|[0.56,1.06)| ks.D=0.067 *** | ks.D=0.042 *** | ks.D=0.053 *** |

| | p.05=0.012***,--| p.05=0.028***,--| p.05=0.020***,--|

| | p.01=0.001***,--| p.01=0.003***,--| p.01=0.002***,--|

+-----------+-----------------+-----------------+-----------------+

|[1.06,1.56)| ks.D=0.074 *** | ks.D=0.043 *** | ks.D=0.057 *** |

| | p.05=0.017***,--| p.05=0.027***,--| p.05=0.022***,--|

| | p.01=0.002***,--| p.01=0.003***,--| p.01=0.003***,--|

+-----------+-----------------+-----------------+-----------------+

|[1.56,2.05]| ks.D=0.077 *** | ks.D=0.036 *** | ks.D=0.052 *** |

| | p.05=0.016***,--| p.05=0.027***,--| p.05=0.022***,--|

| | p.01=0.002***,--| p.01=0.003***,--| p.01=0.002***,--|

+-----------+-----------------+-----------------+-----------------+

|ALL | ks.D=0.072 *** | ks.D=0.036 *** | ks.D=0.054 *** |

| | p.05=0.015***,--| p.05=0.028***,--| p.05=0.021***,--|

| | p.01=0.002***,--| p.01=0.004***,--| p.01=0.003***,--|

+-----------+-----------------+-----------------+-----------------+

$p_{ML_{hs}}$, $t=max$

+-----------+-----------------+-----------------+-----------------+

| sigma0 | 20 | 50 | ALL |

+-----------+-----------------+-----------------+-----------------+

|[0.05,0.56)| ks.D=0.024 ** | ks.D=0.018 (*) | ks.D=0.017 ** |

| | p.05=0.043 * | p.05=0.049 0 | p.05=0.046(*),0 |

| | p.01=0.007 * | p.01=0.011 | p.01=0.009 |

+-----------+-----------------+-----------------+-----------------+

|[0.56,1.06)| ks.D=0.013 | ks.D=0.013 | ks.D=0.006 |

| | p.05=0.050 0 | p.05=0.048 0 | p.05=0.049 00 |

| | p.01=0.010 | p.01=0.008 | p.01=0.009 |

+-----------+-----------------+-----------------+-----------------+

|[1.06,1.56)| ks.D=0.015 | ks.D=0.019 (*) | ks.D=0.016 * |

| | p.05=0.051 0 | p.05=0.048 0 | p.05=0.050 00 |

| | p.01=0.013 * | p.01=0.010 | p.01=0.012 |

+-----------+-----------------+-----------------+-----------------+

|[1.56,2.05]| ks.D=0.014 | ks.D=0.011 | ks.D=0.008 |

| | p.05=0.048 0 | p.05=0.051 0 | p.05=0.050 00 |

| | p.01=0.009 | p.01=0.009 | p.01=0.009 |

+-----------+-----------------+-----------------+-----------------+

|ALL | ks.D=0.014 ** | ks.D=0.009 (*) | ks.D=0.008 * |

| | p.05=0.048 00 | p.05=0.049 00 | p.05=0.048 00 |

| | p.01=0.010 0 | p.01=0.010 0 | p.01=0.010 0 |

+-----------+-----------------+-----------------+-----------------+

# 
