# Supplementary material for: A New Calibrated Bayesian Internal Goodness-of-Fit Method: Sampled Posterior p-Values as Simple and General p-Values That Allow Double Use of the Data
Source: PLoS One. 2011 Mar 18;6(3):e14770. doi: 10.1371/journal.pone.0014770 (PMC3060804; doi:10.1371/journal.pone.0014770)
Supplement: Text S6 — A simple illustration of the strong dependence of prior predictive p-values on the prior distribution. (0.06 MB DOC) [file pone.0014770.s006.doc]

New Calibrated Bayesian Internal Goodness-of-Fit Methods: Sampled Posterior P-values as Simple and General P-values that Allow Double Use of the Data

Frédéric Gosselin

Cemagref, UR EFNO, F-45290 Nogent-sur-Vernisson, France

E-mail: [frederic.gosselin@cemagref.fr](mailto:frederic.gosselin@cemagref.fr)

# Text S6. A simple illustration of the strong dependence of prior predictive p-values ($p_{prp}$) on the prior distribution

We show the results for Scenario 2 for the Poisson model below:

Scenario 2. The setting is the same as in Scenario 1, except that $\rho_{0}$ is replaced in the statistical model by $\mu_{\sigma_{0}}\rho_{0}$, where $\text{log}\left(\mu_{\sigma_{0}}\right)\sim\text{Normal}\left(\text{0, 1.4}^{2}\right)$;

Simulations were performed in R on $p_{prp}$. In the following tables, we display the Kolmogorow-Smirnov statistic of the comparison of the p-values with a uniform distribution (ks.D), the proportion of values in the 5% extreme positions on the interval [0;1] (p.05), and the same for 1% (p.01), according to the interval to which $\mu_{\sigma_{0}}$ (in rows) belonged. Three thousand data set samplings and analyzes were performed. The rest of the notation is the same as in Supplementary Text S1.

$p_{prp}$, $t=mean$

$\mu_{\sigma_{0}}$ ks.D p.05 p.01

1 [0.01, 0.41) 0.236 *** 0.371***,++ 0.246***,++

2 [0.41, 1.03) 0.068 ** 0.105***,++ 0.034***,++

3 [1.03, 2.69) 0.093 *** 0.019***,-- 0.001**,--

4 [2.69,530.25] 0.280 *** 0.000***,-- 0.000**,--

5 ALL 0.050 *** 0.125***,++ 0.071***,++

$p_{prp}$, $t=var$

$\mu_{\sigma_{0}}$ ks.D p.05 p.01

1 [0.01, 0.41) 0.210 *** 0.322***,++ 0.213***,++

2 [0.41, 1.03) 0.061 ** 0.106***,++ 0.034***,++

3 [1.03, 2.69) 0.084 *** 0.022***,-- 0.003 *,-

4 [2.69,530.25] 0.258 *** 0.001***,-- 0.000**,--

5 ALL 0.046 *** 0.114***,++ 0.063***,++

$p_{prp}$, $t=skew$

$\mu_{\sigma_{0}}$ ks.D p.05 p.01

1 [0.01, 0.41) 0.096 *** 0.105***,++ 0.031***,++

2 [0.41, 1.03) 0.033 0.055 0.011

3 [1.03, 2.69) 0.071 ** 0.035 (*) 0.003 *,-

4 [2.69,530.25] 0.221 *** 0.016***,-- 0.000**,--

5 ALL 0.048 *** 0.053 0.011

$p_{prp}$, $t=kurt$

$\mu_{\sigma_{0}}$ ks.D p.05 p.01

1 [0.01, 0.41) 0.063 ** 0.060 0.010

2 [0.41, 1.03) 0.029 0.038 0.004

3 [1.03, 2.69) 0.052 * 0.046 0.011

4 [2.69,530.25] 0.158 *** 0.028 **,- 0.001**,--

5 ALL 0.038 ** 0.043 (*) 0.007 *

# 
