# Supplementary material for: A New Calibrated Bayesian Internal Goodness-of-Fit Method: Sampled Posterior p-Values as Simple and General p-Values That Allow Double Use of the Data
Source: PLoS One. 2011 Mar 18;6(3):e14770. doi: 10.1371/journal.pone.0014770 (PMC3060804; doi:10.1371/journal.pone.0014770)
Supplement: Text S7 — Results of the normalized sampled posterior p-value with frequentist Poisson generalized linear models. (0.07 MB DOC) [file pone.0014770.s007.doc]

New Calibrated Bayesian Internal Goodness-of-Fit Methods: Sampled Posterior P-values as Simple and General P-values that Allow Double Use of the Data

Frédéric Gosselin

Cemagref, UR EFNO, F-45290 Nogent-sur-Vernisson, France

E-mail: [frederic.gosselin@cemagref.fr](mailto:frederic.gosselin@cemagref.fr)

$p_{\text{nsp}}$ results with frequentist Poisson generalized linear models

*Table S1. Kolomogorov-smirnov distance (D) between the simulated $p_{\text{nsp}}$ and the uniform distribution and frequency ($P_{\text{5\%}}$ and $P_{\text{1\%}}$) of $p_{\text{nsp}}$ values that are at the 5% and 1% extremities of the unit interval, for the various t functions, for the Poisson generalized linear model (glm). Based on 3,000 different data values simulated from a Poisson with fixed parameter 1.*

|  | glm |
| --- | --- |
| mean |  |
| variance |  |
| skewness |  |
| kurtosis |  |
|  |  |

NOTE: The notation for the significance of the tests is the same as in Supplementary Text S1.

*Table S2. Kolomogorov-smirnov distance (D) between the simulated $p_{\text{nsp}}$ and the uniform distribution and frequency ($P_{\text{5\%}}$ and $P_{\text{1\%}}$) of $p_{\text{nsp}}$ values that are at the 5% and 1% extremities of the unit interval, for the various t functions, for the Poisson generalized linear model (glm). Based on 3,000 different data values simulated from a Poisson with random parameter , with $\sigma_{0}$ being sampled – for each data set – according to the distribution $U\left( 0;2 \right)+0.05$.*

|  | glm |
| --- | --- |
| mean |  |
| variance |  |
| skewness |  |
| kurtosis |  |
|  |  |

NOTE: The notation for the significance of the tests is the same as in Supplementary Text S1.

# 
