# Supplementary material for: A New Calibrated Bayesian Internal Goodness-of-Fit Method: Sampled Posterior p-Values as Simple and General p-Values That Allow Double Use of the Data
Source: PLoS One. 2011 Mar 18;6(3):e14770. doi: 10.1371/journal.pone.0014770 (PMC3060804; doi:10.1371/journal.pone.0014770)
Supplement: Text S8 — R commands to run and analyze the simulations. (0.93 MB DOC) [file pone.0014770.s008.doc]

New Calibrated Bayesian Internal Goodness-of-Fit Methods: Sampled Posterior P-values as Simple and General P-values that Allow Double Use of the Data

Frédéric Gosselin

Cemagref, UR EFNO, F-45290 Nogent-sur-Vernisson, France

E-mail: [frederic.gosselin@cemagref.fr](mailto:frederic.gosselin@cemagref.fr)

*R commands to run and analyze the simulations*

# R commands to run the simulations

## #shared functions and commands to load before any work under R:

library(Hmisc)

#library Hmisc is necessary for tables

rpolya<-function(N = 1, n = 10, p = 0.5, gamma = 0.1, gen = NULL)

{

#N: number of samples to draw

#n, alpha, beta: parameters of the distroibution (generalized negative binomial)

#gen: random numbers (in case one wants to control it)

n <- floor(n)

valuemax <- 1

res <- rep(0, N)

if(is.null(gen)) {

gen <- runif(N)

}

else {

if(length(gen) == 1) {

gen <- rep(gen, N)

}

}

if(length(n) == 1) {

n <- rep(n, N)

}

if(length(p) == 1) {

p <- rep(p, N)

}

if(length(gamma) == 1) {

gamma <- rep(gamma, N)

}

for(i in 1:N) {

x <- 0

pb <- p[i]

gammab <- gamma[i]

nb <- n[i]

if (gamma[i]==0){x<-rbinom(1,nb,pb)+1} else {

value <- 0

while(gen[i] >= value) {

value = value + exp(lgamma(pb/gammab + x) - lgamma(pb/gammab) + lgamma((1 - pb)/gammab + nb - x) - lgamma((1 - pb)/gammab) - lgamma(1/gammab + nb) + lgamma(1/gammab) - lgamma(x + 1) - lgamma(nb - x + 1) + lgamma(nb + 1))

#value = value + choose(nb,x)*beta(pb/gammab + x, (1 - pb)/gammab + nb - x)/ beta(pb/gammab, (1 - pb)/gammab)

x <- x + 1

#print(c(x,value))

}

}

res[i] <- x - 1

}

res

}

skewness<- function(x){mean((x-mean(x))^3)/mean((x-mean(x))^2)^1.5}

kurtosis<- function(x){mean((x-mean(x))^4)/mean((x-mean(x))^2)^2-3}

zhang<- function(x,y){

#cf. Zhang J., 2002, Powerful goodness-of-fit tests based on the likelihood ratio, Journal of the Royal Statistical Society Series B-statistical Methodology, 64, p. 281-294.

# and: Zhang J. et Wu Y.H., 2005, Likelihood-ratio tests for normality, Computational Statistics & Data Analysis, 49, 3, p. 709-721.

N<-length(x)

#x<-sort(x)

vec.ref.1<-(1:N)-0.5

vec.ref.2<-rev(vec.ref.1)

#old erroneous version: temp<-eval(y)(x)

#old new version: temp<-eval(y)(x-0.00001)+runif(length(x))*( eval(y)(x)- eval(y)(x-0.00001))

temp<- runif(length(x),eval(y)(x-0.00001), eval(y)(x+0.00001))

temp<-sort(temp)

Za<- -sum(log(temp)/vec.ref.2+log(1-temp)/vec.ref.1)

Zc<- sum(log((temp^(-1)-1)/((N-0.5)/(vec.ref.1-0.25)-1))^2)

list(Za=Za,Zc=Zc)}

quantile.for.diags<- function (x, y)

{

prop.equal <- runif(1)

k <- rbeta(1, sum(x < y) + sum(x == y) * prop.equal + 1,

sum(x > y) + sum(x == y) * (1 - prop.equal) + 1)

k

}

symbols.ks<-function(x)

{

zsign<-NULL

znegl<-NULL

if(ks.test(x,"punif")$p<0.1& ks.test(x,"punif")$p>=0.05)

{ zsign<-"(*)"}

if(ks.test(x,"punif")$p<0.05& ks.test(x,"punif")$p>=0.01)

{ zsign<-"*"}

if(ks.test(x,"punif")$p<0.01& ks.test(x,"punif")$p>=0.0001)

{zsign<-"**"}

if(ks.test(x,"punif")$p<0.0001)

{zsign<-"***"}

toto<-paste(zsign,if(!is.null(znegl)&!is.null(zsign)){","},znegl,sep="")

#nchartoto<-if (length(nchar(toto))>0){(3-nchar(toto))/2} else {1.5}

nchartoto<-if (length(nchar(toto))>0){(6-nchar(toto))/2} else {3}

toto<-paste(paste(rep(" ",floor(nchartoto)),collapse=""),toto,paste(rep(" ",ceiling(nchartoto)),collapse=""),sep="")

toto

}

symbols.p05<-function(x)

{

zsign<-NULL

znegl<-NULL

y<-rbeta(10000,sum(x<0.05/2| x>(1-0.05/2))+1,length(x)-sum(x<0.05/2| x>(1-0.05/2))+1);

if({mean({y<0.06&y>0.04})}>=0.95)

{ znegl<-"0"}

if({mean({y<0.055&y>0.045})}>=0.95)

{ znegl<-"00"}

if({mean({y>0.055})}>=0.95)

{ znegl<-"+"}

if({mean({y>0.06})}>=0.95)

{ znegl<-"++"}

if({mean({y<0.045})}>=0.95)

{ znegl<-"-"}

if({mean({y<0.04})}>=0.95)

{ znegl<-"--"}

y<-{y<-pbinom(sum(x<0.05/2| x>(1-0.05/2))-1,length(x),0.05)+runif(1)*dbinom(sum(x<0.05/2| x>(1-0.05/2)),length(x),0.05);pmin(y*2,(1-y)*2)}

if({y}<0.1& {y }>=0.05)

{ zsign<-"(*)"}

if({y}<0.05& {y}>=0.01)

{ zsign<-"*"}

if({y}<0.01& {y}>=0.0001)

{zsign<-"**"}

if({y}<0.0001)

{zsign<-"***"}

toto<-paste(zsign,if(!is.null(znegl)&!is.null(zsign)){","},znegl,sep="")

nchartoto<-if (length(nchar(toto))>0){(6-nchar(toto))/2} else {3}

toto<-paste(paste(rep(" ",floor(nchartoto)),collapse=""),toto,paste(rep(" ",ceiling(nchartoto)),collapse=""),sep="")

toto

}

symbols.p01<-function(x)

{

zsign<-NULL

znegl<-NULL

y<-rbeta(10000,sum(x<0.01/2| x>(1-0.01/2))+1,length(x)-sum(x<0.01/2| x>(1-0.01/2))+1)

if({mean({y<0.012&y>0.008})}>=0.95)

{ znegl<-"0"}

if({mean({y<0.011&y>0.009})}>=0.95)

{ znegl<-"00"}

if({mean({y>0.011})}>=0.95)

{ znegl<-"+"}

if({mean({y>0.012})}>=0.95)

{ znegl<-"++"}

if({mean({y<0.009})}>=0.95)

{ znegl<-"-"}

if({mean({y<0.008})}>=0.95)

{ znegl<-"--"}

y<-{y<-pbinom(sum(x<0.01/2| x>(1-0.01/2))-1,length(x),0.01)+runif(1)*dbinom(sum(x<0.01/2| x>(1-0.01/2)),length(x),0.01);pmin(y*2,(1-y)*2)}

if({y}<0.1& {y }>=0.05)

{ zsign<-"(*)"}

if({y}<0.05& {y}>=0.01)

{ zsign<-"*"}

if({y}<0.01& {y}>=0.0001)

{zsign<-"**"}

if({y}<0.0001)

{zsign<-"***"}

toto<-paste(zsign,if(!is.null(znegl)&!is.null(zsign)){","},znegl,sep="")

nchartoto<-if (length(nchar(toto))>0){(6-nchar(toto))/2} else {3}

toto<-paste(paste(rep(" ",floor(nchartoto)),collapse=""),toto,paste(rep(" ",ceiling(nchartoto)),collapse=""),sep="")

toto

}

## #Poisson-gamma utilisant conjugate, theta1, taurandomV1

### #nspp in Poisson glm

#here C:\\Dossier Frederic\\HDR\\diagnostics\\results_R\\ref_ppos\\ref_ppos.RData contains calculations of the moments of standard normal samples of various sizes (in the object called ref.ppos.rnorm)

#function used to simulate the data and the analyses and p-values

simulate.glm.Poisson.simple<-function(meta.params.expression=expression({toto<- *{temp<- rbinom(1,1,0.7); temp*exp(runif(1)*3.45+3)+(1-temp)* (250+750* runif(1))}; if (toto<200){toto<-round(toto,digits=-1)} else {toto<-round(toto,digits=-2)}; sigma<-(2*runif(1)+0.05);* list(J=toto, N.y=toto,sigma=sigma)}), true.params.expression=expression(list(tau=1)), y.expression=expression({rpois(meta.params$J,true.params$tau)}), rep=1000)

{ res<-(NULL)

attach("C:\\Dossier Frederic\\HDR\\diagnostics\\results_R\\ref_ppos\\ref_ppos.RData", pos = 3, name = "refppos")

print("refppos attached")

for (i in 1:rep)

{ set.seed(i)

meta.params<-eval(meta.params.expression)

true.params<-eval(true.params.expression)

y<-eval(y.expression)

#(moyenne de 1.12: OK)

# (pour ne plus y toucher) y.pois1.forcompareb<-y

glm.refb<-glm(y~1,family="poisson")

theta.ref<- exp(glm.refb$coef+rnorm(1)*sqrt(summary(glm.refb)$cov.unscaled))

res$true.params<-c(res$true.params,true.params)

res$mean<-c(res$mean,sapply(1:1,function(x,y,N,theta){dist.ref<- qnorm(ppois(y-1, theta)+runif(N)* dpois(y, theta));

mean.transf.y.pois1.forcompare <-mean(dist.ref); pmean.transf.y.pois1.forcompare<-0; ref.list<-ref.ppos.rnorm[[as.character(N)]]$mean$original.values; pmean.transf.y.pois1.forcompare <-quantile.for.diags(mean.transf.y.pois1.forcompare, ref.list);pmean.transf.y.pois1.forcompare}, y, meta.params$J, theta.ref))

res$var<-c(res$var,sapply(1:1,function(x,y,N,theta){dist.ref<- qnorm(ppois(y-1, theta)+runif(N)* dpois(y, theta));

var.transf.y.pois1.forcompare <-var(dist.ref); pmean.transf.y.pois1.forcompare<-0; ref.list<-ref.ppos.rnorm[[as.character(N)]]$var$original.values; pmean.transf.y.pois1.forcompare <-quantile.for.diags(var.transf.y.pois1.forcompare, ref.list);pmean.transf.y.pois1.forcompare}, y, meta.params$J, theta.ref))

res$skewness<-c(res$skewness,sapply(1:1,function(x,y,N,theta){dist.ref<- qnorm(ppois(y-1, theta)+runif(N)* dpois(y, theta));

var.transf.y.pois1.forcompare <-skewness(dist.ref); pmean.transf.y.pois1.forcompare<-0; ref.list<-ref.ppos.rnorm[[as.character(N)]]$skewness$original.values; pmean.transf.y.pois1.forcompare <-quantile.for.diags(var.transf.y.pois1.forcompare, ref.list);pmean.transf.y.pois1.forcompare}, y, meta.params$J, theta.ref))

res$kurtosis <-c(res$kurtosis,sapply(1:1,function(x,y,N,theta){dist.ref<- qnorm(ppois(y-1, theta)+runif(N)* dpois(y, theta));

var.transf.y.pois1.forcompare <- kurtosis(dist.ref); pmean.transf.y.pois1.forcompare<-0; ref.list<-ref.ppos.rnorm[[as.character(N)]]$kurt$original.values; pmean.transf.y.pois1.forcompare <-quantile.for.diags(var.transf.y.pois1.forcompare, ref.list);pmean.transf.y.pois1.forcompare}, y, meta.params$J, theta.ref))

res$Za<-c(res$Za,sapply(1:1,function(x,y,N,theta){dist.ref<- qnorm(ppois(y-1, theta)+runif(N)* dpois(y, theta));

var.transf.y.pois1.forcompare <- zhang(dist.ref,expression(pnorm))$Za; pmean.transf.y.pois1.forcompare<-0; ref.list<-ref.ppos.rnorm[[as.character(N)]]$Za$original.values; pmean.transf.y.pois1.forcompare <-quantile.for.diags(var.transf.y.pois1.forcompare, ref.list);pmean.transf.y.pois1.forcompare}, y, meta.params$J, theta.ref))

res$N<-c(res$N,meta.params$J)

}

print("before refppos detached")

detach(3)

res

}

results.Poisson.Nrandom.theta0.fixedtau.glm<- simulate.glm.Poisson.simple(rep=3000)

results.Poisson.Nrandom.taurandom.glm<- simulate.glm.Poisson.simple(true.params.expression=expression(list(tau=exp(rnorm(1,1, meta.params$sigma)))), y.expression=expression({rpois(meta.params$J,true.params$tau)}),rep=3000)

### #nspp, Nrandom, taurandom, Scenario1, complément C, en local

ppp<- NULL

musigma0<-NULL

rep=10000

#rep<-100

rep.inner<-5000

theta0ref<-exp(1)

for (i in 1:rep){

set.seed(i)

meta.params<- ***{toto<-{temp<- rbinom(1,1,0.7); temp*exp(runif(1)*3.45+3)+(1-temp)* (250+750* runif(1))}; if (toto<200){toto<-round(toto,digits=-1)} else {toto<-round(toto,digits=-2)}; sigma<-2*runif(1)+0.05;***

list(J=toto, N.y=toto,sigma=(sigma))}

true.params<-(list(tau=(rgamma(1, theta0ref/meta.params$sigma, 1/meta.params$sigma))))

y<-({rpois(meta.params$J,true.params$tau)})

**#sigma0<-rlnorm(1,0,1.4)*meta.params$sigma**

**sigma0<- meta.params$sigma**

theta0<- theta0ref

**#thetatilde:**

thetatilde<-(rgamma(1, theta0/meta.params$sigma+sum(y), (1/meta.params$sigma+meta.params$J)))

y.norm<-qnorm(ppois(y-0.1,thetatilde)+runif(length(y))*( ppois(y,thetatilde)- ppois(y-0.1,thetatilde)))

#theta<-rnorm(1,0,1)

sample.sim.norm<-sapply(1:rep.inner,function(x){toto<-rnorm(meta.params$J); toto0<-F;if (sum(!is.finite(toto))==0) {toto0=var(toto)==0} else {toto0=T};while (toto0){toto<- rnorm(meta.params$J); toto0<-F;if (sum(!is.finite(toto))==0) {toto0=var(toto)==0} else {toto0=T}}; x<-toto;c(mean(x),var(x),skewness(x),kurtosis(x),zhang(x,pnorm)$Za)})

toto<-c(mean=quantile.for.diags(sample.sim.norm[1,],mean(y.norm)),

var= quantile.for.diags(sample.sim.norm[2,],var(y.norm)),

skew= quantile.for.diags(sample.sim.norm[3,],skewness(y.norm)),

kurt= quantile.for.diags(sample.sim.norm[4,],kurtosis(y.norm)),

Za= quantile.for.diags(sample.sim.norm[5,],zhang(y.norm,pnorm)$Za))

ppp<-rbind(ppp,c(toto,musigma0=sigma0/meta.params$sigma,N=meta.params$J))

}

#1 itération=0.5"

results.nspp.Poisson.Nrandom.theta1.sigma2.taurandomV1.Sc1.conjugate.R<-ppp

***save.image("Diags_Poisson2.RData")***

### #spp, Nrandom, taurandom, Scenario1, complément C, en local

ppp<- NULL

musigma0<-NULL

rep=10000

#rep<-100

rep.inner<-5000

theta0ref<-exp(1)

for (i in 1:rep){

set.seed(i)

meta.params<- ***{toto<-{temp<- rbinom(1,1,0.7); temp*exp(runif(1)*3.45+3)+(1-temp)* (250+750* runif(1))}; if (toto<200){toto<-round(toto,digits=-1)} else {toto<-round(toto,digits=-2)}; sigma<-2*runif(1)+0.05;***

list(J=toto, N.y=toto,sigma=(sigma))}

true.params<-(list(tau=(rgamma(1, theta0ref/meta.params$sigma, 1/meta.params$sigma))))

y<-({rpois(meta.params$J,true.params$tau)})

**#sigma0<-rlnorm(1,0,1.4)*meta.params$sigma**

**sigma0<- meta.params$sigma**

theta0<- theta0ref

**#thetatilde:**

thetatilde<-(rgamma(1, theta0/meta.params$sigma+sum(y), (1/meta.params$sigma+meta.params$J)))

#y.norm<-qnorm(ppois(y-0.1,thetatilde)+runif(length(y))*( ppois(y,thetatilde)- ppois(y-0.1,thetatilde)))

#theta<-rnorm(1,0,1)

sample.sim.norm<-sapply(1:rep.inner,function(x){toto<- rpois(meta.params$J,thetatilde); x<-toto;c(mean(x),var(x),skewness(x),kurtosis(x),zhang(x,pnorm)$Za)})

toto<-c(mean=quantile.for.diags(sample.sim.norm[1,],mean(y)),

var= quantile.for.diags(sample.sim.norm[2,],var(y)))

ppp<-rbind(ppp,c(toto,musigma0=sigma0/meta.params$sigma,N=meta.params$J))

}

#1 itération=0.5"

results.spp.Poisson.Nrandom.theta1.sigma2.taurandomV1.Sc1.conjugate.R<-ppp

***save.image("Diags_Poisson2.RData")***

### #dspp, Nrandom, taurandom, Scenario1, complément C

ppp<- NULL

musigma0<-NULL

rep=10000

#rep<-100

rep.inner<-5000

theta0ref<-exp(1)

for (i in 1:rep){

set.seed(i)

meta.params<- ***{toto<-{temp<- rbinom(1,1,0.7); temp*exp(runif(1)*3.45+3)+(1-temp)* (250+750* runif(1))}; if (toto<200){toto<-round(toto,digits=-1)} else {toto<-round(toto,digits=-2)}; sigma<-2*runif(1)+0.05;***

list(J=toto, N.y=toto,sigma=(sigma))}

true.params<-(list(tau=(rgamma(1, theta0ref/meta.params$sigma, 1/meta.params$sigma))))

y<-({rpois(meta.params$J,true.params$tau)})

**#sigma0<-rlnorm(1,0,1.4)*meta.params$sigma**

**sigma0<- meta.params$sigma**

theta0<- theta0ref

**#thetatilde:**

thetatilde<-(rgamma(1, theta0/meta.params$sigma+sum(y), (1/meta.params$sigma+meta.params$J)))

#y.norm<-qnorm(ppois(y-0.1,thetatilde)+runif(length(y))*( ppois(y,thetatilde)- ppois(y-0.1,thetatilde)))

#theta<-rnorm(1,0,1)

sample.sim.norm<-sapply(1:rep.inner,function(x){toto<- rpois(meta.params$J,thetatilde); x<-toto;c(mean(x)- thetatilde,var(x)- thetatilde,mean(x==0),mean(x[1]==0))})

toto<-c(meanc=quantile.for.diags(sample.sim.norm[1,],mean(y)- thetatilde),

varc= quantile.for.diags(sample.sim.norm[2,],var(y)- thetatilde),p0= quantile.for.diags(sample.sim.norm[3,],mean(y==0)), p01= quantile.for.diags(sample.sim.norm[4,],mean(y[1]==0)))

ppp<-rbind(ppp,c(toto,musigma0=sigma0/meta.params$sigma,N=meta.params$J))

}

#1 itération=0.5"

results.dspp.Poisson.Nrandom.theta1.sigma2.taurandomV1.Sc1.conjugate.R<-ppp

***save.image("Diags_Poisson2.RData")***

### #dspp3, Nrandombig, taurandom, Scenario1, complément C

ppp<- NULL

musigma0<-NULL

rep=20000

#rep<-100

rep.inner<-5000

theta0ref<-exp(1)

for (i in 1:rep){

set.seed(i)

meta.params<- ***{toto<-{temp<- 0; temp*exp(runif(1)*3.45+3)+(1-temp)* (250+750* runif(1))}; if (toto<200){toto<-round(toto,digits=-1)} else {toto<-round(toto,digits=-2)}; sigma<-2*runif(1)+0.05;***

list(J=toto, N.y=toto,sigma=(sigma))}

true.params<-(list(tau=(rgamma(1, theta0ref/meta.params$sigma, 1/meta.params$sigma))))

y<-({rpois(meta.params$J,true.params$tau)})

**#sigma0<-rlnorm(1,0,1.4)*meta.params$sigma**

**sigma0<- meta.params$sigma**

theta0<- theta0ref

**#thetatilde:**

thetatilde<-(rgamma(1, theta0/meta.params$sigma+sum(y), (1/meta.params$sigma+meta.params$J)))

#y.norm<-qnorm(ppois(y-0.1,thetatilde)+runif(length(y))*( ppois(y,thetatilde)- ppois(y-0.1,thetatilde)))

#theta<-rnorm(1,0,1)

sample.sim.norm<-sapply(1:rep.inner,function(x){toto<- rpois(meta.params$J,thetatilde); x<-toto;c(mean(x)- thetatilde, mean(x))})

toto<-c(meanc=quantile.for.diags(sample.sim.norm[1,],mean(y)- thetatilde),

mean= quantile.for.diags(sample.sim.norm[2,],mean(y)))

ppp<-rbind(ppp,c(toto,musigma0=sigma0/meta.params$sigma,N=meta.params$J))

}

#1 itération=0.5"

results.dspp3.Poisson.Nrandombis.theta1.sigma2.taurandomV1.Sc1.conjugate.R<-ppp

***save.image("Diags_Poisson.RData")***

### #dspp2, Nrandom, taurandom, Scenario1, complément C

ppp<- NULL

musigma0<-NULL

rep=10000

#rep<-50

rep.inner<-5000

theta0ref<-exp(1)

for (i in 1:rep){

set.seed(i)

meta.params<- ***{toto<-{temp<- rbinom(1,1,0.7); temp*exp(runif(1)*3.45+3)+(1-temp)* (250+750* runif(1))}; if (toto<200){toto<-round(toto,digits=-1)} else {toto<-round(toto,digits=-2)}; sigma<-2*runif(1)+0.05;***

list(J=toto, N.y=toto,sigma=(sigma))}

true.params<-(list(tau=(rgamma(1, theta0ref/meta.params$sigma, 1/meta.params$sigma))))

y<-({rpois(meta.params$J,true.params$tau)})

**#sigma0<-rlnorm(1,0,1.4)*meta.params$sigma**

**sigma0<- meta.params$sigma**

theta0<- theta0ref

**#thetatilde:**

thetatilde<-(rgamma(1, theta0/meta.params$sigma+sum(y), (1/meta.params$sigma+meta.params$J)))

#y.norm<-qnorm(ppois(y-0.1,thetatilde)+runif(length(y))*( ppois(y,thetatilde)- ppois(y-0.1,thetatilde)))

#theta<-rnorm(1,0,1)

sample.sim.norm<-sapply(1:rep.inner,function(x){toto<- rpois(meta.params$J,thetatilde); x<-toto;c(sum(dpois(x,thetatilde,log=T)))})

toto<-c(LL=quantile.for.diags(sample.sim.norm, sum(dpois(y,thetatilde,log=T))))

ppp<-rbind(ppp,c(toto,musigma0=sigma0/meta.params$sigma,N=meta.params$J))

}

#1 itération=0.8" à 1"

results.dspp2.Poisson.Nrandom.theta1.sigma2.taurandomV1.Sc1.conjugate.R<-ppp

***save.image("Diags_Poisson_CL3.RData")***

### #dsppbis, Nrandom, taurandom, Scenario1, complément C

ppp<-NULL

musigma0<-NULL

**rep=20000**

#rep<-100

rep.inner<-5000

theta0ref<-exp(1)

for (i in 1:rep){

set.seed(i)

meta.params<- ***{toto<-{temp<- rbinom(1,1,0.7); temp*exp(runif(1)*3.45+3)+(1-temp)* (250+750* runif(1))}; if (toto<200){toto<-round(toto,digits=-1)} else {toto<-round(toto,digits=-2)}; sigma<-2*runif(1)+0.05;***

list(J=toto, N.y=toto,sigma=(sigma))}

true.params<-(list(tau=(rgamma(1, theta0ref/meta.params$sigma, 1/meta.params$sigma))))

y<-({rpois(meta.params$J,true.params$tau)})

**#sigma0<-rlnorm(1,0,1.4)*meta.params$sigma**

**sigma0<- meta.params$sigma**

theta0<- theta0ref

**#thetatilde:**

thetatilde<-(rgamma(1, theta0/meta.params$sigma+sum(y), (1/meta.params$sigma+meta.params$J)))

#y.norm<-qnorm(ppois(y-0.1,thetatilde)+runif(length(y))*( ppois(y,thetatilde)- ppois(y-0.1,thetatilde)))

#theta<-rnorm(1,0,1)

sample.sim.norm<-sapply(1:rep.inner,function(x){toto<- rpois(meta.params$J,thetatilde); x<-toto;c(mean(x==0),mean(x[1]==0))})

toto<-c(p0= quantile.for.diags(sample.sim.norm[1,],mean(y==0)), p01= quantile.for.diags(sample.sim.norm[2,],mean(y[1]==0)))

ppp<-rbind(ppp,c(toto,musigma0=sigma0/meta.params$sigma,N=meta.params$J))

}

#1 itération=0.5"

results.dsppbis.Poisson.Nrandom.theta1.sigma2.taurandomV1.Sc1.conjugate.R<-ppp

### #nspp, Nrandom, taurandom, Scenario2, complément C, sur la station

attach("//home//frederic.gosselin//.RData",pos=2)

attach("//home//frederic.gosselin//diags//compare_diags_functions.RData",pos=2)

***load("Diags_Poisson1.RData")***

ppp<-NULL

musigma0<-NULL

rep<-100000

#rep<-50000

rep.inner<-5000

theta0ref<-exp(1)

for (i in 1:rep){

set.seed(i)

meta.params<- ***{toto<-{temp<- rbinom(1,1,0.7); temp*exp(runif(1)*3.45+3)+(1-temp)* (250+750* runif(1))}; if (toto<200){toto<-round(toto,digits=-1)} else {toto<-round(toto,digits=-2)}; sigma<-2*runif(1)+0.05;***

list(J=toto, N.y=toto,sigma=(sigma))}

true.params<-(list(tau=(rgamma(1, theta0ref/meta.params$sigma, 1/meta.params$sigma))))

y<-({rpois(meta.params$J,true.params$tau)})

**sigma0<-rlnorm(1,0,1.4)*meta.params$sigma**

**#sigma0<- meta.params$sigma**

theta0<- theta0ref

**#thetatilde:**

thetatilde<-(rgamma(1, theta0/sigma0+sum(y), (1/sigma0+meta.params$J)))

y.norm<-qnorm(ppois(y-0.1,thetatilde)+runif(length(y))*( ppois(y,thetatilde)- ppois(y-0.1,thetatilde)))

#theta<-rnorm(1,0,1)

sample.sim.norm<-sapply(1:rep.inner,function(x){toto<-rnorm(meta.params$J); toto0<-F;if (sum(!is.finite(toto))==0) {toto0=var(toto)==0} else {toto0=T};while (toto0){toto<- rnorm(meta.params$J); toto0<-F;if (sum(!is.finite(toto))==0) {toto0=var(toto)==0} else {toto0=T}}; x<-toto;c(mean(x),var(x),skewness(x),kurtosis(x),zhang(x,pnorm)$Za)})

toto<-c(mean=quantile.for.diags(sample.sim.norm[1,],mean(y.norm)),

var= quantile.for.diags(sample.sim.norm[2,],var(y.norm)),

skew= quantile.for.diags(sample.sim.norm[3,],skewness(y.norm)),

kurt= quantile.for.diags(sample.sim.norm[4,],kurtosis(y.norm)),

Za= quantile.for.diags(sample.sim.norm[5,],zhang(y.norm,pnorm)$Za))

ppp<-rbind(ppp,c(toto,musigma0=sigma0/meta.params$sigma,N=meta.params$J))

}

#1 itération=0.5"

results.nspp.Poisson.Nrandom.theta1.sigma2.taurandomV1.Sc2.conjugate.R<-ppp

***save.image("Diags_Poisson3.RData")***

### #spp, Nrandom, taurandom, Scenario2, complément C, en local

ppp<-NULL

musigma0<-NULL

rep=100000

#rep<-100

rep.inner<-5000

theta0ref<-exp(1)

for (i in 1:rep){

set.seed(i)

meta.params<- ***{toto<-{temp<- rbinom(1,1,0.7); temp*exp(runif(1)*3.45+3)+(1-temp)* (250+750* runif(1))}; if (toto<200){toto<-round(toto,digits=-1)} else {toto<-round(toto,digits=-2)}; sigma<-2*runif(1)+0.05;***

list(J=toto, N.y=toto,sigma=(sigma))}

true.params<-(list(tau=(rgamma(1, theta0ref/meta.params$sigma, 1/meta.params$sigma))))

y<-({rpois(meta.params$J,true.params$tau)})

**sigma0<-rlnorm(1,0,1.4)*meta.params$sigma**

**#sigma0<- meta.params$sigma**

theta0<- theta0ref

**#thetatilde:**

thetatilde<-(rgamma(1, theta0/sigma0+sum(y), (1/sigma0+meta.params$J)))

#y.norm<-qnorm(ppois(y-0.1,thetatilde)+runif(length(y))*( ppois(y,thetatilde)- ppois(y-0.1,thetatilde)))

#theta<-rnorm(1,0,1)

sample.sim.norm<-sapply(1:rep.inner,function(x){toto<- rpois(meta.params$J,thetatilde); x<-toto;c(mean(x),var(x))})

toto<-c(mean=quantile.for.diags(sample.sim.norm[1,],mean(y)),

var= quantile.for.diags(sample.sim.norm[2,],var(y)))

ppp<-rbind(ppp,c(toto,musigma0=sigma0/meta.params$sigma,N=meta.params$J))

}

#1 itération=0.5"

results.spp.Poisson.Nrandom.theta1.sigma2.taurandomV1.Sc2.conjugate.R<-ppp

save.image("Diags_Poisson2.RData")

### #dspp, Nrandom, taurandom, Scenario2, complément C, en local

ppp<-NULL

musigma0<-NULL

rep=100000

#rep<-100

rep.inner<-5000

theta0ref<-exp(1)

for (i in 1:rep){

set.seed(i)

meta.params<- ***{toto<-{temp<- rbinom(1,1,0.7); temp*exp(runif(1)*3.45+3)+(1-temp)* (250+750* runif(1))}; if (toto<200){toto<-round(toto,digits=-1)} else {toto<-round(toto,digits=-2)}; sigma<-2*runif(1)+0.05;***

list(J=toto, N.y=toto,sigma=(sigma))}

true.params<-(list(tau=(rgamma(1, theta0ref/meta.params$sigma, 1/meta.params$sigma))))

y<-({rpois(meta.params$J,true.params$tau)})

**sigma0<-rlnorm(1,0,1.4)*meta.params$sigma**

**#sigma0<- meta.params$sigma**

theta0<- theta0ref

**#thetatilde:**

thetatilde<-(rgamma(1, theta0/sigma0+sum(y), (1/sigma0+meta.params$J)))

#y.norm<-qnorm(ppois(y-0.1,thetatilde)+runif(length(y))*( ppois(y,thetatilde)- ppois(y-0.1,thetatilde)))

#theta<-rnorm(1,0,1)

sample.sim.norm<-sapply(1:rep.inner,function(x){toto<- rpois(meta.params$J,thetatilde); x<-toto;c(mean(x)- thetatilde,var(x)- thetatilde,mean(x==0),mean(x[1]==0))})

toto<-c(meanc=quantile.for.diags(sample.sim.norm[1,],mean(y)- thetatilde),

varc= quantile.for.diags(sample.sim.norm[2,],var(y)- thetatilde),p0= quantile.for.diags(sample.sim.norm[3,],mean(y==0)), p01= quantile.for.diags(sample.sim.norm[4,],mean(y[1]==0)))

ppp<-rbind(ppp,c(toto,musigma0=sigma0/meta.params$sigma,N=meta.params$J))

}

#1 itération=0.5"

results.dspp.Poisson.Nrandom.theta1.sigma2.taurandomV1.Sc2.conjugate.R<-ppp

save.image("Diags_Poisson2.RData")

### #dspp2, Nrandom, taurandom, Scenario2, complément C, en local

ppp<-NULL

musigma0<-NULL

rep=100000

#rep<-100

rep.inner<-5000

theta0ref<-exp(1)

for (i in 1:rep){

set.seed(i)

meta.params<- ***{toto<-{temp<- rbinom(1,1,0.7); temp*exp(runif(1)*3.45+3)+(1-temp)* (250+750* runif(1))}; if (toto<200){toto<-round(toto,digits=-1)} else {toto<-round(toto,digits=-2)}; sigma<-2*runif(1)+0.05;***

list(J=toto, N.y=toto,sigma=(sigma))}

true.params<-(list(tau=(rgamma(1, theta0ref/meta.params$sigma, 1/meta.params$sigma))))

y<-({rpois(meta.params$J,true.params$tau)})

**sigma0<-rlnorm(1,0,1.4)*meta.params$sigma**

**#sigma0<- meta.params$sigma**

theta0<- theta0ref

**#thetatilde:**

thetatilde<-(rgamma(1, theta0/sigma0+sum(y), (1/sigma0+meta.params$J)))

#y.norm<-qnorm(ppois(y-0.1,thetatilde)+runif(length(y))*( ppois(y,thetatilde)- ppois(y-0.1,thetatilde)))

#theta<-rnorm(1,0,1)

sample.sim.norm<-sapply(1:rep.inner,function(x){toto<- rpois(meta.params$J,thetatilde); x<-toto;c(sum(dpois(x,thetatilde,log=T)))})

toto<-c(LL=quantile.for.diags(sample.sim.norm, sum(dpois(y,thetatilde,log=T))))

ppp<-rbind(ppp,c(toto,musigma0=sigma0/meta.params$sigma,N=meta.params$J))

}

#1 itération=0.5"

results.dspp2.Poisson.Nrandom.theta1.sigma2.taurandomV1.Sc2.conjugate.R<-ppp

save.image("Diags_PoissonCL3.RData")

### #nspp, Nrandom, taurandom, Scenario3, complément C, en local

ppp<-NULL

musigma0<-NULL

rep=100000

#rep<-50000

rep.inner<-5000

theta0ref<-exp(1)

for (i in 1:rep){

set.seed(i)

meta.params<- ***{toto<-{temp<- rbinom(1,1,0.7); temp*exp(runif(1)*3.45+3)+(1-temp)* (250+750* runif(1))}; if (toto<200){toto<-round(toto,digits=-1)} else {toto<-round(toto,digits=-2)}; sigma<-2*runif(1)+0.05;***

list(J=toto, N.y=toto,sigma=(sigma))}

true.params<-(list(tau=(rgamma(1, theta0ref/meta.params$sigma, 1/meta.params$sigma))))

y<-({rpois(meta.params$J,true.params$tau)})

**sigma0<-rlnorm(1,0,1.4)*meta.params$sigma**

**#sigma0<- meta.params$sigma**

theta0<- exp(**rnorm(1,1,0.4))**

**#thetatilde:**

thetatilde<-(rgamma(1, theta0/sigma0+sum(y), (1/sigma0+meta.params$J)))

y.norm<-qnorm(ppois(y-0.1,thetatilde)+runif(length(y))*( ppois(y,thetatilde)- ppois(y-0.1,thetatilde)))

#theta<-rnorm(1,0,1)

sample.sim.norm<-sapply(1:rep.inner,function(x){toto<-rnorm(meta.params$J); toto0<-F;if (sum(!is.finite(toto))==0) {toto0=var(toto)==0} else {toto0=T};while (toto0){toto<- rnorm(meta.params$J); toto0<-F;if (sum(!is.finite(toto))==0) {toto0=var(toto)==0} else {toto0=T}}; x<-toto;c(mean(x),var(x),skewness(x),kurtosis(x),zhang(x,pnorm)$Za)})

toto<-c(mean=quantile.for.diags(sample.sim.norm[1,],mean(y.norm)),

var= quantile.for.diags(sample.sim.norm[2,],var(y.norm)),

skew= quantile.for.diags(sample.sim.norm[3,],skewness(y.norm)),

kurt= quantile.for.diags(sample.sim.norm[4,],kurtosis(y.norm)),

Za= quantile.for.diags(sample.sim.norm[5,],zhang(y.norm,pnorm)$Za))

ppp<-rbind(ppp,c(toto,musigma0=sigma0/meta.params$sigma,theta0=theta0,N=meta.params$J,sigma0= meta.params$sigma))

}

#1 itération=0.5"

results.nspp.Poisson.Nrandom.theta1.sigma2.taurandomV1.Sc3.conjugate.R<-ppp

***save.image("Diags_Poisson3.RData")***

### #spp, Nrandom, taurandom, Scenario3, complément C, en local

ppp<-NULL

musigma0<-NULL

rep=100000

#rep<-100

rep.inner<-5000

theta0ref<-exp(1)

for (i in 1:rep){

set.seed(i)

meta.params<- ***{toto<-{temp<- rbinom(1,1,0.7); temp*exp(runif(1)*3.45+3)+(1-temp)* (250+750* runif(1))}; if (toto<200){toto<-round(toto,digits=-1)} else {toto<-round(toto,digits=-2)}; sigma<-2*runif(1)+0.05;***

list(J=toto, N.y=toto,sigma=(sigma))}

true.params<-(list(tau=(rgamma(1, theta0ref/meta.params$sigma, 1/meta.params$sigma))))

y<-({rpois(meta.params$J,true.params$tau)})

**sigma0<-rlnorm(1,0,1.4)*meta.params$sigma**

**#sigma0<- meta.params$sigma**

theta0<- exp(**rnorm(1,1,0.4) *sqrt(sigma0))**

**#thetatilde:**

thetatilde<-(rgamma(1, theta0/sigma0+sum(y), (1/sigma0+meta.params$J)))

#y.norm<-qnorm(ppois(y-0.1,thetatilde)+runif(length(y))*( ppois(y,thetatilde)- ppois(y-0.1,thetatilde)))

#theta<-rnorm(1,0,1)

sample.sim.norm<-sapply(1:rep.inner,function(x){toto<- rpois(meta.params$J,thetatilde); x<-toto;c(mean(x),var(x))})

toto<-c(mean=quantile.for.diags(sample.sim.norm[1,],mean(y)),

var= quantile.for.diags(sample.sim.norm[2,],var(y)))

ppp<-rbind(ppp,c(toto,musigma0=sigma0/meta.params$sigma,theta0=theta0/sqrt(sigma0),theta00=theta0,N=meta.params$J, sigma0= meta.params$sigma))

}

#1 itération=0.5"

results.spp.Poisson.Nrandom.theta1.sigma2.taurandomV1.Sc3.conjugate.R<-ppp

#save.image("Diags_Poisson2.RData")

save.image("Diags_Poisson3.RData")

### #dspp, Nrandom, taurandom, Scenario3, complément C, en local

ppp<-NULL

musigma0<-NULL

rep=100000

#rep<-100

rep.inner<-5000

theta0ref<-exp(1)

for (i in 1:rep){

set.seed(i)

meta.params<- ***{toto<-{temp<- rbinom(1,1,0.7); temp*exp(runif(1)*3.45+3)+(1-temp)* (250+750* runif(1))}; if (toto<200){toto<-round(toto,digits=-1)} else {toto<-round(toto,digits=-2)}; sigma<-2*runif(1)+0.05;***

list(J=toto, N.y=toto,sigma=(sigma))}

true.params<-(list(tau=(rgamma(1, theta0ref/meta.params$sigma, 1/meta.params$sigma))))

y<-({rpois(meta.params$J,true.params$tau)})

**sigma0<-rlnorm(1,0,1.4)*meta.params$sigma**

**#sigma0<- meta.params$sigma**

theta0<- exp(**rnorm(1,1,0.4))**

**#thetatilde:**

thetatilde<-(rgamma(1, theta0/sigma0+sum(y), (1/sigma0+meta.params$J)))

#theta<-rnorm(1,0,1)

sample.sim.norm<-sapply(1:rep.inner,function(x){toto<- rpois(meta.params$J,thetatilde); x<-toto;c(mean(x)- thetatilde,var(x)- thetatilde,mean(x==0),mean(x[1]==0))})

toto<-c(meanc=quantile.for.diags(sample.sim.norm[1,],mean(y)- thetatilde),

varc= quantile.for.diags(sample.sim.norm[2,],var(y)- thetatilde),p0= quantile.for.diags(sample.sim.norm[3,],mean(y==0)), p01= quantile.for.diags(sample.sim.norm[4,],mean(y[1]==0)))

ppp<-rbind(ppp,c(toto,musigma0=sigma0/meta.params$sigma, theta0=theta0,N=meta.params$J, sigma0= meta.params$sigma))

}

#1 itération=0.5"

results.dspp.Poisson.Nrandom.theta1.sigma2.taurandomV1.Sc3.conjugate.R<-ppp

#save.image("Diags_Poisson1.RData")

save.image("Diags_Poisson3.RData")

### #dspp2, Nrandom, taurandom, Scenario3, complément C, en local

ppp<-NULL

musigma0<-NULL

rep=100000

#rep<-100

rep.inner<-5000

theta0ref<-exp(1)

for (i in 1:rep){

set.seed(i)

meta.params<- ***{toto<-{temp<- rbinom(1,1,0.7); temp*exp(runif(1)*3.45+3)+(1-temp)* (250+750* runif(1))}; if (toto<200){toto<-round(toto,digits=-1)} else {toto<-round(toto,digits=-2)}; sigma<-2*runif(1)+0.05;***

list(J=toto, N.y=toto,sigma=(sigma))}

true.params<-(list(tau=(rgamma(1, theta0ref/meta.params$sigma, 1/meta.params$sigma))))

y<-({rpois(meta.params$J,true.params$tau)})

**sigma0<-rlnorm(1,0,1.4)*meta.params$sigma**

**#sigma0<- meta.params$sigma**

theta0<- exp(**rnorm(1,1,0.4))**

**#thetatilde:**

thetatilde<-(rgamma(1, theta0/sigma0+sum(y), (1/sigma0+meta.params$J)))

#theta<-rnorm(1,0,1)

sample.sim.norm<-sapply(1:rep.inner,function(x){toto<- rpois(meta.params$J,thetatilde); x<-toto;c(sum(dpois(x,thetatilde,log=T))

)})

toto<-c(LL=quantile.for.diags(sample.sim.norm, sum(dpois(y,thetatilde,log=T))))

ppp<-rbind(ppp,c(toto,musigma0=sigma0/meta.params$sigma, theta0=theta0,N=meta.params$J,sigma0= meta.params$sigma))

}

#1 itération=0.5"

results.dspp2.Poisson.Nrandom.theta1.sigma2.taurandomV1.Sc3.conjugate.R<-ppp

#save.image("Diags_Poisson1.RData")

save.image("Diags_Poisson_CL3.RData")

### #nspp, Nrandom, taurandom, Scenario3t, complément C, en local

ppp<-NULL

musigma0<-NULL

rep=100000

#rep<-50000

rep.inner<-5000

theta0ref<-exp(1)

for (i in 1:rep){

set.seed(i)

meta.params<- ***{toto<-{temp<- rbinom(1,1,0.7); temp*exp(runif(1)*3.45+3)+(1-temp)* (250+750* runif(1))}; if (toto<200){toto<-round(toto,digits=-1)} else {toto<-round(toto,digits=-2)}; sigma<-2*runif(1)+0.05;***

list(J=toto, N.y=toto,sigma=(sigma))}

true.params<-(list(tau=(rgamma(1, theta0ref/meta.params$sigma, 1/meta.params$sigma))))

y<-({rpois(meta.params$J,true.params$tau)})

**sigma0<-rlnorm(1,0,1.4)*meta.params$sigma**

**#sigma0<- meta.params$sigma**

theta0<- exp(**rnorm(1,1,0.4)*sqrt(meta.params$sigma))**

**#thetatilde:**

thetatilde<-(rgamma(1, theta0/sigma0+sum(y), (1/sigma0+meta.params$J)))

y.norm<-qnorm(ppois(y-0.1,thetatilde)+runif(length(y))*( ppois(y,thetatilde)- ppois(y-0.1,thetatilde)))

#theta<-rnorm(1,0,1)

sample.sim.norm<-sapply(1:rep.inner,function(x){toto<-rnorm(meta.params$J); toto0<-F;if (sum(!is.finite(toto))==0) {toto0=var(toto)==0} else {toto0=T};while (toto0){toto<- rnorm(meta.params$J); toto0<-F;if (sum(!is.finite(toto))==0) {toto0=var(toto)==0} else {toto0=T}}; x<-toto;c(mean(x),var(x),skewness(x),kurtosis(x),zhang(x,pnorm)$Za)})

toto<-c(mean=quantile.for.diags(sample.sim.norm[1,],mean(y.norm)),

var= quantile.for.diags(sample.sim.norm[2,],var(y.norm)),

skew= quantile.for.diags(sample.sim.norm[3,],skewness(y.norm)),

kurt= quantile.for.diags(sample.sim.norm[4,],kurtosis(y.norm)),

Za= quantile.for.diags(sample.sim.norm[5,],zhang(y.norm,pnorm)$Za))

ppp<-rbind(ppp,c(toto,musigma0=sigma0/meta.params$sigma,theta0=exp(log(theta0)/sqrt(**meta.params$sigma**)),theta00=theta0,N=meta.params$J))

}

#1 itération=0.5"

results.nspp.Poisson.Nrandom.theta1.sigma2.taurandomV1.Sc3t.conjugate.R<-ppp

***save.image("Diags_Poisson2.RData")***

### #nspp, Nrandom, taurandom, Scenario4, complément C, en local

ppp<-NULL

musigma0<-NULL

#rep=1000

rep<-100000

rep.inner<-5000

theta0ref<-exp(1)

for (i in 1:rep){

set.seed(i)

meta.params<- ***{toto<-{temp<- rbinom(1,1,0.7); temp*exp(runif(1)*3.45+3)+(1-temp)* (250+750* runif(1))}; if (toto<200){toto<-round(toto,digits=-1)} else {toto<-round(toto,digits=-2)}; sigma<-2*runif(1)+0.05;***

list(J=toto, N.y=toto,sigma=(sigma))}

true.params<-(list(tau=(rgamma(1, theta0ref/meta.params$sigma, 1/meta.params$sigma))))

y<-({rpois(meta.params$J, theta0ref)})

**#sigma0<-rlnorm(1,0,1.4)*meta.params$sigma**

**sigma0<- meta.params$sigma**

theta0<- theta0ref

**#thetatilde:**

thetatilde<-(rgamma(1, theta0/meta.params$sigma+sum(y), (1/meta.params$sigma+meta.params$J)))

y.norm<-qnorm(ppois(y-0.1,thetatilde)+runif(length(y))*( ppois(y,thetatilde)- ppois(y-0.1,thetatilde)))

#theta<-rnorm(1,0,1)

sample.sim.norm<-sapply(1:rep.inner,function(x){toto<-rnorm(meta.params$J); toto0<-F;if (sum(!is.finite(toto))==0) {toto0=var(toto)==0} else {toto0=T};while (toto0){toto<- rnorm(meta.params$J); toto0<-F;if (sum(!is.finite(toto))==0) {toto0=var(toto)==0} else {toto0=T}}; x<-toto;c(mean(x),var(x),skewness(x),kurtosis(x),zhang(x,pnorm)$Za)})

toto<-c(mean=quantile.for.diags(sample.sim.norm[1,],mean(y.norm)),

var= quantile.for.diags(sample.sim.norm[2,],var(y.norm)),

skew= quantile.for.diags(sample.sim.norm[3,],skewness(y.norm)),

kurt= quantile.for.diags(sample.sim.norm[4,],kurtosis(y.norm)),

Za= quantile.for.diags(sample.sim.norm[5,],zhang(y.norm,pnorm)$Za))

ppp<-rbind(ppp,c(toto,sigma0=sigma0,N=meta.params$J))

}

#1 itération=0.5"

results.nspp.Poisson.Nrandom.theta1.sigma2.taurandomV1.Sc4.conjugate.R<-ppp

***save.image("Diags_Poisson2.RData")***

### #spp, Nrandom, taurandom, Scenario4, complément C

ppp<-NULL

musigma0<-NULL

rep=100000

#rep<-100

rep.inner<-5000

theta0ref<-exp(1)

for (i in 1:rep){

set.seed(i)

meta.params<- ***{toto<-{temp<- rbinom(1,1,0.7); temp*exp(runif(1)*3.45+3)+(1-temp)* (250+750* runif(1))}; if (toto<200){toto<-round(toto,digits=-1)} else {toto<-round(toto,digits=-2)}; sigma<-2*runif(1)+0.05;***

list(J=toto, N.y=toto,sigma=(sigma))}

true.params<-(list(tau=(rgamma(1, theta0ref/meta.params$sigma, 1/meta.params$sigma))))

y<-({rpois(meta.params$J,theta0ref)})

**#sigma0<-rlnorm(1,0,1.4)*meta.params$sigma**

**sigma0<- meta.params$sigma**

theta0<- theta0ref

**#thetatilde:**

thetatilde<-(rgamma(1, theta0/meta.params$sigma+sum(y), (1/meta.params$sigma+meta.params$J)))

#y.norm<-qnorm(ppois(y-0.1,thetatilde)+runif(length(y))*( ppois(y,thetatilde)- ppois(y-0.1,thetatilde)))

#theta<-rnorm(1,0,1)

sample.sim.norm<-sapply(1:rep.inner,function(x){toto<- rpois(meta.params$J,thetatilde); x<-toto;c(mean(x),var(x))})

toto<-c(mean=quantile.for.diags(sample.sim.norm[1,],mean(y)),

var= quantile.for.diags(sample.sim.norm[2,],var(y)))

ppp<-rbind(ppp,c(toto,sigma0=meta.params$sigma,N=meta.params$J))

}

#1 itération=0.5"

results.spp.Poisson.Nrandom.theta1.sigma2.taurandomV1.Sc4.conjugate.R<-ppp

***save.image("Diags_Poisson2.RData")***

### #dspp, Nrandom, taurandom, Scenario4, complément C

ppp<-NULL

musigma0<-NULL

rep=100000

#rep<-100

rep.inner<-5000

theta0ref<-exp(1)

for (i in 1:rep){

set.seed(i)

meta.params<- ***{toto<-{temp<- rbinom(1,1,0.7); temp*exp(runif(1)*3.45+3)+(1-temp)* (250+750* runif(1))}; if (toto<200){toto<-round(toto,digits=-1)} else {toto<-round(toto,digits=-2)}; sigma<-2*runif(1)+0.05;***

list(J=toto, N.y=toto,sigma=(sigma))}

true.params<-(list(tau=(rgamma(1, theta0ref/meta.params$sigma, 1/meta.params$sigma))))

y<-({rpois(meta.params$J,theta0ref)})

**#sigma0<-rlnorm(1,0,1.4)*meta.params$sigma**

**sigma0<- meta.params$sigma**

theta0<- theta0ref

**#thetatilde:**

thetatilde<-(rgamma(1, theta0/meta.params$sigma+sum(y), (1/meta.params$sigma+meta.params$J)))

#y.norm<-qnorm(ppois(y-0.1,thetatilde)+runif(length(y))*( ppois(y,thetatilde)- ppois(y-0.1,thetatilde)))

#theta<-rnorm(1,0,1)

sample.sim.norm<-sapply(1:rep.inner,function(x){toto<- rpois(meta.params$J,thetatilde); x<-toto;c(mean(x)- thetatilde,var(x)- thetatilde,mean(x==0),mean(x[1]==0))})

toto<-c(meanc=quantile.for.diags(sample.sim.norm[1,],mean(y)- thetatilde),

varc= quantile.for.diags(sample.sim.norm[2,],var(y)- thetatilde),p0= quantile.for.diags(sample.sim.norm[3,],mean(y==0)), p01= quantile.for.diags(sample.sim.norm[4,],mean(y[1]==0)))

ppp<-rbind(ppp,c(toto,sigma0=sigma0,N=meta.params$J))

}

#1 itération=0.5"

results.dspp.Poisson.Nrandom.theta1.sigma2.taurandomV1.Sc4.conjugate.R<-ppp

***save.image("Diags_Poisson2.RData")***

### #dspp2, Nrandom, taurandom, Scenario4, complément C

ppp<-NULL

musigma0<-NULL

rep=100000

#rep<-100

rep.inner<-5000

theta0ref<-exp(1)

for (i in 1:rep){

set.seed(i)

meta.params<- ***{toto<-{temp<- rbinom(1,1,0.7); temp*exp(runif(1)*3.45+3)+(1-temp)* (250+750* runif(1))}; if (toto<200){toto<-round(toto,digits=-1)} else {toto<-round(toto,digits=-2)}; sigma<-2*runif(1)+0.05;***

list(J=toto, N.y=toto,sigma=(sigma))}

true.params<-(list(tau=(rgamma(1, theta0ref/meta.params$sigma, 1/meta.params$sigma))))

y<-({rpois(meta.params$J,theta0ref)})

**#sigma0<-rlnorm(1,0,1.4)*meta.params$sigma**

**sigma0<- meta.params$sigma**

theta0<- theta0ref

**#thetatilde:**

thetatilde<-(rgamma(1, theta0/meta.params$sigma+sum(y), (1/meta.params$sigma+meta.params$J)))

#y.norm<-qnorm(ppois(y-0.1,thetatilde)+runif(length(y))*( ppois(y,thetatilde)- ppois(y-0.1,thetatilde)))

#theta<-rnorm(1,0,1)

sample.sim.norm<-sapply(1:rep.inner,function(x){toto<- rpois(meta.params$J,thetatilde); x<-toto;c(sum(dpois(x,thetatilde,log=T)))})

toto<-c(LL=quantile.for.diags(sample.sim.norm, sum(dpois(y,thetatilde,log=T))))

ppp<-rbind(ppp,c(toto,sigma0=sigma0,N=meta.params$J))

}

#1 itération=0.5"

results.dspp2.Poisson.Nrandom.theta1.sigma2.taurandomV1.Sc4.conjugate.R<-ppp

***save.image("Diags_Poisson_CL3.RData")***

### #spp.gen, Nrandom, taurandom, Scenario4, complément C

ppp<-NULL

musigma0<-NULL

rep=40000

#rep<-20

rep.inner<-5000

theta0ref<-exp(1)

for (i in 1:rep){

set.seed(i)

meta.params<- ***{toto<- sample(c(20,50),1); sigma<-2*runif(1)+0.05;nmax<-sample(c(4,5),1);***

list(J=toto, N.y=toto,sigma=(sigma),nmax=nmax)}

true.params<-(list(tau=(rgamma(1, theta0ref/meta.params$sigma, 1/meta.params$sigma))))

y<-({rpois(meta.params$J,theta0ref)})

**#sigma0<-rlnorm(1,0,1.4)*meta.params$sigma**

**sigma0<- meta.params$sigma**

theta0<- theta0ref

**#thetatilde:**

thetatilde<-(rgamma(1, theta0/meta.params$sigma+sum(y), (1/meta.params$sigma+meta.params$J)))

#y.norm<-qnorm(ppois(y-0.1,thetatilde)+runif(length(y))*( ppois(y,thetatilde)- ppois(y-0.1,thetatilde)))

y.norm<-qnorm(ppois(y-0.1,thetatilde)+runif(length(y))*( ppois(y,thetatilde)- ppois(y-0.1,thetatilde)))

#theta<-rnorm(1,0,1)

sample.sim.norm<-sapply(1:rep.inner,function(x){toto<-rnorm(meta.params$J); toto0<-F;if (sum(!is.finite(toto))==0) {toto0=var(toto)==0} else {toto0=T};while (toto0){toto<- rnorm(meta.params$J); toto0<-F;if (sum(!is.finite(toto))==0) {toto0=var(toto)==0} else {toto0=T}}; x<-toto;c(mean(x),var(x),skewness(x),kurtosis(x),zhang(x,pnorm)$Za,max(x))})

toto<-c(mean.norm=quantile.for.diags(sample.sim.norm[1,],mean(y.norm)),

var.norm= quantile.for.diags(sample.sim.norm[2,],var(y.norm)),

skew.norm= quantile.for.diags(sample.sim.norm[3,],skewness(y.norm)),

kurt.norm= quantile.for.diags(sample.sim.norm[4,],kurtosis(y.norm)),

max.norm= quantile.for.diags(sample.sim.norm[6,],max(y.norm)),

Za.norm= quantile.for.diags(sample.sim.norm[5,],zhang(y.norm,pnorm)$Za))

#theta<-rnorm(1,0,1)

sample.sim.norm<-sapply(1:rep.inner,function(x){toto<- rpois(meta.params$J,thetatilde); x<-toto;c(mean(x),var(x),max(x))})

toto2<-c(mean=quantile.for.diags(sample.sim.norm[1,],mean(y)),

var= quantile.for.diags(sample.sim.norm[2,],var(y)),

max= quantile.for.diags(sample.sim.norm[3,],max(y)))

ppp<-rbind(ppp,c(toto,toto2,sigma0=meta.params$sigma,N=meta.params$J,nmax= meta.params$nmax))

}

#1 itération=0.5"

results.spp.Poisson.Nrandom.theta1.sigma2.taurandomV1.Sc4.conjugate.R<-ppp

***save.image("Diags_Poisson3.RData")***

### #ppp.gen, Nrandom, taurandom, Scenario4, complément C

ppp<-NULL

musigma0<-NULL

rep=40000

#rep<-20

rep.inner<-5000

theta0ref<-exp(1)

for (i in 1:rep){

set.seed(i)

meta.params<- ***{toto<- sample(c(20,50),1); sigma<-2*runif(1)+0.05;nmax<-sample(c(4,5),1);***

list(J=toto, N.y=toto,sigma=(sigma),nmax=nmax)}

true.params<-(list(tau=(rgamma(1, theta0ref/meta.params$sigma, 1/meta.params$sigma))))

y<-({rpois(meta.params$J,theta0ref)})

**#sigma0<-rlnorm(1,0,1.4)*meta.params$sigma**

**sigma0<- meta.params$sigma**

theta0<- theta0ref

**#thetatilde:**

#theta<-rnorm(1,0,1)

sample.sim.norm<-sapply(1:rep.inner,function(x){toto<-rnorm(meta.params$J); toto0<-F;if (sum(!is.finite(toto))==0) {toto0=var(toto)==0} else {toto0=T};while (toto0){toto<- rnorm(meta.params$J); toto0<-F;if (sum(!is.finite(toto))==0) {toto0=var(toto)==0} else {toto0=T}}; x<-toto; thetatilde<-(rgamma(1, theta0/meta.params$sigma+sum(y), (1/meta.params$sigma+meta.params$J)));

y.norm<-qnorm(ppois(y-0.1,thetatilde)+runif(length(y))*( ppois(y,thetatilde)- ppois(y-0.1,thetatilde)));

c(mean(x),var(x),skewness(x),kurtosis(x),zhang(x,pnorm)$Za,max(x), mean(y.norm), var(y.norm), skewness(y.norm), kurtosis(y.norm), zhang(y.norm,pnorm)$Za, max(y.norm))})

toto<-c(mean.norm=quantile.for.diags(sample.sim.norm[1,], sample.sim.norm[7,]),

var.norm= quantile.for.diags(sample.sim.norm[2,], sample.sim.norm[8,]),

skew.norm= quantile.for.diags(sample.sim.norm[3,], sample.sim.norm[9,]),

kurt.norm= quantile.for.diags(sample.sim.norm[4,], sample.sim.norm[10,]),

max.norm= quantile.for.diags(sample.sim.norm[6,], sample.sim.norm[12,]),

Za.norm= quantile.for.diags(sample.sim.norm[5,], sample.sim.norm[11,]))

#theta<-rnorm(1,0,1)

sample.sim.norm<-sapply(1:rep.inner,function(x){ thetatilde<-(rgamma(1, theta0/meta.params$sigma+sum(y), (1/meta.params$sigma+meta.params$J)));

toto<- rpois(meta.params$J,thetatilde); x<-toto;c(mean(x),var(x),max(x))})

toto2<-c(mean=quantile.for.diags(sample.sim.norm[1,],mean(y)),

var= quantile.for.diags(sample.sim.norm[2,],var(y)),

max= quantile.for.diags(sample.sim.norm[3,],max(y)))

ppp<-rbind(ppp,c(toto,toto2,sigma0=meta.params$sigma,N=meta.params$J,nmax= meta.params$nmax))

}

#1 itération=0.5"

results.ppp.Poisson.Nrandom.theta1.sigma2.taurandomV1.Sc4.conjugate.R<-ppp

***save.image("Diags_Poisson_CL.RData")***

### #hsMLp.gen, Nrandom, taurandom, Scenario4, complément C

ppp<-NULL

musigma0<-NULL

rep=40000

#rep<-20

rep.inner<-5000

theta0ref<-exp(1)

for (i in 1:rep){

set.seed(i)

meta.params<- ***{toto<- sample(c(20,50),1); sigma<-2*runif(1)+0.05;nmax<-sample(c(4,5),1);***

list(J=toto, N.y=toto,sigma=(sigma),nmax=nmax)}

true.params<-(list(tau=(rgamma(1, theta0ref/meta.params$sigma, 1/meta.params$sigma))))

y<-({rpois(meta.params$J,theta0ref)})

**#sigma0<-rlnorm(1,0,1.4)*meta.params$sigma**

**sigma0<- meta.params$sigma**

theta0<- theta0ref

**#thetatilde:**

#thetatilde<-(rgamma(1, theta0/meta.params$sigma+sum(y), (1/meta.params$sigma+meta.params$J)))

yb<-sample(y,floor(length(y)/2),replace=F)

glm.refb<-glm(yb~1,family="poisson")

thetatilde<- exp(glm.refb$coef)

#y.norm<-qnorm(ppois(y-0.1,thetatilde)+runif(length(y))*( ppois(y,thetatilde)- ppois(y-0.1,thetatilde)))

y.norm<-qnorm(ppois(y-0.1,thetatilde)+runif(length(y))*( ppois(y,thetatilde)- ppois(y-0.1,thetatilde)))

#theta<-rnorm(1,0,1)

sample.sim.norm<-sapply(1:rep.inner,function(x){toto<-rnorm(meta.params$J); toto0<-F;if (sum(!is.finite(toto))==0) {toto0=var(toto)==0} else {toto0=T};while (toto0){toto<- rnorm(meta.params$J); toto0<-F;if (sum(!is.finite(toto))==0) {toto0=var(toto)==0} else {toto0=T}}; x<-toto;c(mean(x),var(x),skewness(x),kurtosis(x),zhang(x,pnorm)$Za,max(x))})

toto<-c(mean.norm=quantile.for.diags(sample.sim.norm[1,],mean(y.norm)),

var.norm= quantile.for.diags(sample.sim.norm[2,],var(y.norm)),

skew.norm= quantile.for.diags(sample.sim.norm[3,],skewness(y.norm)),

kurt.norm= quantile.for.diags(sample.sim.norm[4,],kurtosis(y.norm)),

max.norm= quantile.for.diags(sample.sim.norm[6,],max(y.norm)),

Za.norm= quantile.for.diags(sample.sim.norm[5,],zhang(y.norm,pnorm)$Za))

#theta<-rnorm(1,0,1)

sample.sim.norm<-sapply(1:rep.inner,function(x){toto<- rpois(meta.params$J,thetatilde); x<-toto;c(mean(x),var(x),max(x))})

toto2<-c(mean=quantile.for.diags(sample.sim.norm[1,],mean(y)),

var= quantile.for.diags(sample.sim.norm[2,],var(y)),

max= quantile.for.diags(sample.sim.norm[3,],max(y)))

ppp<-rbind(ppp,c(toto,toto2,sigma0=meta.params$sigma,N=meta.params$J,nmax= meta.params$nmax))

}

#1 itération=0.5"

results.hsMLp.Poisson.Nrandom.theta1.sigma2.taurandomV1.Sc4.conjugate.R<-ppp

***save.image("Diags_Poisson1.RData")***

### #spp.gen, Nrandom, taurandom, Scenario6 (=Sc4 + polya), complément C

ppp<-NULL

musigma0<-NULL

rep=40000

#rep<-20

rep.inner<-5000

theta0ref<-exp(1)

for (i in 1:rep){

set.seed(i)

meta.params<- ***{toto<- sample(c(20,50),1); sigma<-2*runif(1)+0.05;nmax<-sample(c(4,5),1);***

list(J=toto, N.y=toto,sigma=(sigma),nmax=nmax)}

true.params<-(list(tau=(rgamma(1, theta0ref/meta.params$sigma, 1/meta.params$sigma))))

y<-({rpolya.musizen(meta.params$J,theta0ref,1, meta.params$nmax)})

**#sigma0<-rlnorm(1,0,1.4)*meta.params$sigma**

**sigma0<- meta.params$sigma**

theta0<- theta0ref

**#thetatilde:**

thetatilde<-(rgamma(1, theta0/meta.params$sigma+sum(y), (1/meta.params$sigma+meta.params$J)))

#y.norm<-qnorm(ppois(y-0.1,thetatilde)+runif(length(y))*( ppois(y,thetatilde)- ppois(y-0.1,thetatilde)))

y.norm<-qnorm(ppois(y-0.1,thetatilde)+runif(length(y))*( ppois(y,thetatilde)- ppois(y-0.1,thetatilde)))

#theta<-rnorm(1,0,1)

sample.sim.norm<-sapply(1:rep.inner,function(x){toto<-rnorm(meta.params$J); toto0<-F;if (sum(!is.finite(toto))==0) {toto0=var(toto)==0} else {toto0=T};while (toto0){toto<- rnorm(meta.params$J); toto0<-F;if (sum(!is.finite(toto))==0) {toto0=var(toto)==0} else {toto0=T}}; x<-toto;c(mean(x),var(x),skewness(x),kurtosis(x),zhang(x,pnorm)$Za,max(x))})

toto<-c(mean.norm=quantile.for.diags(sample.sim.norm[1,],mean(y.norm)),

var.norm= quantile.for.diags(sample.sim.norm[2,],var(y.norm)),

skew.norm= quantile.for.diags(sample.sim.norm[3,],skewness(y.norm)),

kurt.norm= quantile.for.diags(sample.sim.norm[4,],kurtosis(y.norm)),

max.norm= quantile.for.diags(sample.sim.norm[6,],max(y.norm)),

Za.norm= quantile.for.diags(sample.sim.norm[5,],zhang(y.norm,pnorm)$Za))

#theta<-rnorm(1,0,1)

sample.sim.norm<-sapply(1:rep.inner,function(x){toto<- rpois(meta.params$J,thetatilde); x<-toto;c(mean(x),var(x),max(x))})

toto2<-c(mean=quantile.for.diags(sample.sim.norm[1,],mean(y)),

var= quantile.for.diags(sample.sim.norm[2,],var(y)),

max= quantile.for.diags(sample.sim.norm[3,],max(y)))

ppp<-rbind(ppp,c(toto,toto2,sigma0=meta.params$sigma,N=meta.params$J,nmax= meta.params$nmax))

}

#1 itération=0.5"

results.spp.Poisson.Nrandom.theta1.sigma2.taurandomV1.Sc6.conjugate.R<-ppp

***save.image("Diags_Poisson_CL3.RData")***

### #spp2.gen, Nrandom, taurandom, Scenario6 (=Sc4 + polya), complément C

ppp<-NULL

musigma0<-NULL

rep=40000

#rep<-20

rep.inner<-5000

theta0ref<-exp(1)

for (i in 1:rep){

set.seed(i)

meta.params<- ***{toto<- sample(c(20,30,40,50,60,70,80),1); sigma<-2*runif(1)+0.05;nmax<-sample(c(5,5),1);***

list(J=toto, N.y=toto,sigma=(sigma),nmax=nmax)}

true.params<-(list(tau=(rgamma(1, theta0ref/meta.params$sigma, 1/meta.params$sigma))))

y<-({rpolya.musizen(meta.params$J,theta0ref,1, meta.params$nmax)})

**#sigma0<-rlnorm(1,0,1.4)*meta.params$sigma**

**sigma0<- meta.params$sigma**

theta0<- theta0ref

**#thetatilde:**

thetatilde<-(rgamma(1, theta0/meta.params$sigma+sum(y), (1/meta.params$sigma+meta.params$J)))

#y.norm<-qnorm(ppois(y-0.1,thetatilde)+runif(length(y))*( ppois(y,thetatilde)- ppois(y-0.1,thetatilde)))

y.norm<-qnorm(ppois(y-0.1,thetatilde)+runif(length(y))*( ppois(y,thetatilde)- ppois(y-0.1,thetatilde)))

#theta<-rnorm(1,0,1)

sample.sim.norm<-sapply(1:rep.inner,function(x){toto<-rnorm(meta.params$J); toto0<-F;if (sum(!is.finite(toto))==0) {toto0=var(toto)==0} else {toto0=T};while (toto0){toto<- rnorm(meta.params$J); toto0<-F;if (sum(!is.finite(toto))==0) {toto0=var(toto)==0} else {toto0=T}}; x<-toto;c(mean(x),var(x),skewness(x),kurtosis(x),zhang(x,pnorm)$Za,max(x))})

toto<-c(mean.norm=quantile.for.diags(sample.sim.norm[1,],mean(y.norm)),

var.norm= quantile.for.diags(sample.sim.norm[2,],var(y.norm)),

skew.norm= quantile.for.diags(sample.sim.norm[3,],skewness(y.norm)),

kurt.norm= quantile.for.diags(sample.sim.norm[4,],kurtosis(y.norm)),

max.norm= quantile.for.diags(sample.sim.norm[6,],max(y.norm)),

Za.norm= quantile.for.diags(sample.sim.norm[5,],zhang(y.norm,pnorm)$Za))

#theta<-rnorm(1,0,1)

sample.sim.norm<-sapply(1:rep.inner,function(x){toto<- rpois(meta.params$J,thetatilde); x<-toto;c(mean(x),var(x),max(x))})

toto2<-c(mean=quantile.for.diags(sample.sim.norm[1,],mean(y)),

var= quantile.for.diags(sample.sim.norm[2,],var(y)),

max= quantile.for.diags(sample.sim.norm[3,],max(y)))

ppp<-rbind(ppp,c(toto,toto2,sigma0=meta.params$sigma,N=meta.params$J,nmax= meta.params$nmax))

}

#1 itération=0.5"

results.spp2.Poisson.Nrandom.theta1.sigma2.taurandomV1.Sc6.conjugate.R<-ppp

***save.image("Diags_Poisson_CL3.RData")***

### #ppp.gen, Nrandom, taurandom, Scenario6 (=Sc4 + polya), complément C

ppp<-NULL

musigma0<-NULL

rep=40000

#rep<-20

rep.inner<-5000

theta0ref<-exp(1)

for (i in 1:rep){

set.seed(i)

meta.params<- ***{toto<- sample(c(20,50),1); sigma<-2*runif(1)+0.05;nmax<-sample(c(4,5),1);***

list(J=toto, N.y=toto,sigma=(sigma),nmax=nmax)}

true.params<-(list(tau=(rgamma(1, theta0ref/meta.params$sigma, 1/meta.params$sigma))))

y<-({rpolya.musizen(meta.params$J,theta0ref,1, meta.params$nmax)})

**#sigma0<-rlnorm(1,0,1.4)*meta.params$sigma**

**sigma0<- meta.params$sigma**

theta0<- theta0ref

**#thetatilde:**

#theta<-rnorm(1,0,1)

sample.sim.norm<-sapply(1:rep.inner,function(x){toto<-rnorm(meta.params$J); toto0<-F;if (sum(!is.finite(toto))==0) {toto0=var(toto)==0} else {toto0=T};while (toto0){toto<- rnorm(meta.params$J); toto0<-F;if (sum(!is.finite(toto))==0) {toto0=var(toto)==0} else {toto0=T}}; x<-toto; thetatilde<-(rgamma(1, theta0/meta.params$sigma+sum(y), (1/meta.params$sigma+meta.params$J)));

y.norm<-qnorm(ppois(y-0.1,thetatilde)+runif(length(y))*( ppois(y,thetatilde)- ppois(y-0.1,thetatilde)));

c(mean(x),var(x),skewness(x),kurtosis(x),zhang(x,pnorm)$Za,max(x), mean(y.norm), var(y.norm), skewness(y.norm), kurtosis(y.norm), zhang(y.norm,pnorm)$Za, max(y.norm))})

toto<-c(mean.norm=quantile.for.diags(sample.sim.norm[1,], sample.sim.norm[7,]),

var.norm= quantile.for.diags(sample.sim.norm[2,], sample.sim.norm[8,]),

skew.norm= quantile.for.diags(sample.sim.norm[3,], sample.sim.norm[9,]),

kurt.norm= quantile.for.diags(sample.sim.norm[4,], sample.sim.norm[10,]),

max.norm= quantile.for.diags(sample.sim.norm[6,], sample.sim.norm[12,]),

Za.norm= quantile.for.diags(sample.sim.norm[5,], sample.sim.norm[11,]))

#theta<-rnorm(1,0,1)

sample.sim.norm<-sapply(1:rep.inner,function(x){ thetatilde<-(rgamma(1, theta0/meta.params$sigma+sum(y), (1/meta.params$sigma+meta.params$J)));

toto<- rpois(meta.params$J,thetatilde); x<-toto;c(mean(x),var(x),max(x))})

toto2<-c(mean=quantile.for.diags(sample.sim.norm[1,],mean(y)),

var= quantile.for.diags(sample.sim.norm[2,],var(y)),

max= quantile.for.diags(sample.sim.norm[3,],max(y)))

ppp<-rbind(ppp,c(toto,toto2,sigma0=meta.params$sigma,N=meta.params$J,nmax= meta.params$nmax))

}

#1 itération=0.5"

results.ppp.Poisson.Nrandom.theta1.sigma2.taurandomV1.Sc6.conjugate.R<-ppp

***save.image("Diags_Poisson_CL2.RData")***

### #ppp2.gen, Nrandom, taurandom, Scenario6 (=Sc4 + polya), complément C

ppp<-NULL

musigma0<-NULL

rep=40000

#rep<-20

rep.inner<-5000

theta0ref<-exp(1)

for (i in 1:rep){

set.seed(i)

meta.params<- ***{toto<- sample(c(20,30,40,50,60,70,80),1); sigma<-2*runif(1)+0.05;nmax<-sample(c(5,5),1);***

list(J=toto, N.y=toto,sigma=(sigma),nmax=nmax)}

true.params<-(list(tau=(rgamma(1, theta0ref/meta.params$sigma, 1/meta.params$sigma))))

y<-({rpolya.musizen(meta.params$J,theta0ref,1, meta.params$nmax)})

**#sigma0<-rlnorm(1,0,1.4)*meta.params$sigma**

**sigma0<- meta.params$sigma**

theta0<- theta0ref

**#thetatilde:**

#theta<-rnorm(1,0,1)

sample.sim.norm<-sapply(1:rep.inner,function(x){toto<-rnorm(meta.params$J); toto0<-F;if (sum(!is.finite(toto))==0) {toto0=var(toto)==0} else {toto0=T};while (toto0){toto<- rnorm(meta.params$J); toto0<-F;if (sum(!is.finite(toto))==0) {toto0=var(toto)==0} else {toto0=T}}; x<-toto; thetatilde<-(rgamma(1, theta0/meta.params$sigma+sum(y), (1/meta.params$sigma+meta.params$J)));

y.norm<-qnorm(ppois(y-0.1,thetatilde)+runif(length(y))*( ppois(y,thetatilde)- ppois(y-0.1,thetatilde)));

c(mean(x),var(x),skewness(x),kurtosis(x),zhang(x,pnorm)$Za,max(x), mean(y.norm), var(y.norm), skewness(y.norm), kurtosis(y.norm), zhang(y.norm,pnorm)$Za, max(y.norm))})

toto<-c(mean.norm=quantile.for.diags(sample.sim.norm[1,], sample.sim.norm[7,]),

var.norm= quantile.for.diags(sample.sim.norm[2,], sample.sim.norm[8,]),

skew.norm= quantile.for.diags(sample.sim.norm[3,], sample.sim.norm[9,]),

kurt.norm= quantile.for.diags(sample.sim.norm[4,], sample.sim.norm[10,]),

max.norm= quantile.for.diags(sample.sim.norm[6,], sample.sim.norm[12,]),

Za.norm= quantile.for.diags(sample.sim.norm[5,], sample.sim.norm[11,]))

#theta<-rnorm(1,0,1)

sample.sim.norm<-sapply(1:rep.inner,function(x){ thetatilde<-(rgamma(1, theta0/meta.params$sigma+sum(y), (1/meta.params$sigma+meta.params$J)));

toto<- rpois(meta.params$J,thetatilde); x<-toto;c(mean(x),var(x),max(x))})

toto2<-c(mean=quantile.for.diags(sample.sim.norm[1,],mean(y)),

var= quantile.for.diags(sample.sim.norm[2,],var(y)),

max= quantile.for.diags(sample.sim.norm[3,],max(y)))

ppp<-rbind(ppp,c(toto,toto2,sigma0=meta.params$sigma,N=meta.params$J,nmax= meta.params$nmax))

}

#1 itération=0.5"

results.ppp2.Poisson.Nrandom.theta1.sigma2.taurandomV1.Sc6.conjugate.R<-ppp

***save.image("Diags_Poisson_CL2.RData")***

### #hsMLp.gen, Nrandom, taurandom, Scenario6 (=Sc4 + polya), complément C

ppp<-NULL

musigma0<-NULL

rep=40000

#rep<-20

rep.inner<-5000

theta0ref<-exp(1)

for (i in 1:rep){

set.seed(i)

meta.params<- ***{toto<- sample(c(20,50),1); sigma<-2*runif(1)+0.05;nmax<-sample(c(4,5),1);***

list(J=toto, N.y=toto,sigma=(sigma),nmax=nmax)}

true.params<-(list(tau=(rgamma(1, theta0ref/meta.params$sigma, 1/meta.params$sigma))))

y<-({rpolya.musizen(meta.params$J,theta0ref,1, meta.params$nmax)})

**#sigma0<-rlnorm(1,0,1.4)*meta.params$sigma**

**sigma0<- meta.params$sigma**

theta0<- theta0ref

**#thetatilde:**

yb<-sample(y,floor(length(y)/2),replace=F)

glm.refb<-glm(yb~1,family="poisson")

thetatilde<- exp(glm.refb$coef)

#y.norm<-qnorm(ppois(y-0.1,thetatilde)+runif(length(y))*( ppois(y,thetatilde)- ppois(y-0.1,thetatilde)))

y.norm<-qnorm(ppois(y-0.1,thetatilde)+runif(length(y))*( ppois(y,thetatilde)- ppois(y-0.1,thetatilde)))

#theta<-rnorm(1,0,1)

sample.sim.norm<-sapply(1:rep.inner,function(x){toto<-rnorm(meta.params$J); toto0<-F;if (sum(!is.finite(toto))==0) {toto0=var(toto)==0} else {toto0=T};while (toto0){toto<- rnorm(meta.params$J); toto0<-F;if (sum(!is.finite(toto))==0) {toto0=var(toto)==0} else {toto0=T}}; x<-toto;c(mean(x),var(x),skewness(x),kurtosis(x),zhang(x,pnorm)$Za,max(x))})

toto<-c(mean.norm=quantile.for.diags(sample.sim.norm[1,],mean(y.norm)),

var.norm= quantile.for.diags(sample.sim.norm[2,],var(y.norm)),

skew.norm= quantile.for.diags(sample.sim.norm[3,],skewness(y.norm)),

kurt.norm= quantile.for.diags(sample.sim.norm[4,],kurtosis(y.norm)),

max.norm= quantile.for.diags(sample.sim.norm[6,],max(y.norm)),

Za.norm= quantile.for.diags(sample.sim.norm[5,],zhang(y.norm,pnorm)$Za))

#theta<-rnorm(1,0,1)

sample.sim.norm<-sapply(1:rep.inner,function(x){toto<- rpois(meta.params$J,thetatilde); x<-toto;c(mean(x),var(x),max(x))})

toto2<-c(mean=quantile.for.diags(sample.sim.norm[1,],mean(y)),

var= quantile.for.diags(sample.sim.norm[2,],var(y)),

max= quantile.for.diags(sample.sim.norm[3,],max(y)))

ppp<-rbind(ppp,c(toto,toto2,sigma0=meta.params$sigma,N=meta.params$J,nmax= meta.params$nmax))

}

#1 itération=0.5"

results.hsMLp.Poisson.Nrandom.theta1.sigma2.taurandomV1.Sc6.conjugate.R<-ppp

***save.image("Diags_Poisson2.RData")***

### #hsMLp2.gen, Nrandom, taurandom, Scenario6 (=Sc4 + polya), complément C

ppp<-NULL

musigma0<-NULL

rep=40000

#rep<-20

rep.inner<-5000

theta0ref<-exp(1)

for (i in 1:rep){

set.seed(i)

meta.params<- ***{toto<- sample(c(20,30,40,50,60,70,80),1); sigma<-2*runif(1)+0.05;nmax<-sample(c(5,5),1);***

list(J=toto, N.y=toto,sigma=(sigma),nmax=nmax)}

true.params<-(list(tau=(rgamma(1, theta0ref/meta.params$sigma, 1/meta.params$sigma))))

y<-({rpolya.musizen(meta.params$J,theta0ref,1, meta.params$nmax)})

**#sigma0<-rlnorm(1,0,1.4)*meta.params$sigma**

**sigma0<- meta.params$sigma**

theta0<- theta0ref

**#thetatilde:**

yb<-sample(y,floor(length(y)/2),replace=F)

glm.refb<-glm(yb~1,family="poisson")

thetatilde<- exp(glm.refb$coef)

#y.norm<-qnorm(ppois(y-0.1,thetatilde)+runif(length(y))*( ppois(y,thetatilde)- ppois(y-0.1,thetatilde)))

y.norm<-qnorm(ppois(y-0.1,thetatilde)+runif(length(y))*( ppois(y,thetatilde)- ppois(y-0.1,thetatilde)))

#theta<-rnorm(1,0,1)

sample.sim.norm<-sapply(1:rep.inner,function(x){toto<-rnorm(meta.params$J); toto0<-F;if (sum(!is.finite(toto))==0) {toto0=var(toto)==0} else {toto0=T};while (toto0){toto<- rnorm(meta.params$J); toto0<-F;if (sum(!is.finite(toto))==0) {toto0=var(toto)==0} else {toto0=T}}; x<-toto;c(mean(x),var(x),skewness(x),kurtosis(x),zhang(x,pnorm)$Za,max(x))})

toto<-c(mean.norm=quantile.for.diags(sample.sim.norm[1,],mean(y.norm)),

var.norm= quantile.for.diags(sample.sim.norm[2,],var(y.norm)),

skew.norm= quantile.for.diags(sample.sim.norm[3,],skewness(y.norm)),

kurt.norm= quantile.for.diags(sample.sim.norm[4,],kurtosis(y.norm)),

max.norm= quantile.for.diags(sample.sim.norm[6,],max(y.norm)),

Za.norm= quantile.for.diags(sample.sim.norm[5,],zhang(y.norm,pnorm)$Za))

#theta<-rnorm(1,0,1)

sample.sim.norm<-sapply(1:rep.inner,function(x){toto<- rpois(meta.params$J,thetatilde); x<-toto;c(mean(x),var(x),max(x))})

toto2<-c(mean=quantile.for.diags(sample.sim.norm[1,],mean(y)),

var= quantile.for.diags(sample.sim.norm[2,],var(y)),

max= quantile.for.diags(sample.sim.norm[3,],max(y)))

ppp<-rbind(ppp,c(toto,toto2,sigma0=meta.params$sigma,N=meta.params$J,nmax= meta.params$nmax))

}

#1 itération=0.5"

results.hsMLp2.Poisson.Nrandom.theta1.sigma2.taurandomV1.Sc6.conjugate.R<-ppp

***save.image("Diags_Poisson3.RData")***

## #Normal – Normal-gamma utilisant conjugate avec rho0 entre 0.1 et 1 (permettant d'éviter les erreurs numériques manifestement)=> nom: tausigmarandom2

### #nspp, Nrandom, taurandom, Scenario1, en local

ppp<-NULL

musigma0<-NULL

rep<-10000

#rep<-3

rep.inner<-5000

theta0ref<-1

for (i in 1:rep){

***if (i/10==floor(i/10)){gc(reset=TRUE)}***

set.seed(i)

meta.params<- ***{toto<-{temp<- rbinom(1,1,0.7); temp*exp(runif(1)*3.45+3)+(1-temp)* (250+750* runif(1))}; if (toto<200){toto<-round(toto,digits=-1)} else {toto<-round(toto,digits=-2)}; sigma<-10^(runif(1)-1);***

list(J=toto, N.y=toto,sigma=(sigma))}

true.params<-{toto<- 1/sqrt(rgamma(1,theta0ref*theta0ref/meta.params$sigma, theta0ref /meta.params$sigma)); list(tau=rnorm(1,0,toto),sigma=toto)}

y<- rnorm(meta.params$J,true.params$tau, true.params$sigma)

**#sigma0<-rlnorm(1,0,1.4)*meta.params$sigma**

**sigma0<- meta.params$sigma**

theta0<- theta0ref

**#thetatilde:**

sigmatilde<-1/sqrt(rgamma(1,theta0ref*theta0ref/meta.params$sigma+length(y)/2, theta0ref /meta.params$sigma+ 1/2*sum((y-mean(y))^2)+ length(y)/2*1*((mean(y)-0)^2)/( length(y)+1^2)))

mutilde<-rnorm(1,(0*1^2+mean(y)*length(y))/(1^2+length(y)), 1/sqrt((1^2+length(y)))*sigmatilde)

y.norm<-qnorm(pnorm(y,mutilde,sigmatilde))

#theta<-rnorm(1,0,1)

sample.sim.norm<-sapply(1:rep.inner,function(x){toto<-rnorm(meta.params$J); toto0<-F;if (sum(!is.finite(toto))==0) {toto0=var(toto)==0} else {toto0=T};while (toto0){toto<- rnorm(meta.params$J); toto0<-F;if (sum(!is.finite(toto))==0) {toto0=var(toto)==0} else {toto0=T}}; x<-toto;c(mean(x),var(x),skewness(x),kurtosis(x),zhang(x,pnorm)$Za)})

toto<-c(mean=quantile.for.diags(sample.sim.norm[1,],mean(y.norm)),

var= quantile.for.diags(sample.sim.norm[2,],var(y.norm)),

skew= quantile.for.diags(sample.sim.norm[3,],skewness(y.norm)),

kurt= quantile.for.diags(sample.sim.norm[4,],kurtosis(y.norm)),

Za= quantile.for.diags(sample.sim.norm[5,],zhang(y.norm,pnorm)$Za))

ppp<-rbind(ppp,c(toto,sigma0=meta.params$sigma,N=meta.params$J))

}

#1 itération=0.5"

results.nspp.Normal.Nrandom.tausigmarandom2.Sc1.conjugate.R<-ppp

***save.image("Diags_Poisson.RData")***

### #spp, Nrandom, taurandom, Scenario1, en local

ppp<- NULL

musigma0<-NULL

rep<-10000

#rep<-3

rep.inner<-5000

theta0ref<-1

for (i in 1:rep){

***if (i/10==floor(i/10)){gc(reset=TRUE)}***

set.seed(i)

meta.params<- ***{toto<-{temp<- rbinom(1,1,0.7); temp*exp(runif(1)*3.45+3)+(1-temp)* (250+750* runif(1))}; if (toto<200){toto<-round(toto,digits=-1)} else {toto<-round(toto,digits=-2)}; sigma<-10^(runif(1)-1);***

list(J=toto, N.y=toto,sigma=(sigma))}

true.params<-{toto<- 1/sqrt(rgamma(1,theta0ref*theta0ref/meta.params$sigma, theta0ref /meta.params$sigma)); list(tau=rnorm(1,0,toto),sigma=toto)}

y<- rnorm(meta.params$J,true.params$tau, true.params$sigma)

**#sigma0<-rlnorm(1,0,1.4)*meta.params$sigma**

**sigma0<- meta.params$sigma**

theta0<- theta0ref

**#thetatilde:**

sigmatilde<-1/sqrt(rgamma(1,theta0ref*theta0ref/meta.params$sigma+length(y)/2, theta0ref /meta.params$sigma+ 1/2*sum((y-mean(y))^2)+ length(y)/2*1*((mean(y)-0)^2)/( length(y)+1^2)))

mutilde<-rnorm(1,(0*1^2+mean(y)*length(y))/(1^2+length(y)), 1/sqrt((1^2+length(y)))*sigmatilde)

y.norm<-y

#theta<-rnorm(1,0,1)

sample.sim.norm<-sapply(1:rep.inner,function(x){toto<-rnorm(meta.params$J,mutilde,sigmatilde); x<-toto;c(mean(x),var(x),skewness(x),kurtosis(x),zhang(x,pnorm)$Za)})

toto<-c(mean=quantile.for.diags(sample.sim.norm[1,],mean(y.norm)),

var= quantile.for.diags(sample.sim.norm[2,],var(y.norm)),

skew= quantile.for.diags(sample.sim.norm[3,],skewness(y.norm)),

kurt= quantile.for.diags(sample.sim.norm[4,],kurtosis(y.norm)),

Za= quantile.for.diags(sample.sim.norm[5,],zhang(y.norm,pnorm)$Za))

ppp<-rbind(ppp,c(toto,musigma0=sigma0/meta.params$sigma,N=meta.params$J))

}

#1 itération=0.5"

results.spp.Normal.Nrandom.tausigmarandom2.Sc1.conjugate.R<-ppp

***save.image("Diags_Poisson.RData")***

### #dspp, Nrandom, taurandom, Scenario1

ppp<- NULL

musigma0<-NULL

rep<-10000

#rep<-3

rep.inner<-5000

theta0ref<-1

for (i in 1:rep){

***if (i/10==floor(i/10)){gc(reset=TRUE)}***

set.seed(i)

meta.params<- ***{toto<-{temp<- rbinom(1,1,0.7); temp*exp(runif(1)*3.45+3)+(1-temp)* (250+750* runif(1))}; if (toto<200){toto<-round(toto,digits=-1)} else {toto<-round(toto,digits=-2)}; sigma<-10^(runif(1)-1);***

list(J=toto, N.y=toto,sigma=(sigma))}

true.params<-{toto<- 1/sqrt(rgamma(1,theta0ref*theta0ref/meta.params$sigma, theta0ref /meta.params$sigma)); list(tau=rnorm(1,0,toto),sigma=toto)}

y<- rnorm(meta.params$J,true.params$tau, true.params$sigma)

**#sigma0<-rlnorm(1,0,1.4)*meta.params$sigma**

**sigma0<- meta.params$sigma**

theta0<- theta0ref

**#thetatilde:**

sigmatilde<-1/sqrt(rgamma(1,theta0ref*theta0ref/meta.params$sigma+length(y)/2, theta0ref /meta.params$sigma+ 1/2*sum((y-mean(y))^2)+ length(y)/2*1*((mean(y)-0)^2)/( length(y)+1^2)))

mutilde<-rnorm(1,(0*1^2+mean(y)*length(y))/(1^2+length(y)), 1/sqrt((1^2+length(y)))*sigmatilde)

y.norm<-y

#theta<-rnorm(1,0,1)

sample.sim.norm<-sapply(1:rep.inner,function(x){toto<-rnorm(meta.params$J,mutilde,sigmatilde); x<-toto;c(mean(x)-mutilde,var(x)-sigmatilde^2,mean(x<=0))})

toto<-c(meanc=quantile.for.diags(sample.sim.norm[1,],mean(y.norm)-mutilde),

varc= quantile.for.diags(sample.sim.norm[2,],var(y.norm)-sigmatilde^2),

plt0= quantile.for.diags(sample.sim.norm[3,],mean(y.norm<=0)))

ppp<-rbind(ppp,c(toto,musigma0=sigma0/meta.params$sigma,N=meta.params$J))

}

#1 itération=0.5"

results.dspp.Normal.Nrandom.tausigmarandom2.Sc1.conjugate.R<-ppp

***save.image("Diags_Poisson.RData")***

### #dspp2, Nrandom, taurandom, Scenario1

ppp<- NULL

musigma0<-NULL

rep<-10000

#rep<-20

rep.inner<-5000

theta0ref<-1

for (i in 1:rep){

***if (i/10==floor(i/10)){gc(reset=TRUE)}***

set.seed(i)

meta.params<- ***{toto<-{temp<- rbinom(1,1,0.7); temp*exp(runif(1)*3.45+3)+(1-temp)* (250+750* runif(1))}; if (toto<200){toto<-round(toto,digits=-1)} else {toto<-round(toto,digits=-2)}; sigma<-10^(runif(1)-1);***

list(J=toto, N.y=toto,sigma=(sigma))}

true.params<-{toto<- 1/sqrt(rgamma(1,theta0ref*theta0ref/meta.params$sigma, theta0ref /meta.params$sigma)); list(tau=rnorm(1,0,toto),sigma=toto)}

y<- rnorm(meta.params$J,true.params$tau, true.params$sigma)

**#sigma0<-rlnorm(1,0,1.4)*meta.params$sigma**

**sigma0<- meta.params$sigma**

theta0<- theta0ref

**#thetatilde:**

sigmatilde<-1/sqrt(rgamma(1,theta0ref*theta0ref/meta.params$sigma+length(y)/2, theta0ref /meta.params$sigma+ 1/2*sum((y-mean(y))^2)+ length(y)/2*1*((mean(y)-0)^2)/( length(y)+1^2)))

mutilde<-rnorm(1,(0*1^2+mean(y)*length(y))/(1^2+length(y)), 1/sqrt((1^2+length(y)))*sigmatilde)

y.norm<-y

#theta<-rnorm(1,0,1)

sample.sim.norm<-sapply(1:rep.inner,function(x){toto<-rnorm(meta.params$J,mutilde,sigmatilde); x<-toto;c(sum(dnorm(x,mutilde,sigmatilde,log=T)))})

toto<-c(LL=quantile.for.diags(sample.sim.norm, sum(dnorm(y,mutilde,sigmatilde,log=T))))

ppp<-rbind(ppp,c(toto,musigma0=sigma0/meta.params$sigma,N=meta.params$J))

}

#1 itération=0.5"

results.dspp2.Normal.Nrandom.tausigmarandom2.Sc1.conjugate.R<-ppp

***save.image("Diags_Poisson_CL3.RData")***

### #dspp3, Nrandombig, taurandom, Scenario1

ppp<- NULL

musigma0<-NULL

rep<-20000

#rep<-3

rep.inner<-5000

theta0ref<-1

for (i in 1:rep){

***if (i/10==floor(i/10)){gc(reset=TRUE)}***

set.seed(i)

meta.params<- ***{toto<-{temp<- 0; temp*exp(runif(1)*3.45+3)+(1-temp)* (250+750* runif(1))}; if (toto<200){toto<-round(toto,digits=-1)} else {toto<-round(toto,digits=-2)}; sigma<-10^(runif(1)-1);***

list(J=toto, N.y=toto,sigma=(sigma))}

true.params<-{toto<- 1/sqrt(rgamma(1,theta0ref*theta0ref/meta.params$sigma, theta0ref /meta.params$sigma)); list(tau=rnorm(1,0,toto),sigma=toto)}

y<- rnorm(meta.params$J,true.params$tau, true.params$sigma)

**#sigma0<-rlnorm(1,0,1.4)*meta.params$sigma**

**sigma0<- meta.params$sigma**

theta0<- theta0ref

**#thetatilde:**

sigmatilde<-1/sqrt(rgamma(1,theta0ref*theta0ref/meta.params$sigma+length(y)/2, theta0ref /meta.params$sigma+ 1/2*sum((y-mean(y))^2)+ length(y)/2*1*((mean(y)-0)^2)/( length(y)+1^2)))

mutilde<-rnorm(1,(0*1^2+mean(y)*length(y))/(1^2+length(y)), 1/sqrt((1^2+length(y)))*sigmatilde)

y.norm<-y

#theta<-rnorm(1,0,1)

sample.sim.norm<-sapply(1:rep.inner,function(x){toto<-rnorm(meta.params$J,mutilde,sigmatilde); x<-toto;c(mean(x)-mutilde,mean(x))})

toto<-c(meanc=quantile.for.diags(sample.sim.norm[1,],mean(y.norm)-mutilde),

mean= quantile.for.diags(sample.sim.norm[2,],mean(y.norm)))

ppp<-rbind(ppp,c(toto,musigma0=sigma0/meta.params$sigma,N=meta.params$J))

}

#1 itération=0.5"

results.dspp3.Normal.Nrandombig.tausigmarandom2.Sc1.conjugate.R<-ppp

***save.image("Diags_Poisson.RData")***

### #maxspp, Nrandom, taurandom, Scenario1,

ppp<-NULL

musigma0<-NULL

rep<-10000

#rep<-3

rep.inner<-5000

theta0ref<-1

for (i in 1:rep){

***if (i/10==floor(i/10)){gc(reset=TRUE)}***

set.seed(i)

meta.params<- ***{toto<-{temp<- rbinom(1,1,0.7); exp(-runif(1)*2+4) }; if (toto<200){toto<-round(toto,digits=-1)} else {toto<-round(toto,digits=-2)}; sigma<-10^(runif(1)-1);***

list(J=toto, N.y=toto,sigma=(sigma))}

true.params<-{toto<- 1/sqrt(rgamma(1,theta0ref*theta0ref/meta.params$sigma, theta0ref /meta.params$sigma)); list(tau=rnorm(1,0,toto),sigma=toto)}

y<- rnorm(meta.params$J,true.params$tau, true.params$sigma)

**#sigma0<-rlnorm(1,0,1.4)*meta.params$sigma**

**sigma0<- meta.params$sigma**

theta0<- theta0ref

**#thetatilde:**

sigmatilde<-1/sqrt(rgamma(1,theta0ref*theta0ref/meta.params$sigma+length(y)/2, theta0ref /meta.params$sigma+ 1/2*sum((y-mean(y))^2)+ length(y)/2*1*((mean(y)-0)^2)/( length(y)+1^2)))

mutilde<-rnorm(1,(0*1^2+mean(y)*length(y))/(1^2+length(y)), 1/sqrt((1^2+length(y)))*sigmatilde)

y.norm<-y

#theta<-rnorm(1,0,1)

sample.sim.norm<-sapply(1:rep.inner,function(x){toto<-rnorm(meta.params$J,mutilde,sigmatilde); x<-toto;c(max(x))})

toto<-c(max=quantile.for.diags(sample.sim.norm,max(y.norm)))

ppp<-rbind(ppp,c(toto,sigma0=sigma0,N=meta.params$J))

}

#1 itération=0.5"

results.maxspp.Normal.Nrandom.tausigmarandom2.Sc1.conjugate.R<-ppp

save.image("Diags_Poisson2.RData")

### #nspp, Nrandom, taurandom, Scenario2, complément C, sur la station

ppp<-NULL

musigma0<-NULL

rep<-100000

#rep<-50000

rep.inner<-5000

theta0ref<-1

for (i in 1:rep){

set.seed(i)

meta.params<- ***{toto<-{temp<- rbinom(1,1,0.7); temp*exp(runif(1)*3.45+3)+(1-temp)* (250+750* runif(1))}; if (toto<200){toto<-round(toto,digits=-1)} else {toto<-round(toto,digits=-2)}; sigma<-10^(runif(1)-1);***

list(J=toto, N.y=toto,sigma=(sigma))}

true.params<-{toto<- 1/sqrt(rgamma(1,theta0ref*theta0ref/meta.params$sigma, theta0ref /meta.params$sigma)); list(tau=rnorm(1,0,toto),sigma=toto)}

y<- rnorm(meta.params$J,true.params$tau, true.params$sigma)

**sigma0<-rlnorm(1,0,1.4)*meta.params$sigma**

**#sigma0<- meta.params$sigma**

#theta0<- rnorm(1,0,3)

theta0<-0

**#thetatilde:**

sigmatilde<-1/sqrt(rgamma(1,theta0ref*theta0ref/sigma0+length(y)/2, theta0ref /sigma0+ 1/2*sum((y-mean(y))^2)+ length(y)/2*1*((mean(y)-theta0)^2)/( length(y)+1^2)))

mutilde<-rnorm(1,(theta0*1^2+mean(y)*length(y))/(1^2+length(y)), 1/sqrt((1^2+length(y)))*sigmatilde)

y.norm<-qnorm(pnorm(y,mutilde,sigmatilde))

#theta<-rnorm(1,0,1)

sample.sim.norm<-sapply(1:rep.inner,function(x){toto<-rnorm(meta.params$J); toto0<-F;if (sum(!is.finite(toto))==0) {toto0=var(toto)==0} else {toto0=T};while (toto0){toto<- rnorm(meta.params$J); toto0<-F;if (sum(!is.finite(toto))==0) {toto0=var(toto)==0} else {toto0=T}}; x<-toto;c(mean(x),var(x),skewness(x),kurtosis(x),zhang(x,pnorm)$Za)})

toto<-c(mean=quantile.for.diags(sample.sim.norm[1,],mean(y.norm)),

var= quantile.for.diags(sample.sim.norm[2,],var(y.norm)),

skew= quantile.for.diags(sample.sim.norm[3,],skewness(y.norm)),

kurt= quantile.for.diags(sample.sim.norm[4,],kurtosis(y.norm)),

Za= quantile.for.diags(sample.sim.norm[5,],zhang(y.norm,pnorm)$Za))

ppp<-rbind(ppp,c(toto,musigma0=sigma0/meta.params$sigma,N=meta.params$J))

}

#1 itération=0.5"

results.nspp.Normal.Nrandom.tausigmarandom2.Sc2.conjugate.R<-ppp

***save.image("Diags_Poisson_CL3.RData")***

### #spp, Nrandom, taurandom, Scenario2, en local

ppp<-NULL

musigma0<-NULL

rep<-100000

#rep<-3

rep.inner<-5000

theta0ref<-1

for (i in 1:rep){

***if (i/10==floor(i/10)){gc(reset=TRUE)}***

set.seed(i)

meta.params<- ***{toto<-{temp<- rbinom(1,1,0.7); temp*exp(runif(1)*3.45+3)+(1-temp)* (250+750* runif(1))}; if (toto<200){toto<-round(toto,digits=-1)} else {toto<-round(toto,digits=-2)}; sigma<-10^(runif(1)-1);***

list(J=toto, N.y=toto,sigma=(sigma))}

true.params<-{toto<- 1/sqrt(rgamma(1,theta0ref*theta0ref/meta.params$sigma, theta0ref /meta.params$sigma)); list(tau=rnorm(1,0,toto),sigma=toto)}

y<- rnorm(meta.params$J,true.params$tau, true.params$sigma)

**sigma0<-rlnorm(1,0,1.4)*meta.params$sigma**

**#sigma0<- meta.params$sigma**

theta0<- theta0ref

**#thetatilde:**

sigmatilde<-1/sqrt(rgamma(1,theta0ref*theta0ref/sigma0+length(y)/2, theta0ref /sigma0+ 1/2*sum((y-mean(y))^2)+ length(y)/2*1*((mean(y)-0)^2)/( length(y)+1^2)))

mutilde<-rnorm(1,(0*1^2+mean(y)*length(y))/(1^2+length(y)), 1/sqrt((1^2+length(y)))*sigmatilde)

y.norm<-y

#theta<-rnorm(1,0,1)

sample.sim.norm<-sapply(1:rep.inner,function(x){toto<-rnorm(meta.params$J,mutilde,sigmatilde); x<-toto;c(mean(x),var(x),skewness(x),kurtosis(x),zhang(x,pnorm)$Za)})

toto<-c(mean=quantile.for.diags(sample.sim.norm[1,],mean(y.norm)),

var= quantile.for.diags(sample.sim.norm[2,],var(y.norm)),

skew= quantile.for.diags(sample.sim.norm[3,],skewness(y.norm)),

kurt= quantile.for.diags(sample.sim.norm[4,],kurtosis(y.norm)),

Za= quantile.for.diags(sample.sim.norm[5,],zhang(y.norm,pnorm)$Za))

ppp<-rbind(ppp,c(toto,musigma0=sigma0/meta.params$sigma,N=meta.params$J))

}

#1 itération=0.5"

results.spp.Normal.Nrandom.tausigmarandom2.Sc2.conjugate.R<-ppp

save.image("Diags_Poisson2.RData")

### #dspp, Nrandom, taurandom, Scenario2

ppp<-NULL

musigma0<-NULL

rep<-100000

#rep<-3

rep.inner<-5000

theta0ref<-1

for (i in 1:rep){

***if (i/10==floor(i/10)){gc(reset=TRUE)}***

set.seed(i)

meta.params<- ***{toto<-{temp<- rbinom(1,1,0.7); temp*exp(runif(1)*3.45+3)+(1-temp)* (250+750* runif(1))}; if (toto<200){toto<-round(toto,digits=-1)} else {toto<-round(toto,digits=-2)}; sigma<-10^(runif(1)-1);***

list(J=toto, N.y=toto,sigma=(sigma))}

true.params<-{toto<- 1/sqrt(rgamma(1,theta0ref*theta0ref/meta.params$sigma, theta0ref /meta.params$sigma)); list(tau=rnorm(1,0,toto),sigma=toto)}

y<- rnorm(meta.params$J,true.params$tau, true.params$sigma)

**sigma0<-rlnorm(1,0,1.4)*meta.params$sigma**

**#sigma0<- meta.params$sigma**

theta0<- theta0ref

**#thetatilde:**

sigmatilde<-1/sqrt(rgamma(1,theta0ref*theta0ref/sigma0+length(y)/2, theta0ref /sigma0+ 1/2*sum((y-mean(y))^2)+ length(y)/2*1*((mean(y)-0)^2)/( length(y)+1^2)))

mutilde<-rnorm(1,(0*1^2+mean(y)*length(y))/(1^2+length(y)), 1/sqrt((1^2+length(y)))*sigmatilde)

y.norm<-y

#theta<-rnorm(1,0,1)

sample.sim.norm<-sapply(1:rep.inner,function(x){toto<-rnorm(meta.params$J,mutilde,sigmatilde); x<-toto;c(mean(x)-mutilde,var(x)-sigmatilde^2,mean(x<=0))})

toto<-c(meanc=quantile.for.diags(sample.sim.norm[1,],mean(y.norm)-mutilde),

varc= quantile.for.diags(sample.sim.norm[2,],var(y.norm)-sigmatilde^2),

plt0= quantile.for.diags(sample.sim.norm[3,],mean(y.norm<=0)))

ppp<-rbind(ppp,c(toto,musigma0=sigma0/meta.params$sigma,N=meta.params$J))

}

#1 itération=0.5"

results.dspp.Normal.Nrandom.tausigmarandom2.Sc2.conjugate.R<-ppp

save.image("Diags_Poisson2.RData")

### #dspp2, Nrandom, taurandom, Scenario2

ppp<-NULL

musigma0<-NULL

rep<-100000

#rep<-3

rep.inner<-5000

theta0ref<-1

for (i in 1:rep){

***if (i/10==floor(i/10)){gc(reset=TRUE)}***

set.seed(i)

meta.params<- ***{toto<-{temp<- rbinom(1,1,0.7); temp*exp(runif(1)*3.45+3)+(1-temp)* (250+750* runif(1))}; if (toto<200){toto<-round(toto,digits=-1)} else {toto<-round(toto,digits=-2)}; sigma<-10^(runif(1)-1);***

list(J=toto, N.y=toto,sigma=(sigma))}

true.params<-{toto<- 1/sqrt(rgamma(1,theta0ref*theta0ref/meta.params$sigma, theta0ref /meta.params$sigma)); list(tau=rnorm(1,0,toto),sigma=toto)}

y<- rnorm(meta.params$J,true.params$tau, true.params$sigma)

**sigma0<-rlnorm(1,0,1.4)*meta.params$sigma**

**#sigma0<- meta.params$sigma**

theta0<- theta0ref

**#thetatilde:**

sigmatilde<-1/sqrt(rgamma(1,theta0ref*theta0ref/sigma0+length(y)/2, theta0ref /sigma0+ 1/2*sum((y-mean(y))^2)+ length(y)/2*1*((mean(y)-0)^2)/( length(y)+1^2)))

mutilde<-rnorm(1,(0*1^2+mean(y)*length(y))/(1^2+length(y)), 1/sqrt((1^2+length(y)))*sigmatilde)

y.norm<-y

#theta<-rnorm(1,0,1)

sample.sim.norm<-sapply(1:rep.inner,function(x){toto<-rnorm(meta.params$J,mutilde,sigmatilde); x<-toto;c(sum(dnorm(x,mutilde,sigmatilde,log=T)))})

toto<-c(LL=quantile.for.diags(sample.sim.norm, sum(dnorm(y,mutilde,sigmatilde,log=T))))

ppp<-rbind(ppp,c(toto,musigma0=sigma0/meta.params$sigma,N=meta.params$J))

}

#1 itération=0.5"

results.dspp2.Normal.Nrandom.tausigmarandom2.Sc2.conjugate.R<-ppp

save.image("Diags_Poisson_CL3.RData")

### #nspp, Nrandom, taurandom, Scenario3, complément C, sur la station

ppp<-NULL

musigma0<-NULL

rep=100000

#rep<-50000

rep.inner<-5000

theta0ref<-1

for (i in 1:rep){

set.seed(i)

meta.params<- ***{toto<-{temp<- rbinom(1,1,0.7); temp*exp(runif(1)*3.45+3)+(1-temp)* (250+750* runif(1))}; if (toto<200){toto<-round(toto,digits=-1)} else {toto<-round(toto,digits=-2)}; sigma<-10^(runif(1)-1);***

list(J=toto, N.y=toto,sigma=(sigma))}

true.params<-{toto<- 1/sqrt(rgamma(1,theta0ref*theta0ref/meta.params$sigma, theta0ref /meta.params$sigma)); list(tau=rnorm(1,0,toto),sigma=toto)}

y<- rnorm(meta.params$J,true.params$tau, true.params$sigma)

**sigma0<-rlnorm(1,0,1.4)*meta.params$sigma**

**#sigma0<- meta.params$sigma**

theta0<- rnorm(1,0,3)

#theta0<-0

**#thetatilde:**

sigmatilde<-1/sqrt(rgamma(1,theta0ref*theta0ref/sigma0+length(y)/2, theta0ref /sigma0+ 1/2*sum((y-mean(y))^2)+ length(y)/2*1*((mean(y)-theta0)^2)/( length(y)+1^2)))

mutilde<-rnorm(1,(theta0*1^2+mean(y)*length(y))/(1^2+length(y)), 1/sqrt((1^2+length(y)))*sigmatilde)

y.norm<-qnorm(pnorm(y,mutilde,sigmatilde))

#theta<-rnorm(1,0,1)

sample.sim.norm<-sapply(1:rep.inner,function(x){toto<-rnorm(meta.params$J); toto0<-F;if (sum(!is.finite(toto))==0) {toto0=var(toto)==0} else {toto0=T};while (toto0){toto<- rnorm(meta.params$J); toto0<-F;if (sum(!is.finite(toto))==0) {toto0=var(toto)==0} else {toto0=T}}; x<-toto;c(mean(x),var(x),skewness(x),kurtosis(x),zhang(x,pnorm)$Za)})

toto<-c(mean=quantile.for.diags(sample.sim.norm[1,],mean(y.norm)),

var= quantile.for.diags(sample.sim.norm[2,],var(y.norm)),

skew= quantile.for.diags(sample.sim.norm[3,],skewness(y.norm)),

kurt= quantile.for.diags(sample.sim.norm[4,],kurtosis(y.norm)),

Za= quantile.for.diags(sample.sim.norm[5,],zhang(y.norm,pnorm)$Za))

ppp<-rbind(ppp,c(toto,musigma0=sigma0/meta.params$sigma,theta0=theta0,theta00=theta0,N=meta.params$J, sigma0= meta.params$sigma))

}

#1 itération=0.5"

results.nspp.Normal.Nrandom.tausigmarandom2.Sc3.conjugate.R<-ppp

***save.image("Diags_Poisson3.RData")***

### #spp, Nrandom, taurandom, Scenario3, en local

ppp<-NULL

musigma0<-NULL

rep<-100000

#rep<-3

rep.inner<-5000

theta0ref<-1

for (i in 1:rep){

***if (i/10==floor(i/10)){gc(reset=TRUE)}***

set.seed(i)

meta.params<- ***{toto<-{temp<- rbinom(1,1,0.7); temp*exp(runif(1)*3.45+3)+(1-temp)* (250+750* runif(1))}; if (toto<200){toto<-round(toto,digits=-1)} else {toto<-round(toto,digits=-2)}; sigma<-10^(runif(1)-1);***

list(J=toto, N.y=toto,sigma=(sigma))}

true.params<-{toto<- 1/sqrt(rgamma(1,theta0ref*theta0ref/meta.params$sigma, theta0ref /meta.params$sigma)); list(tau=rnorm(1,0,toto),sigma=toto)}

y<- rnorm(meta.params$J,true.params$tau, true.params$sigma)

**sigma0<-rlnorm(1,0,1.4)*meta.params$sigma**

**#sigma0<- meta.params$sigma**

theta0<- rnorm(1,0,3)

#theta0<-0

**#thetatilde:**

sigmatilde<-1/sqrt(rgamma(1,theta0ref*theta0ref/sigma0+length(y)/2, theta0ref /sigma0+ 1/2*sum((y-mean(y))^2)+ length(y)/2*1*((mean(y)-theta0)^2)/( length(y)+1^2)))

mutilde<-rnorm(1,(theta0*1^2+mean(y)*length(y))/(1^2+length(y)), 1/sqrt((1^2+length(y)))*sigmatilde)

y.norm<-y

#theta<-rnorm(1,0,1)

sample.sim.norm<-sapply(1:rep.inner,function(x){toto<-rnorm(meta.params$J,mutilde,sigmatilde); x<-toto;c(mean(x),var(x),skewness(x),kurtosis(x),zhang(x,pnorm)$Za)})

toto<-c(mean=quantile.for.diags(sample.sim.norm[1,],mean(y.norm)),

var= quantile.for.diags(sample.sim.norm[2,],var(y.norm)),

skew= quantile.for.diags(sample.sim.norm[3,],skewness(y.norm)),

kurt= quantile.for.diags(sample.sim.norm[4,],kurtosis(y.norm)),

Za= quantile.for.diags(sample.sim.norm[5,],zhang(y.norm,pnorm)$Za))

ppp<-rbind(ppp,c(toto,musigma0=sigma0/meta.params$sigma, theta0=theta0,theta00=theta0,N=meta.params$J, sigma0=meta.params$sigma))

}

#1 itération=0.5"

results.spp.Normal.Nrandom.tausigmarandom2.Sc3.conjugate.R<-ppp

save.image("Diags_Poisson2.RData")

### #dspp, Nrandom, taurandom, Scenario3

ppp<-NULL

musigma0<-NULL

rep<-100000

#rep<-3

rep.inner<-5000

theta0ref<-1

for (i in 1:rep){

***if (i/10==floor(i/10)){gc(reset=TRUE)}***

set.seed(i)

meta.params<- ***{toto<-{temp<- rbinom(1,1,0.7); temp*exp(runif(1)*3.45+3)+(1-temp)* (250+750* runif(1))}; if (toto<200){toto<-round(toto,digits=-1)} else {toto<-round(toto,digits=-2)}; sigma<-10^(runif(1)-1);***

list(J=toto, N.y=toto,sigma=(sigma))}

true.params<-{toto<- 1/sqrt(rgamma(1,theta0ref*theta0ref/meta.params$sigma, theta0ref /meta.params$sigma)); list(tau=rnorm(1,0,toto),sigma=toto)}

y<- rnorm(meta.params$J,true.params$tau, true.params$sigma)

**sigma0<-rlnorm(1,0,1.4)*meta.params$sigma**

**#sigma0<- meta.params$sigma**

theta0<- rnorm(1,0,3)

#theta0<-0

**#thetatilde:**

sigmatilde<-1/sqrt(rgamma(1,theta0ref*theta0ref/sigma0+length(y)/2, theta0ref /sigma0+ 1/2*sum((y-mean(y))^2)+ length(y)/2*1*((mean(y)-theta0)^2)/( length(y)+1^2)))

mutilde<-rnorm(1,(theta0*1^2+mean(y)*length(y))/(1^2+length(y)), 1/sqrt((1^2+length(y)))*sigmatilde)

y.norm<-y

#theta<-rnorm(1,0,1)

sample.sim.norm<-sapply(1:rep.inner,function(x){toto<-rnorm(meta.params$J,mutilde,sigmatilde); x<-toto;c(mean(x)-mutilde,var(x)-sigmatilde^2,mean(x<=0))})

toto<-c(meanc=quantile.for.diags(sample.sim.norm[1,],mean(y.norm)-mutilde),

varc= quantile.for.diags(sample.sim.norm[2,],var(y.norm)-sigmatilde^2),

plt0= quantile.for.diags(sample.sim.norm[3,],mean(y.norm<=0)))

ppp<-rbind(ppp,c(toto,musigma0=sigma0/meta.params$sigma, theta0=theta0,theta00=theta0,N=meta.params$J, sigma0=meta.params$sigma))

}

#1 itération=0.5"

results.dspp.Normal.Nrandom.tausigmarandom2.Sc3.conjugate.R<-ppp

save.image("Diags_Poisson2.RData")

### #dspp2, Nrandom, taurandom, Scenario3

ppp<-NULL

musigma0<-NULL

rep<-100000

#rep<-3

rep.inner<-5000

theta0ref<-1

for (i in 1:rep){

***if (i/10==floor(i/10)){gc(reset=TRUE)}***

set.seed(i)

meta.params<- ***{toto<-{temp<- rbinom(1,1,0.7); temp*exp(runif(1)*3.45+3)+(1-temp)* (250+750* runif(1))}; if (toto<200){toto<-round(toto,digits=-1)} else {toto<-round(toto,digits=-2)}; sigma<-10^(runif(1)-1);***

list(J=toto, N.y=toto,sigma=(sigma))}

true.params<-{toto<- 1/sqrt(rgamma(1,theta0ref*theta0ref/meta.params$sigma, theta0ref /meta.params$sigma)); list(tau=rnorm(1,0,toto),sigma=toto)}

y<- rnorm(meta.params$J,true.params$tau, true.params$sigma)

**sigma0<-rlnorm(1,0,1.4)*meta.params$sigma**

**#sigma0<- meta.params$sigma**

theta0<- rnorm(1,0,3)

#theta0<-0

**#thetatilde:**

sigmatilde<-1/sqrt(rgamma(1,theta0ref*theta0ref/sigma0+length(y)/2, theta0ref /sigma0+ 1/2*sum((y-mean(y))^2)+ length(y)/2*1*((mean(y)-theta0)^2)/( length(y)+1^2)))

mutilde<-rnorm(1,(theta0*1^2+mean(y)*length(y))/(1^2+length(y)), 1/sqrt((1^2+length(y)))*sigmatilde)

y.norm<-y

#theta<-rnorm(1,0,1)

sample.sim.norm<-sapply(1:rep.inner,function(x){toto<-rnorm(meta.params$J,mutilde,sigmatilde); x<-toto;c(sum(dnorm(x,mutilde,sigmatilde,log=T)))})

toto<-c(LL=quantile.for.diags(sample.sim.norm, sum(dnorm(y,mutilde,sigmatilde,log=T))))

ppp<-rbind(ppp,c(toto,musigma0=sigma0/meta.params$sigma, theta0=theta0,theta00=theta0,N=meta.params$J, sigma0=meta.params$sigma))

}

#1 itération=0.5"

results.dspp2.Normal.Nrandom.tausigmarandom2.Sc3.conjugate.R<-ppp

save.image("Diags_Poisson_CL3.RData")

### #nspp, Nrandom, taurandom, Scenario4, complément C, en local

ppp<-NULL

musigma0<-NULL

rep<-100000

#rep<-3

rep.inner<-5000

theta0ref<-1

for (i in 1:rep){

***if (i/10==floor(i/10)){gc(reset=TRUE)}***

set.seed(i)

meta.params<- ***{toto<-{temp<- rbinom(1,1,0.7); temp*exp(runif(1)*3.45+3)+(1-temp)* (250+750* runif(1))}; if (toto<200){toto<-round(toto,digits=-1)} else {toto<-round(toto,digits=-2)}; sigma<-10^(runif(1)-1);***

list(J=toto, N.y=toto,sigma=(sigma))}

true.params<-{toto<- 1/sqrt(rgamma(1,theta0ref*theta0ref/meta.params$sigma, theta0ref /meta.params$sigma)); list(tau=rnorm(1,0,toto),sigma=toto)}

y<- rnorm(meta.params$J,0, 1)

**#sigma0<-rlnorm(1,0,1.4)*meta.params$sigma**

**sigma0<- meta.params$sigma**

theta0<- theta0ref

**#thetatilde:**

sigmatilde<-1/sqrt(rgamma(1,theta0ref*theta0ref/meta.params$sigma+length(y)/2, theta0ref /meta.params$sigma+ 1/2*sum((y-mean(y))^2)+ length(y)/2*1*((mean(y)-0)^2)/( length(y)+1^2)))

mutilde<-rnorm(1,(0*1^2+mean(y)*length(y))/(1^2+length(y)), 1/sqrt((1^2+length(y)))*sigmatilde)

y.norm<-qnorm(pnorm(y,mutilde,sigmatilde))

#theta<-rnorm(1,0,1)

sample.sim.norm<-sapply(1:rep.inner,function(x){toto<-rnorm(meta.params$J); toto0<-F;if (sum(!is.finite(toto))==0) {toto0=var(toto)==0} else {toto0=T};while (toto0){toto<- rnorm(meta.params$J); toto0<-F;if (sum(!is.finite(toto))==0) {toto0=var(toto)==0} else {toto0=T}}; x<-toto;c(mean(x),var(x),skewness(x),kurtosis(x),zhang(x,pnorm)$Za)})

toto<-c(mean=quantile.for.diags(sample.sim.norm[1,],mean(y.norm)),

var= quantile.for.diags(sample.sim.norm[2,],var(y.norm)),

skew= quantile.for.diags(sample.sim.norm[3,],skewness(y.norm)),

kurt= quantile.for.diags(sample.sim.norm[4,],kurtosis(y.norm)),

Za= quantile.for.diags(sample.sim.norm[5,],zhang(y.norm,pnorm)$Za))

ppp<-rbind(ppp,c(toto,sigma0=sigma0,N=meta.params$J))

}

#1 itération=0.5"

results.nspp.Normal.Nrandom.tausigmarandom2.Sc4.conjugate.R<-ppp

***save.image("Diags_Poisson4.RData")***

***# et éventuellement 2***

### #spp, Nrandom, taurandom, Scenario4,

ppp<-NULL

musigma0<-NULL

rep<-100000

#rep<-3

rep.inner<-5000

theta0ref<-1

for (i in 1:rep){

***if (i/10==floor(i/10)){gc(reset=TRUE)}***

set.seed(i)

meta.params<- ***{toto<-{temp<- rbinom(1,1,0.7); temp*exp(runif(1)*3.45+3)+(1-temp)* (250+750* runif(1))}; if (toto<200){toto<-round(toto,digits=-1)} else {toto<-round(toto,digits=-2)}; sigma<-10^(runif(1)-1);***

list(J=toto, N.y=toto,sigma=(sigma))}

true.params<-{toto<- 1/sqrt(rgamma(1,theta0ref*theta0ref/meta.params$sigma, theta0ref /meta.params$sigma)); list(tau=rnorm(1,0,toto),sigma=toto)}

y<- rnorm(meta.params$J,0,1)

**#sigma0<-rlnorm(1,0,1.4)*meta.params$sigma**

**sigma0<- meta.params$sigma**

theta0<- theta0ref

**#thetatilde:**

sigmatilde<-1/sqrt(rgamma(1,theta0ref*theta0ref/meta.params$sigma+length(y)/2, theta0ref /meta.params$sigma+ 1/2*sum((y-mean(y))^2)+ length(y)/2*1*((mean(y)-0)^2)/( length(y)+1^2)))

mutilde<-rnorm(1,(0*1^2+mean(y)*length(y))/(1^2+length(y)), 1/sqrt((1^2+length(y)))*sigmatilde)

y.norm<-y

#theta<-rnorm(1,0,1)

sample.sim.norm<-sapply(1:rep.inner,function(x){toto<-rnorm(meta.params$J,mutilde,sigmatilde); x<-toto;c(mean(x),var(x),skewness(x),kurtosis(x),zhang(x,pnorm)$Za)})

toto<-c(mean=quantile.for.diags(sample.sim.norm[1,],mean(y.norm)),

var= quantile.for.diags(sample.sim.norm[2,],var(y.norm)),

skew= quantile.for.diags(sample.sim.norm[3,],skewness(y.norm)),

kurt= quantile.for.diags(sample.sim.norm[4,],kurtosis(y.norm)),

Za= quantile.for.diags(sample.sim.norm[5,],zhang(y.norm,pnorm)$Za))

ppp<-rbind(ppp,c(toto,sigma0=sigma0,N=meta.params$J))

}

#1 itération=0.5"

results.spp.Normal.Nrandom.tausigmarandom2.Sc4.conjugate.R<-ppp

save.image("Diags_Poisson2.RData")

### #dspp, Nrandom, taurandom, Scenario4

ppp<-NULL

musigma0<-NULL

rep<-100000

#rep<-3

rep.inner<-5000

theta0ref<-1

for (i in 1:rep){

***if (i/10==floor(i/10)){gc(reset=TRUE)}***

set.seed(i)

meta.params<- ***{toto<-{temp<- rbinom(1,1,0.7); temp*exp(runif(1)*3.45+3)+(1-temp)* (250+750* runif(1))}; if (toto<200){toto<-round(toto,digits=-1)} else {toto<-round(toto,digits=-2)}; sigma<-10^(runif(1)-1);***

list(J=toto, N.y=toto,sigma=(sigma))}

true.params<-{toto<- 1/sqrt(rgamma(1,theta0ref*theta0ref/meta.params$sigma, theta0ref /meta.params$sigma)); list(tau=rnorm(1,0,toto),sigma=toto)}

y<- rnorm(meta.params$J,0,1)

**#sigma0<-rlnorm(1,0,1.4)*meta.params$sigma**

**sigma0<- meta.params$sigma**

theta0<- theta0ref

**#thetatilde:**

sigmatilde<-1/sqrt(rgamma(1,theta0ref*theta0ref/meta.params$sigma+length(y)/2, theta0ref /meta.params$sigma+ 1/2*sum((y-mean(y))^2)+ length(y)/2*1*((mean(y)-0)^2)/( length(y)+1^2)))

mutilde<-rnorm(1,(0*1^2+mean(y)*length(y))/(1^2+length(y)), 1/sqrt((1^2+length(y)))*sigmatilde)

y.norm<-y

#theta<-rnorm(1,0,1)

sample.sim.norm<-sapply(1:rep.inner,function(x){toto<-rnorm(meta.params$J,mutilde,sigmatilde); x<-toto;c(mean(x)-mutilde,var(x)-sigmatilde^2,mean(x<=0))})

toto<-c(meanc=quantile.for.diags(sample.sim.norm[1,],mean(y.norm)-mutilde),

varc= quantile.for.diags(sample.sim.norm[2,],var(y.norm)-sigmatilde^2),

plt0= quantile.for.diags(sample.sim.norm[3,],mean(y.norm<=0)))

ppp<-rbind(ppp,c(toto,sigma0=sigma0,N=meta.params$J))

}

#1 itération=0.5"

results.dspp.Normal.Nrandom.tausigmarandom2.Sc4.conjugate.R<-ppp

save.image("Diags_Poisson4.RData")

#et éventuellement 2

### #dspp2, Nrandom, taurandom, Scenario4

ppp<-NULL

musigma0<-NULL

rep<-100000

#rep<-3

rep.inner<-5000

theta0ref<-1

for (i in 1:rep){

***if (i/10==floor(i/10)){gc(reset=TRUE)}***

set.seed(i)

meta.params<- ***{toto<-{temp<- rbinom(1,1,0.7); temp*exp(runif(1)*3.45+3)+(1-temp)* (250+750* runif(1))}; if (toto<200){toto<-round(toto,digits=-1)} else {toto<-round(toto,digits=-2)}; sigma<-10^(runif(1)-1);***

list(J=toto, N.y=toto,sigma=(sigma))}

true.params<-{toto<- 1/sqrt(rgamma(1,theta0ref*theta0ref/meta.params$sigma, theta0ref /meta.params$sigma)); list(tau=rnorm(1,0,toto),sigma=toto)}

y<- rnorm(meta.params$J,0,1)

**#sigma0<-rlnorm(1,0,1.4)*meta.params$sigma**

**sigma0<- meta.params$sigma**

theta0<- theta0ref

**#thetatilde:**

sigmatilde<-1/sqrt(rgamma(1,theta0ref*theta0ref/meta.params$sigma+length(y)/2, theta0ref /meta.params$sigma+ 1/2*sum((y-mean(y))^2)+ length(y)/2*1*((mean(y)-0)^2)/( length(y)+1^2)))

mutilde<-rnorm(1,(0*1^2+mean(y)*length(y))/(1^2+length(y)), 1/sqrt((1^2+length(y)))*sigmatilde)

y.norm<-y

#theta<-rnorm(1,0,1)

sample.sim.norm<-sapply(1:rep.inner,function(x){toto<-rnorm(meta.params$J,mutilde,sigmatilde); x<-toto;c(sum(dnorm(x,mutilde,sigmatilde,log=T)))})

toto<-c(LL=quantile.for.diags(sample.sim.norm, sum(dnorm(y,mutilde,sigmatilde,log=T))))

ppp<-rbind(ppp,c(toto,sigma0=sigma0,N=meta.params$J))

}

#1 itération=0.5"

results.dspp2.Normal.Nrandom.tausigmarandom2.Sc4.conjugate.R<-ppp

save.image("Diags_Poisson_CL3.RData")

### ##predictive prior, Nrandom, taurandom, Scenario2, en local

ppp<-NULL

musigma0<-NULL

rep<-3000

#rep<-10

rep.inner<-5000

for (i in 1:rep){

***if (i/10==floor(i/10)){gc(reset=TRUE)}***

set.seed(i)

meta.params<- ***{toto<-{temp<- rbinom(1,1,0.7); temp*exp(runif(1)*3.45+3)+(1-temp)* (250+750* runif(1))}; if (toto<200){toto<-round(toto,digits=-1)} else {toto<-round(toto,digits=-2)}; sigma<-10^(runif(1)-1);***

list(J=toto, N.y=toto,sigma=sigma)}

true.params<-{toto<- 1/sqrt(rgamma(1,theta0ref*theta0ref/meta.params$sigma, theta0ref /meta.params$sigma)); list(tau=rnorm(1,0,toto),sigma=toto)}

y<- rnorm(meta.params$J,true.params$tau, true.params$sigma)

**sigma0<-rlnorm(1,0,1.4)*meta.params$sigma**

#theta<-rnorm(1,0,1)

sample.sim<-sapply(1:rep.inner,function(x){temp<- 1/sqrt(rgamma(1,theta0ref*theta0ref/sigma0, theta0ref /sigma0));toto<-rnorm(meta.params$J, rnorm(1,0,temp), temp); toto0<-F;if (sum(!is.finite(toto))==0) {toto0=var(toto)==0} else {toto0=T};while (toto0){ temp<- 1/sqrt(rgamma(1,theta0ref*theta0ref/sigma0, theta0ref /sigma0));toto<-rnorm(meta.params$J, rnorm(1,0,temp), temp);toto0<-F;if (sum(!is.finite(toto))==0) {toto0=var(toto)==0} else {toto0=T}};toto})

toto<-c(mean=quantile.for.diags(apply(sample.sim,2,function(x){c(mean(x),var(x),skewness(x),kurtosis(x))})[1,],mean(y)),

var=quantile.for.diags(apply(sample.sim,2,function(x){c(mean(x),var(x),skewness(x),kurtosis(x))})[2,],var(y)),

skew=quantile.for.diags(apply(sample.sim,2,function(x){c(mean(x),var(x),skewness(x),kurtosis(x))})[3,],skewness(y)),

kurt=quantile.for.diags(apply(sample.sim,2,function(x){c(mean(x),var(x),skewness(x),kurtosis(x))})[4,],kurtosis(y)))

ppp<-rbind(ppp,c(toto,musigma0=sigma0/meta.params$sigma,N=meta.params$J))

}

results.prior.pred.Normal.Nrandom.tausigmarandom2.Sc2.conjugate.R<-ppp

***save.image("Diags_Poisson.RData")***

## #Bernouilli :

### #nspp, Nrandom, thetarandom5, Scenario1, en local

ppp<- NULL

musigma0<-NULL

rep=10000

#rep<-10

rep.inner<-5000

theta0ref<-1

for (i in 1:rep){

***if (i/10==floor(i/10)){gc(reset=TRUE)}***

set.seed(i)

meta.params<- ***{toto<-{temp<- rbinom(1,1,0.7); temp*exp(runif(1)*3.45+3)+(1-temp)* (250+750* runif(1))}; if (toto<200){toto<-round(toto,digits=-1)} else {toto<-round(toto,digits=-2)}; p=10^runif(1);*** list(J=toto, N.y=toto, p=p)}

true.params=list(theta={toto<-rbeta(1, meta.params$p, meta.params$p);toto})

y<- rbinom(meta.params$J,1,true.params$theta)

**#sigma0<-rlnorm(1,0,1.4)*meta.params$sigma**

**sigma0<- 1**

theta0<- theta0ref

**#thetatilde:**

thetatilde<-rbeta(1, meta.params$p*sigma0+sum(y) , meta.params$p*sigma0+length(y)-sum(y))

y.norm<-qnorm(pbinom(y-0.1,1,thetatilde)+runif(length(y))*( pbinom(y,1,thetatilde)- pbinom(y-0.1,1,thetatilde)))

#theta<-rnorm(1,0,1)

sample.sim.norm<-sapply(1:rep.inner,function(x){toto<-rnorm(meta.params$J); toto0<-F;if (sum(!is.finite(toto))==0) {toto0=var(toto)==0} else {toto0=T};while (toto0){toto<- rnorm(meta.params$J); toto0<-F;if (sum(!is.finite(toto))==0) {toto0=var(toto)==0} else {toto0=T}}; x<-toto;c(mean(x),var(x),skewness(x),kurtosis(x),zhang(x,pnorm)$Za)})

toto<-c(mean=quantile.for.diags(sample.sim.norm[1,],mean(y.norm)),

var= quantile.for.diags(sample.sim.norm[2,],var(y.norm)),

skew= quantile.for.diags(sample.sim.norm[3,],skewness(y.norm)),

kurt= quantile.for.diags(sample.sim.norm[4,],kurtosis(y.norm)),

Za= quantile.for.diags(sample.sim.norm[5,],zhang(y.norm,pnorm)$Za))

ppp<-rbind(ppp,c(toto,p=meta.params$p,musigma0=sigma0,N=meta.params$J))

}

#1 itération=0.5"

results.nspp.Bernoulli.Nrandom.thetarandom5.Sc1.conjugate.R<-ppp

***save.image("Diags_Poisson.RData")***

### #spp, Nrandom, thetarandom5, Scenario1, en local

ppp<- NULL

musigma0<-NULL

rep=10000

#rep<-30

rep.inner<-5000

theta0ref<-1

for (i in 1:rep){

***if (i/10==floor(i/10)){gc(reset=TRUE)}***

set.seed(i)

meta.params<- ***{toto<-{temp<- rbinom(1,1,0.7); temp*exp(runif(1)*3.45+3)+(1-temp)* (250+750* runif(1))}; if (toto<200){toto<-round(toto,digits=-1)} else {toto<-round(toto,digits=-2)}; p=10^runif(1);*** list(J=toto, N.y=toto, p=p)}

true.params=list(theta={toto<-rbeta(1, meta.params$p, meta.params$p);toto})

y<- rbinom(meta.params$J,1,true.params$theta)

**#sigma0<-rlnorm(1,0,1.4)*meta.params$sigma**

**sigma0<- 1**

theta0<- theta0ref

**#thetatilde:**

thetatilde<-rbeta(1, meta.params$p*sigma0+sum(y) , meta.params$p*sigma0+length(y)-sum(y))

y.norm<-y

#theta<-rnorm(1,0,1)

sample.sim.norm<-sapply(1:rep.inner,function(x){toto<- rbinom(meta.params$J,1,thetatilde); x<-toto;c(mean(x),var(x))})

toto<-c(mean=quantile.for.diags(sample.sim.norm[1,],mean(y.norm)),

var= quantile.for.diags(sample.sim.norm[2,],var(y.norm)))

ppp<-rbind(ppp,c(toto,p=meta.params$p,musigma0=sigma0,N=meta.params$J))

}

#1 itération=0.5"

results.spp.Bernoulli.Nrandom.thetarandom5.Sc1.conjugate.R<-ppp

***save.image("Diags_Poisson.RData")***

### #dspp, Nrandom, thetarandom5, Scenario1, en local

ppp<- NULL

musigma0<-NULL

rep=10000

#rep<-30

rep.inner<-5000

theta0ref<-1

for (i in 1:rep){

***if (i/10==floor(i/10)){gc(reset=TRUE)}***

set.seed(i)

meta.params<- ***{toto<-{temp<- rbinom(1,1,0.7); temp*exp(runif(1)*3.45+3)+(1-temp)* (250+750* runif(1))}; if (toto<200){toto<-round(toto,digits=-1)} else {toto<-round(toto,digits=-2)}; p=10^runif(1);*** list(J=toto, N.y=toto, p=p)}

true.params=list(theta={toto<-rbeta(1, meta.params$p, meta.params$p);toto})

y<- rbinom(meta.params$J,1,true.params$theta)

**#sigma0<-rlnorm(1,0,1.4)*meta.params$sigma**

**sigma0<- 1**

theta0<- theta0ref

**#thetatilde:**

thetatilde<-rbeta(1, meta.params$p*sigma0+sum(y) , meta.params$p*sigma0+length(y)-sum(y))

y.norm<-y

#theta<-rnorm(1,0,1)

sample.sim.norm<-sapply(1:rep.inner,function(x){toto<- rbinom(meta.params$J,1,thetatilde); x<-toto;c(mean(x)-thetatilde,var(x)-thetatilde*(1-thetatilde),mean(x==0),mean(x[1]==0))})

toto<-c(meanc=quantile.for.diags(sample.sim.norm[1,],mean(y.norm)-thetatilde),

varc= quantile.for.diags(sample.sim.norm[2,],var(y.norm)-thetatilde*(1-thetatilde)),p0= quantile.for.diags(sample.sim.norm[3,],mean(y.norm==0)), p01= quantile.for.diags(sample.sim.norm[4,],mean(y.norm[1]==0)))

ppp<-rbind(ppp,c(toto,p=meta.params$p,musigma0=sigma0,N=meta.params$J))

}

#1 itération=0.5"

results.dspp.Bernoulli.Nrandom.thetarandom5.Sc1.conjugate.R<-ppp

save.image("Diags_Poisson4.RData")

### #dspp2, Nrandom, thetarandom5, Scenario1, en local

ppp<- NULL

musigma0<-NULL

rep=10000

#rep<-30

rep.inner<-5000

theta0ref<-1

for (i in 1:rep){

***if (i/10==floor(i/10)){gc(reset=TRUE)}***

set.seed(i)

meta.params<- ***{toto<-{temp<- rbinom(1,1,0.7); temp*exp(runif(1)*3.45+3)+(1-temp)* (250+750* runif(1))}; if (toto<200){toto<-round(toto,digits=-1)} else {toto<-round(toto,digits=-2)}; p=10^runif(1);*** list(J=toto, N.y=toto, p=p)}

true.params=list(theta={toto<-rbeta(1, meta.params$p, meta.params$p);toto})

y<- rbinom(meta.params$J,1,true.params$theta)

**#sigma0<-rlnorm(1,0,1.4)*meta.params$sigma**

**sigma0<- 1**

theta0<- theta0ref

**#thetatilde:**

thetatilde<-rbeta(1, meta.params$p*sigma0+sum(y) , meta.params$p*sigma0+length(y)-sum(y))

y.norm<-y

#theta<-rnorm(1,0,1)

sample.sim.norm<-sapply(1:rep.inner,function(x){toto<- rbinom(meta.params$J,1,thetatilde); x<-toto;c(sum(dbinom(x,1,thetatilde,log=T)))})

toto<-c(LL=quantile.for.diags(sample.sim.norm, sum(dbinom(y,1,thetatilde,log=T))))

ppp<-rbind(ppp,c(toto,p=meta.params$p,musigma0=sigma0,N=meta.params$J))

}

#1 itération=0.5"

results.dspp2.Bernoulli.Nrandom.thetarandom5.Sc1.conjugate.R<-ppp

save.image("Diags_Poisson_CL3.RData")

### #nspp, Nrandom, thetarandom5, Scenario2, sur la station de calcul

#scenario 2: since var(theta)=mean(theta)*1/(4p+2) with mean(theta=1/2)

**#pour rester avec prior au minimum uniform, on va prendre la forme suivante: non pas p*sigma0 mais 1+(p-1)*sigma0**

ppp<-NULL

musigma0<-NULL

rep=100000

#rep=50000

#rep<-10

rep.inner<-5000

theta0ref<-1

for (i in 1:rep){

***if (i/10==floor(i/10)){gc(reset=TRUE)}***

set.seed(i)

meta.params<- ***{toto<-{temp<- rbinom(1,1,0.7); temp*exp(runif(1)*3.45+3)+(1-temp)* (250+750* runif(1))}; if (toto<200){toto<-round(toto,digits=-1)} else {toto<-round(toto,digits=-2)}; p=10^runif(1);*** list(J=toto, N.y=toto, p=p)}

true.params=list(theta={toto<-rbeta(1, meta.params$p, meta.params$p);toto})

y<- rbinom(meta.params$J,1,true.params$theta)

**sigma0<-rlnorm(1,0,1.4)**

**#sigma0<- meta.params$sigma**

theta0<- theta0ref

**#thetatilde:**

thetatilde<-rbeta(1, 1+(meta.params$p-1)*sigma0+sum(y) , 1+(meta.params$p-1)*sigma0+length(y)-sum(y))

y.norm<-qnorm(pbinom(y-0.1,1,thetatilde)+runif(length(y))*( pbinom(y,1,thetatilde)- pbinom(y-0.1,1,thetatilde)))

#theta<-rnorm(1,0,1)

sample.sim.norm<-sapply(1:rep.inner,function(x){toto<-rnorm(meta.params$J); toto0<-F;if (sum(!is.finite(toto))==0) {toto0=var(toto)==0} else {toto0=T};while (toto0){toto<- rnorm(meta.params$J); toto0<-F;if (sum(!is.finite(toto))==0) {toto0=var(toto)==0} else {toto0=T}}; x<-toto;c(mean(x),var(x),skewness(x),kurtosis(x),zhang(x,pnorm)$Za)})

toto<-c(mean=quantile.for.diags(sample.sim.norm[1,],mean(y.norm)),

var= quantile.for.diags(sample.sim.norm[2,],var(y.norm)),

skew= quantile.for.diags(sample.sim.norm[3,],skewness(y.norm)),

kurt= quantile.for.diags(sample.sim.norm[4,],kurtosis(y.norm)),

Za= quantile.for.diags(sample.sim.norm[5,],zhang(y.norm,pnorm)$Za))

ppp<-rbind(ppp,c(toto, p=meta.params$p,musigma0=sigma0,N=meta.params$J))

}

#1 itération=0.5"

results.nspp.Bernoulli.Nrandom.thetarandom5.Sc2.conjugate.R<-ppp

save.image("Diags_Poisson3.RData")

### #spp, Nrandom, thetarandom5, Scenario2, en local

ppp<-NULL

musigma0<-NULL

rep=100000

#rep<-30

rep.inner<-5000

theta0ref<-1

for (i in 1:rep){

***if (i/10==floor(i/10)){gc(reset=TRUE)}***

set.seed(i)

meta.params<- ***{toto<-{temp<- rbinom(1,1,0.7); temp*exp(runif(1)*3.45+3)+(1-temp)* (250+750* runif(1))}; if (toto<200){toto<-round(toto,digits=-1)} else {toto<-round(toto,digits=-2)}; p=10^runif(1);*** list(J=toto, N.y=toto, p=p)}

true.params=list(theta={toto<-rbeta(1, meta.params$p, meta.params$p);toto})

y<- rbinom(meta.params$J,1,true.params$theta)

**sigma0<-rlnorm(1,0,1.4)**

**#sigma0<- meta.params$sigma**

theta0<- theta0ref

**#thetatilde:**

thetatilde<-rbeta(1, 1+(meta.params$p-1)*sigma0+sum(y) , 1+(meta.params$p-1)*sigma0+length(y)-sum(y))

y.norm<-y

#theta<-rnorm(1,0,1)

sample.sim.norm<-sapply(1:rep.inner,function(x){toto<- rbinom(meta.params$J,1,thetatilde); x<-toto;c(mean(x),var(x))})

toto<-c(mean=quantile.for.diags(sample.sim.norm[1,],mean(y.norm)),

var= quantile.for.diags(sample.sim.norm[2,],var(y.norm)))

ppp<-rbind(ppp,c(toto,p=meta.params$p,musigma0=sigma0,N=meta.params$J))

}

#1 itération=0.5"

results.spp.Bernoulli.Nrandom.thetarandom5.Sc2.conjugate.R<-ppp

save.image("Diags_Poisson1.RData")

### #dspp, Nrandom, thetarandom5, Scenario2

ppp<-NULL

musigma0<-NULL

rep=100000

#rep<-30

rep.inner<-5000

theta0ref<-1

for (i in 1:rep){

***if (i/10==floor(i/10)){gc(reset=TRUE)}***

set.seed(i)

meta.params<- ***{toto<-{temp<- rbinom(1,1,0.7); temp*exp(runif(1)*3.45+3)+(1-temp)* (250+750* runif(1))}; if (toto<200){toto<-round(toto,digits=-1)} else {toto<-round(toto,digits=-2)}; p=10^runif(1);*** list(J=toto, N.y=toto, p=p)}

true.params=list(theta={toto<-rbeta(1, meta.params$p, meta.params$p);toto})

y<- rbinom(meta.params$J,1,true.params$theta)

**sigma0<-rlnorm(1,0,1.4)**

**#sigma0<- meta.params$sigma**

theta0<- theta0ref

**#thetatilde:**

thetatilde<-rbeta(1, 1+(meta.params$p-1)*sigma0+sum(y) , 1+(meta.params$p-1)*sigma0+length(y)-sum(y))

y.norm<-y

#theta<-rnorm(1,0,1)

sample.sim.norm<-sapply(1:rep.inner,function(x){toto<- rbinom(meta.params$J,1,thetatilde); x<-toto;c(mean(x)-thetatilde,var(x)-thetatilde*(1-thetatilde),mean(x==0),mean(x[1]==0))})

toto<-c(meanc=quantile.for.diags(sample.sim.norm[1,],mean(y.norm)-thetatilde),

varc= quantile.for.diags(sample.sim.norm[2,],var(y.norm)-thetatilde*(1-thetatilde)),p0= quantile.for.diags(sample.sim.norm[3,],mean(y.norm==0)), p01= quantile.for.diags(sample.sim.norm[4,],mean(y.norm[1]==0)))

ppp<-rbind(ppp,c(toto,p=meta.params$p,musigma0=sigma0,N=meta.params$J))

}

#1 itération=0.5"

results.dspp.Bernoulli.Nrandom.thetarandom5.Sc2.conjugate.R<-ppp

save.image("Diags_Poisson3.RData")

### #dspp2, Nrandom, thetarandom5, Scenario2

ppp<-NULL

musigma0<-NULL

rep=100000

#rep<-30

rep.inner<-5000

theta0ref<-1

for (i in 1:rep){

***if (i/10==floor(i/10)){gc(reset=TRUE)}***

set.seed(i)

meta.params<- ***{toto<-{temp<- rbinom(1,1,0.7); temp*exp(runif(1)*3.45+3)+(1-temp)* (250+750* runif(1))}; if (toto<200){toto<-round(toto,digits=-1)} else {toto<-round(toto,digits=-2)}; p=10^runif(1);*** list(J=toto, N.y=toto, p=p)}

true.params=list(theta={toto<-rbeta(1, meta.params$p, meta.params$p);toto})

y<- rbinom(meta.params$J,1,true.params$theta)

**sigma0<-rlnorm(1,0,1.4)**

**#sigma0<- meta.params$sigma**

theta0<- theta0ref

**#thetatilde:**

thetatilde<-rbeta(1, 1+(meta.params$p-1)*sigma0+sum(y) , 1+(meta.params$p-1)*sigma0+length(y)-sum(y))

y.norm<-y

#theta<-rnorm(1,0,1)

sample.sim.norm<-sapply(1:rep.inner,function(x){toto<- rbinom(meta.params$J,1,thetatilde); x<-toto;c(sum(dbinom(x,1,thetatilde,log=T)))})

toto<-c(LL=quantile.for.diags(sample.sim.norm, sum(dbinom(y,1,thetatilde,log=T))))

ppp<-rbind(ppp,c(toto,p=meta.params$p,musigma0=sigma0,N=meta.params$J))

}

#1 itération=0.5"

results.dspp2.Bernoulli.Nrandom.thetarandom5.Sc2.conjugate.R<-ppp

save.image("Diags_Poisson_CL3.RData")

### #nspp, Nrandom, thetarandom5, Scenario3, sur la station de calcul

ppp<-NULL

musigma0<-NULL

#rep=100000

rep<-100000

#rep<-1000

rep.inner2<-1000

#rep<-10

rep.inner<-5000

theta0ref<-1

dim.ref<-0

while (dim.ref<rep){

for (i in 1:rep.inner2){

***if (i/10==floor(i/10)){gc(reset=TRUE)}***

set.seed(i+dim.ref)

meta.params<- ***{toto<-{temp<- rbinom(1,1,0.7); temp*exp(runif(1)*3.45+3)+(1-temp)* (250+750* runif(1))}; if (toto<200){toto<-round(toto,digits=-1)} else {toto<-round(toto,digits=-2)}; p=10^runif(1);*** list(J=toto, N.y=toto, p=p)}

true.params=list(theta={toto<-rbeta(1, meta.params$p, meta.params$p);toto})

y<- rbinom(meta.params$J,1,true.params$theta)

**sigma0<-rlnorm(1,0,1.4)**

**#sigma0bis<-rlnorm(1,0,1.4)**

**#sigma0<- meta.params$sigma**

theta0<- runif(1,0.25,0.75)

#formula chosen so that mean of beta=theta0 and variance the same as in scenario 2

**#thetatilde:**

thetatilde<-rbeta(1, theta0*(2*(1+(meta.params$p-1)*sigma0)+1)-theta0+sum(y) , (1-theta0)*(2*(1+(meta.params$p-1)*sigma0)+1)-(1-theta0)+length(y)-sum(y))

y.norm<-qnorm(pbinom(y-0.1,1,thetatilde)+runif(length(y))*( pbinom(y,1,thetatilde)- pbinom(y-0.1,1,thetatilde)))

#theta<-rnorm(1,0,1)

sample.sim.norm<-sapply(1:rep.inner,function(x){toto<-rnorm(meta.params$J); toto0<-F;if (sum(!is.finite(toto))==0) {toto0=var(toto)==0} else {toto0=T};while (toto0){toto<- rnorm(meta.params$J); toto0<-F;if (sum(!is.finite(toto))==0) {toto0=var(toto)==0} else {toto0=T}}; x<-toto;c(mean(x),var(x),skewness(x),kurtosis(x),zhang(x,pnorm)$Za)})

toto<-c(mean=quantile.for.diags(sample.sim.norm[1,],mean(y.norm)),

var= quantile.for.diags(sample.sim.norm[2,],var(y.norm)),

skew= quantile.for.diags(sample.sim.norm[3,],skewness(y.norm)),

kurt= quantile.for.diags(sample.sim.norm[4,],kurtosis(y.norm)),

Za= quantile.for.diags(sample.sim.norm[5,],zhang(y.norm,pnorm)$Za))

ppp<-rbind(ppp,c(toto, p=meta.params$p,musigma0=sigma0,theta0=theta0,N=meta.params$J))

}

#1 itération=0.5"

results.nspp.Bernoulli.Nrandom.thetarandom5.Sc3.conjugate.R<-ppp

save.image("Diags_Poisson4.RData")

dim.ref<-dim(ppp)[1]

***}***

save.image("Diags_Poisson4.RData")

### #spp, Nrandom, thetarandom5, Scenario3, en local

ppp<-NULL

musigma0<-NULL

rep=100000

#rep<-30

rep.inner<-5000

theta0ref<-1

for (i in 1:rep){

***if (i/10==floor(i/10)){gc(reset=TRUE)}***

set.seed(i)

meta.params<- ***{toto<-{temp<- rbinom(1,1,0.7); temp*exp(runif(1)*3.45+3)+(1-temp)* (250+750* runif(1))}; if (toto<200){toto<-round(toto,digits=-1)} else {toto<-round(toto,digits=-2)}; p=10^runif(1);*** list(J=toto, N.y=toto, p=p)}

true.params=list(theta={toto<-rbeta(1, meta.params$p, meta.params$p);toto})

y<- rbinom(meta.params$J,1,true.params$theta)

**sigma0<-rlnorm(1,0,1.4)**

**#sigma0bis<-rlnorm(1,0,1.4)**

**#sigma0<- meta.params$sigma**

theta0<- runif(1,0.25,0.75)

#formula chosen so that mean of beta=theta0 and variance the same as in scenario 2

**#thetatilde:**

thetatilde<-rbeta(1, theta0*(2*(1+(meta.params$p-1)*sigma0)+1)-theta0+sum(y) , (1-theta0)*(2*(1+(meta.params$p-1)*sigma0)+1)-(1-theta0)+length(y)-sum(y))

y.norm<-y

#theta<-rnorm(1,0,1)

sample.sim.norm<-sapply(1:rep.inner,function(x){toto<- rbinom(meta.params$J,1,thetatilde); x<-toto;c(mean(x),var(x))})

toto<-c(mean=quantile.for.diags(sample.sim.norm[1,],mean(y.norm)),

var= quantile.for.diags(sample.sim.norm[2,],var(y.norm)))

ppp<-rbind(ppp,c(toto,p=meta.params$p,musigma0=sigma0,theta0=theta0,N=meta.params$J))

}

#1 itération=0.5"

results.spp.Bernoulli.Nrandom.thetarandom5.Sc3.conjugate.R<-ppp

save.image("Diags_Poisson2.RData")

### #dspp, Nrandom, thetarandom5, Scenario3, en local

ppp<-NULL

musigma0<-NULL

rep=100000

#rep<-30

rep.inner<-5000

theta0ref<-1

for (i in 1:rep){

***if (i/10==floor(i/10)){gc(reset=TRUE)}***

set.seed(i)

meta.params<- ***{toto<-{temp<- rbinom(1,1,0.7); temp*exp(runif(1)*3.45+3)+(1-temp)* (250+750* runif(1))}; if (toto<200){toto<-round(toto,digits=-1)} else {toto<-round(toto,digits=-2)}; p=10^runif(1);*** list(J=toto, N.y=toto, p=p)}

true.params=list(theta={toto<-rbeta(1, meta.params$p, meta.params$p);toto})

y<- rbinom(meta.params$J,1,true.params$theta)

**sigma0<-rlnorm(1,0,1.4)**

**#sigma0bis<-rlnorm(1,0,1.4)**

**#sigma0<- meta.params$sigma**

theta0<- runif(1,0.25,0.75)

#formula chosen so that mean of beta=theta0 and variance the same as in scenario 2

**#thetatilde:**

thetatilde<-rbeta(1, theta0*(2*(1+(meta.params$p-1)*sigma0)+1)-theta0+sum(y) , (1-theta0)*(2*(1+(meta.params$p-1)*sigma0)+1)-(1-theta0)+length(y)-sum(y))

y.norm<-y

#theta<-rnorm(1,0,1)

sample.sim.norm<-sapply(1:rep.inner,function(x){toto<- rbinom(meta.params$J,1,thetatilde); x<-toto; ;c(mean(x)-thetatilde,var(x)-thetatilde*(1-thetatilde),mean(x==0),mean(x[1]==0))})

toto<-c(meanc=quantile.for.diags(sample.sim.norm[1,],mean(y.norm)-thetatilde),

varc= quantile.for.diags(sample.sim.norm[2,],var(y.norm)-thetatilde*(1-thetatilde)),p0= quantile.for.diags(sample.sim.norm[3,],mean(y.norm==0)), p01= quantile.for.diags(sample.sim.norm[4,],mean(y.norm[1]==0)))

ppp<-rbind(ppp,c(toto,p=meta.params$p,musigma0=sigma0,theta0=theta0,N=meta.params$J))

}

#1 itération=0.5"

results.dspp.Bernoulli.Nrandom.thetarandom5.Sc3.conjugate.R<-ppp

save.image("Diags_Poisson3.RData")

### #dspp2, Nrandom, thetarandom5, Scenario3, en local

ppp<-NULL

musigma0<-NULL

rep=100000

#rep<-30

rep.inner<-5000

theta0ref<-1

for (i in 1:rep){

***if (i/10==floor(i/10)){gc(reset=TRUE)}***

set.seed(i)

meta.params<- ***{toto<-{temp<- rbinom(1,1,0.7); temp*exp(runif(1)*3.45+3)+(1-temp)* (250+750* runif(1))}; if (toto<200){toto<-round(toto,digits=-1)} else {toto<-round(toto,digits=-2)}; p=10^runif(1);*** list(J=toto, N.y=toto, p=p)}

true.params=list(theta={toto<-rbeta(1, meta.params$p, meta.params$p);toto})

y<- rbinom(meta.params$J,1,true.params$theta)

**sigma0<-rlnorm(1,0,1.4)**

**#sigma0bis<-rlnorm(1,0,1.4)**

**#sigma0<- meta.params$sigma**

theta0<- runif(1,0.25,0.75)

#formula chosen so that mean of beta=theta0 and variance the same as in scenario 2

**#thetatilde:**

thetatilde<-rbeta(1, theta0*(2*(1+(meta.params$p-1)*sigma0)+1)-theta0+sum(y) , (1-theta0)*(2*(1+(meta.params$p-1)*sigma0)+1)-(1-theta0)+length(y)-sum(y))

y.norm<-y

#theta<-rnorm(1,0,1)

sample.sim.norm<-sapply(1:rep.inner,function(x){toto<- rbinom(meta.params$J,1,thetatilde); x<-toto; c(sum(dbinom(x,1,thetatilde,log=T)))})

toto<-c(LL=quantile.for.diags(sample.sim.norm, sum(dbinom(y,1,thetatilde,log=T))))

ppp<-rbind(ppp,c(toto,p=meta.params$p,musigma0=sigma0,theta0=theta0,N=meta.params$J))

}

#1 itération=0.5"

results.dspp2.Bernoulli.Nrandom.thetarandom5.Sc3.conjugate.R<-ppp

save.image("Diags_Poisson_CL3.RData")

### #nspp, Nrandom, thetarandom5, Scenario4, en local

ppp<-NULL

musigma0<-NULL

rep=100000

#rep<-10

rep.inner<-5000

theta0ref<-1

for (i in 1:rep){

***if (i/10==floor(i/10)){gc(reset=TRUE)}***

set.seed(i)

meta.params<- ***{toto<-{temp<- rbinom(1,1,0.7); temp*exp(runif(1)*3.45+3)+(1-temp)* (250+750* runif(1))}; if (toto<200){toto<-round(toto,digits=-1)} else {toto<-round(toto,digits=-2)}; p=10^runif(1);*** list(J=toto, N.y=toto, p=p)}

true.params=list(theta={toto<-rbeta(1, meta.params$p, meta.params$p);toto})

y<- rbinom(meta.params$J,1,0.5)

**#sigma0<-rlnorm(1,0,1.4)*meta.params$sigma**

**sigma0<- 1**

theta0<- theta0ref

**#thetatilde:**

thetatilde<-rbeta(1, (1+(meta.params$p-1)*sigma0)+sum(y) , (1+(meta.params$p-1)*sigma0)+length(y)-sum(y))

y.norm<-qnorm(pbinom(y-0.1,1,thetatilde)+runif(length(y))*( pbinom(y,1,thetatilde)- pbinom(y-0.1,1,thetatilde)))

#theta<-rnorm(1,0,1)

sample.sim.norm<-sapply(1:rep.inner,function(x){toto<-rnorm(meta.params$J); toto0<-F;if (sum(!is.finite(toto))==0) {toto0=var(toto)==0} else {toto0=T};while (toto0){toto<- rnorm(meta.params$J); toto0<-F;if (sum(!is.finite(toto))==0) {toto0=var(toto)==0} else {toto0=T}}; x<-toto;c(mean(x),var(x),skewness(x),kurtosis(x),zhang(x,pnorm)$Za)})

toto<-c(mean=quantile.for.diags(sample.sim.norm[1,],mean(y.norm)),

var= quantile.for.diags(sample.sim.norm[2,],var(y.norm)),

skew= quantile.for.diags(sample.sim.norm[3,],skewness(y.norm)),

kurt= quantile.for.diags(sample.sim.norm[4,],kurtosis(y.norm)),

Za= quantile.for.diags(sample.sim.norm[5,],zhang(y.norm,pnorm)$Za))

ppp<-rbind(ppp,c(toto,p=meta.params$p,sigma0=sigma0* meta.params$p,N=meta.params$J))

}

#1 itération=0.5"

results.nspp.Bernoulli.Nrandom.thetarandom5.Sc4.conjugate.R<-ppp

save.image("Diags_Poisson1.RData")

#in diags3

### #spp, Nrandom, thetarandom5, Scenario4, en local

ppp<-NULL

musigma0<-NULL

rep=100000

#rep<-30

rep.inner<-5000

theta0ref<-1

for (i in 1:rep){

***if (i/10==floor(i/10)){gc(reset=TRUE)}***

set.seed(i)

meta.params<- ***{toto<-{temp<- rbinom(1,1,0.7); temp*exp(runif(1)*3.45+3)+(1-temp)* (250+750* runif(1))}; if (toto<200){toto<-round(toto,digits=-1)} else {toto<-round(toto,digits=-2)}; p=10^runif(1);*** list(J=toto, N.y=toto, p=p)}

true.params=list(theta={toto<-rbeta(1, meta.params$p, meta.params$p);toto})

y<- rbinom(meta.params$J,1,0.5)

**#sigma0<-rlnorm(1,0,1.4)**

**sigma0<- 1**

theta0<- theta0ref

**#thetatilde:**

thetatilde<-rbeta(1, (1+(meta.params$p-1)*sigma0)+sum(y) , (1+(meta.params$p-1)*sigma0)+length(y)-sum(y))

y.norm<-y

#theta<-rnorm(1,0,1)

sample.sim.norm<-sapply(1:rep.inner,function(x){toto<- rbinom(meta.params$J,1,thetatilde); x<-toto;c(mean(x),var(x))})

toto<-c(mean=quantile.for.diags(sample.sim.norm[1,],mean(y.norm)),

var= quantile.for.diags(sample.sim.norm[2,],var(y.norm)))

ppp<-rbind(ppp,c(toto, p=meta.params$p,sigma0=sigma0* meta.params$p,N=meta.params$J))

}

#1 itération=0.5"

results.spp.Bernoulli.Nrandom.thetarandom5.Sc4.conjugate.R<-ppp

save.image("Diags_Poisson2.RData")

### #spp, theta0.7, Nrandom, thetarandom5, Scenario4, en local

ppp<-NULL

musigma0<-NULL

rep=100000

#rep<-30

rep.inner<-5000

theta0ref<-1

for (i in 1:rep){

***if (i/10==floor(i/10)){gc(reset=TRUE)}***

set.seed(i)

meta.params<- ***{toto<-{temp<- rbinom(1,1,0.7); temp*exp(runif(1)*3.45+3)+(1-temp)* (250+750* runif(1))}; if (toto<200){toto<-round(toto,digits=-1)} else {toto<-round(toto,digits=-2)}; p=10^runif(1);*** list(J=toto, N.y=toto, p=p)}

true.params=list(theta={toto<-rbeta(1, meta.params$p, meta.params$p);toto})

y<- rbinom(meta.params$J,1,0.7)

**#sigma0<-rlnorm(1,0,1.4)**

**sigma0<- 1**

theta0<- theta0ref

**#thetatilde:**

thetatilde<-rbeta(1, 0.7/0.3*(1+(meta.params$p-1)*sigma0)+sum(y) , (1+(meta.params$p-1)*sigma0)+length(y)-sum(y))

y.norm<-y

#theta<-rnorm(1,0,1)

sample.sim.norm<-sapply(1:rep.inner,function(x){toto<- rbinom(meta.params$J,1,thetatilde); x<-toto;c(mean(x),var(x))})

toto<-c(mean=quantile.for.diags(sample.sim.norm[1,],mean(y.norm)),

var= quantile.for.diags(sample.sim.norm[2,],var(y.norm)))

ppp<-rbind(ppp,c(toto, p=meta.params$p,sigma0=sigma0* meta.params$p,N=meta.params$J))

}

results.spp.Bernoulli.theta07.Nrandom.thetarandom5.Sc4.conjugate.R<-ppp

save.image("Diags_Poisson2.RData")

### #dspp, Nrandom, thetarandom5, Scenario4

ppp<-NULL

musigma0<-NULL

rep=100000

#rep<-30

rep.inner<-5000

theta0ref<-1

for (i in 1:rep){

***if (i/10==floor(i/10)){gc(reset=TRUE)}***

set.seed(i)

meta.params<- ***{toto<-{temp<- rbinom(1,1,0.7); temp*exp(runif(1)*3.45+3)+(1-temp)* (250+750* runif(1))}; if (toto<200){toto<-round(toto,digits=-1)} else {toto<-round(toto,digits=-2)}; p=10^runif(1);*** list(J=toto, N.y=toto, p=p)}

true.params=list(theta={toto<-rbeta(1, meta.params$p, meta.params$p);toto})

y<- rbinom(meta.params$J,1,0.5)

**#sigma0<-rlnorm(1,0,1.4)**

**sigma0<- 1**

theta0<- theta0ref

**#thetatilde:**

thetatilde<-rbeta(1, (1+(meta.params$p-1)*sigma0)+sum(y) , (1+(meta.params$p-1)*sigma0)+length(y)-sum(y))

y.norm<-y

#theta<-rnorm(1,0,1)

sample.sim.norm<-sapply(1:rep.inner,function(x){toto<- rbinom(meta.params$J,1,thetatilde); x<-toto;c(mean(x)-thetatilde,var(x)-thetatilde*(1-thetatilde),mean(x==0),mean(x[1]==0))})

toto<-c(meanc=quantile.for.diags(sample.sim.norm[1,],mean(y.norm)-thetatilde),

varc= quantile.for.diags(sample.sim.norm[2,],var(y.norm)-thetatilde*(1-thetatilde)),p0= quantile.for.diags(sample.sim.norm[3,],mean(y.norm==0)), p01= quantile.for.diags(sample.sim.norm[4,],mean(y.norm[1]==0)))

ppp<-rbind(ppp,c(toto, p=meta.params$p,sigma0=sigma0* meta.params$p,N=meta.params$J))

}

#1 itération=0.5"

results.dspp.Bernoulli.Nrandom.thetarandom5.Sc4.conjugate.R<-ppp

save.image("Diags_Poisson2.RData")

### #dspp2, Nrandom, thetarandom5, Scenario4

ppp<-NULL

musigma0<-NULL

rep=100000

#rep<-30

rep.inner<-5000

theta0ref<-1

for (i in 1:rep){

***if (i/10==floor(i/10)){gc(reset=TRUE)}***

set.seed(i)

meta.params<- ***{toto<-{temp<- rbinom(1,1,0.7); temp*exp(runif(1)*3.45+3)+(1-temp)* (250+750* runif(1))}; if (toto<200){toto<-round(toto,digits=-1)} else {toto<-round(toto,digits=-2)}; p=10^runif(1);*** list(J=toto, N.y=toto, p=p)}

true.params=list(theta={toto<-rbeta(1, meta.params$p, meta.params$p);toto})

y<- rbinom(meta.params$J,1,0.5)

**#sigma0<-rlnorm(1,0,1.4)**

**sigma0<- 1**

theta0<- theta0ref

**#thetatilde:**

thetatilde<-rbeta(1, (1+(meta.params$p-1)*sigma0)+sum(y) , (1+(meta.params$p-1)*sigma0)+length(y)-sum(y))

y.norm<-y

#theta<-rnorm(1,0,1)

sample.sim.norm<-sapply(1:rep.inner,function(x){toto<- rbinom(meta.params$J,1,thetatilde); x<-toto;c(sum(dbinom(x,1,thetatilde,log=T)))})

toto<-c(LL=quantile.for.diags(sample.sim.norm, sum(dbinom(y,1,thetatilde,log=T))))

ppp<-rbind(ppp,c(toto, p=meta.params$p,sigma0=sigma0* meta.params$p,N=meta.params$J))

}

#1 itération=0.5"

results.dspp2.Bernoulli.Nrandom.thetarandom5.Sc4.conjugate.R<-ppp

save.image("Diags_Poisson_CL3.RData")

# R commands to build the tables and figures

## Tables for Text S1 (Scenario 1)

#### #=>A1: Poisson, nspp, conjugate, Scenario 1

**#R, nspp, conjugate**

No<- results.nspp.Poisson.Nrandom.theta1.sigma2.taurandomV1.Sc1.conjugate.R [,"N"]

x<-summary(results.nspp.Poisson.Nrandom.theta1.sigma2.taurandomV1.Sc1.conjugate.R [,"mean"]~No, fun=function(x){c(

ks.D=( (paste(format(round(ks.test(x,"punif")$s,3), format="f", nsmall=3), symbols.ks(x),sep=""))),

p.0.05=( (paste(format(round({mean(x<0.05/2| x>(1-0.05/2))},3), format="f", nsmall=3), symbols.p05(x),sep=""))),

p.0.01=( (paste(format(round({mean(x<0.01/2| x>(1-0.01/2))},3), format="f", nsmall=3), symbols.p01(x),sep=""))))},method="cross",g=4)

print(x,prn=F)

x<-summary(results.nspp.Poisson.Nrandom.theta1.sigma2.taurandomV1.Sc1.conjugate.R [,"var"]~No, fun=function(x){c(

ks.D=( (paste(format(round(ks.test(x,"punif")$s,3), format="f", nsmall=3), symbols.ks(x),sep=""))),

p.0.05=( (paste(format(round({mean(x<0.05/2| x>(1-0.05/2))},3), format="f", nsmall=3), symbols.p05(x),sep=""))),

p.0.01=( (paste(format(round({mean(x<0.01/2| x>(1-0.01/2))},3), format="f", nsmall=3), symbols.p01(x),sep=""))))},method="cross",g=4)

#write.table("pnsp, t=var",file="test.txt",sep="\t",quote=F,row.names=F,append=T)

print(x,prn=F)

#write.table(as.data.frame(x)[,1:2],file="test.txt",sep="\t",quote=F,row.names=F,append=T)

x<-summary(results.nspp.Poisson.Nrandom.theta1.sigma2.taurandomV1.Sc1.conjugate.R [,"skew"]~No, fun=function(x){c(

ks.D=( (paste(format(round(ks.test(x,"punif")$s,3), format="f", nsmall=3), symbols.ks(x),sep=""))),

p.0.05=( (paste(format(round({mean(x<0.05/2| x>(1-0.05/2))},3), format="f", nsmall=3), symbols.p05(x),sep=""))),

p.0.01=( (paste(format(round({mean(x<0.01/2| x>(1-0.01/2))},3), format="f", nsmall=3), symbols.p01(x),sep=""))))},method="cross",g=4)

print(x,prn=F)

#write.table("pnsp, t=skewness",file="test.txt",sep="\t",quote=F,row.names=F,append=T)

#write.table(as.data.frame(x)[,1:2],file="test.txt",sep="\t",quote=F,row.names=F,append=T)

x<-summary(results.nspp.Poisson.Nrandom.theta1.sigma2.taurandomV1.Sc1.conjugate.R [,"kurt"]~No, fun=function(x){c(

ks.D=( (paste(format(round(ks.test(x,"punif")$s,3), format="f", nsmall=3), symbols.ks(x),sep=""))),

p.0.05=( (paste(format(round({mean(x<0.05/2| x>(1-0.05/2))},3), format="f", nsmall=3), symbols.p05(x),sep=""))),

p.0.01=( (paste(format(round({mean(x<0.01/2| x>(1-0.01/2))},3), format="f", nsmall=3), symbols.p01(x),sep=""))))},method="cross",g=4)

print(x,prn=F)

#write.table("pnsp, t=kurtosis",file="test.txt",sep="\t",quote=F,row.names=F,append=T)

#write.table(as.data.frame(x)[,1:2],file="test.txt",sep="\t",quote=F,row.names=F,append=T)

x<-summary(results.nspp.Poisson.Nrandom.theta1.sigma2.taurandomV1.Sc1.conjugate.R [,"Za"]~No, fun=function(x){c(

ks.D=( (paste(format(round(ks.test(x,"punif")$s,3), format="f", nsmall=3), symbols.ks(x),sep=""))),

p.0.05=( (paste(format(round({mean(x<0.05/2| x>(1-0.05/2))},3), format="f", nsmall=3), symbols.p05(x),sep=""))),

p.0.01=( (paste(format(round({mean(x<0.01/2| x>(1-0.01/2))},3), format="f", nsmall=3), symbols.p01(x),sep=""))))},method="cross",g=4)

print(x,prn=F)

#write.table("pnsp, t=Za",file="test.txt",sep="\t",quote=F,row.names=F,append=T)

#write.table(as.data.frame(x)[,1:2],file="test.txt",sep="\t",quote=F,row.names=F,append=T)

#### #=>A1: Poisson, spp et dspp & dspp2 & dspp3, conjugate, Scenario 1

**#R, spp, conjugate**

No<- results.spp.Poisson.Nrandom.theta1.sigma2.taurandomV1.Sc1.conjugate.R [,"N"]

x<-summary(results.spp.Poisson.Nrandom.theta1.sigma2.taurandomV1.Sc1.conjugate.R [,"mean"]~No, fun=function(x){c(

ks.D=( (paste(format(round(ks.test(x,"punif")$s,3), format="f", nsmall=3), symbols.ks(x),sep=""))),

p.0.05=( (paste(format(round({mean(x<0.05/2| x>(1-0.05/2))},3), format="f", nsmall=3), symbols.p05(x),sep=""))),

p.0.01=( (paste(format(round({mean(x<0.01/2| x>(1-0.01/2))},3), format="f", nsmall=3), symbols.p01(x),sep=""))))},method="cross",g=4)

print(x,prn=F)

#write.table("psp, t=mean",file="test.txt",sep="\t",quote=F,row.names=F,append=T)

#write.table(as.data.frame(x)[,1:2],file="test.txt",sep="\t",quote=F,row.names=F,append=T)

x<-summary(results.spp.Poisson.Nrandom.theta1.sigma2.taurandomV1.Sc1.conjugate.R [,"var"]~No, fun=function(x){c(

ks.D=( (paste(format(round(ks.test(x,"punif")$s,3), format="f", nsmall=3), symbols.ks(x),sep=""))),

p.0.05=( (paste(format(round({mean(x<0.05/2| x>(1-0.05/2))},3), format="f", nsmall=3), symbols.p05(x),sep=""))),

p.0.01=( (paste(format(round({mean(x<0.01/2| x>(1-0.01/2))},3), format="f", nsmall=3), symbols.p01(x),sep=""))))},method="cross",g=4)

print(x,prn=F)

#write.table("pnsp, t=var",file="test.txt",sep="\t",quote=F,row.names=F,append=T)

#write.table(as.data.frame(x)[,1:2],file="test.txt",sep="\t",quote=F,row.names=F,append=T)

**#R, dspp, conjugate**

No<- results.dspp.Poisson.Nrandom.theta1.sigma2.taurandomV1.Sc1.conjugate.R [,"N"]

x<-summary(results.dspp.Poisson.Nrandom.theta1.sigma2.taurandomV1.Sc1.conjugate.R [,"p0"]~No, fun=function(x){c(

ks.D=( (paste(format(round(ks.test(x,"punif")$s,3), format="f", nsmall=3), symbols.ks(x),sep=""))),

p.0.05=( (paste(format(round({mean(x<0.05/2| x>(1-0.05/2))},3), format="f", nsmall=3), symbols.p05(x),sep=""))),

p.0.01=( (paste(format(round({mean(x<0.01/2| x>(1-0.01/2))},3), format="f", nsmall=3), symbols.p01(x),sep=""))))},method="cross",g=4)

print(x,prn=F)

#write.table("psp, t=p0",file="test.txt",sep="\t",quote=F,row.names=F,append=T)

#write.table(as.data.frame(x)[,1:2],file="test.txt",sep="\t",quote=F,row.names=F,append=T)

#########"just for ref.

summary(results.dspp.Poisson.Nrandom.theta1.sigma2.taurandomV1.Sc1.conjugate.R [,"p01"]~No, fun=function(x){c(

ks.D=( (paste(format(round(ks.test(x,"punif")$s,3), format="f", nsmall=3), symbols.ks(x),sep=""))),

p.0.05=( (paste(format(round({mean(x<0.05/2| x>(1-0.05/2))},3), format="f", nsmall=3), symbols.p05(x),sep=""))),

p.0.01=( (paste(format(round({mean(x<0.01/2| x>(1-0.01/2))},3), format="f", nsmall=3), symbols.p01(x),sep=""))))},method="cross",g=4)

x<-summary(results.dspp.Poisson.Nrandom.theta1.sigma2.taurandomV1.Sc1.conjugate.R [,"meanc"]~No, fun=function(x){c(

ks.D=( (paste(format(round(ks.test(x,"punif")$s,3), format="f", nsmall=3), symbols.ks(x),sep=""))),

p.0.05=( (paste(format(round({mean(x<0.05/2| x>(1-0.05/2))},3), format="f", nsmall=3), symbols.p05(x),sep=""))),

p.0.01=( (paste(format(round({mean(x<0.01/2| x>(1-0.01/2))},3), format="f", nsmall=3), symbols.p01(x),sep=""))))},method="cross",g=4)

#write.table("psp, d=meanc",file="test.txt",sep="\t",quote=F,row.names=F,append=T)

#write.table(as.data.frame(x)[,1:2],file="test.txt",sep="\t",quote=F,row.names=F,append=T)

print(x,prn=F)

x<-summary(results.dspp.Poisson.Nrandom.theta1.sigma2.taurandomV1.Sc1.conjugate.R [,"varc"]~No, fun=function(x){c(

ks.D=( (paste(format(round(ks.test(x,"punif")$s,3), format="f", nsmall=3), symbols.ks(x),sep=""))),

p.0.05=( (paste(format(round({mean(x<0.05/2| x>(1-0.05/2))},3), format="f", nsmall=3), symbols.p05(x),sep=""))),

p.0.01=( (paste(format(round({mean(x<0.01/2| x>(1-0.01/2))},3), format="f", nsmall=3), symbols.p01(x),sep=""))))},method="cross",g=4)

print(x,prn=F)

#write.table("psp, d=varc",file="test.txt",sep="\t",quote=F,row.names=F,append=T)

#write.table(as.data.frame(x)[,1:2],file="test.txt",sep="\t",quote=F,row.names=F,append=T)

**#R, dspp2, conjugate**

No<- results.dspp2.Poisson.Nrandom.theta1.sigma2.taurandomV1.Sc1.conjugate.R [,"N"]

x<-summary(results.dspp2.Poisson.Nrandom.theta1.sigma2.taurandomV1.Sc1.conjugate.R [,"LL"]~No, fun=function(x){c(

ks.D=( (paste(format(round(ks.test(x,"punif")$s,3), format="f", nsmall=3), symbols.ks(x),sep=""))),

p.0.05=( (paste(format(round({mean(x<0.05/2| x>(1-0.05/2))},3), format="f", nsmall=3), symbols.p05(x),sep=""))),

p.0.01=( (paste(format(round({mean(x<0.01/2| x>(1-0.01/2))},3), format="f", nsmall=3), symbols.p01(x),sep=""))))},method="cross",g=4)

print(x,prn=F)

#write.table("psp, d=LL",file="test.txt",sep="\t",quote=F,row.names=F,append=T)

#write.table(as.data.frame(x)[,1:2],file="test.txt",sep="\t",quote=F,row.names=F,append=T)

**#R, dspp3, conjugate**

No<- results.dspp3.Poisson.Nrandombis.theta1.sigma2.taurandomV1.Sc1.conjugate.R [,"N"]

summary(results.dspp3.Poisson.Nrandombis.theta1.sigma2.taurandomV1.Sc1.conjugate.R [,"mean"]~No, fun=function(x){c(

ks.D=( (paste(format(round(ks.test(x,"punif")$s,3), format="f", nsmall=3), symbols.ks(x),sep=""))),

p.0.05=( (paste(format(round({mean(x<0.05/2| x>(1-0.05/2))},3), format="f", nsmall=3), symbols.p05(x),sep=""))),

p.0.01=( (paste(format(round({mean(x<0.01/2| x>(1-0.01/2))},3), format="f", nsmall=3), symbols.p01(x),sep=""))))},method="cross",g=4)

summary(results.dspp3.Poisson.Nrandombis.theta1.sigma2.taurandomV1.Sc1.conjugate.R [,"meanc"]~No, fun=function(x){c(

ks.D=( (paste(format(round(ks.test(x,"punif")$s,3), format="f", nsmall=3), symbols.ks(x),sep=""))),

p.0.05=( (paste(format(round({mean(x<0.05/2| x>(1-0.05/2))},3), format="f", nsmall=3), symbols.p05(x),sep=""))),

p.0.01=( (paste(format(round({mean(x<0.01/2| x>(1-0.01/2))},3), format="f", nsmall=3), symbols.p01(x),sep=""))))},method="cross",g=4)

#### #=>A1: Normal, nspp, conjugate, Scenario 1

**#R, nspp, conjugate**

No<- results.nspp.Normal.Nrandom.tausigmarandom2.Sc1.conjugate.R [,"N"]

x<-summary(results.nspp.Normal.Nrandom.tausigmarandom2.Sc1.conjugate.R [,"mean"]~No, fun=function(x){c(

ks.D=( (paste(format(round(ks.test(x,"punif")$s,3), format="f", nsmall=3), symbols.ks(x),sep=""))),

p.0.05=( (paste(format(round({mean(x<0.05/2| x>(1-0.05/2))},3), format="f", nsmall=3), symbols.p05(x),sep=""))),

p.0.01=( (paste(format(round({mean(x<0.01/2| x>(1-0.01/2))},3), format="f", nsmall=3), symbols.p01(x),sep=""))))},method="cross",g=4)

print(x,prn=F)

x<-summary(results.nspp.Normal.Nrandom.tausigmarandom2.Sc1.conjugate.R [,"var"]~No, fun=function(x){c(

ks.D=( (paste(format(round(ks.test(x,"punif")$s,3), format="f", nsmall=3), symbols.ks(x),sep=""))),

p.0.05=( (paste(format(round({mean(x<0.05/2| x>(1-0.05/2))},3), format="f", nsmall=3), symbols.p05(x),sep=""))),

p.0.01=( (paste(format(round({mean(x<0.01/2| x>(1-0.01/2))},3), format="f", nsmall=3), symbols.p01(x),sep=""))))},method="cross",g=4)

print(x,prn=F)

x<-summary(results.nspp.Normal.Nrandom.tausigmarandom2.Sc1.conjugate.R [,"skew"]~No, fun=function(x){c(

ks.D=( (paste(format(round(ks.test(x,"punif")$s,3), format="f", nsmall=3), symbols.ks(x),sep=""))),

p.0.05=( (paste(format(round({mean(x<0.05/2| x>(1-0.05/2))},3), format="f", nsmall=3), symbols.p05(x),sep=""))),

p.0.01=( (paste(format(round({mean(x<0.01/2| x>(1-0.01/2))},3), format="f", nsmall=3), symbols.p01(x),sep=""))))},method="cross",g=4)

print(x,prn=F)

x<-summary(results.nspp.Normal.Nrandom.tausigmarandom2.Sc1.conjugate.R [,"kurt"]~No, fun=function(x){c(

ks.D=( (paste(format(round(ks.test(x,"punif")$s,3), format="f", nsmall=3), symbols.ks(x),sep=""))),

p.0.05=( (paste(format(round({mean(x<0.05/2| x>(1-0.05/2))},3), format="f", nsmall=3), symbols.p05(x),sep=""))),

p.0.01=( (paste(format(round({mean(x<0.01/2| x>(1-0.01/2))},3), format="f", nsmall=3), symbols.p01(x),sep=""))))},method="cross",g=4)

print(x,prn=F)

x<-summary(results.nspp.Normal.Nrandom.tausigmarandom2.Sc1.conjugate.R [,"Za"]~No, fun=function(x){c(

ks.D=( (paste(format(round(ks.test(x,"punif")$s,3), format="f", nsmall=3), symbols.ks(x),sep=""))),

p.0.05=( (paste(format(round({mean(x<0.05/2| x>(1-0.05/2))},3), format="f", nsmall=3), symbols.p05(x),sep=""))),

p.0.01=( (paste(format(round({mean(x<0.01/2| x>(1-0.01/2))},3), format="f", nsmall=3), symbols.p01(x),sep=""))))},method="cross",g=4)

print(x,prn=F)

#### #=>A1: Normal, spp, dspp et dspp2 & dspp3, conjugate, Scenario 1

**#R, spp, conjugate**

No<- results.spp.Normal.Nrandom.tausigmarandom2.Sc1.conjugate.R [,"N"]

print(x,prn=F)

**x<-summary**(results.spp.Normal.Nrandom.tausigmarandom2.Sc1.conjugate.R [,"mean"]~No, fun=function(x){c(

ks.D=( (paste(format(round(ks.test(x,"punif")$s,3), format="f", nsmall=3), symbols.ks(x),sep=""))),

p.0.05=( (paste(format(round({mean(x<0.05/2| x>(1-0.05/2))},3), format="f", nsmall=3), symbols.p05(x),sep=""))),

p.0.01=( (paste(format(round({mean(x<0.01/2| x>(1-0.01/2))},3), format="f", nsmall=3), symbols.p01(x),sep=""))))},method="cross",g=4)

print(x,prn=F)

x<-summary(results.spp.Normal.Nrandom.tausigmarandom2.Sc1.conjugate.R [,"var"]~No, fun=function(x){c(

ks.D=( (paste(format(round(ks.test(x,"punif")$s,3), format="f", nsmall=3), symbols.ks(x),sep=""))),

p.0.05=( (paste(format(round({mean(x<0.05/2| x>(1-0.05/2))},3), format="f", nsmall=3), symbols.p05(x),sep=""))),

p.0.01=( (paste(format(round({mean(x<0.01/2| x>(1-0.01/2))},3), format="f", nsmall=3), symbols.p01(x),sep=""))))},method="cross",g=4)

print(x,prn=F)

**#R, dspp, conjugate**

No<- results.dspp.Normal.Nrandom.tausigmarandom2.Sc1.conjugate.R [,"N"]

print(x,prn=F)

x<-summary(results.dspp.Normal.Nrandom.tausigmarandom2.Sc1.conjugate.R [,"plt0"]~No, fun=function(x){c(

ks.D=( (paste(format(round(ks.test(x,"punif")$s,3), format="f", nsmall=3), symbols.ks(x),sep=""))),

p.0.05=( (paste(format(round({mean(x<0.05/2| x>(1-0.05/2))},3), format="f", nsmall=3), symbols.p05(x),sep=""))),

p.0.01=( (paste(format(round({mean(x<0.01/2| x>(1-0.01/2))},3), format="f", nsmall=3), symbols.p01(x),sep=""))))},method="cross",g=4)

print(x,prn=F)

**x<-summary**(results.dspp.Normal.Nrandom.tausigmarandom2.Sc1.conjugate.R [,"meanc"]~No, fun=function(x){c(

ks.D=( (paste(format(round(ks.test(x,"punif")$s,3), format="f", nsmall=3), symbols.ks(x),sep=""))),

p.0.05=( (paste(format(round({mean(x<0.05/2| x>(1-0.05/2))},3), format="f", nsmall=3), symbols.p05(x),sep=""))),

p.0.01=( (paste(format(round({mean(x<0.01/2| x>(1-0.01/2))},3), format="f", nsmall=3), symbols.p01(x),sep=""))))},method="cross",g=4)

print(x,prn=F)

x<-summary(results.dspp.Normal.Nrandom.tausigmarandom2.Sc1.conjugate.R [,"varc"]~No, fun=function(x){c(

ks.D=( (paste(format(round(ks.test(x,"punif")$s,3), format="f", nsmall=3), symbols.ks(x),sep=""))),

p.0.05=( (paste(format(round({mean(x<0.05/2| x>(1-0.05/2))},3), format="f", nsmall=3), symbols.p05(x),sep=""))),

p.0.01=( (paste(format(round({mean(x<0.01/2| x>(1-0.01/2))},3), format="f", nsmall=3), symbols.p01(x),sep=""))))},method="cross",g=4)

print(x,prn=F)

**#R, dspp2, conjugate**

No<- results.dspp2.Normal.Nrandom.tausigmarandom2.Sc1.conjugate.R [,"N"]

print(x,prn=F)

x<-summary(results.dspp2.Normal.Nrandom.tausigmarandom2.Sc1.conjugate.R [,"LL"]~No, fun=function(x){c(

ks.D=( (paste(format(round(ks.test(x,"punif")$s,3), format="f", nsmall=3), symbols.ks(x),sep=""))),

p.0.05=( (paste(format(round({mean(x<0.05/2| x>(1-0.05/2))},3), format="f", nsmall=3), symbols.p05(x),sep=""))),

p.0.01=( (paste(format(round({mean(x<0.01/2| x>(1-0.01/2))},3), format="f", nsmall=3), symbols.p01(x),sep=""))))},method="cross",g=4)

print(x,prn=F)

**#R, dspp3, conjugate**

No<- results.dspp3.Normal.Nrandombig.tausigmarandom2.Sc1.conjugate.R [,"N"]

x<-summary(results.dspp3.Normal.Nrandombig.tausigmarandom2.Sc1.conjugate.R [,"mean"]~No, fun=function(x){c(

ks.D=( (paste(format(round(ks.test(x,"punif")$s,3), format="f", nsmall=3), symbols.ks(x),sep=""))),

p.0.05=( (paste(format(round({mean(x<0.05/2| x>(1-0.05/2))},3), format="f", nsmall=3), symbols.p05(x),sep=""))),

p.0.01=( (paste(format(round({mean(x<0.01/2| x>(1-0.01/2))},3), format="f", nsmall=3), symbols.p01(x),sep=""))))},method="cross",g=4)

print(x,prn=F)

**x<-summary**(results.dspp3.Normal.Nrandombig.tausigmarandom2.Sc1.conjugate.R [,"meanc"]~No, fun=function(x){c(

ks.D=( (paste(format(round(ks.test(x,"punif")$s,3), format="f", nsmall=3), symbols.ks(x),sep=""))),

p.0.05=( (paste(format(round({mean(x<0.05/2| x>(1-0.05/2))},3), format="f", nsmall=3), symbols.p05(x),sep=""))),

p.0.01=( (paste(format(round({mean(x<0.01/2| x>(1-0.01/2))},3), format="f", nsmall=3), symbols.p01(x),sep=""))))},method="cross",g=4)

print(x,prn=F)

#### #=>A1: Bernoulli, nspp, conjugate, Scebario 1

**#R, nspp, conjugate**

No<- results.nspp.Bernoulli.Nrandom.thetarandom5.Sc1.conjugate.R [,"N"]

**x<-summary**(results.nspp.Bernoulli.Nrandom.thetarandom5.Sc1.conjugate.R [,"mean"]~No, fun=function(x){c(

ks.D=( (paste(format(round(ks.test(x,"punif")$s,3), format="f", nsmall=3), symbols.ks(x),sep=""))),

p.0.05=( (paste(format(round({mean(x<0.05/2| x>(1-0.05/2))},3), format="f", nsmall=3), symbols.p05(x),sep=""))),

p.0.01=( (paste(format(round({mean(x<0.01/2| x>(1-0.01/2))},3), format="f", nsmall=3), symbols.p01(x),sep=""))))},method="cross",g=4)

print(x,prn=F)

x<-summary(results.nspp.Bernoulli.Nrandom.thetarandom5.Sc1.conjugate.R [,"var"]~No, fun=function(x){c(

ks.D=( (paste(format(round(ks.test(x,"punif")$s,3), format="f", nsmall=3), symbols.ks(x),sep=""))),

p.0.05=( (paste(format(round({mean(x<0.05/2| x>(1-0.05/2))},3), format="f", nsmall=3), symbols.p05(x),sep=""))),

p.0.01=( (paste(format(round({mean(x<0.01/2| x>(1-0.01/2))},3), format="f", nsmall=3), symbols.p01(x),sep=""))))},method="cross",g=4)

print(x,prn=F)

x<-summary(results.nspp.Bernoulli.Nrandom.thetarandom5.Sc1.conjugate.R [,"skew"]~No, fun=function(x){c(

ks.D=( (paste(format(round(ks.test(x,"punif")$s,3), format="f", nsmall=3), symbols.ks(x),sep=""))),

p.0.05=( (paste(format(round({mean(x<0.05/2| x>(1-0.05/2))},3), format="f", nsmall=3), symbols.p05(x),sep=""))),

p.0.01=( (paste(format(round({mean(x<0.01/2| x>(1-0.01/2))},3), format="f", nsmall=3), symbols.p01(x),sep=""))))},method="cross",g=4)

print(x,prn=F)

x<-summary(results.nspp.Bernoulli.Nrandom.thetarandom5.Sc1.conjugate.R [,"kurt"]~No, fun=function(x){c(

ks.D=( (paste(format(round(ks.test(x,"punif")$s,3), format="f", nsmall=3), symbols.ks(x),sep=""))),

p.0.05=( (paste(format(round({mean(x<0.05/2| x>(1-0.05/2))},3), format="f", nsmall=3), symbols.p05(x),sep=""))),

p.0.01=( (paste(format(round({mean(x<0.01/2| x>(1-0.01/2))},3), format="f", nsmall=3), symbols.p01(x),sep=""))))},method="cross",g=4)

print(x,prn=F)

x<-summary(results.nspp.Bernoulli.Nrandom.thetarandom5.Sc1.conjugate.R [,"Za"]~No, fun=function(x){c(

ks.D=( (paste(format(round(ks.test(x,"punif")$s,3), format="f", nsmall=3), symbols.ks(x),sep=""))),

p.0.05=( (paste(format(round({mean(x<0.05/2| x>(1-0.05/2))},3), format="f", nsmall=3), symbols.p05(x),sep=""))),

p.0.01=( (paste(format(round({mean(x<0.01/2| x>(1-0.01/2))},3), format="f", nsmall=3), symbols.p01(x),sep=""))))},method="cross",g=4)

print(x,prn=F)

#### #=>A1: Bernoulli, spp, dspp, dspp2, conjugate, Scenario 1

**#R, spp, conjugate**

No<- results.spp.Bernoulli.Nrandom.thetarandom5.Sc1.conjugate.R [,"N"]

**x<-summary**(results.spp.Bernoulli.Nrandom.thetarandom5.Sc1.conjugate.R [,"mean"]~No, fun=function(x){c(

ks.D=( (paste(format(round(ks.test(x,"punif")$s,3), format="f", nsmall=3), symbols.ks(x),sep=""))),

p.0.05=( (paste(format(round({mean(x<0.05/2| x>(1-0.05/2))},3), format="f", nsmall=3), symbols.p05(x),sep=""))),

p.0.01=( (paste(format(round({mean(x<0.01/2| x>(1-0.01/2))},3), format="f", nsmall=3), symbols.p01(x),sep=""))))},method="cross",g=4)

print(x,prn=F)

x<-summary(results.spp.Bernoulli.Nrandom.thetarandom5.Sc1.conjugate.R [,"var"]~No, fun=function(x){c(

ks.D=( (paste(format(round(ks.test(x,"punif")$s,3), format="f", nsmall=3), symbols.ks(x),sep=""))),

p.0.05=( (paste(format(round({mean(x<0.05/2| x>(1-0.05/2))},3), format="f", nsmall=3), symbols.p05(x),sep=""))),

p.0.01=( (paste(format(round({mean(x<0.01/2| x>(1-0.01/2))},3), format="f", nsmall=3), symbols.p01(x),sep=""))))},method="cross",g=4)

print(x,prn=F)

**#R, dspp, conjugate**

No<- results.dspp.Bernoulli.Nrandom.thetarandom5.Sc1.conjugate.R [,"N"]

x<-summary(results.dspp.Bernoulli.Nrandom.thetarandom5.Sc1.conjugate.R [,"p0"]~No, fun=function(x){c(

ks.D=( (paste(format(round(ks.test(x,"punif")$s,3), format="f", nsmall=3), symbols.ks(x),sep=""))),

p.0.05=( (paste(format(round({mean(x<0.05/2| x>(1-0.05/2))},3), format="f", nsmall=3), symbols.p05(x),sep=""))),

p.0.01=( (paste(format(round({mean(x<0.01/2| x>(1-0.01/2))},3), format="f", nsmall=3), symbols.p01(x),sep=""))))},method="cross",g=4)

print(x,prn=F)

######## not to be copied

x<-summary(results.dspp.Bernoulli.Nrandom.thetarandom5.Sc1.conjugate.R [,"p01"]~No, fun=function(x){c(

ks.D=( (paste(format(round(ks.test(x,"punif")$s,3), format="f", nsmall=3), symbols.ks(x),sep=""))),

p.0.05=( (paste(format(round({mean(x<0.05/2| x>(1-0.05/2))},3), format="f", nsmall=3), symbols.p05(x),sep=""))),

p.0.01=( (paste(format(round({mean(x<0.01/2| x>(1-0.01/2))},3), format="f", nsmall=3), symbols.p01(x),sep=""))))},method="cross",g=4)

print(x,prn=F)

**x<-summary**(results.dspp.Bernoulli.Nrandom.thetarandom5.Sc1.conjugate.R [,"meanc"]~No, fun=function(x){c(

ks.D=( (paste(format(round(ks.test(x,"punif")$s,3), format="f", nsmall=3), symbols.ks(x),sep=""))),

p.0.05=( (paste(format(round({mean(x<0.05/2| x>(1-0.05/2))},3), format="f", nsmall=3), symbols.p05(x),sep=""))),

p.0.01=( (paste(format(round({mean(x<0.01/2| x>(1-0.01/2))},3), format="f", nsmall=3), symbols.p01(x),sep=""))))},method="cross",g=4)

print(x,prn=F)

x<-summary(results.dspp.Bernoulli.Nrandom.thetarandom5.Sc1.conjugate.R [,"varc"]~No, fun=function(x){c(

ks.D=( (paste(format(round(ks.test(x,"punif")$s,3), format="f", nsmall=3), symbols.ks(x),sep=""))),

p.0.05=( (paste(format(round({mean(x<0.05/2| x>(1-0.05/2))},3), format="f", nsmall=3), symbols.p05(x),sep=""))),

p.0.01=( (paste(format(round({mean(x<0.01/2| x>(1-0.01/2))},3), format="f", nsmall=3), symbols.p01(x),sep=""))))},method="cross",g=4)

print(x,prn=F)

**#R, dspp2, conjugate**

No<- results.dspp2.Bernoulli.Nrandom.thetarandom5.Sc1.conjugate.R [,"N"]

x<-summary(results.dspp2.Bernoulli.Nrandom.thetarandom5.Sc1.conjugate.R [,"LL"]~No, fun=function(x){c(

ks.D=( (paste(format(round(ks.test(x,"punif")$s,3), format="f", nsmall=3), symbols.ks(x),sep=""))),

p.0.05=( (paste(format(round({mean(x<0.05/2| x>(1-0.05/2))},3), format="f", nsmall=3), symbols.p05(x),sep=""))),

p.0.01=( (paste(format(round({mean(x<0.01/2| x>(1-0.01/2))},3), format="f", nsmall=3), symbols.p01(x),sep=""))))},method="cross",g=4)

print(x,prn=F)

**#R, dspp3, conjugate**

No<- results.dspp3.Bernoulli.Nrandombig.thetarandom5.Sc1.conjugate.R [,"N"]

summary(results.dspp3.Bernoulli.Nrandombig.thetarandom5.Sc1.conjugate.R [,"mean"]~No, fun=function(x){c(

ks.D=( (paste(format(round(ks.test(x,"punif")$s,3), format="f", nsmall=3), symbols.ks(x),sep=""))),

p.0.05=( (paste(format(round({mean(x<0.05/2| x>(1-0.05/2))},3), format="f", nsmall=3), symbols.p05(x),sep=""))),

p.0.01=( (paste(format(round({mean(x<0.01/2| x>(1-0.01/2))},3), format="f", nsmall=3), symbols.p01(x),sep=""))))},method="cross",g=4)

summary(results.dspp3.Bernoulli.Nrandombig.thetarandom5.Sc1.conjugate.R [,"meanc"]~No, fun=function(x){c(

ks.D=( (paste(format(round(ks.test(x,"punif")$s,3), format="f", nsmall=3), symbols.ks(x),sep=""))),

p.0.05=( (paste(format(round({mean(x<0.05/2| x>(1-0.05/2))},3), format="f", nsmall=3), symbols.p05(x),sep=""))),

p.0.01=( (paste(format(round({mean(x<0.01/2| x>(1-0.01/2))},3), format="f", nsmall=3), symbols.p01(x),sep=""))))},method="cross",g=4)

## Tables for Text S2 (Scenario 2)

#### #=>A2: Poisson, nspp, conjugate

**#R, nspp, conjugate, juste sigma (en masqué)**

No<- results.nspp.Poisson.Nrandom.theta1.sigma2.taurandomV1.Sc2.conjugate.R [,"N"]

mult.sigma<- round(results.nspp.Poisson.Nrandom.theta1.sigma2.taurandomV1.Sc2.conjugate.R[,"musigma0"],digits=2)

**x<-summary(**results.nspp.Poisson.Nrandom.theta1.sigma2.taurandomV1.Sc2.conjugate.R [,"mean"]~mult.sigma, fun=function(x){c(

ks.D=( (paste(" ks.D=",format(round(ks.test(x,"punif")$s,3), format="f", dig=3), symbols.ks(x),sep=""))),

p.0.05=( (paste(" p.05=",format(round({mean(x<0.05/2| x>(1-0.05/2))},3), format="f", nsmall=3), symbols.p05(x),sep=""))),

p.0.01=( (paste(" p.01=",format(round({mean(x<0.01/2| x>(1-0.01/2))},3), format="f", nsmall=3), symbols.p01(x),sep=""))))},method="cross",g=4)

print(x,prn=F)

write.table("psp, t=mean",file="test.txt",sep="\t",quote=F,row.names=F,append=T)

write.table(as.data.frame(x)[,1:2],file="test.txt",sep="\t",quote=F,row.names=F,append=T)

x<-summary(results.nspp.Poisson.Nrandom.theta1.sigma2.taurandomV1.Sc2.conjugate.R [,"var"]~mult.sigma, fun=function(x){c(

ks.D=( (paste(" ks.D=",format(round(ks.test(x,"punif")$s,3), format="f", dig=3), symbols.ks(x),sep=""))),

p.0.05=( (paste(" p.05=",format(round({mean(x<0.05/2| x>(1-0.05/2))},3), format="f", nsmall=3), symbols.p05(x),sep=""))),

p.0.01=( (paste(" p.01=",format(round({mean(x<0.01/2| x>(1-0.01/2))},3), format="f", nsmall=3), symbols.p01(x),sep=""))))},method="cross",g=4)

print(x,prn=F)

x<-summary(results.nspp.Poisson.Nrandom.theta1.sigma2.taurandomV1.Sc2.conjugate.R [,"skew"]~ mult.sigma, fun=function(x){c(

ks.D=( (paste(" ks.D=",format(round(ks.test(x,"punif")$s,3), format="f", dig=3), symbols.ks(x),sep=""))),

p.0.05=( (paste(" p.05=",format(round({mean(x<0.05/2| x>(1-0.05/2))},3), format="f", nsmall=3), symbols.p05(x),sep=""))),

p.0.01=( (paste(" p.01=",format(round({mean(x<0.01/2| x>(1-0.01/2))},3), format="f", nsmall=3), symbols.p01(x),sep=""))))},method="cross",g=4)

print(x,prn=F)

x<-summary(results.nspp.Poisson.Nrandom.theta1.sigma2.taurandomV1.Sc2.conjugate.R [,"kurt"]~ mult.sigma, fun=function(x){c(

ks.D=( (paste(" ks.D=",format(round(ks.test(x,"punif")$s,3), format="f", dig=3), symbols.ks(x),sep=""))),

p.0.05=( (paste(" p.05=",format(round({mean(x<0.05/2| x>(1-0.05/2))},3), format="f", nsmall=3), symbols.p05(x),sep=""))),

p.0.01=( (paste(" p.01=",format(round({mean(x<0.01/2| x>(1-0.01/2))},3), format="f", nsmall=3), symbols.p01(x),sep=""))))},method="cross",g=4)

print(x,prn=F)

x<-summary(results.nspp.Poisson.Nrandom.theta1.sigma2.taurandomV1.Sc2.conjugate.R [,"Za"]~ mult.sigma, fun=function(x){c(

ks.D=( (paste(" ks.D=",format(round(ks.test(x,"punif")$s,3), format="f", dig=3), symbols.ks(x),sep=""))),

p.0.05=( (paste(" p.05=",format(round({mean(x<0.05/2| x>(1-0.05/2))},3), format="f", nsmall=3), symbols.p05(x),sep=""))),

p.0.01=( (paste(" p.01=",format(round({mean(x<0.01/2| x>(1-0.01/2))},3), format="f", nsmall=3), symbols.p01(x),sep=""))))},method="cross",g=4)

print(x,prn=F)

**#R, nspp, conjugate, sigma et N**

No<- results.nspp.Poisson.Nrandom.theta1.sigma2.taurandomV1.Sc2.conjugate.R [,"N"]

mult.sigma<- round(results.nspp.Poisson.Nrandom.theta1.sigma2.taurandomV1.Sc2.conjugate.R[,"musigma0"],digits=2)

**x<-summary(**results.nspp.Poisson.Nrandom.theta1.sigma2.taurandomV1.Sc2.conjugate.R [,"mean"]~mult.sigma+No, fun=function(x){c(

ks.D=( (paste(" ks.D=",format(round(ks.test(x,"punif")$s,3), format="f", nsmall=3), symbols.ks(x),sep=""))),

p.0.05=( (paste(" p.05=",format(round({mean(x<0.05/2| x>(1-0.05/2))},3), format="f", nsmall=3), symbols.p05(x),sep=""))),

p.0.01=( (paste(" p.01=",format(round({mean(x<0.01/2| x>(1-0.01/2))},3), format="f", nsmall=3), symbols.p01(x),sep=""))))},method="cross",g=4)

print(x,prn=F)

x<-summary(results.nspp.Poisson.Nrandom.theta1.sigma2.taurandomV1.Sc2.conjugate.R [,"var"]~mult.sigma+No, fun=function(x){c(

ks.D=( (paste(" ks.D=",format(round(ks.test(x,"punif")$s,3), format="f", nsmall=3), symbols.ks(x),sep=""))),

p.0.05=( (paste(" p.05=",format(round({mean(x<0.05/2| x>(1-0.05/2))},3), format="f", nsmall=3), symbols.p05(x),sep=""))),

p.0.01=( (paste(" p.01=",format(round({mean(x<0.01/2| x>(1-0.01/2))},3), format="f", nsmall=3), symbols.p01(x),sep=""))))},method="cross",g=4)

print(x,prn=F)

x<-summary(results.nspp.Poisson.Nrandom.theta1.sigma2.taurandomV1.Sc2.conjugate.R [,"skew"]~mult.sigma+No, fun=function(x){c(

ks.D=( (paste(" ks.D=",format(round(ks.test(x,"punif")$s,3), format="f", nsmall=3), symbols.ks(x),sep=""))),

p.0.05=( (paste(" p.05=",format(round({mean(x<0.05/2| x>(1-0.05/2))},3), format="f", nsmall=3), symbols.p05(x),sep=""))),

p.0.01=( (paste(" p.01=",format(round({mean(x<0.01/2| x>(1-0.01/2))},3), format="f", nsmall=3), symbols.p01(x),sep=""))))},method="cross",g=4)

print(x,prn=F)

x<-summary(results.nspp.Poisson.Nrandom.theta1.sigma2.taurandomV1.Sc2.conjugate.R [,"kurt"]~mult.sigma+No, fun=function(x){c(

ks.D=( (paste(" ks.D=",format(round(ks.test(x,"punif")$s,3), format="f", nsmall=3), symbols.ks(x),sep=""))),

p.0.05=( (paste(" p.05=",format(round({mean(x<0.05/2| x>(1-0.05/2))},3), format="f", nsmall=3), symbols.p05(x),sep=""))),

p.0.01=( (paste(" p.01=",format(round({mean(x<0.01/2| x>(1-0.01/2))},3), format="f", nsmall=3), symbols.p01(x),sep=""))))},method="cross",g=4)

print(x,prn=F)

x<-summary(results.nspp.Poisson.Nrandom.theta1.sigma2.taurandomV1.Sc2.conjugate.R [,"Za"]~mult.sigma+No, fun=function(x){c(

ks.D=( (paste(" ks.D=",format(round(ks.test(x,"punif")$s,3), format="f", nsmall=3), symbols.ks(x),sep=""))),

p.0.05=( (paste(" p.05=",format(round({mean(x<0.05/2| x>(1-0.05/2))},3), format="f", nsmall=3), symbols.p05(x),sep=""))),

p.0.01=( (paste(" p.01=",format(round({mean(x<0.01/2| x>(1-0.01/2))},3), format="f", nsmall=3), symbols.p01(x),sep=""))))},method="cross",g=4)

print(x,prn=F)

#### #=>A2: Poisson, spp et dspp & dspp2, conjugate

**#R, spp, conjugate, sigma et N**

No<- results.spp.Poisson.Nrandom.theta1.sigma2.taurandomV1.Sc2.conjugate.R [,"N"]

mult.sigma<- round(results.spp.Poisson.Nrandom.theta1.sigma2.taurandomV1.Sc2.conjugate.R[,"musigma0"],digits=2)

**x<-summary(**results.spp.Poisson.Nrandom.theta1.sigma2.taurandomV1.Sc2.conjugate.R [,"mean"]~mult.sigma+No, fun=function(x){c(

ks.D=( (paste(" ks.D=",format(round(ks.test(x,"punif")$s,3), format="f", nsmall=3), symbols.ks(x),sep=""))),

p.0.05=( (paste(" p.05=",format(round({mean(x<0.05/2| x>(1-0.05/2))},3), format="f", nsmall=3), symbols.p05(x),sep=""))),

p.0.01=( (paste(" p.01=",format(round({mean(x<0.01/2| x>(1-0.01/2))},3), format="f", nsmall=3), symbols.p01(x),sep=""))))},method="cross",g=4)

print(x,prn=F)

x<-summary(results.spp.Poisson.Nrandom.theta1.sigma2.taurandomV1.Sc2.conjugate.R [,"var"]~mult.sigma+No, fun=function(x){c(

ks.D=( (paste(" ks.D=",format(round(ks.test(x,"punif")$s,3), format="f", nsmall=3), symbols.ks(x),sep=""))),

p.0.05=( (paste(" p.05=",format(round({mean(x<0.05/2| x>(1-0.05/2))},3), format="f", nsmall=3), symbols.p05(x),sep=""))),

p.0.01=( (paste(" p.01=",format(round({mean(x<0.01/2| x>(1-0.01/2))},3), format="f", nsmall=3), symbols.p01(x),sep=""))))},method="cross",g=4)

print(x,prn=F)

**#R, dspp, conjugate, sigma et N**

No<- results.dspp.Poisson.Nrandom.theta1.sigma2.taurandomV1.Sc2.conjugate.R [,"N"]

mult.sigma<- round(results.dspp.Poisson.Nrandom.theta1.sigma2.taurandomV1.Sc2.conjugate.R[,"musigma0"],digits=2)

**x<-summary(**results.dspp.Poisson.Nrandom.theta1.sigma2.taurandomV1.Sc2.conjugate.R [,"p0"]~mult.sigma+No, fun=function(x){c(

ks.D=( (paste(" ks.D=",format(round(ks.test(x,"punif")$s,3), format="f", nsmall=3), symbols.ks(x),sep=""))),

p.0.05=( (paste(" p.05=",format(round({mean(x<0.05/2| x>(1-0.05/2))},3), format="f", nsmall=3), symbols.p05(x),sep=""))),

p.0.01=( (paste(" p.01=",format(round({mean(x<0.01/2| x>(1-0.01/2))},3), format="f", nsmall=3), symbols.p01(x),sep=""))))},method="cross",g=4)

print(x,prn=F)

x<-summary(results.dspp.Poisson.Nrandom.theta1.sigma2.taurandomV1.Sc2.conjugate.R [,"p01"]~mult.sigma+No, fun=function(x){c(

ks.D=( (paste(" ks.D=",format(round(ks.test(x,"punif")$s,3), format="f", nsmall=3), symbols.ks(x),sep=""))),

p.0.05=( (paste(" p.05=",format(round({mean(x<0.05/2| x>(1-0.05/2))},3), format="f", nsmall=3), symbols.p05(x),sep=""))),

p.0.01=( (paste(" p.01=",format(round({mean(x<0.01/2| x>(1-0.01/2))},3), format="f", nsmall=3), symbols.p01(x),sep=""))))},method="cross",g=4)

print(x,prn=F)

**x<-summary(**results.dspp.Poisson.Nrandom.theta1.sigma2.taurandomV1.Sc2.conjugate.R [,"meanc"]~mult.sigma+No, fun=function(x){c(

ks.D=( (paste(" ks.D=",format(round(ks.test(x,"punif")$s,3), format="f", nsmall=3), symbols.ks(x),sep=""))),

p.0.05=( (paste(" p.05=",format(round({mean(x<0.05/2| x>(1-0.05/2))},3), format="f", nsmall=3), symbols.p05(x),sep=""))),

p.0.01=( (paste(" p.01=",format(round({mean(x<0.01/2| x>(1-0.01/2))},3), format="f", nsmall=3), symbols.p01(x),sep=""))))},method="cross",g=4)

print(x,prn=F)

x<-summary(results.dspp.Poisson.Nrandom.theta1.sigma2.taurandomV1.Sc2.conjugate.R [,"varc"]~mult.sigma+No, fun=function(x){c(

ks.D=( (paste(" ks.D=",format(round(ks.test(x,"punif")$s,3), format="f", nsmall=3), symbols.ks(x),sep=""))),

p.0.05=( (paste(" p.05=",format(round({mean(x<0.05/2| x>(1-0.05/2))},3), format="f", nsmall=3), symbols.p05(x),sep=""))),

p.0.01=( (paste(" p.01=",format(round({mean(x<0.01/2| x>(1-0.01/2))},3), format="f", nsmall=3), symbols.p01(x),sep=""))))},method="cross",g=4)

print(x,prn=F)

**#R, dspp2, conjugate, sigma et N**

No<- results.dspp2.Poisson.Nrandom.theta1.sigma2.taurandomV1.Sc2.conjugate.R [,"N"]

mult.sigma<- round(results.dspp2.Poisson.Nrandom.theta1.sigma2.taurandomV1.Sc2.conjugate.R[,"musigma0"],digits=2)

**x<-summary(**results.dspp2.Poisson.Nrandom.theta1.sigma2.taurandomV1.Sc2.conjugate.R [,"LL"]~mult.sigma+No, fun=function(x){c(

ks.D=( (paste(" ks.D=",format(round(ks.test(x,"punif")$s,3), format="f", nsmall=3), symbols.ks(x),sep=""))),

p.0.05=( (paste(" p.05=",format(round({mean(x<0.05/2| x>(1-0.05/2))},3), format="f", nsmall=3), symbols.p05(x),sep=""))),

p.0.01=( (paste(" p.01=",format(round({mean(x<0.01/2| x>(1-0.01/2))},3), format="f", nsmall=3), symbols.p01(x),sep=""))))},method="cross",g=4)

print(x,prn=F)

#### #=>A2: Poisson, nspp, conjugate

**#R, nspp, conjugate, juste sigma (en masqué)**

No<- results.nspp.Poisson.Nrandom.theta1.sigma2.taurandomV1.Sc2.conjugate.R [,"N"]

mult.sigma<- round(results.nspp.Poisson.Nrandom.theta1.sigma2.taurandomV1.Sc2.conjugate.R[,"musigma0"],digits=2)

**x<-summary(**results.nspp.Poisson.Nrandom.theta1.sigma2.taurandomV1.Sc2.conjugate.R [,"mean"]~mult.sigma, fun=function(x){c(

ks.D=( (paste(" ks.D=",format(round(ks.test(x,"punif")$s,3), format="f", dig=3), symbols.ks(x),sep=""))),

p.0.05=( (paste(" p.05=",format(round({mean(x<0.05/2| x>(1-0.05/2))},3), format="f", nsmall=3), symbols.p05(x),sep=""))),

p.0.01=( (paste(" p.01=",format(round({mean(x<0.01/2| x>(1-0.01/2))},3), format="f", nsmall=3), symbols.p01(x),sep=""))))},method="cross",g=4)

print(x,prn=F)

write.table("psp, t=mean",file="test.txt",sep="\t",quote=F,row.names=F,append=T)

write.table(as.data.frame(x)[,1:2],file="test.txt",sep="\t",quote=F,row.names=F,append=T)

x<-summary(results.nspp.Poisson.Nrandom.theta1.sigma2.taurandomV1.Sc2.conjugate.R [,"var"]~mult.sigma, fun=function(x){c(

ks.D=( (paste(" ks.D=",format(round(ks.test(x,"punif")$s,3), format="f", dig=3), symbols.ks(x),sep=""))),

p.0.05=( (paste(" p.05=",format(round({mean(x<0.05/2| x>(1-0.05/2))},3), format="f", nsmall=3), symbols.p05(x),sep=""))),

p.0.01=( (paste(" p.01=",format(round({mean(x<0.01/2| x>(1-0.01/2))},3), format="f", nsmall=3), symbols.p01(x),sep=""))))},method="cross",g=4)

print(x,prn=F)

x<-summary(results.nspp.Poisson.Nrandom.theta1.sigma2.taurandomV1.Sc2.conjugate.R [,"skew"]~ mult.sigma, fun=function(x){c(

ks.D=( (paste(" ks.D=",format(round(ks.test(x,"punif")$s,3), format="f", dig=3), symbols.ks(x),sep=""))),

p.0.05=( (paste(" p.05=",format(round({mean(x<0.05/2| x>(1-0.05/2))},3), format="f", nsmall=3), symbols.p05(x),sep=""))),

p.0.01=( (paste(" p.01=",format(round({mean(x<0.01/2| x>(1-0.01/2))},3), format="f", nsmall=3), symbols.p01(x),sep=""))))},method="cross",g=4)

print(x,prn=F)

x<-summary(results.nspp.Poisson.Nrandom.theta1.sigma2.taurandomV1.Sc2.conjugate.R [,"kurt"]~ mult.sigma, fun=function(x){c(

ks.D=( (paste(" ks.D=",format(round(ks.test(x,"punif")$s,3), format="f", dig=3), symbols.ks(x),sep=""))),

p.0.05=( (paste(" p.05=",format(round({mean(x<0.05/2| x>(1-0.05/2))},3), format="f", nsmall=3), symbols.p05(x),sep=""))),

p.0.01=( (paste(" p.01=",format(round({mean(x<0.01/2| x>(1-0.01/2))},3), format="f", nsmall=3), symbols.p01(x),sep=""))))},method="cross",g=4)

print(x,prn=F)

x<-summary(results.nspp.Poisson.Nrandom.theta1.sigma2.taurandomV1.Sc2.conjugate.R [,"Za"]~ mult.sigma, fun=function(x){c(

ks.D=( (paste(" ks.D=",format(round(ks.test(x,"punif")$s,3), format="f", dig=3), symbols.ks(x),sep=""))),

p.0.05=( (paste(" p.05=",format(round({mean(x<0.05/2| x>(1-0.05/2))},3), format="f", nsmall=3), symbols.p05(x),sep=""))),

p.0.01=( (paste(" p.01=",format(round({mean(x<0.01/2| x>(1-0.01/2))},3), format="f", nsmall=3), symbols.p01(x),sep=""))))},method="cross",g=4)

print(x,prn=F)

**#R, nspp, conjugate, sigma et N**

No<- results.nspp.Poisson.Nrandom.theta1.sigma2.taurandomV1.Sc2.conjugate.R [,"N"]

mult.sigma<- round(results.nspp.Poisson.Nrandom.theta1.sigma2.taurandomV1.Sc2.conjugate.R[,"musigma0"],digits=2)

**x<-summary(**results.nspp.Poisson.Nrandom.theta1.sigma2.taurandomV1.Sc2.conjugate.R [,"mean"]~mult.sigma+No, fun=function(x){c(

ks.D=( (paste(" ks.D=",format(round(ks.test(x,"punif")$s,3), format="f", nsmall=3), symbols.ks(x),sep=""))),

p.0.05=( (paste(" p.05=",format(round({mean(x<0.05/2| x>(1-0.05/2))},3), format="f", nsmall=3), symbols.p05(x),sep=""))),

p.0.01=( (paste(" p.01=",format(round({mean(x<0.01/2| x>(1-0.01/2))},3), format="f", nsmall=3), symbols.p01(x),sep=""))))},method="cross",g=4)

print(x,prn=F)

x<-summary(results.nspp.Poisson.Nrandom.theta1.sigma2.taurandomV1.Sc2.conjugate.R [,"var"]~mult.sigma+No, fun=function(x){c(

ks.D=( (paste(" ks.D=",format(round(ks.test(x,"punif")$s,3), format="f", nsmall=3), symbols.ks(x),sep=""))),

p.0.05=( (paste(" p.05=",format(round({mean(x<0.05/2| x>(1-0.05/2))},3), format="f", nsmall=3), symbols.p05(x),sep=""))),

p.0.01=( (paste(" p.01=",format(round({mean(x<0.01/2| x>(1-0.01/2))},3), format="f", nsmall=3), symbols.p01(x),sep=""))))},method="cross",g=4)

print(x,prn=F)

x<-summary(results.nspp.Poisson.Nrandom.theta1.sigma2.taurandomV1.Sc2.conjugate.R [,"skew"]~mult.sigma+No, fun=function(x){c(

ks.D=( (paste(" ks.D=",format(round(ks.test(x,"punif")$s,3), format="f", nsmall=3), symbols.ks(x),sep=""))),

p.0.05=( (paste(" p.05=",format(round({mean(x<0.05/2| x>(1-0.05/2))},3), format="f", nsmall=3), symbols.p05(x),sep=""))),

p.0.01=( (paste(" p.01=",format(round({mean(x<0.01/2| x>(1-0.01/2))},3), format="f", nsmall=3), symbols.p01(x),sep=""))))},method="cross",g=4)

print(x,prn=F)

x<-summary(results.nspp.Poisson.Nrandom.theta1.sigma2.taurandomV1.Sc2.conjugate.R [,"kurt"]~mult.sigma+No, fun=function(x){c(

ks.D=( (paste(" ks.D=",format(round(ks.test(x,"punif")$s,3), format="f", nsmall=3), symbols.ks(x),sep=""))),

p.0.05=( (paste(" p.05=",format(round({mean(x<0.05/2| x>(1-0.05/2))},3), format="f", nsmall=3), symbols.p05(x),sep=""))),

p.0.01=( (paste(" p.01=",format(round({mean(x<0.01/2| x>(1-0.01/2))},3), format="f", nsmall=3), symbols.p01(x),sep=""))))},method="cross",g=4)

print(x,prn=F)

x<-summary(results.nspp.Poisson.Nrandom.theta1.sigma2.taurandomV1.Sc2.conjugate.R [,"Za"]~mult.sigma+No, fun=function(x){c(

ks.D=( (paste(" ks.D=",format(round(ks.test(x,"punif")$s,3), format="f", nsmall=3), symbols.ks(x),sep=""))),

p.0.05=( (paste(" p.05=",format(round({mean(x<0.05/2| x>(1-0.05/2))},3), format="f", nsmall=3), symbols.p05(x),sep=""))),

p.0.01=( (paste(" p.01=",format(round({mean(x<0.01/2| x>(1-0.01/2))},3), format="f", nsmall=3), symbols.p01(x),sep=""))))},method="cross",g=4)

print(x,prn=F)

#### #=>A2: Poisson, spp et dspp & dspp2, conjugate

**#R, spp, conjugate, sigma et N**

No<- results.spp.Poisson.Nrandom.theta1.sigma2.taurandomV1.Sc2.conjugate.R [,"N"]

mult.sigma<- round(results.spp.Poisson.Nrandom.theta1.sigma2.taurandomV1.Sc2.conjugate.R[,"musigma0"],digits=2)

**x<-summary(**results.spp.Poisson.Nrandom.theta1.sigma2.taurandomV1.Sc2.conjugate.R [,"mean"]~mult.sigma+No, fun=function(x){c(

ks.D=( (paste(" ks.D=",format(round(ks.test(x,"punif")$s,3), format="f", nsmall=3), symbols.ks(x),sep=""))),

p.0.05=( (paste(" p.05=",format(round({mean(x<0.05/2| x>(1-0.05/2))},3), format="f", nsmall=3), symbols.p05(x),sep=""))),

p.0.01=( (paste(" p.01=",format(round({mean(x<0.01/2| x>(1-0.01/2))},3), format="f", nsmall=3), symbols.p01(x),sep=""))))},method="cross",g=4)

print(x,prn=F)

x<-summary(results.spp.Poisson.Nrandom.theta1.sigma2.taurandomV1.Sc2.conjugate.R [,"var"]~mult.sigma+No, fun=function(x){c(

ks.D=( (paste(" ks.D=",format(round(ks.test(x,"punif")$s,3), format="f", nsmall=3), symbols.ks(x),sep=""))),

p.0.05=( (paste(" p.05=",format(round({mean(x<0.05/2| x>(1-0.05/2))},3), format="f", nsmall=3), symbols.p05(x),sep=""))),

p.0.01=( (paste(" p.01=",format(round({mean(x<0.01/2| x>(1-0.01/2))},3), format="f", nsmall=3), symbols.p01(x),sep=""))))},method="cross",g=4)

print(x,prn=F)

**#R, dspp, conjugate, sigma et N**

No<- results.dspp.Poisson.Nrandom.theta1.sigma2.taurandomV1.Sc2.conjugate.R [,"N"]

mult.sigma<- round(results.dspp.Poisson.Nrandom.theta1.sigma2.taurandomV1.Sc2.conjugate.R[,"musigma0"],digits=2)

**x<-summary(**results.dspp.Poisson.Nrandom.theta1.sigma2.taurandomV1.Sc2.conjugate.R [,"p0"]~mult.sigma+No, fun=function(x){c(

ks.D=( (paste(" ks.D=",format(round(ks.test(x,"punif")$s,3), format="f", nsmall=3), symbols.ks(x),sep=""))),

p.0.05=( (paste(" p.05=",format(round({mean(x<0.05/2| x>(1-0.05/2))},3), format="f", nsmall=3), symbols.p05(x),sep=""))),

p.0.01=( (paste(" p.01=",format(round({mean(x<0.01/2| x>(1-0.01/2))},3), format="f", nsmall=3), symbols.p01(x),sep=""))))},method="cross",g=4)

print(x,prn=F)

x<-summary(results.dspp.Poisson.Nrandom.theta1.sigma2.taurandomV1.Sc2.conjugate.R [,"p01"]~mult.sigma+No, fun=function(x){c(

ks.D=( (paste(" ks.D=",format(round(ks.test(x,"punif")$s,3), format="f", nsmall=3), symbols.ks(x),sep=""))),

p.0.05=( (paste(" p.05=",format(round({mean(x<0.05/2| x>(1-0.05/2))},3), format="f", nsmall=3), symbols.p05(x),sep=""))),

p.0.01=( (paste(" p.01=",format(round({mean(x<0.01/2| x>(1-0.01/2))},3), format="f", nsmall=3), symbols.p01(x),sep=""))))},method="cross",g=4)

print(x,prn=F)

**x<-summary(**results.dspp.Poisson.Nrandom.theta1.sigma2.taurandomV1.Sc2.conjugate.R [,"meanc"]~mult.sigma+No, fun=function(x){c(

ks.D=( (paste(" ks.D=",format(round(ks.test(x,"punif")$s,3), format="f", nsmall=3), symbols.ks(x),sep=""))),

p.0.05=( (paste(" p.05=",format(round({mean(x<0.05/2| x>(1-0.05/2))},3), format="f", nsmall=3), symbols.p05(x),sep=""))),

p.0.01=( (paste(" p.01=",format(round({mean(x<0.01/2| x>(1-0.01/2))},3), format="f", nsmall=3), symbols.p01(x),sep=""))))},method="cross",g=4)

print(x,prn=F)

x<-summary(results.dspp.Poisson.Nrandom.theta1.sigma2.taurandomV1.Sc2.conjugate.R [,"varc"]~mult.sigma+No, fun=function(x){c(

ks.D=( (paste(" ks.D=",format(round(ks.test(x,"punif")$s,3), format="f", nsmall=3), symbols.ks(x),sep=""))),

p.0.05=( (paste(" p.05=",format(round({mean(x<0.05/2| x>(1-0.05/2))},3), format="f", nsmall=3), symbols.p05(x),sep=""))),

p.0.01=( (paste(" p.01=",format(round({mean(x<0.01/2| x>(1-0.01/2))},3), format="f", nsmall=3), symbols.p01(x),sep=""))))},method="cross",g=4)

print(x,prn=F)

**#R, dspp2, conjugate, sigma et N**

No<- results.dspp2.Poisson.Nrandom.theta1.sigma2.taurandomV1.Sc2.conjugate.R [,"N"]

mult.sigma<- round(results.dspp2.Poisson.Nrandom.theta1.sigma2.taurandomV1.Sc2.conjugate.R[,"musigma0"],digits=2)

**x<-summary(**results.dspp2.Poisson.Nrandom.theta1.sigma2.taurandomV1.Sc2.conjugate.R [,"LL"]~mult.sigma+No, fun=function(x){c(

ks.D=( (paste(" ks.D=",format(round(ks.test(x,"punif")$s,3), format="f", nsmall=3), symbols.ks(x),sep=""))),

p.0.05=( (paste(" p.05=",format(round({mean(x<0.05/2| x>(1-0.05/2))},3), format="f", nsmall=3), symbols.p05(x),sep=""))),

p.0.01=( (paste(" p.01=",format(round({mean(x<0.01/2| x>(1-0.01/2))},3), format="f", nsmall=3), symbols.p01(x),sep=""))))},method="cross",g=4)

print(x,prn=F)

#### #=>A2: Normal, nspp, conjugate

**#R, nspp, conjugate, juste sigma (en texte masqué)**

No<- results.nspp.Normal.Nrandom.tausigmarandom2.Sc2.conjugate.R [,"N"]

mult.sigma<- round(results.nspp.Normal.Nrandom.tausigmarandom2.Sc2.conjugate.R[,"musigma0"],digits=1)

**x<-summary(**results.nspp.Normal.Nrandom.tausigmarandom2.Sc2.conjugate.R [,"mean"]~mult.sigma, fun=function(x){c(

ks.D=( (paste(" ks.D=",format(round(ks.test(x,"punif")$s,3), format="f", nsmall=3), symbols.ks(x),sep=""))),

p.0.05=( (paste(" p.05=",format(round({mean(x<0.05/2| x>(1-0.05/2))},3), format="f", nsmall=3), symbols.p05(x),sep=""))),

p.0.01=( (paste(" p.01=",format(round({mean(x<0.01/2| x>(1-0.01/2))},3), format="f", nsmall=3), symbols.p01(x),sep=""))))},method="cross",g=4)

print(x,prn=F)

x<-summary(results.nspp.Normal.Nrandom.tausigmarandom2.Sc2.conjugate.R [,"var"]~mult.sigma, fun=function(x){c(

ks.D=( (paste(" ks.D=",format(round(ks.test(x,"punif")$s,3), format="f", nsmall=3), symbols.ks(x),sep=""))),

p.0.05=( (paste(" p.05=",format(round({mean(x<0.05/2| x>(1-0.05/2))},3), format="f", nsmall=3), symbols.p05(x),sep=""))),

p.0.01=( (paste(" p.01=",format(round({mean(x<0.01/2| x>(1-0.01/2))},3), format="f", nsmall=3), symbols.p01(x),sep=""))))},method="cross",g=4)

print(x,prn=F)

x<-summary(results.nspp.Normal.Nrandom.tausigmarandom2.Sc2.conjugate.R [,"skew"]~ mult.sigma, fun=function(x){c(

ks.D=( (paste(" ks.D=",format(round(ks.test(x,"punif")$s,3), format="f", nsmall=3), symbols.ks(x),sep=""))),

p.0.05=( (paste(" p.05=",format(round({mean(x<0.05/2| x>(1-0.05/2))},3), format="f", nsmall=3), symbols.p05(x),sep=""))),

p.0.01=( (paste(" p.01=",format(round({mean(x<0.01/2| x>(1-0.01/2))},3), format="f", nsmall=3), symbols.p01(x),sep=""))))},method="cross",g=4)

print(x,prn=F)

x<-summary(results.nspp.Normal.Nrandom.tausigmarandom2.Sc2.conjugate.R [,"kurt"]~ mult.sigma, fun=function(x){c(

ks.D=( (paste(" ks.D=",format(round(ks.test(x,"punif")$s,3), format="f", nsmall=3), symbols.ks(x),sep=""))),

p.0.05=( (paste(" p.05=",format(round({mean(x<0.05/2| x>(1-0.05/2))},3), format="f", nsmall=3), symbols.p05(x),sep=""))),

p.0.01=( (paste(" p.01=",format(round({mean(x<0.01/2| x>(1-0.01/2))},3), format="f", nsmall=3), symbols.p01(x),sep=""))))},method="cross",g=4)

print(x,prn=F)

x<-summary(results.nspp.Normal.Nrandom.tausigmarandom2.Sc2.conjugate.R [,"Za"]~ mult.sigma, fun=function(x){c(

ks.D=( (paste(" ks.D=",format(round(ks.test(x,"punif")$s,3), format="f", nsmall=3), symbols.ks(x),sep=""))),

p.0.05=( (paste(" p.05=",format(round({mean(x<0.05/2| x>(1-0.05/2))},3), format="f", nsmall=3), symbols.p05(x),sep=""))),

p.0.01=( (paste(" p.01=",format(round({mean(x<0.01/2| x>(1-0.01/2))},3), format="f", nsmall=3), symbols.p01(x),sep=""))))},method="cross",g=4)

print(x,prn=F)

**#R, nspp, conjugate, sigma et N**

No<- results.nspp.Normal.Nrandom.tausigmarandom2.Sc2.conjugate.R [,"N"]

No<- results.nspp.Normal.Nrandom.tausigmarandom2.Sc2.conjugate.R [,"N"]

mult.sigma<- round(results.nspp.Normal.Nrandom.tausigmarandom2.Sc2.conjugate.R[,"musigma0"],digits=2)

**x<-summary(**results.nspp.Normal.Nrandom.tausigmarandom2.Sc2.conjugate.R [,"mean"]~mult.sigma+No, fun=function(x){c(

ks.D=( (paste(" ks.D=",format(round(ks.test(x,"punif")$s,3), format="f", nsmall=3), symbols.ks(x),sep=""))),

p.0.05=( (paste(" p.05=",format(round({mean(x<0.05/2| x>(1-0.05/2))},3), format="f", nsmall=3), symbols.p05(x),sep=""))),

p.0.01=( (paste(" p.01=",format(round({mean(x<0.01/2| x>(1-0.01/2))},3), format="f", nsmall=3), symbols.p01(x),sep=""))))},method="cross",g=4)

print(x,prn=F)

x<-summary(results.nspp.Normal.Nrandom.tausigmarandom2.Sc2.conjugate.R [,"var"]~mult.sigma+No, fun=function(x){c(

ks.D=( (paste(" ks.D=",format(round(ks.test(x,"punif")$s,3), format="f", nsmall=3), symbols.ks(x),sep=""))),

p.0.05=( (paste(" p.05=",format(round({mean(x<0.05/2| x>(1-0.05/2))},3), format="f", nsmall=3), symbols.p05(x),sep=""))),

p.0.01=( (paste(" p.01=",format(round({mean(x<0.01/2| x>(1-0.01/2))},3), format="f", nsmall=3), symbols.p01(x),sep=""))))},method="cross",g=4)

print(x,prn=F)

x<-summary(results.nspp.Normal.Nrandom.tausigmarandom2.Sc2.conjugate.R [,"skew"]~mult.sigma+No, fun=function(x){c(

ks.D=( (paste(" ks.D=",format(round(ks.test(x,"punif")$s,3), format="f", nsmall=3), symbols.ks(x),sep=""))),

p.0.05=( (paste(" p.05=",format(round({mean(x<0.05/2| x>(1-0.05/2))},3), format="f", nsmall=3), symbols.p05(x),sep=""))),

p.0.01=( (paste(" p.01=",format(round({mean(x<0.01/2| x>(1-0.01/2))},3), format="f", nsmall=3), symbols.p01(x),sep=""))))},method="cross",g=4)

print(x,prn=F)

x<-summary(results.nspp.Normal.Nrandom.tausigmarandom2.Sc2.conjugate.R [,"kurt"]~mult.sigma+No, fun=function(x){c(

ks.D=( (paste(" ks.D=",format(round(ks.test(x,"punif")$s,3), format="f", nsmall=3), symbols.ks(x),sep=""))),

p.0.05=( (paste(" p.05=",format(round({mean(x<0.05/2| x>(1-0.05/2))},3), format="f", nsmall=3), symbols.p05(x),sep=""))),

p.0.01=( (paste(" p.01=",format(round({mean(x<0.01/2| x>(1-0.01/2))},3), format="f", nsmall=3), symbols.p01(x),sep=""))))},method="cross",g=4)

print(x,prn=F)

x<-summary(results.nspp.Normal.Nrandom.tausigmarandom2.Sc2.conjugate.R [,"Za"]~mult.sigma+No, fun=function(x){c(

ks.D=( (paste(" ks.D=",format(round(ks.test(x,"punif")$s,3), format="f", nsmall=3), symbols.ks(x),sep=""))),

p.0.05=( (paste(" p.05=",format(round({mean(x<0.05/2| x>(1-0.05/2))},3), format="f", nsmall=3), symbols.p05(x),sep=""))),

p.0.01=( (paste(" p.01=",format(round({mean(x<0.01/2| x>(1-0.01/2))},3), format="f", nsmall=3), symbols.p01(x),sep=""))))},method="cross",g=4)

print(x,prn=F)

#### #=>A2: Normal, spp et dspp & dspp2, conjugate

**#R, spp, conjugate, sigma et N**

No<- results.spp.Normal.Nrandom.tausigmarandom2.Sc2.conjugate.R [,"N"]

mult.sigma<- round(results.spp.Normal.Nrandom.tausigmarandom2.Sc2.conjugate.R[,"musigma0"],digits=2)

**x<-summary(**results.spp.Normal.Nrandom.tausigmarandom2.Sc2.conjugate.R [,"mean"]~mult.sigma+No, fun=function(x){c(

ks.D=( (paste(" ks.D=",format(round(ks.test(x,"punif")$s,3), format="f", nsmall=3), symbols.ks(x),sep=""))),

p.0.05=( (paste(" p.05=",format(round({mean(x<0.05/2| x>(1-0.05/2))},3), format="f", nsmall=3), symbols.p05(x),sep=""))),

p.0.01=( (paste(" p.01=",format(round({mean(x<0.01/2| x>(1-0.01/2))},3), format="f", nsmall=3), symbols.p01(x),sep=""))))},method="cross",g=4)

print(x,prn=F)

x<-summary(results.spp.Normal.Nrandom.tausigmarandom2.Sc2.conjugate.R [,"var"]~mult.sigma+No, fun=function(x){c(

ks.D=( (paste(" ks.D=",format(round(ks.test(x,"punif")$s,3), format="f", nsmall=3), symbols.ks(x),sep=""))),

p.0.05=( (paste(" p.05=",format(round({mean(x<0.05/2| x>(1-0.05/2))},3), format="f", nsmall=3), symbols.p05(x),sep=""))),

p.0.01=( (paste(" p.01=",format(round({mean(x<0.01/2| x>(1-0.01/2))},3), format="f", nsmall=3), symbols.p01(x),sep=""))))},method="cross",g=4)

print(x,prn=F)

**#R, dspp, conjugate, sigma et N**

No<- results.dspp.Normal.Nrandom.tausigmarandom2.Sc2.conjugate.R [,"N"]

mult.sigma<- round(results.dspp.Normal.Nrandom.tausigmarandom2.Sc2.conjugate.R[,"musigma0"],digits=2)

**x<-summary(**results.dspp.Normal.Nrandom.tausigmarandom2.Sc2.conjugate.R [,"plt0"]~mult.sigma+No, fun=function(x){c(

ks.D=( (paste(" ks.D=",format(round(ks.test(x,"punif")$s,3), format="f", nsmall=3), symbols.ks(x),sep=""))),

p.0.05=( (paste(" p.05=",format(round({mean(x<0.05/2| x>(1-0.05/2))},3), format="f", nsmall=3), symbols.p05(x),sep=""))),

p.0.01=( (paste(" p.01=",format(round({mean(x<0.01/2| x>(1-0.01/2))},3), format="f", nsmall=3), symbols.p01(x),sep=""))))},method="cross",g=4)

print(x,prn=F)

x<-summary(results.dspp.Normal.Nrandom.tausigmarandom2.Sc2.conjugate.R [,"meanc"]~mult.sigma+No, fun=function(x){c(

ks.D=( (paste(" ks.D=",format(round(ks.test(x,"punif")$s,3), format="f", nsmall=3), symbols.ks(x),sep=""))),

p.0.05=( (paste(" p.05=",format(round({mean(x<0.05/2| x>(1-0.05/2))},3), format="f", nsmall=3), symbols.p05(x),sep=""))),

p.0.01=( (paste(" p.01=",format(round({mean(x<0.01/2| x>(1-0.01/2))},3), format="f", nsmall=3), symbols.p01(x),sep=""))))},method="cross",g=4)

print(x,prn=F)

x<-summary(results.dspp.Normal.Nrandom.tausigmarandom2.Sc2.conjugate.R [,"varc"]~mult.sigma+No, fun=function(x){c(

ks.D=( (paste(" ks.D=",format(round(ks.test(x,"punif")$s,3), format="f", nsmall=3), symbols.ks(x),sep=""))),

p.0.05=( (paste(" p.05=",format(round({mean(x<0.05/2| x>(1-0.05/2))},3), format="f", nsmall=3), symbols.p05(x),sep=""))),

p.0.01=( (paste(" p.01=",format(round({mean(x<0.01/2| x>(1-0.01/2))},3), format="f", nsmall=3), symbols.p01(x),sep=""))))},method="cross",g=4)

print(x,prn=F)

**#R, dspp2, conjugate, sigma et N**

No<- results.dspp2.Normal.Nrandom.tausigmarandom2.Sc2.conjugate.R [,"N"]

mult.sigma<- round(results.dspp2.Normal.Nrandom.tausigmarandom2.Sc2.conjugate.R[,"musigma0"],digits=2)

**x<-summary(**results.dspp2.Normal.Nrandom.tausigmarandom2.Sc2.conjugate.R [,"LL"]~mult.sigma+No, fun=function(x){c(

ks.D=( (paste(" ks.D=",format(round(ks.test(x,"punif")$s,3), format="f", nsmall=3), symbols.ks(x),sep=""))),

p.0.05=( (paste(" p.05=",format(round({mean(x<0.05/2| x>(1-0.05/2))},3), format="f", nsmall=3), symbols.p05(x),sep=""))),

p.0.01=( (paste(" p.01=",format(round({mean(x<0.01/2| x>(1-0.01/2))},3), format="f", nsmall=3), symbols.p01(x),sep=""))))},method="cross",g=4)

print(x,prn=F)

#### #=>A2: Bernoulli, nspp, conjugate

#important remark: musigma0 inverted due to inversion between ppaer and commands here.

**#R, nspp, conjugate, juste sigma (texte masqué)**

No<- results.nspp.Bernoulli.Nrandom.thetarandom5.Sc2.conjugate.R [,"N"]

mult.sigma<- round(1/results.nspp.Bernoulli.Nrandom.thetarandom5.Sc2.conjugate.R[,"musigma0"],digits=2)

**x<-summary(**results.nspp.Bernoulli.Nrandom.thetarandom5.Sc2.conjugate.R [,"mean"]~mult.sigma, fun=function(x){c(

ks.D=( (paste(" ks.D=",format(round(ks.test(x,"punif")$s,3), format="f", nsmall=3), symbols.ks(x),sep=""))),

p.0.05=( (paste(" p.05=",format(round({mean(x<0.05/2| x>(1-0.05/2))},3), format="f", nsmall=3), symbols.p05(x),sep=""))),

p.0.01=( (paste(" p.01=",format(round({mean(x<0.01/2| x>(1-0.01/2))},3), format="f", nsmall=3), symbols.p01(x),sep=""))))},method="cross",g=4)

print(x,prn=F)

x<-summary(results.nspp.Bernoulli.Nrandom.thetarandom5.Sc2.conjugate.R [,"var"]~mult.sigma, fun=function(x){c(

ks.D=( (paste(" ks.D=",format(round(ks.test(x,"punif")$s,3), format="f", nsmall=3), symbols.ks(x),sep=""))),

p.0.05=( (paste(" p.05=",format(round({mean(x<0.05/2| x>(1-0.05/2))},3), format="f", nsmall=3), symbols.p05(x),sep=""))),

p.0.01=( (paste(" p.01=",format(round({mean(x<0.01/2| x>(1-0.01/2))},3), format="f", nsmall=3), symbols.p01(x),sep=""))))},method="cross",g=4)

print(x,prn=F)

x<-summary(results.nspp.Bernoulli.Nrandom.thetarandom5.Sc2.conjugate.R [,"skew"]~ mult.sigma, fun=function(x){c(

ks.D=( (paste(" ks.D=",format(round(ks.test(x,"punif")$s,3), format="f", nsmall=3), symbols.ks(x),sep=""))),

p.0.05=( (paste(" p.05=",format(round({mean(x<0.05/2| x>(1-0.05/2))},3), format="f", nsmall=3), symbols.p05(x),sep=""))),

p.0.01=( (paste(" p.01=",format(round({mean(x<0.01/2| x>(1-0.01/2))},3), format="f", nsmall=3), symbols.p01(x),sep=""))))},method="cross",g=4)

print(x,prn=F)

x<-summary(results.nspp.Bernoulli.Nrandom.thetarandom5.Sc2.conjugate.R [,"kurt"]~ mult.sigma, fun=function(x){c(

ks.D=( (paste(" ks.D=",format(round(ks.test(x,"punif")$s,3), format="f", nsmall=3), symbols.ks(x),sep=""))),

p.0.05=( (paste(" p.05=",format(round({mean(x<0.05/2| x>(1-0.05/2))},3), format="f", nsmall=3), symbols.p05(x),sep=""))),

p.0.01=( (paste(" p.01=",format(round({mean(x<0.01/2| x>(1-0.01/2))},3), format="f", nsmall=3), symbols.p01(x),sep=""))))},method="cross",g=4)

print(x,prn=F)

x<-summary(results.nspp.Bernoulli.Nrandom.thetarandom5.Sc2.conjugate.R [,"Za"]~ mult.sigma, fun=function(x){c(

ks.D=( (paste(" ks.D=",format(round(ks.test(x,"punif")$s,3), format="f", nsmall=3), symbols.ks(x),sep=""))),

p.0.05=( (paste(" p.05=",format(round({mean(x<0.05/2| x>(1-0.05/2))},3), format="f", nsmall=3), symbols.p05(x),sep=""))),

p.0.01=( (paste(" p.01=",format(round({mean(x<0.01/2| x>(1-0.01/2))},3), format="f", nsmall=3), symbols.p01(x),sep=""))))},method="cross",g=4)

print(x,prn=F)

**#R, nspp, conjugate, sigma et N**

No<- results.nspp.Bernoulli.Nrandom.thetarandom5.Sc2.conjugate.R [,"N"]

mult.sigma<- round(1/results.nspp.Bernoulli.Nrandom.thetarandom5.Sc2.conjugate.R[,"musigma0"],digits=2)

**x<-summary(**results.nspp.Bernoulli.Nrandom.thetarandom5.Sc2.conjugate.R [,"mean"]~mult.sigma+No, fun=function(x){c(

ks.D=( (paste(" ks.D=",format(round(ks.test(x,"punif")$s,3), format="f", nsmall=3), symbols.ks(x),sep=""))),

p.0.05=( (paste(" p.05=",format(round({mean(x<0.05/2| x>(1-0.05/2))},3), format="f", nsmall=3), symbols.p05(x),sep=""))),

p.0.01=( (paste(" p.01=",format(round({mean(x<0.01/2| x>(1-0.01/2))},3), format="f", nsmall=3), symbols.p01(x),sep=""))))},method="cross",g=4)

print(x,prn=F)

x<-summary(results.nspp.Bernoulli.Nrandom.thetarandom5.Sc2.conjugate.R [,"var"]~mult.sigma+No, fun=function(x){c(

ks.D=( (paste(" ks.D=",format(round(ks.test(x,"punif")$s,3), format="f", nsmall=3), symbols.ks(x),sep=""))),

p.0.05=( (paste(" p.05=",format(round({mean(x<0.05/2| x>(1-0.05/2))},3), format="f", nsmall=3), symbols.p05(x),sep=""))),

p.0.01=( (paste(" p.01=",format(round({mean(x<0.01/2| x>(1-0.01/2))},3), format="f", nsmall=3), symbols.p01(x),sep=""))))},method="cross",g=4)

print(x,prn=F)

x<-summary(results.nspp.Bernoulli.Nrandom.thetarandom5.Sc2.conjugate.R [,"skew"]~mult.sigma+No, fun=function(x){c(

ks.D=( (paste(" ks.D=",format(round(ks.test(x,"punif")$s,3), format="f", nsmall=3), symbols.ks(x),sep=""))),

p.0.05=( (paste(" p.05=",format(round({mean(x<0.05/2| x>(1-0.05/2))},3), format="f", nsmall=3), symbols.p05(x),sep=""))),

p.0.01=( (paste(" p.01=",format(round({mean(x<0.01/2| x>(1-0.01/2))},3), format="f", nsmall=3), symbols.p01(x),sep=""))))},method="cross",g=4)

print(x,prn=F)

x<-summary(results.nspp.Bernoulli.Nrandom.thetarandom5.Sc2.conjugate.R [,"kurt"]~mult.sigma+No, fun=function(x){c(

ks.D=( (paste(" ks.D=",format(round(ks.test(x,"punif")$s,3), format="f", nsmall=3), symbols.ks(x),sep=""))),

p.0.05=( (paste(" p.05=",format(round({mean(x<0.05/2| x>(1-0.05/2))},3), format="f", nsmall=3), symbols.p05(x),sep=""))),

p.0.01=( (paste(" p.01=",format(round({mean(x<0.01/2| x>(1-0.01/2))},3), format="f", nsmall=3), symbols.p01(x),sep=""))))},method="cross",g=4)

print(x,prn=F)

x<-summary(results.nspp.Bernoulli.Nrandom.thetarandom5.Sc2.conjugate.R [,"Za"]~mult.sigma+No, fun=function(x){c(

ks.D=( (paste(" ks.D=",format(round(ks.test(x,"punif")$s,3), format="f", nsmall=3), symbols.ks(x),sep=""))),

p.0.05=( (paste(" p.05=",format(round({mean(x<0.05/2| x>(1-0.05/2))},3), format="f", nsmall=3), symbols.p05(x),sep=""))),

p.0.01=( (paste(" p.01=",format(round({mean(x<0.01/2| x>(1-0.01/2))},3), format="f", nsmall=3), symbols.p01(x),sep=""))))},method="cross",g=4)

print(x,prn=F)

#### #=>A2: Bernoulli, spp et dspp & dspp2, conjugate

#important remark: musigma0 inverted due to inversion between ppaer and commands here.

**#R, spp, conjugate, sigma et N**

No<- results.spp.Bernoulli.Nrandom.thetarandom5.Sc2.conjugate.R [,"N"]

mult.sigma<- round(1/results.spp.Bernoulli.Nrandom.thetarandom5.Sc2.conjugate.R[,"musigma0"],digits=2)

**x<-summary(**results.spp.Bernoulli.Nrandom.thetarandom5.Sc2.conjugate.R [,"mean"]~mult.sigma+No, fun=function(x){c(

ks.D=( (paste(" ks.D=",format(round(ks.test(x,"punif")$s,3), format="f", nsmall=3), symbols.ks(x),sep=""))),

p.0.05=( (paste(" p.05=",format(round({mean(x<0.05/2| x>(1-0.05/2))},3), format="f", nsmall=3), symbols.p05(x),sep=""))),

p.0.01=( (paste(" p.01=",format(round({mean(x<0.01/2| x>(1-0.01/2))},3), format="f", nsmall=3), symbols.p01(x),sep=""))))},method="cross",g=4)

print(x,prn=F)

x<-summary(results.spp.Bernoulli.Nrandom.thetarandom5.Sc2.conjugate.R [,"var"]~mult.sigma+No, fun=function(x){c(

ks.D=( (paste(" ks.D=",format(round(ks.test(x,"punif")$s,3), format="f", nsmall=3), symbols.ks(x),sep=""))),

p.0.05=( (paste(" p.05=",format(round({mean(x<0.05/2| x>(1-0.05/2))},3), format="f", nsmall=3), symbols.p05(x),sep=""))),

p.0.01=( (paste(" p.01=",format(round({mean(x<0.01/2| x>(1-0.01/2))},3), format="f", nsmall=3), symbols.p01(x),sep=""))))},method="cross",g=4)

print(x,prn=F)

**#R, dspp, conjugate, sigma et N**

No<- results.dspp.Bernoulli.Nrandom.thetarandom5.Sc2.conjugate.R [,"N"]

mult.sigma<- round(1/results.dspp.Bernoulli.Nrandom.thetarandom5.Sc2.conjugate.R[,"musigma0"],digits=2)

**x<-summary(**results.dspp.Bernoulli.Nrandom.thetarandom5.Sc2.conjugate.R [,"p0"]~mult.sigma+No, fun=function(x){c(

ks.D=( (paste(" ks.D=",format(round(ks.test(x,"punif")$s,3), format="f", nsmall=3), symbols.ks(x),sep=""))),

p.0.05=( (paste(" p.05=",format(round({mean(x<0.05/2| x>(1-0.05/2))},3), format="f", nsmall=3), symbols.p05(x),sep=""))),

p.0.01=( (paste(" p.01=",format(round({mean(x<0.01/2| x>(1-0.01/2))},3), format="f", nsmall=3), symbols.p01(x),sep=""))))},method="cross",g=4)

print(x,prn=F)

x<-summary(results.dspp.Bernoulli.Nrandom.thetarandom5.Sc2.conjugate.R [,"p01"]~mult.sigma+No, fun=function(x){c(

ks.D=( (paste(" ks.D=",format(round(ks.test(x,"punif")$s,3), format="f", nsmall=3), symbols.ks(x),sep=""))),

p.0.05=( (paste(" p.05=",format(round({mean(x<0.05/2| x>(1-0.05/2))},3), format="f", nsmall=3), symbols.p05(x),sep=""))),

p.0.01=( (paste(" p.01=",format(round({mean(x<0.01/2| x>(1-0.01/2))},3), format="f", nsmall=3), symbols.p01(x),sep=""))))},method="cross",g=4)

print(x,prn=F)

x<-summary(results.dspp.Bernoulli.Nrandom.thetarandom5.Sc2.conjugate.R [,"meanc"]~mult.sigma+No, fun=function(x){c(

ks.D=( (paste(" ks.D=",format(round(ks.test(x,"punif")$s,3), format="f", nsmall=3), symbols.ks(x),sep=""))),

p.0.05=( (paste(" p.05=",format(round({mean(x<0.05/2| x>(1-0.05/2))},3), format="f", nsmall=3), symbols.p05(x),sep=""))),

p.0.01=( (paste(" p.01=",format(round({mean(x<0.01/2| x>(1-0.01/2))},3), format="f", nsmall=3), symbols.p01(x),sep=""))))},method="cross",g=4)

print(x,prn=F)

x<-summary(results.dspp.Bernoulli.Nrandom.thetarandom5.Sc2.conjugate.R [,"varc"]~mult.sigma+No, fun=function(x){c(

ks.D=( (paste(" ks.D=",format(round(ks.test(x,"punif")$s,3), format="f", nsmall=3), symbols.ks(x),sep=""))),

p.0.05=( (paste(" p.05=",format(round({mean(x<0.05/2| x>(1-0.05/2))},3), format="f", nsmall=3), symbols.p05(x),sep=""))),

p.0.01=( (paste(" p.01=",format(round({mean(x<0.01/2| x>(1-0.01/2))},3), format="f", nsmall=3), symbols.p01(x),sep=""))))},method="cross",g=4)

print(x,prn=F)

**#R, dspp2, conjugate, sigma et N**

No<- results.dspp2.Bernoulli.Nrandom.thetarandom5.Sc2.conjugate.R [,"N"]

mult.sigma<- round(1/results.dspp2.Bernoulli.Nrandom.thetarandom5.Sc2.conjugate.R[,"musigma0"],digits=2)

**x<-summary(**results.dspp2.Bernoulli.Nrandom.thetarandom5.Sc2.conjugate.R [,"LL"]~mult.sigma+No, fun=function(x){c(

ks.D=( (paste(" ks.D=",format(round(ks.test(x,"punif")$s,3), format="f", nsmall=3), symbols.ks(x),sep=""))),

p.0.05=( (paste(" p.05=",format(round({mean(x<0.05/2| x>(1-0.05/2))},3), format="f", nsmall=3), symbols.p05(x),sep=""))),

p.0.01=( (paste(" p.01=",format(round({mean(x<0.01/2| x>(1-0.01/2))},3), format="f", nsmall=3), symbols.p01(x),sep=""))))},method="cross",g=4)

print(x,prn=F)

## Tables for Text S3 (Scenario 3 results)

#### #=>A3: Poisson, nspp, conjugate

#commentaire: résultats peuvent paraître surpenants par rapport à Normal/Bernoulli (notamment croissance departures fréquentiste avec valeur theta0): mais il n'en est rien: c'est normal: cf. forme du posterior où c'est theat0/sigma0 et theta0^2/sigma0 qui jouent...

#à sigma0 constant, plut theta0 est petit moins il inlfue le posterior

#à theta0 constant, plus sigma0 est grand, moins il compte dans le posterior

# (sous entendu à y et J constants)

**#R, nspp, conjugate, sigma et mu**

No<- results.nspp.Poisson.Nrandom.theta1.sigma2.taurandomV1.Sc3.conjugate.R [,"N"]

mult.sigma<- round(results.nspp.Poisson.Nrandom.theta1.sigma2.taurandomV1.Sc3.conjugate.R[,"musigma0"],digits=2)

theta0<- round(abs(log(results.nspp.Poisson.Nrandom.theta1.sigma2.taurandomV1.Sc3.conjugate.R[,"theta0"])-1),digits=2)

**x<-summary(**results.nspp.Poisson.Nrandom.theta1.sigma2.taurandomV1.Sc3.conjugate.R [,"mean"]~mult.sigma+theta0, fun=function(x){c(

ks.D=( (paste(" ks.D=",format(round(ks.test(x,"punif")$s,3), format="f", nsmall=3), symbols.ks(x),sep=""))),

p.0.05=( (paste(" p.05=",format(round({mean(x<0.05/2| x>(1-0.05/2))},3), format="f", nsmall=3), symbols.p05(x),sep=""))),

p.0.01=( (paste(" p.01=",format(round({mean(x<0.01/2| x>(1-0.01/2))},3), format="f", nsmall=3), symbols.p01(x),sep=""))))},method="cross",g=4)

print(x,prn=F)

x<-summary(results.nspp.Poisson.Nrandom.theta1.sigma2.taurandomV1.Sc3.conjugate.R [,"var"]~mult.sigma+theta0, fun=function(x){c(

ks.D=( (paste(" ks.D=",format(round(ks.test(x,"punif")$s,3), format="f", nsmall=3), symbols.ks(x),sep=""))),

p.0.05=( (paste(" p.05=",format(round({mean(x<0.05/2| x>(1-0.05/2))},3), format="f", nsmall=3), symbols.p05(x),sep=""))),

p.0.01=( (paste(" p.01=",format(round({mean(x<0.01/2| x>(1-0.01/2))},3), format="f", nsmall=3), symbols.p01(x),sep=""))))},method="cross",g=4)

print(x,prn=F)

x<-summary(results.nspp.Poisson.Nrandom.theta1.sigma2.taurandomV1.Sc3.conjugate.R [,"skew"]~mult.sigma+theta0, fun=function(x){c(

ks.D=( (paste(" ks.D=",format(round(ks.test(x,"punif")$s,3), format="f", nsmall=3), symbols.ks(x),sep=""))),

p.0.05=( (paste(" p.05=",format(round({mean(x<0.05/2| x>(1-0.05/2))},3), format="f", nsmall=3), symbols.p05(x),sep=""))),

p.0.01=( (paste(" p.01=",format(round({mean(x<0.01/2| x>(1-0.01/2))},3), format="f", nsmall=3), symbols.p01(x),sep=""))))},method="cross",g=4)

print(x,prn=F)

x<-summary(results.nspp.Poisson.Nrandom.theta1.sigma2.taurandomV1.Sc3.conjugate.R [,"kurt"]~mult.sigma+theta0, fun=function(x){c(

ks.D=( (paste(" ks.D=",format(round(ks.test(x,"punif")$s,3), format="f", nsmall=3), symbols.ks(x),sep=""))),

p.0.05=( (paste(" p.05=",format(round({mean(x<0.05/2| x>(1-0.05/2))},3), format="f", nsmall=3), symbols.p05(x),sep=""))),

p.0.01=( (paste(" p.01=",format(round({mean(x<0.01/2| x>(1-0.01/2))},3), format="f", nsmall=3), symbols.p01(x),sep=""))))},method="cross",g=4)

print(x,prn=F)

x<-summary(results.nspp.Poisson.Nrandom.theta1.sigma2.taurandomV1.Sc3.conjugate.R [,"Za"]~mult.sigma+theta0, fun=function(x){c(

ks.D=( (paste(" ks.D=",format(round(ks.test(x,"punif")$s,3), format="f", nsmall=3), symbols.ks(x),sep=""))),

p.0.05=( (paste(" p.05=",format(round({mean(x<0.05/2| x>(1-0.05/2))},3), format="f", nsmall=3), symbols.p05(x),sep=""))),

p.0.01=( (paste(" p.01=",format(round({mean(x<0.01/2| x>(1-0.01/2))},3), format="f", nsmall=3), symbols.p01(x),sep=""))))},method="cross",g=4)

print(x,prn=F)

**#(((R, nspp, conjugate, sigma et mu scaled)))**

No<- results.nspp.Poisson.Nrandom.theta1.sigma2.taurandomV1.Sc3.conjugate.R [,"N"]

mult.sigma<- round(results.nspp.Poisson.Nrandom.theta1.sigma2.taurandomV1.Sc3.conjugate.R[,"musigma0"],digits=2)

theta0<- round(abs(log(results.nspp.Poisson.Nrandom.theta1.sigma2.taurandomV1.Sc3.conjugate.R[,"theta0"])-1)/ results.nspp.Poisson.Nrandom.theta1.sigma2.taurandomV1.Sc3.conjugate.R[,"musigma0"],digits=2)

**x<-summary(**results.nspp.Poisson.Nrandom.theta1.sigma2.taurandomV1.Sc3.conjugate.R [,"mean"]~mult.sigma+theta0, fun=function(x){c(

ks.D=( (paste(" ks.D=",format(round(ks.test(x,"punif")$s,3), format="f", nsmall=3), symbols.ks(x),sep=""))),

p.0.05=( (paste(" p.05=",format(round({mean(x<0.05/2| x>(1-0.05/2))},3), format="f", nsmall=3), symbols.p05(x),sep=""))),

p.0.01=( (paste(" p.01=",format(round({mean(x<0.01/2| x>(1-0.01/2))},3), format="f", nsmall=3), symbols.p01(x),sep=""))))},method="cross",g=4)

print(x,prn=F)

x<-summary(results.nspp.Poisson.Nrandom.theta1.sigma2.taurandomV1.Sc3.conjugate.R [,"var"]~mult.sigma+theta0, fun=function(x){c(

ks.D=( (paste(" ks.D=",format(round(ks.test(x,"punif")$s,3), format="f", nsmall=3), symbols.ks(x),sep=""))),

p.0.05=( (paste(" p.05=",format(round({mean(x<0.05/2| x>(1-0.05/2))},3), format="f", nsmall=3), symbols.p05(x),sep=""))),

p.0.01=( (paste(" p.01=",format(round({mean(x<0.01/2| x>(1-0.01/2))},3), format="f", nsmall=3), symbols.p01(x),sep=""))))},method="cross",g=4)

print(x,prn=F)

x<-summary(results.nspp.Poisson.Nrandom.theta1.sigma2.taurandomV1.Sc3.conjugate.R [,"skew"]~mult.sigma+theta0, fun=function(x){c(

ks.D=( (paste(" ks.D=",format(round(ks.test(x,"punif")$s,3), format="f", nsmall=3), symbols.ks(x),sep=""))),

p.0.05=( (paste(" p.05=",format(round({mean(x<0.05/2| x>(1-0.05/2))},3), format="f", nsmall=3), symbols.p05(x),sep=""))),

p.0.01=( (paste(" p.01=",format(round({mean(x<0.01/2| x>(1-0.01/2))},3), format="f", nsmall=3), symbols.p01(x),sep=""))))},method="cross",g=4)

print(x,prn=F)

x<-summary(results.nspp.Poisson.Nrandom.theta1.sigma2.taurandomV1.Sc3.conjugate.R [,"kurt"]~mult.sigma+theta0, fun=function(x){c(

ks.D=( (paste(" ks.D=",format(round(ks.test(x,"punif")$s,3), format="f", nsmall=3), symbols.ks(x),sep=""))),

p.0.05=( (paste(" p.05=",format(round({mean(x<0.05/2| x>(1-0.05/2))},3), format="f", nsmall=3), symbols.p05(x),sep=""))),

p.0.01=( (paste(" p.01=",format(round({mean(x<0.01/2| x>(1-0.01/2))},3), format="f", nsmall=3), symbols.p01(x),sep=""))))},method="cross",g=4)

print(x,prn=F)

x<-summary(results.nspp.Poisson.Nrandom.theta1.sigma2.taurandomV1.Sc3.conjugate.R [,"Za"]~mult.sigma+theta0, fun=function(x){c(

ks.D=( (paste(" ks.D=",format(round(ks.test(x,"punif")$s,3), format="f", nsmall=3), symbols.ks(x),sep=""))),

p.0.05=( (paste(" p.05=",format(round({mean(x<0.05/2| x>(1-0.05/2))},3), format="f", nsmall=3), symbols.p05(x),sep=""))),

p.0.01=( (paste(" p.01=",format(round({mean(x<0.01/2| x>(1-0.01/2))},3), format="f", nsmall=3), symbols.p01(x),sep=""))))},method="cross",g=4)

print(x,prn=F)

**#R, nspp, conjugate, No et mu**

**#étonnant ici par rapport à Normal – Diags3: moins discriminant que mult.sigma+theta0**

No<- results.nspp.Poisson.Nrandom.theta1.sigma2.taurandomV1.Sc3.conjugate.R [,"N"]

mult.sigma<- round(results.nspp.Poisson.Nrandom.theta1.sigma2.taurandomV1.Sc3.conjugate.R[,"musigma0"],digits=2)

theta0<- round(abs(log(results.nspp.Poisson.Nrandom.theta1.sigma2.taurandomV1.Sc3.conjugate.R[,"theta0"])-1),digits=2)

**x<-summary(**results.nspp.Poisson.Nrandom.theta1.sigma2.taurandomV1.Sc3.conjugate.R [,"mean"]~No+theta0, fun=function(x){c(

ks.D=( (paste(" ks.D=",format(round(ks.test(x,"punif")$s,3), format="f", nsmall=3), symbols.ks(x),sep=""))),

p.0.05=( (paste(" p.05=",format(round({mean(x<0.05/2| x>(1-0.05/2))},3), format="f", nsmall=3), symbols.p05(x),sep=""))),

p.0.01=( (paste(" p.01=",format(round({mean(x<0.01/2| x>(1-0.01/2))},3), format="f", nsmall=3), symbols.p01(x),sep=""))))},method="cross",g=4)

print(x,prn=F)

x<-summary(results.nspp.Poisson.Nrandom.theta1.sigma2.taurandomV1.Sc3.conjugate.R [,"var"]~No+theta0, fun=function(x){c(

ks.D=( (paste(" ks.D=",format(round(ks.test(x,"punif")$s,3), format="f", nsmall=3), symbols.ks(x),sep=""))),

p.0.05=( (paste(" p.05=",format(round({mean(x<0.05/2| x>(1-0.05/2))},3), format="f", nsmall=3), symbols.p05(x),sep=""))),

p.0.01=( (paste(" p.01=",format(round({mean(x<0.01/2| x>(1-0.01/2))},3), format="f", nsmall=3), symbols.p01(x),sep=""))))},method="cross",g=4)

print(x,prn=F)

x<-summary(results.nspp.Poisson.Nrandom.theta1.sigma2.taurandomV1.Sc3.conjugate.R [,"skew"]~No+theta0, fun=function(x){c(

ks.D=( (paste(" ks.D=",format(round(ks.test(x,"punif")$s,3), format="f", nsmall=3), symbols.ks(x),sep=""))),

p.0.05=( (paste(" p.05=",format(round({mean(x<0.05/2| x>(1-0.05/2))},3), format="f", nsmall=3), symbols.p05(x),sep=""))),

p.0.01=( (paste(" p.01=",format(round({mean(x<0.01/2| x>(1-0.01/2))},3), format="f", nsmall=3), symbols.p01(x),sep=""))))},method="cross",g=4)

print(x,prn=F)

x<-summary(results.nspp.Poisson.Nrandom.theta1.sigma2.taurandomV1.Sc3.conjugate.R [,"kurt"]~No+theta0, fun=function(x){c(

ks.D=( (paste(" ks.D=",format(round(ks.test(x,"punif")$s,3), format="f", nsmall=3), symbols.ks(x),sep=""))),

p.0.05=( (paste(" p.05=",format(round({mean(x<0.05/2| x>(1-0.05/2))},3), format="f", nsmall=3), symbols.p05(x),sep=""))),

p.0.01=( (paste(" p.01=",format(round({mean(x<0.01/2| x>(1-0.01/2))},3), format="f", nsmall=3), symbols.p01(x),sep=""))))},method="cross",g=4)

print(x,prn=F)

x<-summary(results.nspp.Poisson.Nrandom.theta1.sigma2.taurandomV1.Sc3.conjugate.R [,"Za"]~No+theta0, fun=function(x){c(

ks.D=( (paste(" ks.D=",format(round(ks.test(x,"punif")$s,3), format="f", nsmall=3), symbols.ks(x),sep=""))),

p.0.05=( (paste(" p.05=",format(round({mean(x<0.05/2| x>(1-0.05/2))},3), format="f", nsmall=3), symbols.p05(x),sep=""))),

p.0.01=( (paste(" p.01=",format(round({mean(x<0.01/2| x>(1-0.01/2))},3), format="f", nsmall=3), symbols.p01(x),sep=""))))},method="cross",g=4)

print(x,prn=F)

#### #=>A3: Poisson, spp & dspp & dspp2, conjugate

**#R, spp, conjugate, sigma et mu**

No<- results.spp.Poisson.Nrandom.theta1.sigma2.taurandomV1.Sc3.conjugate.R [,"N"]

mult.sigma<- round(results.spp.Poisson.Nrandom.theta1.sigma2.taurandomV1.Sc3.conjugate.R[,"musigma0"],digits=2)

theta0<- round(abs(log(results.spp.Poisson.Nrandom.theta1.sigma2.taurandomV1.Sc3.conjugate.R[,"theta0"])-1),digits=2)

**x<-summary(**results.spp.Poisson.Nrandom.theta1.sigma2.taurandomV1.Sc3.conjugate.R [,"mean"]~mult.sigma+theta0, fun=function(x){c(

ks.D=( (paste(" ks.D=",format(round(ks.test(x,"punif")$s,3), format="f", nsmall=3), symbols.ks(x),sep=""))),

p.0.05=( (paste(" p.05=",format(round({mean(x<0.05/2| x>(1-0.05/2))},3), format="f", nsmall=3), symbols.p05(x),sep=""))),

p.0.01=( (paste(" p.01=",format(round({mean(x<0.01/2| x>(1-0.01/2))},3), format="f", nsmall=3), symbols.p01(x),sep=""))))},method="cross",g=4)

print(x,prn=F)

x<-summary(results.spp.Poisson.Nrandom.theta1.sigma2.taurandomV1.Sc3.conjugate.R [,"var"]~mult.sigma+theta0, fun=function(x){c(

ks.D=( (paste(" ks.D=",format(round(ks.test(x,"punif")$s,3), format="f", nsmall=3), symbols.ks(x),sep=""))),

p.0.05=( (paste(" p.05=",format(round({mean(x<0.05/2| x>(1-0.05/2))},3), format="f", nsmall=3), symbols.p05(x),sep=""))),

p.0.01=( (paste(" p.01=",format(round({mean(x<0.01/2| x>(1-0.01/2))},3), format="f", nsmall=3), symbols.p01(x),sep=""))))},method="cross",g=4)

print(x,prn=F)

**#R, dspp, conjugate, sigma et mu**

No<- results.dspp.Poisson.Nrandom.theta1.sigma2.taurandomV1.Sc3.conjugate.R [,"N"]

mult.sigma<- round(results.dspp.Poisson.Nrandom.theta1.sigma2.taurandomV1.Sc3.conjugate.R[,"musigma0"],digits=2)

theta0<- round(abs(log(results.dspp.Poisson.Nrandom.theta1.sigma2.taurandomV1.Sc3.conjugate.R[,"theta0"])-1),digits=2)

**x<-summary(**results.dspp.Poisson.Nrandom.theta1.sigma2.taurandomV1.Sc3.conjugate.R [,"p0"]~mult.sigma+theta0, fun=function(x){c(

ks.D=( (paste(" ks.D=",format(round(ks.test(x,"punif")$s,3), format="f", nsmall=3), symbols.ks(x),sep=""))),

p.0.05=( (paste(" p.05=",format(round({mean(x<0.05/2| x>(1-0.05/2))},3), format="f", nsmall=3), symbols.p05(x),sep=""))),

p.0.01=( (paste(" p.01=",format(round({mean(x<0.01/2| x>(1-0.01/2))},3), format="f", nsmall=3), symbols.p01(x),sep=""))))},method="cross",g=4)

print(x,prn=F)

**x<-summary(**results.dspp.Poisson.Nrandom.theta1.sigma2.taurandomV1.Sc3.conjugate.R [,"meanc"]~mult.sigma+theta0, fun=function(x){c(

ks.D=( (paste(" ks.D=",format(round(ks.test(x,"punif")$s,3), format="f", nsmall=3), symbols.ks(x),sep=""))),

p.0.05=( (paste(" p.05=",format(round({mean(x<0.05/2| x>(1-0.05/2))},3), format="f", nsmall=3), symbols.p05(x),sep=""))),

p.0.01=( (paste(" p.01=",format(round({mean(x<0.01/2| x>(1-0.01/2))},3), format="f", nsmall=3), symbols.p01(x),sep=""))))},method="cross",g=4)

print(x,prn=F)

x<-summary(results.dspp.Poisson.Nrandom.theta1.sigma2.taurandomV1.Sc3.conjugate.R [,"varc"]~mult.sigma+theta0, fun=function(x){c(

ks.D=( (paste(" ks.D=",format(round(ks.test(x,"punif")$s,3), format="f", nsmall=3), symbols.ks(x),sep=""))),

p.0.05=( (paste(" p.05=",format(round({mean(x<0.05/2| x>(1-0.05/2))},3), format="f", nsmall=3), symbols.p05(x),sep=""))),

p.0.01=( (paste(" p.01=",format(round({mean(x<0.01/2| x>(1-0.01/2))},3), format="f", nsmall=3), symbols.p01(x),sep=""))))},method="cross",g=4)

print(x,prn=F)

**#R, dspp2, conjugate, sigma et mu**

No<- results.dspp2.Poisson.Nrandom.theta1.sigma2.taurandomV1.Sc3.conjugate.R [,"N"]

mult.sigma<- round(results.dspp2.Poisson.Nrandom.theta1.sigma2.taurandomV1.Sc3.conjugate.R[,"musigma0"],digits=2)

theta0<- round(abs(log(results.dspp2.Poisson.Nrandom.theta1.sigma2.taurandomV1.Sc3.conjugate.R[,"theta0"])-1),digits=2)

**x<-summary(**results.dspp2.Poisson.Nrandom.theta1.sigma2.taurandomV1.Sc3.conjugate.R [,"LL"]~mult.sigma+theta0, fun=function(x){c(

ks.D=( (paste(" ks.D=",format(round(ks.test(x,"punif")$s,3), format="f", nsmall=3), symbols.ks(x),sep=""))),

p.0.05=( (paste(" p.05=",format(round({mean(x<0.05/2| x>(1-0.05/2))},3), format="f", nsmall=3), symbols.p05(x),sep=""))),

p.0.01=( (paste(" p.01=",format(round({mean(x<0.01/2| x>(1-0.01/2))},3), format="f", nsmall=3), symbols.p01(x),sep=""))))},method="cross",g=4)

print(x,prn=F)

#### #=>A3: Normal, nspp, conjugate

**#R, nspp, conjugate, sigma et mu**

No<- results.nspp.Normal.Nrandom.tausigmarandom2.Sc3.conjugate.R [,"N"]

#mult.sigma<- cut2(round(results.nspp.Normal.Nrandom.tausigmarandom2.Sc3.conjugate.R[,"musigma0"],digits=2), c(1,2,4,6,10))

mult.sigma<- cut2(round(results.nspp.Normal.Nrandom.tausigmarandom2.Sc3.conjugate.R[,"musigma0"],digits=2), g=4)

#mult.sigma<- round(results.nspp.Normal.Nrandom.tausigmarandom2.Sc3.conjugate.R[,"musigma0"],digits=2)

#theta0<- round((results.nspp.Normal.Nrandom.tausigmarandom2.Sc3.conjugate.R[,"theta0"]),digits=1)

theta0<- round(abs(results.nspp.Normal.Nrandom.tausigmarandom2.Sc3.conjugate.R[,"theta0"]),digits=1)

**x<-summary(**results.nspp.Normal.Nrandom.tausigmarandom2.Sc3.conjugate.R [,"mean"]~mult.sigma+theta0, fun=function(x){c(

ks.D=( (paste(" ks.D=",format(round(ks.test(x,"punif")$s,3), format="f", nsmall=3), symbols.ks(x),sep=""))),

p.0.05=( (paste(" p.05=",format(round({mean(x<0.05/2| x>(1-0.05/2))},3), format="f", nsmall=3), symbols.p05(x),sep=""))),

p.0.01=( (paste(" p.01=",format(round({mean(x<0.01/2| x>(1-0.01/2))},3), format="f", nsmall=3), symbols.p01(x),sep=""))))},method="cross",g=4)

print(x,prn=F)

x<-summary(results.nspp.Normal.Nrandom.tausigmarandom2.Sc3.conjugate.R [,"var"]~mult.sigma+theta0, fun=function(x){c(

ks.D=( (paste(" ks.D=",format(round(ks.test(x,"punif")$s,3), format="f", nsmall=3), symbols.ks(x),sep=""))),

p.0.05=( (paste(" p.05=",format(round({mean(x<0.05/2| x>(1-0.05/2))},3), format="f", nsmall=3), symbols.p05(x),sep=""))),

p.0.01=( (paste(" p.01=",format(round({mean(x<0.01/2| x>(1-0.01/2))},3), format="f", nsmall=3), symbols.p01(x),sep=""))))},method="cross",g=4)

print(x,prn=F)

x<-summary(results.nspp.Normal.Nrandom.tausigmarandom2.Sc3.conjugate.R [,"skew"]~mult.sigma+theta0, fun=function(x){c(

ks.D=( (paste(" ks.D=",format(round(ks.test(x,"punif")$s,3), format="f", nsmall=3), symbols.ks(x),sep=""))),

p.0.05=( (paste(" p.05=",format(round({mean(x<0.05/2| x>(1-0.05/2))},3), format="f", nsmall=3), symbols.p05(x),sep=""))),

p.0.01=( (paste(" p.01=",format(round({mean(x<0.01/2| x>(1-0.01/2))},3), format="f", nsmall=3), symbols.p01(x),sep=""))))},method="cross",g=4)

print(x,prn=F)

x<-summary(results.nspp.Normal.Nrandom.tausigmarandom2.Sc3.conjugate.R [,"kurt"]~mult.sigma+theta0, fun=function(x){c(

ks.D=( (paste(" ks.D=",format(round(ks.test(x,"punif")$s,3), format="f", nsmall=3), symbols.ks(x),sep=""))),

p.0.05=( (paste(" p.05=",format(round({mean(x<0.05/2| x>(1-0.05/2))},3), format="f", nsmall=3), symbols.p05(x),sep=""))),

p.0.01=( (paste(" p.01=",format(round({mean(x<0.01/2| x>(1-0.01/2))},3), format="f", nsmall=3), symbols.p01(x),sep=""))))},method="cross",g=4)

print(x,prn=F)

x<-summary(results.nspp.Normal.Nrandom.tausigmarandom2.Sc3.conjugate.R [,"Za"]~mult.sigma+theta0, fun=function(x){c(

ks.D=( (paste(" ks.D=",format(round(ks.test(x,"punif")$s,3), format="f", nsmall=3), symbols.ks(x),sep=""))),

p.0.05=( (paste(" p.05=",format(round({mean(x<0.05/2| x>(1-0.05/2))},3), format="f", nsmall=3), symbols.p05(x),sep=""))),

p.0.01=( (paste(" p.01=",format(round({mean(x<0.01/2| x>(1-0.01/2))},3), format="f", nsmall=3), symbols.p01(x),sep=""))))},method="cross",g=4)

print(x,prn=F)

**#R, nspp, conjugate, No et mu**

**#pas présdenté car marginalement plus discriminant seulement pour theta élévé et beaucoup mopins discriminant pour theta faible (que mult.sigma+mu)**

No<- results.nspp.Normal.Nrandom.tausigmarandom2.Sc3.conjugate.R [,"N"]

#mult.sigma<- cut2(round(results.nspp.Normal.Nrandom.tausigmarandom2.Sc3.conjugate.R[,"musigma0"],digits=2), c(1,2,4,6,10))

mult.sigma<- cut2(round(results.nspp.Normal.Nrandom.tausigmarandom2.Sc3.conjugate.R[,"musigma0"],digits=2), g=4)

#mult.sigma<- round(results.nspp.Normal.Nrandom.tausigmarandom2.Sc3.conjugate.R[,"musigma0"],digits=2)

#theta0<- round((results.nspp.Normal.Nrandom.tausigmarandom2.Sc3.conjugate.R[,"theta0"]),digits=1)

theta0<- round(abs(results.nspp.Normal.Nrandom.tausigmarandom2.Sc3.conjugate.R[,"theta0"]),digits=1)

**x<-summary(**results.nspp.Normal.Nrandom.tausigmarandom2.Sc3.conjugate.R [,"mean"]~No+theta0, fun=function(x){c(

ks.D=( (paste(" ks.D=",format(round(ks.test(x,"punif")$s,3), format="f", nsmall=3), symbols.ks(x),sep=""))),

p.0.05=( (paste(" p.05=",format(round({mean(x<0.05/2| x>(1-0.05/2))},3), format="f", nsmall=3), symbols.p05(x),sep=""))),

p.0.01=( (paste(" p.01=",format(round({mean(x<0.01/2| x>(1-0.01/2))},3), format="f", nsmall=3), symbols.p01(x),sep=""))))},method="cross",g=4)

print(x,prn=F)

x<-summary(results.nspp.Normal.Nrandom.tausigmarandom2.Sc3.conjugate.R [,"var"]~No+theta0, fun=function(x){c(

ks.D=( (paste(" ks.D=",format(round(ks.test(x,"punif")$s,3), format="f", nsmall=3), symbols.ks(x),sep=""))),

p.0.05=( (paste(" p.05=",format(round({mean(x<0.05/2| x>(1-0.05/2))},3), format="f", nsmall=3), symbols.p05(x),sep=""))),

p.0.01=( (paste(" p.01=",format(round({mean(x<0.01/2| x>(1-0.01/2))},3), format="f", nsmall=3), symbols.p01(x),sep=""))))},method="cross",g=4)

print(x,prn=F)

x<-summary(results.nspp.Normal.Nrandom.tausigmarandom2.Sc3.conjugate.R [,"skew"]~No+theta0, fun=function(x){c(

ks.D=( (paste(" ks.D=",format(round(ks.test(x,"punif")$s,3), format="f", nsmall=3), symbols.ks(x),sep=""))),

p.0.05=( (paste(" p.05=",format(round({mean(x<0.05/2| x>(1-0.05/2))},3), format="f", nsmall=3), symbols.p05(x),sep=""))),

p.0.01=( (paste(" p.01=",format(round({mean(x<0.01/2| x>(1-0.01/2))},3), format="f", nsmall=3), symbols.p01(x),sep=""))))},method="cross",g=4)

print(x,prn=F)

x<-summary(results.nspp.Normal.Nrandom.tausigmarandom2.Sc3.conjugate.R [,"kurt"]~No+theta0, fun=function(x){c(

ks.D=( (paste(" ks.D=",format(round(ks.test(x,"punif")$s,3), format="f", nsmall=3), symbols.ks(x),sep=""))),

p.0.05=( (paste(" p.05=",format(round({mean(x<0.05/2| x>(1-0.05/2))},3), format="f", nsmall=3), symbols.p05(x),sep=""))),

p.0.01=( (paste(" p.01=",format(round({mean(x<0.01/2| x>(1-0.01/2))},3), format="f", nsmall=3), symbols.p01(x),sep=""))))},method="cross",g=4)

print(x,prn=F)

x<-summary(results.nspp.Normal.Nrandom.tausigmarandom2.Sc3.conjugate.R [,"Za"]~No+theta0, fun=function(x){c(

ks.D=( (paste(" ks.D=",format(round(ks.test(x,"punif")$s,3), format="f", nsmall=3), symbols.ks(x),sep=""))),

p.0.05=( (paste(" p.05=",format(round({mean(x<0.05/2| x>(1-0.05/2))},3), format="f", nsmall=3), symbols.p05(x),sep=""))),

p.0.01=( (paste(" p.01=",format(round({mean(x<0.01/2| x>(1-0.01/2))},3), format="f", nsmall=3), symbols.p01(x),sep=""))))},method="cross",g=4)

print(x,prn=F)

**#(((R, nspp, conjugate, sigma et mu scaled))) pas très différent finalement**

No<- results.nspp.Normal.Nrandom.tausigmarandom2.Sc3.conjugate.R [,"N"]

#mult.sigma<- cut2(round(results.nspp.Normal.Nrandom.tausigmarandom2.Sc3.conjugate.R[,"musigma0"],digits=2), c(1,2,4,6,10))

mult.sigma<- cut2(round(results.nspp.Normal.Nrandom.tausigmarandom2.Sc3.conjugate.R[,"musigma0"],digits=2), g=4)

#mult.sigma<- round(results.nspp.Normal.Nrandom.tausigmarandom2.Sc3.conjugate.R[,"musigma0"],digits=2)

#theta0<- round((results.nspp.Normal.Nrandom.tausigmarandom2.Sc3.conjugate.R[,"theta0"]),digits=1)

theta0<- round(abs(results.nspp.Normal.Nrandom.tausigmarandom2.Sc3.conjugate.R[,"theta0"]/ results.nspp.Normal.Nrandom.tausigmarandom2.Sc3.conjugate.R[,"sigma0"]),digits=1)

**x<-summary(**results.nspp.Normal.Nrandom.tausigmarandom2.Sc3.conjugate.R [,"mean"]~mult.sigma+theta0, fun=function(x){c(

ks.D=( (paste(" ks.D=",format(round(ks.test(x,"punif")$s,3), format="f", nsmall=3), symbols.ks(x),sep=""))),

p.0.05=( (paste(" p.05=",format(round({mean(x<0.05/2| x>(1-0.05/2))},3), format="f", nsmall=3), symbols.p05(x),sep=""))),

p.0.01=( (paste(" p.01=",format(round({mean(x<0.01/2| x>(1-0.01/2))},3), format="f", nsmall=3), symbols.p01(x),sep=""))))},method="cross",g=4)

print(x,prn=F)

x<-summary(results.nspp.Normal.Nrandom.tausigmarandom2.Sc3.conjugate.R [,"var"]~mult.sigma+theta0, fun=function(x){c(

ks.D=( (paste(" ks.D=",format(round(ks.test(x,"punif")$s,3), format="f", nsmall=3), symbols.ks(x),sep=""))),

p.0.05=( (paste(" p.05=",format(round({mean(x<0.05/2| x>(1-0.05/2))},3), format="f", nsmall=3), symbols.p05(x),sep=""))),

p.0.01=( (paste(" p.01=",format(round({mean(x<0.01/2| x>(1-0.01/2))},3), format="f", nsmall=3), symbols.p01(x),sep=""))))},method="cross",g=4)

print(x,prn=F)

x<-summary(results.nspp.Normal.Nrandom.tausigmarandom2.Sc3.conjugate.R [,"skew"]~mult.sigma+theta0, fun=function(x){c(

ks.D=( (paste(" ks.D=",format(round(ks.test(x,"punif")$s,3), format="f", nsmall=3), symbols.ks(x),sep=""))),

p.0.05=( (paste(" p.05=",format(round({mean(x<0.05/2| x>(1-0.05/2))},3), format="f", nsmall=3), symbols.p05(x),sep=""))),

p.0.01=( (paste(" p.01=",format(round({mean(x<0.01/2| x>(1-0.01/2))},3), format="f", nsmall=3), symbols.p01(x),sep=""))))},method="cross",g=4)

print(x,prn=F)

x<-summary(results.nspp.Normal.Nrandom.tausigmarandom2.Sc3.conjugate.R [,"kurt"]~mult.sigma+theta0, fun=function(x){c(

ks.D=( (paste(" ks.D=",format(round(ks.test(x,"punif")$s,3), format="f", nsmall=3), symbols.ks(x),sep=""))),

p.0.05=( (paste(" p.05=",format(round({mean(x<0.05/2| x>(1-0.05/2))},3), format="f", nsmall=3), symbols.p05(x),sep=""))),

p.0.01=( (paste(" p.01=",format(round({mean(x<0.01/2| x>(1-0.01/2))},3), format="f", nsmall=3), symbols.p01(x),sep=""))))},method="cross",g=4)

print(x,prn=F)

x<-summary(results.nspp.Normal.Nrandom.tausigmarandom2.Sc3.conjugate.R [,"Za"]~mult.sigma+theta0, fun=function(x){c(

ks.D=( (paste(" ks.D=",format(round(ks.test(x,"punif")$s,3), format="f", nsmall=3), symbols.ks(x),sep=""))),

p.0.05=( (paste(" p.05=",format(round({mean(x<0.05/2| x>(1-0.05/2))},3), format="f", nsmall=3), symbols.p05(x),sep=""))),

p.0.01=( (paste(" p.01=",format(round({mean(x<0.01/2| x>(1-0.01/2))},3), format="f", nsmall=3), symbols.p01(x),sep=""))))},method="cross",g=4)

print(x,prn=F)

#### #=>A3: Normal, spp & dspp & dspp2, conjugate

**#R, spp, conjugate, sigma et mu**

No<- results.spp.Normal.Nrandom.tausigmarandom2.Sc3.conjugate.R [,"N"]

#mult.sigma<- cut2(round(results.spp.Normal.Nrandom.tausigmarandom2.Sc3.conjugate.R[,"musigma0"],digits=2), g=6)

mult.sigma<- round(results.spp.Normal.Nrandom.tausigmarandom2.Sc3.conjugate.R[,"musigma0"],digits=2)

#theta0<- round((results.nspp.Normal.Nrandom.tausigmarandom2.Sc3.conjugate.R[,"theta0"]),digits=1)

theta0<- round(abs(results.spp.Normal.Nrandom.tausigmarandom2.Sc3.conjugate.R[,"theta0"]),digits=1)

**x<-summary(**results.spp.Normal.Nrandom.tausigmarandom2.Sc3.conjugate.R [,"mean"]~mult.sigma+theta0, fun=function(x){c(

ks.D=( (paste(" ks.D=",format(round(ks.test(x,"punif")$s,3), format="f", nsmall=3), symbols.ks(x),sep=""))),

p.0.05=( (paste(" p.05=",format(round({mean(x<0.05/2| x>(1-0.05/2))},3), format="f", nsmall=3), symbols.p05(x),sep=""))),

p.0.01=( (paste(" p.01=",format(round({mean(x<0.01/2| x>(1-0.01/2))},3), format="f", nsmall=3), symbols.p01(x),sep=""))))},method="cross",g=4)

print(x,prn=F)

x<-summary(results.spp.Normal.Nrandom.tausigmarandom2.Sc3.conjugate.R [,"var"]~mult.sigma+theta0, fun=function(x){c(

ks.D=( (paste(" ks.D=",format(round(ks.test(x,"punif")$s,3), format="f", nsmall=3), symbols.ks(x),sep=""))),

p.0.05=( (paste(" p.05=",format(round({mean(x<0.05/2| x>(1-0.05/2))},3), format="f", nsmall=3), symbols.p05(x),sep=""))),

p.0.01=( (paste(" p.01=",format(round({mean(x<0.01/2| x>(1-0.01/2))},3), format="f", nsmall=3), symbols.p01(x),sep=""))))},method="cross",g=4)

print(x,prn=F)

**#R, dspp, conjugate, sigma et mu**

No<- results.dspp.Normal.Nrandom.tausigmarandom2.Sc3.conjugate.R [,"N"]

#mult.sigma<- cut2(round(results.dspp.Normal.Nrandom.tausigmarandom2.Sc3.conjugate.R[,"musigma0"],digits=2), g=6)

mult.sigma<- round(results.dspp.Normal.Nrandom.tausigmarandom2.Sc3.conjugate.R[,"musigma0"],digits=2)

#theta0<- round((results.dspp.Normal.Nrandom.tausigmarandom2.Sc3.conjugate.R[,"theta0"]),digits=1)

theta0<- round(abs(results.dspp.Normal.Nrandom.tausigmarandom2.Sc3.conjugate.R[,"theta0"]),digits=1)

x<-summary(results.dspp.Normal.Nrandom.tausigmarandom2.Sc3.conjugate.R [,"plt0"]~mult.sigma+theta0, fun=function(x){c(

ks.D=( (paste(" ks.D=",format(round(ks.test(x,"punif")$s,3), format="f", nsmall=3), symbols.ks(x),sep=""))),

p.0.05=( (paste(" p.05=",format(round({mean(x<0.05/2| x>(1-0.05/2))},3), format="f", nsmall=3), symbols.p05(x),sep=""))),

p.0.01=( (paste(" p.01=",format(round({mean(x<0.01/2| x>(1-0.01/2))},3), format="f", nsmall=3), symbols.p01(x),sep=""))))},method="cross",g=4)

print(x,prn=F)

x<-summary(results.dspp.Normal.Nrandom.tausigmarandom2.Sc3.conjugate.R [,"meanc"]~mult.sigma+theta0, fun=function(x){c(

ks.D=( (paste(" ks.D=",format(round(ks.test(x,"punif")$s,3), format="f", nsmall=3), symbols.ks(x),sep=""))),

p.0.05=( (paste(" p.05=",format(round({mean(x<0.05/2| x>(1-0.05/2))},3), format="f", nsmall=3), symbols.p05(x),sep=""))),

p.0.01=( (paste(" p.01=",format(round({mean(x<0.01/2| x>(1-0.01/2))},3), format="f", nsmall=3), symbols.p01(x),sep=""))))},method="cross",g=4)

print(x,prn=F)

x<-summary(results.dspp.Normal.Nrandom.tausigmarandom2.Sc3.conjugate.R [,"varc"]~mult.sigma+theta0, fun=function(x){c(

ks.D=( (paste(" ks.D=",format(round(ks.test(x,"punif")$s,3), format="f", nsmall=3), symbols.ks(x),sep=""))),

p.0.05=( (paste(" p.05=",format(round({mean(x<0.05/2| x>(1-0.05/2))},3), format="f", nsmall=3), symbols.p05(x),sep=""))),

p.0.01=( (paste(" p.01=",format(round({mean(x<0.01/2| x>(1-0.01/2))},3), format="f", nsmall=3), symbols.p01(x),sep=""))))},method="cross",g=4)

print(x,prn=F)

**#R, dspp2, conjugate, sigma et mu**

No<- results.dspp2.Normal.Nrandom.tausigmarandom2.Sc3.conjugate.R [,"N"]

#mult.sigma<- cut2(round(results.dspp2.Normal.Nrandom.tausigmarandom2.Sc3.conjugate.R[,"musigma0"],digits=2), g=6)

mult.sigma<- round(results.dspp2.Normal.Nrandom.tausigmarandom2.Sc3.conjugate.R[,"musigma0"],digits=2)

#theta0<- round((results.dspp2.Normal.Nrandom.tausigmarandom2.Sc3.conjugate.R[,"theta0"]),digits=1)

theta0<- round(abs(results.dspp2.Normal.Nrandom.tausigmarandom2.Sc3.conjugate.R[,"theta0"]),digits=1)

x<-summary(results.dspp2.Normal.Nrandom.tausigmarandom2.Sc3.conjugate.R [,"LL"]~mult.sigma+theta0, fun=function(x){c(

ks.D=( (paste(" ks.D=",format(round(ks.test(x,"punif")$s,3), format="f", nsmall=3), symbols.ks(x),sep=""))),

p.0.05=( (paste(" p.05=",format(round({mean(x<0.05/2| x>(1-0.05/2))},3), format="f", nsmall=3), symbols.p05(x),sep=""))),

p.0.01=( (paste(" p.01=",format(round({mean(x<0.01/2| x>(1-0.01/2))},3), format="f", nsmall=3), symbols.p01(x),sep=""))))},method="cross",g=4)

print(x,prn=F)

#### #=>A3: Bernoulli, nspp, conjugate

#important remark: musigma0 inverted due to inversion between ppaer and commands here.

**#R, nspp, conjugate, sigma et mu**

No<- results.nspp.Bernoulli.Nrandom.thetarandom5.Sc3.conjugate.R [,"N"]

mult.sigma<- round(1/results.nspp.Bernoulli.Nrandom.thetarandom5.Sc3.conjugate.R[,"musigma0"],digits=2)

theta0<- round(abs(results.nspp.Bernoulli.Nrandom.thetarandom5.Sc3.conjugate.R[,"theta0"]-0.5),digits=2)

**x<-summary(**results.nspp.Bernoulli.Nrandom.thetarandom5.Sc3.conjugate.R [,"mean"]~mult.sigma+theta0, fun=function(x){c(

ks.D=( (paste(" ks.D=",format(round(ks.test(x,"punif")$s,3), format="f", nsmall=3), symbols.ks(x),sep=""))),

p.0.05=( (paste(" p.05=",format(round({mean(x<0.05/2| x>(1-0.05/2))},3), format="f", nsmall=3), symbols.p05(x),sep=""))),

p.0.01=( (paste(" p.01=",format(round({mean(x<0.01/2| x>(1-0.01/2))},3), format="f", nsmall=3), symbols.p01(x),sep=""))))},method="cross",g=4)

print(x,prn=F)

x<-summary(results.nspp.Bernoulli.Nrandom.thetarandom5.Sc3.conjugate.R [,"var"]~mult.sigma+theta0, fun=function(x){c(

ks.D=( (paste(" ks.D=",format(round(ks.test(x,"punif")$s,3), format="f", nsmall=3), symbols.ks(x),sep=""))),

p.0.05=( (paste(" p.05=",format(round({mean(x<0.05/2| x>(1-0.05/2))},3), format="f", nsmall=3), symbols.p05(x),sep=""))),

p.0.01=( (paste(" p.01=",format(round({mean(x<0.01/2| x>(1-0.01/2))},3), format="f", nsmall=3), symbols.p01(x),sep=""))))},method="cross",g=4)

print(x,prn=F)

x<-summary(results.nspp.Bernoulli.Nrandom.thetarandom5.Sc3.conjugate.R [,"skew"]~mult.sigma+theta0, fun=function(x){c(

ks.D=( (paste(" ks.D=",format(round(ks.test(x,"punif")$s,3), format="f", nsmall=3), symbols.ks(x),sep=""))),

p.0.05=( (paste(" p.05=",format(round({mean(x<0.05/2| x>(1-0.05/2))},3), format="f", nsmall=3), symbols.p05(x),sep=""))),

p.0.01=( (paste(" p.01=",format(round({mean(x<0.01/2| x>(1-0.01/2))},3), format="f", nsmall=3), symbols.p01(x),sep=""))))},method="cross",g=4)

print(x,prn=F)

x<-summary(results.nspp.Bernoulli.Nrandom.thetarandom5.Sc3.conjugate.R [,"kurt"]~mult.sigma+theta0, fun=function(x){c(

ks.D=( (paste(" ks.D=",format(round(ks.test(x,"punif")$s,3), format="f", nsmall=3), symbols.ks(x),sep=""))),

p.0.05=( (paste(" p.05=",format(round({mean(x<0.05/2| x>(1-0.05/2))},3), format="f", nsmall=3), symbols.p05(x),sep=""))),

p.0.01=( (paste(" p.01=",format(round({mean(x<0.01/2| x>(1-0.01/2))},3), format="f", nsmall=3), symbols.p01(x),sep=""))))},method="cross",g=4)

print(x,prn=F)

x<-summary(results.nspp.Bernoulli.Nrandom.thetarandom5.Sc3.conjugate.R [,"Za"]~mult.sigma+theta0, fun=function(x){c(

ks.D=( (paste(" ks.D=",format(round(ks.test(x,"punif")$s,3), format="f", nsmall=3), symbols.ks(x),sep=""))),

p.0.05=( (paste(" p.05=",format(round({mean(x<0.05/2| x>(1-0.05/2))},3), format="f", nsmall=3), symbols.p05(x),sep=""))),

p.0.01=( (paste(" p.01=",format(round({mean(x<0.01/2| x>(1-0.01/2))},3), format="f", nsmall=3), symbols.p01(x),sep=""))))},method="cross",g=4)

print(x,prn=F)

#### #=>A3: Bernoulli, spp & dspp & dspp2, conjugate

#important remark: musigma0 inverted due to inversion between ppaer and commands here.

**#R, spp, conjugate, sigma et mu**

No<- results.spp.Bernoulli.Nrandom.thetarandom5.Sc3.conjugate.R [,"N"]

mult.sigma<- round(1/results.spp.Bernoulli.Nrandom.thetarandom5.Sc3.conjugate.R[,"musigma0"],digits=2)

theta0<- round(abs(results.spp.Bernoulli.Nrandom.thetarandom5.Sc3.conjugate.R[,"theta0"]-0.5),digits=2)

**x<-summary(**results.spp.Bernoulli.Nrandom.thetarandom5.Sc3.conjugate.R [,"mean"]~mult.sigma+theta0, fun=function(x){c(

ks.D=( (paste(" ks.D=",format(round(ks.test(x,"punif")$s,3), format="f", nsmall=3), symbols.ks(x),sep=""))),

p.0.05=( (paste(" p.05=",format(round({mean(x<0.05/2| x>(1-0.05/2))},3), format="f", nsmall=3), symbols.p05(x),sep=""))),

p.0.01=( (paste(" p.01=",format(round({mean(x<0.01/2| x>(1-0.01/2))},3), format="f", nsmall=3), symbols.p01(x),sep=""))))},method="cross",g=4)

print(x,prn=F)

x<-summary(results.spp.Bernoulli.Nrandom.thetarandom5.Sc3.conjugate.R [,"var"]~mult.sigma+theta0, fun=function(x){c(

ks.D=( (paste(" ks.D=",format(round(ks.test(x,"punif")$s,3), format="f", nsmall=3), symbols.ks(x),sep=""))),

p.0.05=( (paste(" p.05=",format(round({mean(x<0.05/2| x>(1-0.05/2))},3), format="f", nsmall=3), symbols.p05(x),sep=""))),

p.0.01=( (paste(" p.01=",format(round({mean(x<0.01/2| x>(1-0.01/2))},3), format="f", nsmall=3), symbols.p01(x),sep=""))))},method="cross",g=4)

print(x,prn=F)

**#R, dspp, conjugate, sigma et mu**

No<- results.dspp.Bernoulli.Nrandom.thetarandom5.Sc3.conjugate.R [,"N"]

mult.sigma<- round(1/results.dspp.Bernoulli.Nrandom.thetarandom5.Sc3.conjugate.R[,"musigma0"],digits=2)

theta0<- round(abs(results.dspp.Bernoulli.Nrandom.thetarandom5.Sc3.conjugate.R[,"theta0"]-0.5), digits=2)

**x<-summary(**results.dspp.Bernoulli.Nrandom.thetarandom5.Sc3.conjugate.R [,"p0"]~mult.sigma+theta0, fun=function(x){c(

ks.D=( (paste(" ks.D=",format(round(ks.test(x,"punif")$s,3), format="f", nsmall=3), symbols.ks(x),sep=""))),

p.0.05=( (paste(" p.05=",format(round({mean(x<0.05/2| x>(1-0.05/2))},3), format="f", nsmall=3), symbols.p05(x),sep=""))),

p.0.01=( (paste(" p.01=",format(round({mean(x<0.01/2| x>(1-0.01/2))},3), format="f", nsmall=3), symbols.p01(x),sep=""))))},method="cross",g=4)

print(x,prn=F)

**x<-summary(**results.dspp.Bernoulli.Nrandom.thetarandom5.Sc3.conjugate.R [,"meanc"]~mult.sigma+theta0, fun=function(x){c(

ks.D=( (paste(" ks.D=",format(round(ks.test(x,"punif")$s,3), format="f", nsmall=3), symbols.ks(x),sep=""))),

p.0.05=( (paste(" p.05=",format(round({mean(x<0.05/2| x>(1-0.05/2))},3), format="f", nsmall=3), symbols.p05(x),sep=""))),

p.0.01=( (paste(" p.01=",format(round({mean(x<0.01/2| x>(1-0.01/2))},3), format="f", nsmall=3), symbols.p01(x),sep=""))))},method="cross",g=4)

print(x,prn=F)

x<-summary(results.dspp.Bernoulli.Nrandom.thetarandom5.Sc3.conjugate.R [,"varc"]~mult.sigma+theta0, fun=function(x){c(

ks.D=( (paste(" ks.D=",format(round(ks.test(x,"punif")$s,3), format="f", nsmall=3), symbols.ks(x),sep=""))),

p.0.05=( (paste(" p.05=",format(round({mean(x<0.05/2| x>(1-0.05/2))},3), format="f", nsmall=3), symbols.p05(x),sep=""))),

p.0.01=( (paste(" p.01=",format(round({mean(x<0.01/2| x>(1-0.01/2))},3), format="f", nsmall=3), symbols.p01(x),sep=""))))},method="cross",g=4)

print(x,prn=F)

**#R, dspp2, conjugate, sigma et mu**

No<- results.dspp2.Bernoulli.Nrandom.thetarandom5.Sc3.conjugate.R [,"N"]

mult.sigma<- round(1/results.dspp2.Bernoulli.Nrandom.thetarandom5.Sc3.conjugate.R[,"musigma0"],digits=2)

theta0<- round(abs(results.dspp2.Bernoulli.Nrandom.thetarandom5.Sc3.conjugate.R[,"theta0"]-0.5), digits=2)

**x<-summary(**results.dspp2.Bernoulli.Nrandom.thetarandom5.Sc3.conjugate.R [,"LL"]~mult.sigma+theta0, fun=function(x){c(

ks.D=( (paste(" ks.D=",format(round(ks.test(x,"punif")$s,3), format="f", nsmall=3), symbols.ks(x),sep=""))),

p.0.05=( (paste(" p.05=",format(round({mean(x<0.05/2| x>(1-0.05/2))},3), format="f", nsmall=3), symbols.p05(x),sep=""))),

p.0.01=( (paste(" p.01=",format(round({mean(x<0.01/2| x>(1-0.01/2))},3), format="f", nsmall=3), symbols.p01(x),sep=""))))},method="cross",g=4)

print(x,prn=F)

## Tables for Text S4 (Scenario 4 results)

#### #=>A4: Poisson, nspp, conjugate

**#R, nspp, conjugate, sigma et N**

No<- results.nspp.Poisson.Nrandom.theta1.sigma2.taurandomV1.Sc4.conjugate.R [,"N"]

sigma0<- round(results.nspp.Poisson.Nrandom.theta1.sigma2.taurandomV1.Sc4.conjugate.R[,"sigma0"],digits=2)

**x<-summary(**results.nspp.Poisson.Nrandom.theta1.sigma2.taurandomV1.Sc4.conjugate.R [,"mean"]~sigma0+No, fun=function(x){c(

ks.D=( (paste(" ks.D=",format(round(ks.test(x,"punif")$s,3), format="f", nsmall=3), symbols.ks(x),sep=""))),

p.0.05=( (paste(" p.05=",format(round({mean(x<0.05/2| x>(1-0.05/2))},3), format="f", nsmall=3), symbols.p05(x),sep=""))),

p.0.01=( (paste(" p.01=",format(round({mean(x<0.01/2| x>(1-0.01/2))},3), format="f", nsmall=3), symbols.p01(x),sep=""))))},method="cross",g=4)

print(x,prn=F)

x<-summary(results.nspp.Poisson.Nrandom.theta1.sigma2.taurandomV1.Sc4.conjugate.R [,"var"]~sigma0+No, fun=function(x){c(

ks.D=( (paste(" ks.D=",format(round(ks.test(x,"punif")$s,3), format="f", nsmall=3), symbols.ks(x),sep=""))),

p.0.05=( (paste(" p.05=",format(round({mean(x<0.05/2| x>(1-0.05/2))},3), format="f", nsmall=3), symbols.p05(x),sep=""))),

p.0.01=( (paste(" p.01=",format(round({mean(x<0.01/2| x>(1-0.01/2))},3), format="f", nsmall=3), symbols.p01(x),sep=""))))},method="cross",g=4)

print(x,prn=F)

x<-summary(results.nspp.Poisson.Nrandom.theta1.sigma2.taurandomV1.Sc4.conjugate.R [,"skew"]~sigma0+No, fun=function(x){c(

ks.D=( (paste(" ks.D=",format(round(ks.test(x,"punif")$s,3), format="f", nsmall=3), symbols.ks(x),sep=""))),

p.0.05=( (paste(" p.05=",format(round({mean(x<0.05/2| x>(1-0.05/2))},3), format="f", nsmall=3), symbols.p05(x),sep=""))),

p.0.01=( (paste(" p.01=",format(round({mean(x<0.01/2| x>(1-0.01/2))},3), format="f", nsmall=3), symbols.p01(x),sep=""))))},method="cross",g=4)

print(x,prn=F)

x<-summary(results.nspp.Poisson.Nrandom.theta1.sigma2.taurandomV1.Sc4.conjugate.R [,"kurt"]~sigma0+No, fun=function(x){c(

ks.D=( (paste(" ks.D=",format(round(ks.test(x,"punif")$s,3), format="f", nsmall=3), symbols.ks(x),sep=""))),

p.0.05=( (paste(" p.05=",format(round({mean(x<0.05/2| x>(1-0.05/2))},3), format="f", nsmall=3), symbols.p05(x),sep=""))),

p.0.01=( (paste(" p.01=",format(round({mean(x<0.01/2| x>(1-0.01/2))},3), format="f", nsmall=3), symbols.p01(x),sep=""))))},method="cross",g=4)

print(x,prn=F)

x<-summary(results.nspp.Poisson.Nrandom.theta1.sigma2.taurandomV1.Sc4.conjugate.R [,"Za"]~sigma0+No, fun=function(x){c(

ks.D=( (paste(" ks.D=",format(round(ks.test(x,"punif")$s,3), format="f", nsmall=3), symbols.ks(x),sep=""))),

p.0.05=( (paste(" p.05=",format(round({mean(x<0.05/2| x>(1-0.05/2))},3), format="f", nsmall=3), symbols.p05(x),sep=""))),

p.0.01=( (paste(" p.01=",format(round({mean(x<0.01/2| x>(1-0.01/2))},3), format="f", nsmall=3), symbols.p01(x),sep=""))))},method="cross",g=4)

print(x,prn=F)

#### #=>A4: Poisson, spp et dspp & dspp2, conjugate

**#R, spp, conjugate, sigma et N**

No<- results.spp.Poisson.Nrandom.theta1.sigma2.taurandomV1.Sc4.conjugate.R [,"N"]

sigma0<- round(results.spp.Poisson.Nrandom.theta1.sigma2.taurandomV1.Sc4.conjugate.R[,"sigma0"], digits=2)

**x<-summary(**results.spp.Poisson.Nrandom.theta1.sigma2.taurandomV1.Sc4.conjugate.R [,"mean"]~sigma0+No, fun=function(x){c(

ks.D=( (paste(" ks.D=",format(round(ks.test(x,"punif")$s,3), format="f", nsmall=3), symbols.ks(x),sep=""))),

p.0.05=( (paste(" p.05=",format(round({mean(x<0.05/2| x>(1-0.05/2))},3), format="f", nsmall=3), symbols.p05(x),sep=""))),

p.0.01=( (paste(" p.01=",format(round({mean(x<0.01/2| x>(1-0.01/2))},3), format="f", nsmall=3), symbols.p01(x),sep=""))))},method="cross",g=4)

print(x,prn=F)

x<-summary(results.spp.Poisson.Nrandom.theta1.sigma2.taurandomV1.Sc4.conjugate.R [,"var"]~sigma0+No, fun=function(x){c(

ks.D=( (paste(" ks.D=",format(round(ks.test(x,"punif")$s,3), format="f", nsmall=3), symbols.ks(x),sep=""))),

p.0.05=( (paste(" p.05=",format(round({mean(x<0.05/2| x>(1-0.05/2))},3), format="f", nsmall=3), symbols.p05(x),sep=""))),

p.0.01=( (paste(" p.01=",format(round({mean(x<0.01/2| x>(1-0.01/2))},3), format="f", nsmall=3), symbols.p01(x),sep=""))))},method="cross",g=4)

print(x,prn=F)

**#R, dspp, conjugate, sigma et N**

No<- results.dspp.Poisson.Nrandom.theta1.sigma2.taurandomV1.Sc4.conjugate.R [,"N"]

sigma0<- round(results.dspp.Poisson.Nrandom.theta1.sigma2.taurandomV1.Sc4.conjugate.R[,"sigma0"],digits=2)

x<-summary(results.dspp.Poisson.Nrandom.theta1.sigma2.taurandomV1.Sc4.conjugate.R [,"p0"]~sigma0+No, fun=function(x){c(

ks.D=( (paste(" ks.D=",format(round(ks.test(x,"punif")$s,3), format="f", nsmall=3), symbols.ks(x),sep=""))),

p.0.05=( (paste(" p.05=",format(round({mean(x<0.05/2| x>(1-0.05/2))},3), format="f", nsmall=3), symbols.p05(x),sep=""))),

p.0.01=( (paste(" p.01=",format(round({mean(x<0.01/2| x>(1-0.01/2))},3), format="f", nsmall=3), symbols.p01(x),sep=""))))},method="cross",g=4)

print(x,prn=F)

**x<-summary(**results.dspp.Poisson.Nrandom.theta1.sigma2.taurandomV1.Sc4.conjugate.R [,"meanc"]~sigma0+No, fun=function(x){c(

ks.D=( (paste(" ks.D=",format(round(ks.test(x,"punif")$s,3), format="f", nsmall=3), symbols.ks(x),sep=""))),

p.0.05=( (paste(" p.05=",format(round({mean(x<0.05/2| x>(1-0.05/2))},3), format="f", nsmall=3), symbols.p05(x),sep=""))),

p.0.01=( (paste(" p.01=",format(round({mean(x<0.01/2| x>(1-0.01/2))},3), format="f", nsmall=3), symbols.p01(x),sep=""))))},method="cross",g=4)

print(x,prn=F)

x<-summary(results.dspp.Poisson.Nrandom.theta1.sigma2.taurandomV1.Sc4.conjugate.R [,"varc"]~sigma0+No, fun=function(x){c(

ks.D=( (paste(" ks.D=",format(round(ks.test(x,"punif")$s,3), format="f", nsmall=3), symbols.ks(x),sep=""))),

p.0.05=( (paste(" p.05=",format(round({mean(x<0.05/2| x>(1-0.05/2))},3), format="f", nsmall=3), symbols.p05(x),sep=""))),

p.0.01=( (paste(" p.01=",format(round({mean(x<0.01/2| x>(1-0.01/2))},3), format="f", nsmall=3), symbols.p01(x),sep=""))))},method="cross",g=4)

print(x,prn=F)

**#R, dspp2, conjugate, sigma et N**

No<- results.dspp2.Poisson.Nrandom.theta1.sigma2.taurandomV1.Sc4.conjugate.R [,"N"]

sigma0<- round(results.dspp2.Poisson.Nrandom.theta1.sigma2.taurandomV1.Sc4.conjugate.R[,"sigma0"],digits=2)

x<-summary(results.dspp2.Poisson.Nrandom.theta1.sigma2.taurandomV1.Sc4.conjugate.R [,"LL"]~sigma0+No, fun=function(x){c(

ks.D=( (paste(" ks.D=",format(round(ks.test(x,"punif")$s,3), format="f", nsmall=3), symbols.ks(x),sep=""))),

p.0.05=( (paste(" p.05=",format(round({mean(x<0.05/2| x>(1-0.05/2))},3), format="f", nsmall=3), symbols.p05(x),sep=""))),

p.0.01=( (paste(" p.01=",format(round({mean(x<0.01/2| x>(1-0.01/2))},3), format="f", nsmall=3), symbols.p01(x),sep=""))))},method="cross",g=4)

print(x,prn=F)

#### #=>A4: Normal, nspp, conjugate

**#R, nspp, conjugate, sigma et N**

No<- results.nspp.Normal.Nrandom.tausigmarandom2.Sc4.conjugate.R [,"N"]

sigma0<- round(results.nspp.Normal.Nrandom.tausigmarandom2.Sc4.conjugate.R[,"sigma0"],digits=2)

**x<-summary(**results.nspp.Normal.Nrandom.tausigmarandom2.Sc4.conjugate.R [,"mean"]~sigma0+No, fun=function(x){c(

ks.D=( (paste(" ks.D=",format(round(ks.test(x,"punif")$s,3), format="f", nsmall=3), symbols.ks(x),sep=""))),

p.0.05=( (paste(" p.05=",format(round({mean(x<0.05/2| x>(1-0.05/2))},3), format="f", nsmall=3), symbols.p05(x),sep=""))),

p.0.01=( (paste(" p.01=",format(round({mean(x<0.01/2| x>(1-0.01/2))},3), format="f", nsmall=3), symbols.p01(x),sep=""))))},method="cross",g=4)

print(x,prn=F)

x<-summary(results.nspp.Normal.Nrandom.tausigmarandom2.Sc4.conjugate.R [,"var"]~sigma0+No, fun=function(x){c(

ks.D=( (paste(" ks.D=",format(round(ks.test(x,"punif")$s,3), format="f", nsmall=3), symbols.ks(x),sep=""))),

p.0.05=( (paste(" p.05=",format(round({mean(x<0.05/2| x>(1-0.05/2))},3), format="f", nsmall=3), symbols.p05(x),sep=""))),

p.0.01=( (paste(" p.01=",format(round({mean(x<0.01/2| x>(1-0.01/2))},3), format="f", nsmall=3), symbols.p01(x),sep=""))))},method="cross",g=4)

print(x,prn=F)

x<-summary(results.nspp.Normal.Nrandom.tausigmarandom2.Sc4.conjugate.R [,"skew"]~sigma0+No, fun=function(x){c(

ks.D=( (paste(" ks.D=",format(round(ks.test(x,"punif")$s,3), format="f", nsmall=3), symbols.ks(x),sep=""))),

p.0.05=( (paste(" p.05=",format(round({mean(x<0.05/2| x>(1-0.05/2))},3), format="f", nsmall=3), symbols.p05(x),sep=""))),

p.0.01=( (paste(" p.01=",format(round({mean(x<0.01/2| x>(1-0.01/2))},3), format="f", nsmall=3), symbols.p01(x),sep=""))))},method="cross",g=4)

print(x,prn=F)

x<-summary(results.nspp.Normal.Nrandom.tausigmarandom2.Sc4.conjugate.R [,"kurt"]~sigma0+No, fun=function(x){c(

ks.D=( (paste(" ks.D=",format(round(ks.test(x,"punif")$s,3), format="f", nsmall=3), symbols.ks(x),sep=""))),

p.0.05=( (paste(" p.05=",format(round({mean(x<0.05/2| x>(1-0.05/2))},3), format="f", nsmall=3), symbols.p05(x),sep=""))),

p.0.01=( (paste(" p.01=",format(round({mean(x<0.01/2| x>(1-0.01/2))},3), format="f", nsmall=3), symbols.p01(x),sep=""))))},method="cross",g=4)

print(x,prn=F)

x<-summary(results.nspp.Normal.Nrandom.tausigmarandom2.Sc4.conjugate.R [,"Za"]~sigma0+No, fun=function(x){c(

ks.D=( (paste(" ks.D=",format(round(ks.test(x,"punif")$s,3), format="f", nsmall=3), symbols.ks(x),sep=""))),

p.0.05=( (paste(" p.05=",format(round({mean(x<0.05/2| x>(1-0.05/2))},3), format="f", nsmall=3), symbols.p05(x),sep=""))),

p.0.01=( (paste(" p.01=",format(round({mean(x<0.01/2| x>(1-0.01/2))},3), format="f", nsmall=3), symbols.p01(x),sep=""))))},method="cross",g=4)

print(x,prn=F)

#### #=>A4: Normal, spp&dspp&dspp2, conjugate

**#R, spp, conjugate, sigma et N**

No<- results.spp.Normal.Nrandom.tausigmarandom2.Sc4.conjugate.R [,"N"]

sigma0<- round(results.spp.Normal.Nrandom.tausigmarandom2.Sc4.conjugate.R[,"sigma0"],digits=2)

**x<-summary(**results.spp.Normal.Nrandom.tausigmarandom2.Sc4.conjugate.R [,"mean"]~sigma0+No, fun=function(x){c(

ks.D=( (paste(" ks.D=",format(round(ks.test(x,"punif")$s,3), format="f", nsmall=3), symbols.ks(x),sep=""))),

p.0.05=( (paste(" p.05=",format(round({mean(x<0.05/2| x>(1-0.05/2))},3), format="f", nsmall=3), symbols.p05(x),sep=""))),

p.0.01=( (paste(" p.01=",format(round({mean(x<0.01/2| x>(1-0.01/2))},3), format="f", nsmall=3), symbols.p01(x),sep=""))))},method="cross",g=4)

print(x,prn=F)

x<-summary(results.spp.Normal.Nrandom.tausigmarandom2.Sc4.conjugate.R [,"var"]~sigma0+No, fun=function(x){c(

ks.D=( (paste(" ks.D=",format(round(ks.test(x,"punif")$s,3), format="f", nsmall=3), symbols.ks(x),sep=""))),

p.0.05=( (paste(" p.05=",format(round({mean(x<0.05/2| x>(1-0.05/2))},3), format="f", nsmall=3), symbols.p05(x),sep=""))),

p.0.01=( (paste(" p.01=",format(round({mean(x<0.01/2| x>(1-0.01/2))},3), format="f", nsmall=3), symbols.p01(x),sep=""))))},method="cross",g=4)

print(x,prn=F)

**#R, dspp, conjugate, sigma et N**

No<- results.dspp.Normal.Nrandom.tausigmarandom2.Sc4.conjugate.R [,"N"]

sigma0<- round(results.dspp.Normal.Nrandom.tausigmarandom2.Sc4.conjugate.R[,"sigma0"],digits=2)

**x<-summary(**results.dspp.Normal.Nrandom.tausigmarandom2.Sc4.conjugate.R [,"plt0"]~sigma0+No, fun=function(x){c(

ks.D=( (paste(" ks.D=",format(round(ks.test(x,"punif")$s,3), format="f", nsmall=3), symbols.ks(x),sep=""))),

p.0.05=( (paste(" p.05=",format(round({mean(x<0.05/2| x>(1-0.05/2))},3), format="f", nsmall=3), symbols.p05(x),sep=""))),

p.0.01=( (paste(" p.01=",format(round({mean(x<0.01/2| x>(1-0.01/2))},3), format="f", nsmall=3), symbols.p01(x),sep=""))))},method="cross",g=4)

print(x,prn=F)

**x<-summary(**results.dspp.Normal.Nrandom.tausigmarandom2.Sc4.conjugate.R [,"meanc"]~sigma0+No, fun=function(x){c(

ks.D=( (paste(" ks.D=",format(round(ks.test(x,"punif")$s,3), format="f", nsmall=3), symbols.ks(x),sep=""))),

p.0.05=( (paste(" p.05=",format(round({mean(x<0.05/2| x>(1-0.05/2))},3), format="f", nsmall=3), symbols.p05(x),sep=""))),

p.0.01=( (paste(" p.01=",format(round({mean(x<0.01/2| x>(1-0.01/2))},3), format="f", nsmall=3), symbols.p01(x),sep=""))))},method="cross",g=4)

print(x,prn=F)

x<-summary(results.dspp.Normal.Nrandom.tausigmarandom2.Sc4.conjugate.R [,"varc"]~sigma0+No, fun=function(x){c(

ks.D=( (paste(" ks.D=",format(round(ks.test(x,"punif")$s,3), format="f", nsmall=3), symbols.ks(x),sep=""))),

p.0.05=( (paste(" p.05=",format(round({mean(x<0.05/2| x>(1-0.05/2))},3), format="f", nsmall=3), symbols.p05(x),sep=""))),

p.0.01=( (paste(" p.01=",format(round({mean(x<0.01/2| x>(1-0.01/2))},3), format="f", nsmall=3), symbols.p01(x),sep=""))))},method="cross",g=4)

print(x,prn=F)

**#R, dspp2, conjugate, sigma et N**

No<- results.dspp2.Normal.Nrandom.tausigmarandom2.Sc4.conjugate.R [,"N"]

sigma0<- round(results.dspp2.Normal.Nrandom.tausigmarandom2.Sc4.conjugate.R[,"sigma0"],digits=2)

**x<-summary(**results.dspp2.Normal.Nrandom.tausigmarandom2.Sc4.conjugate.R [,"LL"]~sigma0+No, fun=function(x){c(

ks.D=( (paste(" ks.D=",format(round(ks.test(x,"punif")$s,3), format="f", nsmall=3), symbols.ks(x),sep=""))),

p.0.05=( (paste(" p.05=",format(round({mean(x<0.05/2| x>(1-0.05/2))},3), format="f", nsmall=3), symbols.p05(x),sep=""))),

p.0.01=( (paste(" p.01=",format(round({mean(x<0.01/2| x>(1-0.01/2))},3), format="f", nsmall=3), symbols.p01(x),sep=""))))},method="cross",g=4)

print(x,prn=F)

#### #=>A4: Bernoulli, nspp, conjugate

**#R, nspp, conjugate, sigma et N**

No<- results.nspp.Bernoulli.Nrandom.thetarandom5.Sc4.conjugate.R [,"N"]

inv.rho<- round(1/ results.nspp.Bernoulli.Nrandom.thetarandom5.Sc4.conjugate.R[,"sigma0"], digits=2)

**x<-summary(**results.nspp.Bernoulli.Nrandom.thetarandom5.Sc4.conjugate.R [,"mean"]~inv.rho+No, fun=function(x){c(

ks.D=( (paste(" ks.D=",format(round(ks.test(x,"punif")$s,3), format="f", nsmall=3), symbols.ks(x),sep=""))),

p.0.05=( (paste(" p.05=",format(round({mean(x<0.05/2| x>(1-0.05/2))},3), format="f", nsmall=3), symbols.p05(x),sep=""))),

p.0.01=( (paste(" p.01=",format(round({mean(x<0.01/2| x>(1-0.01/2))},3), format="f", nsmall=3), symbols.p01(x),sep=""))))},method="cross",g=4)

print(x,prn=F)

x<-summary(results.nspp.Bernoulli.Nrandom.thetarandom5.Sc4.conjugate.R [,"var"]~inv.rho+No, fun=function(x){c(

ks.D=( (paste(" ks.D=",format(round(ks.test(x,"punif")$s,3), format="f", nsmall=3), symbols.ks(x),sep=""))),

p.0.05=( (paste(" p.05=",format(round({mean(x<0.05/2| x>(1-0.05/2))},3), format="f", nsmall=3), symbols.p05(x),sep=""))),

p.0.01=( (paste(" p.01=",format(round({mean(x<0.01/2| x>(1-0.01/2))},3), format="f", nsmall=3), symbols.p01(x),sep=""))))},method="cross",g=4)

print(x,prn=F)

x<-summary(results.nspp.Bernoulli.Nrandom.thetarandom5.Sc4.conjugate.R [,"skew"]~inv.rho+No, fun=function(x){c(

ks.D=( (paste(" ks.D=",format(round(ks.test(x,"punif")$s,3), format="f", nsmall=3), symbols.ks(x),sep=""))),

p.0.05=( (paste(" p.05=",format(round({mean(x<0.05/2| x>(1-0.05/2))},3), format="f", nsmall=3), symbols.p05(x),sep=""))),

p.0.01=( (paste(" p.01=",format(round({mean(x<0.01/2| x>(1-0.01/2))},3), format="f", nsmall=3), symbols.p01(x),sep=""))))},method="cross",g=4)

print(x,prn=F)

x<-summary(results.nspp.Bernoulli.Nrandom.thetarandom5.Sc4.conjugate.R [,"kurt"]~inv.rho+No, fun=function(x){c(

ks.D=( (paste(" ks.D=",format(round(ks.test(x,"punif")$s,3), format="f", nsmall=3), symbols.ks(x),sep=""))),

p.0.05=( (paste(" p.05=",format(round({mean(x<0.05/2| x>(1-0.05/2))},3), format="f", nsmall=3), symbols.p05(x),sep=""))),

p.0.01=( (paste(" p.01=",format(round({mean(x<0.01/2| x>(1-0.01/2))},3), format="f", nsmall=3), symbols.p01(x),sep=""))))},method="cross",g=4)

print(x,prn=F)

x<-summary(results.nspp.Bernoulli.Nrandom.thetarandom5.Sc4.conjugate.R [,"Za"]~inv.rho+No, fun=function(x){c(

ks.D=( (paste(" ks.D=",format(round(ks.test(x,"punif")$s,3), format="f", nsmall=3), symbols.ks(x),sep=""))),

p.0.05=( (paste(" p.05=",format(round({mean(x<0.05/2| x>(1-0.05/2))},3), format="f", nsmall=3), symbols.p05(x),sep=""))),

p.0.01=( (paste(" p.01=",format(round({mean(x<0.01/2| x>(1-0.01/2))},3), format="f", nsmall=3), symbols.p01(x),sep=""))))},method="cross",g=4)

print(x,prn=F)

#### #=>A4: Bernoulli, spp & dspp, conjugate

**#R, spp, conjugate, sigma et N**

No<- results.spp.Bernoulli.Nrandom.thetarandom5.Sc4.conjugate.R [,"N"]

inv.rho<- round(1/ results.spp.Bernoulli.Nrandom.thetarandom5.Sc4.conjugate.R[,"sigma0"], digits=2)

**x<-summary(**results.spp.Bernoulli.Nrandom.thetarandom5.Sc4.conjugate.R [,"mean"]~inv.rho+No, fun=function(x){c(

ks.D=( (paste(" ks.D=",format(round(ks.test(x,"punif")$s,3), format="f", nsmall=3), symbols.ks(x),sep=""))),

p.0.05=( (paste(" p.05=",format(round({mean(x<0.05/2| x>(1-0.05/2))},3), format="f", nsmall=3), symbols.p05(x),sep=""))),

p.0.01=( (paste(" p.01=",format(round({mean(x<0.01/2| x>(1-0.01/2))},3), format="f", nsmall=3), symbols.p01(x),sep=""))))},method="cross",g=4)

print(x,prn=F)

x<-summary(results.spp.Bernoulli.Nrandom.thetarandom5.Sc4.conjugate.R [,"var"]~inv.rho+No, fun=function(x){c(

ks.D=( (paste(" ks.D=",format(round(ks.test(x,"punif")$s,3), format="f", nsmall=3), symbols.ks(x),sep=""))),

p.0.05=( (paste(" p.05=",format(round({mean(x<0.05/2| x>(1-0.05/2))},3), format="f", nsmall=3), symbols.p05(x),sep=""))),

p.0.01=( (paste(" p.01=",format(round({mean(x<0.01/2| x>(1-0.01/2))},3), format="f", nsmall=3), symbols.p01(x),sep=""))))},method="cross",g=4)

print(x,prn=F)

**#R, dspp, conjugate, sigma et N**

No<- results.dspp.Bernoulli.Nrandom.thetarandom5.Sc4.conjugate.R [,"N"]

inv.rho<- round(1/results.dspp.Bernoulli.Nrandom.thetarandom5.Sc4.conjugate.R[,"sigma0"], digits=2)

**x<-summary(**results.dspp.Bernoulli.Nrandom.thetarandom5.Sc4.conjugate.R [,"p0"]~inv.rho+No, fun=function(x){c(

ks.D=( (paste(" ks.D=",format(round(ks.test(x,"punif")$s,3), format="f", nsmall=3), symbols.ks(x),sep=""))),

p.0.05=( (paste(" p.05=",format(round({mean(x<0.05/2| x>(1-0.05/2))},3), format="f", nsmall=3), symbols.p05(x),sep=""))),

p.0.01=( (paste(" p.01=",format(round({mean(x<0.01/2| x>(1-0.01/2))},3), format="f", nsmall=3), symbols.p01(x),sep=""))))},method="cross",g=4)

print(x,prn=F)

**x<-summary(**results.dspp.Bernoulli.Nrandom.thetarandom5.Sc4.conjugate.R [,"meanc"]~inv.rho+No, fun=function(x){c(

ks.D=( (paste(" ks.D=",format(round(ks.test(x,"punif")$s,3), format="f", nsmall=3), symbols.ks(x),sep=""))),

p.0.05=( (paste(" p.05=",format(round({mean(x<0.05/2| x>(1-0.05/2))},3), format="f", nsmall=3), symbols.p05(x),sep=""))),

p.0.01=( (paste(" p.01=",format(round({mean(x<0.01/2| x>(1-0.01/2))},3), format="f", nsmall=3), symbols.p01(x),sep=""))))},method="cross",g=4)

print(x,prn=F)

x<-summary(results.dspp.Bernoulli.Nrandom.thetarandom5.Sc4.conjugate.R [,"varc"]~inv.rho+No, fun=function(x){c(

ks.D=( (paste(" ks.D=",format(round(ks.test(x,"punif")$s,3), format="f", nsmall=3), symbols.ks(x),sep=""))),

p.0.05=( (paste(" p.05=",format(round({mean(x<0.05/2| x>(1-0.05/2))},3), format="f", nsmall=3), symbols.p05(x),sep=""))),

p.0.01=( (paste(" p.01=",format(round({mean(x<0.01/2| x>(1-0.01/2))},3), format="f", nsmall=3), symbols.p01(x),sep=""))))},method="cross",g=4)

print(x,prn=F)

**#R, dspp2, conjugate, sigma et N**

No<- results.dspp2.Bernoulli.Nrandom.thetarandom5.Sc4.conjugate.R [,"N"]

inv.rho<- round(1/results.dspp2.Bernoulli.Nrandom.thetarandom5.Sc4.conjugate.R[,"sigma0"], digits=2)

**x<-summary(**results.dspp2.Bernoulli.Nrandom.thetarandom5.Sc4.conjugate.R [,"LL"]~inv.rho+No, fun=function(x){c(

ks.D=( (paste(" ks.D=",format(round(ks.test(x,"punif")$s,3), format="f", nsmall=3), symbols.ks(x),sep=""))),

p.0.05=( (paste(" p.05=",format(round({mean(x<0.05/2| x>(1-0.05/2))},3), format="f", nsmall=3), symbols.p05(x),sep=""))),

p.0.01=( (paste(" p.01=",format(round({mean(x<0.01/2| x>(1-0.01/2))},3), format="f", nsmall=3), symbols.p01(x),sep=""))))},method="cross",g=4)

print(x,prn=F)

**#R, theta=0.7, spp, conjugate, sigma et N**

No<- results.spp.Bernoulli.theta07.Nrandom.thetarandom5.Sc4.conjugate.R [,"N"]

inv.rho<- round(1/results.spp.Bernoulli.theta07.Nrandom.thetarandom5.Sc4.conjugate.R[,"sigma0"], digits=2)

**x<-summary(**results.spp.Bernoulli.theta07.Nrandom.thetarandom5.Sc4.conjugate.R [,"mean"]~inv.rho+No, fun=function(x){c(

ks.D=( (paste(" ks.D=",format(round(ks.test(x,"punif")$s,3), format="f", nsmall=3), symbols.ks(x),sep=""))),

p.0.05=( (paste(" p.05=",format(round({mean(x<0.05/2| x>(1-0.05/2))},3), format="f", nsmall=3), symbols.p05(x),sep=""))),

p.0.01=( (paste(" p.01=",format(round({mean(x<0.01/2| x>(1-0.01/2))},3), format="f", nsmall=3), symbols.p01(x),sep=""))))},method="cross",g=4)

print(x,prn=F)

x<-summary(results.spp.Bernoulli.theta07.Nrandom.thetarandom5.Sc4.conjugate.R [,"var"]~inv.rho+No, fun=function(x){c(

ks.D=( (paste(" ks.D=",format(round(ks.test(x,"punif")$s,3), format="f", nsmall=3), symbols.ks(x),sep=""))),

p.0.05=( (paste(" p.05=",format(round({mean(x<0.05/2| x>(1-0.05/2))},3), format="f", nsmall=3), symbols.p05(x),sep=""))),

p.0.01=( (paste(" p.01=",format(round({mean(x<0.01/2| x>(1-0.01/2))},3), format="f", nsmall=3), symbols.p01(x),sep=""))))},method="cross",g=4)

print(x,prn=F)

## Tables for Text S5 (Scenario 4 results in the Poisson case, comparing spp and ppp )

**#R, spp, conjugate, sigma et N**

No<- results.spp.Poisson.Nrandom.theta1.sigma2.taurandomV1.Sc4.conjugate.R [,"N"]

sigma0<- round(results.spp.Poisson.Nrandom.theta1.sigma2.taurandomV1.Sc4.conjugate.R [,"sigma0"],digits=2)

**x<-summary(**results.spp.Poisson.Nrandom.theta1.sigma2.taurandomV1.Sc4.conjugate.R [,"max"]~sigma0+No, fun=function(x){c(

ks.D=( (paste(" ks.D=",format(round(ks.test(x,"punif")$s,3), format="f", nsmall=3), symbols.ks(x),sep=""))),

p.0.05=( (paste(" p.05=",format(round({mean(x<0.05/2| x>(1-0.05/2))},3), format="f", nsmall=3), symbols.p05(x),sep=""))),

p.0.01=( (paste(" p.01=",format(round({mean(x<0.01/2| x>(1-0.01/2))},3), format="f", nsmall=3), symbols.p01(x),sep=""))))},method="cross",g=4)

print(x,prn=F)

**#R, ppp, conjugate, sigma et N**

No<- results.ppp.Poisson.Nrandom.theta1.sigma2.taurandomV1.Sc4.conjugate.R [,"N"]

sigma0<- round(results.ppp.Poisson.Nrandom.theta1.sigma2.taurandomV1.Sc4.conjugate.R [,"sigma0"],digits=2)

**x<-summary(**results.ppp.Poisson.Nrandom.theta1.sigma2.taurandomV1.Sc4.conjugate.R [,"max"]~sigma0+No, fun=function(x){c(

ks.D=( (paste(" ks.D=",format(round(ks.test(x,"punif")$s,3), format="f", nsmall=3), symbols.ks(x),sep=""))),

p.0.05=( (paste(" p.05=",format(round({mean(x<0.05/2| x>(1-0.05/2))},3), format="f", nsmall=3), symbols.p05(x),sep=""))),

p.0.01=( (paste(" p.01=",format(round({mean(x<0.01/2| x>(1-0.01/2))},3), format="f", nsmall=3), symbols.p01(x),sep=""))))},method="cross",g=4)

print(x,prn=F)

**#R, hsMLp, conjugate, sigma et N**

No<- results.hsMLp.Poisson.Nrandom.theta1.sigma2.taurandomV1.Sc4.conjugate.R [,"N"]

sigma0<- round(results.hsMLp.Poisson.Nrandom.theta1.sigma2.taurandomV1.Sc4.conjugate.R [,"sigma0"],digits=2)

**x<-summary(**results.hsMLp.Poisson.Nrandom.theta1.sigma2.taurandomV1.Sc4.conjugate.R [,"max"]~sigma0+No, fun=function(x){c(

ks.D=( (paste(" ks.D=",format(round(ks.test(x,"punif")$s,3), format="f", nsmall=3), symbols.ks(x),sep=""))),

p.0.05=( (paste(" p.05=",format(round({mean(x<0.05/2| x>(1-0.05/2))},3), format="f", nsmall=3), symbols.p05(x),sep=""))),

p.0.01=( (paste(" p.01=",format(round({mean(x<0.01/2| x>(1-0.01/2))},3), format="f", nsmall=3), symbols.p01(x),sep=""))))},method="cross",g=4)

print(x,prn=F)

## Tables for Text S6

#### #=>A7/Sc2: Poisson, prior predictive

**#R, prior predictive**

results.Poisson.Nrandom.theta1.sigma2.taurandomV1.prior.pred.R<- results.prior.pred.Poisson.Nrandom.theta1.sigma2.taurandomV1.Sc2.conjugate.R

No<- results.Poisson.Nrandom.theta1.sigma2.taurandomV1.prior.pred.R [,"N"]

mult.sigma<- round(results.Poisson.Nrandom.theta1.sigma2.taurandomV1.prior.pred.R[,"musigma0"],digits=2)

x<-summary(results.Poisson.Nrandom.theta1.sigma2.taurandomV1.prior.pred.R [,"mean"]~mult.sigma, fun=function(x){c(

ks.D=( (paste("",format(round(ks.test(x,"punif")$s,3), format="f", nsmall=3), symbols.ks(x),sep=""))),

p.0.05=( (paste("",format(round({mean(x<0.05/2| x>(1-0.05/2))},3), format="f", nsmall=3), symbols.p05(x),sep=""))),

p.0.01=( (paste("",format(round({mean(x<0.01/2| x>(1-0.01/2))},3), format="f", nsmall=3), symbols.p01(x),sep=""))))},method="cross",g=4)

print(x,prn=F)

x<-summary(results.Poisson.Nrandom.theta1.sigma2.taurandomV1.prior.pred.R [,"var"]~mult.sigma, fun=function(x){c(

ks.D=( (paste("",format(round(ks.test(x,"punif")$s,3), format="f", nsmall=3), symbols.ks(x),sep=""))),

p.0.05=( (paste("",format(round({mean(x<0.05/2| x>(1-0.05/2))},3), format="f", nsmall=3), symbols.p05(x),sep=""))),

p.0.01=( (paste("",format(round({mean(x<0.01/2| x>(1-0.01/2))},3), format="f", nsmall=3), symbols.p01(x),sep=""))))},method="cross",g=4)

print(x,prn=F)

x<-summary(results.Poisson.Nrandom.theta1.sigma2.taurandomV1.prior.pred.R [,"skew"]~ mult.sigma, fun=function(x){c(

ks.D=( (paste("",format(round(ks.test(x,"punif")$s,3), format="f", nsmall=3), symbols.ks(x),sep=""))),

p.0.05=( (paste("",format(round({mean(x<0.05/2| x>(1-0.05/2))},3), format="f", nsmall=3), symbols.p05(x),sep=""))),

p.0.01=( (paste("",format(round({mean(x<0.01/2| x>(1-0.01/2))},3), format="f", nsmall=3), symbols.p01(x),sep=""))))},method="cross",g=4)

print(x,prn=F,prnmiss=F)

x<-summary(results.Poisson.Nrandom.theta1.sigma2.taurandomV1.prior.pred.R [,"kurt"]~ mult.sigma, fun=function(x){c(

ks.D=( (paste("",format(round(ks.test(x,"punif")$s,3), format="f", nsmall=3), symbols.ks(x),sep=""))),

p.0.05=( (paste("",format(round({mean(x<0.05/2| x>(1-0.05/2))},3), format="f", nsmall=3), symbols.p05(x),sep=""))),

p.0.01=( (paste("",format(round({mean(x<0.01/2| x>(1-0.01/2))},3), format="f", nsmall=3), symbols.p01(x),sep=""))))},method="cross",g=4)

print(x,prn=F, prnmiss=F)

## Tables for Text S7 (nspp in poisson glm)

### #for Table S1:

table.glm.pois.fixed<-lapply(c("mean","var","skewness","kurt","Za"),function(x,y,z,C,Ct){ c(

ks.D=( (paste(" ks.D=",format(round(ks.test(y[[x]],"punif")$s,3), format="f", nsmall=3), symbols.ks(y[[x]]),sep=""))),

p.0.05=( (paste(" p.05=",format(round({mean(y[[x]]<0.05/2| y[[x]]>(1-0.05/2))},3), format="f", nsmall=3), symbols.p05(y[[x]]),sep=""))),

p.0.01=( (paste(" p.01=",format(round({mean(y[[x]]<0.01/2| y[[x]]>(1-0.01/2))},3), format="f", nsmall=3), symbols.p01(y[[x]]),sep=""))))}, results.Poisson.Nrandom.theta0.fixedtau.glm , results.Poisson.Nrandom.theta0.fixedtau$archive$y$rep.ppi, results.Poisson.Nrandom.theta0.fixedtau.C$archive$model1$gof$p[[1]][[1]][["p.gof.trandom"]], results.Poisson.Nrandom.theta0.fixedtau.C$archive$model1$gof$p[[1]][[1]][["p.gof.trandom"]][ ,results.Poisson.Nrandom.theta0.fixedtau.C$archive$model1$gof$p[[1]][[1]][[1]]["N",]>200])

names(table.glm.pois.fixed)<- c("mean","var","skewness","kurt","Za")

### #for Table S2:

table.glm.pois.random<-lapply(c("mean","var","skewness","kurt","Za"),function(x,y){ c(

ks.D=( (paste(" ks.D=",format(round(ks.test(y[[x]],"punif")$s,3), format="f", nsmall=3), symbols.ks(y[[x]]),sep=""))),

p.0.05=( (paste(" p.05=",format(round({mean(y[[x]]<0.05/2| y[[x]]>(1-0.05/2))},3), format="f", nsmall=3), symbols.p05(y[[x]]),sep=""))),

p.0.01=( (paste(" p.01=",format(round({mean(y[[x]]<0.01/2| y[[x]]>(1-0.01/2))},3), format="f", nsmall=3), symbols.p01(y[[x]]),sep=""))))}, results.Poisson.Nrandom.taurandom.glm)

names(table.glm.pois.random)<- c("mean","var","skewness","kurt","Za")

## Figure 1

**#nmax=5, max, pp2 :**

order.t<- results.spp2.Poisson.Nrandom.theta1.sigma2.taurandomV1.Sc6.conjugate.R [,"N"]

close.screen(all=T)

graphics.off()

x11()

plot(order.t, results.spp2.Poisson.Nrandom.theta1.sigma2.taurandomV1.Sc6.conjugate.R [,"max"],type="n", ylab="power",xlab="N", cex.main=1.5,cex.lab=1.3,cex.axis=1.1,ylim=c(0,1))

#axis(1,at=(0:2)/2,lab= c(".0",".5","1.0"),cex.axis=1.2,las=1)

#axis(2,at=(0:2)/2,lab= c(".0",".5","1.0"),cex.axis=1.2,las=1)

toto<-tapply(results.spp2.Poisson.Nrandom.theta1.sigma2.taurandomV1.Sc6.conjugate.R [,"max"],order.t,function(x){mean(x<0.025|x>0.975)})

lines(as.double(names(toto)),toto,lwd=1.5)

toto<-tapply(results.ppp2.Poisson.Nrandom.theta1.sigma2.taurandomV1.Sc6.conjugate.R [,"max"],order.t,function(x){mean(x<0.025|x>0.975)})

lines(as.double(names(toto)),toto,lty=4,lwd=1.5)

toto<-tapply(results.hsMLp2.Poisson.Nrandom.theta1.sigma2.taurandomV1.Sc6.conjugate.R [,"max"],order.t,function(x){mean(x<0.025|x>0.975)})

lines(as.double(names(toto)),toto,lty=3,lwd=1.5)

abline(0.05,0,lty=2,lwd=1.5)
