# Supplementary material for: A New Calibrated Bayesian Internal Goodness-of-Fit Method: Sampled Posterior p-Values as Simple and General p-Values That Allow Double Use of the Data
Source: PLoS One. 2011 Mar 18;6(3):e14770. doi: 10.1371/journal.pone.0014770 (PMC3060804; doi:10.1371/journal.pone.0014770)
Supplement: Text S9 — R commands to illustrate the discrepancy between the rough deterministic and the stochastic methods to transform α and β to p-values. (0.05 MB DOC) [file pone.0014770.s009.doc]

A New Calibrated Bayesian Internal Goodness-of-Fit Method: Sampled Posterior P-values as Simple and General P-values that Allow Double Use of the Data

Frédéric Gosselin

Cemagref, UR EFNO, F-45290 Nogent-sur-Vernisson, France

E-mail: [frederic.gosselin@cemagref.fr](mailto:frederic.gosselin@cemagref.fr)

*R commands to illustrate the discrepancy between the rough deterministic and the stochastic methods to transform α and β to p-values*

# Aim

The aim is to illustrate the following statements in the main text:

It can be shown that this distribution is the posterior distribution of the underlying p-value once we have observed or sampled $\left(\mathbf{X}_{j}\right)_{j}, \tilde{\theta}$ and $\mathbf{x}_{\mathbf{obs}}$, provided the prior of the p-value is uninformative [Gelman, 2004 (, p. 40) #89468]. In contrast, the use of $\alpha/(\alpha+\beta)$ can result in significant departures from the uniform distribution which would be due to the calculation method and not to the underlying p-value; this would especially occur with a low number of replicated data $\left(\mathbf{X}_{j}\right)_{j}$ or to estimate the tails of the uniform distribution.

We here assume that the **X** are sampled from exactly the same distribution as $\mathbf{x}_{\mathbf{obs}}$, that both are continuous and that we use a test statsitic function so that sampling first $\mathbf{x}_{\mathbf{obs}}$ and then comparing it to *n* replicated **X** through the test statistic function is equivalent to sampling first a value p in [0;1] according to the uniform distribution and then sampling from a binomial distribution with parameter p and *N*=*n*.

## # case with 100 replicated X values:

sample<-10000

nx<-100

set.seed(1)

toto<-rbinom(sample,nx,runif(sample))

p.det<-toto/nx

ks.test(p.det,"punif")

pbinom(sum(p.det<0.0501),sample,0.0501)

pbinom(sum(p.det<0.05),sample,0.05)

pbinom(sum(p.det<0.0101),sample,0.0101)

pbinom(sum(p.det<0.01),sample,0.01)

pbinom(sum(p.det<0.001),sample,0.001)

pbinom(sum(p.det<0.0001),sample,0.0001)

p.stoch<-rbeta(sample,toto+1,nx-toto+1)

ks.test(p.stoch,"punif")

pbinom(sum(p.stoch<0.0501),sample,0.0501)

pbinom(sum(p.stoch<0.05),sample,0.05)

pbinom(sum(p.stoch<0.0101),sample,0.0101)

pbinom(sum(p.stoch<0.01),sample,0.01)

pbinom(sum(p.stoch<0.001),sample,0.001)

pbinom(sum(p.stoch<0.0001),sample,0.0001)

## # case with 1000 replicated X values:

sample<-10000

nx<-1000

set.seed(1)

toto<-rbinom(sample,nx,runif(sample))

p.det<-toto/nx

ks.test(p.det,"punif")

pbinom(sum(p.det<0.0501),sample,0.0501)

pbinom(sum(p.det<0.05),sample,0.05)

pbinom(sum(p.det<0.0101),sample,0.0101)

pbinom(sum(p.det<0.01),sample,0.01)

pbinom(sum(p.det<0.00501),sample,0.00501)

pbinom(sum(p.det<0.00101),sample,0.00101)

pbinom(sum(p.det<0.001),sample,0.001)

pbinom(sum(p.det<0.0001),sample,0.0001)

p.stoch<-rbeta(sample,toto+1,nx-toto+1)

ks.test(p.stoch,"punif")

pbinom(sum(p.stoch<0.0501),sample,0.0501)

pbinom(sum(p.stoch<0.05),sample,0.05)

pbinom(sum(p.stoch<0.0101),sample,0.0101)

pbinom(sum(p.stoch<0.01),sample,0.01)

pbinom(sum(p.stoch<0.00501),sample,0.00501)

pbinom(sum(p.stoch<0.00101),sample,0.00101)

pbinom(sum(p.stoch<0.001),sample,0.001)

pbinom(sum(p.stoch<0.0001),sample,0.0001)
